# Integrative Pan-Cancer Mapping of Proteasome Dependency Prioritizes PSMB5 and PSMB6 as Context-Dependent Vulnerability Biomarkers Linked to Immune Context

**DOI:** 10.3390/molecules31111954

**Published:** 2026-06-04

**Authors:** Jeong Han Kim, Hansol Park, Hyo Jin Kim, Myoung-Eun Han, Dongjun Lee, Sik Yoon, Sae-Ock Oh

**Affiliations:** 1 Department of Anatomy, School of Medicine, Pusan National University, 49 Busandaehak-ro, Yangsan 50612, Republic of Koreasikyoon@pusan.ac.kr (S.Y.); 2 Research Center for Molecular Control for Cancer Cell Diversity, Pusan National University, Yangsan 50612, Republic of Korea; 3 Department of Convergence Medicine, School of Medicine, Pusan National University, Yangsan 50612, Republic of Korea

**Keywords:** proteasome subunits, biomarker, molecular-targeted therapy, PSMB5, PSMB6, cancer dependency, immune infiltration, pan-cancer analysis

## Abstract

The prioritization of biomarkers that inform molecular-targeted cancer research remains challenging because tumor vulnerabilities are context-dependent. The ubiquitin–proteasome system is essential for cancer cell survival; however, the functional and biomarker-level relevance of individual proteasome subunits has not been systematically defined across cancer types. In this study, we performed an integrative pan-cancer analysis to prioritize proteasome subunits that function as context-dependent vulnerability biomarkers. We analyzed proteasome subunits and proteasome-associated genes across 54 cancer types by integrating large-scale CRISPR (*n* = 1178 cell lines) and RNAi (*n* = 707 cell lines) dependency datasets with transcriptomic, survival, immune infiltration, and co-essentiality network analyses. PSMB5 and PSMB6 were prioritized as robust cross-platform and cross-lineage dependency biomarkers, exhibiting reproducible and selective vulnerability patterns across diverse malignancies. Their dependency strength was quantitatively associated with immune-related signaling pathways, including MHC and interferon responses, and inversely correlated with key immune regulatory genes such as NLRC5 and IRF1. Co-essentiality network analysis revealed modular functional organization of proteasome-associated genes, supporting context-dependent roles rather than uniform essentiality. Importantly, the association between proteasome subunits and tumor immune context was externally validated through meta-analysis across 24 independent hepatocellular carcinoma cohorts, demonstrating reproducible correlations with CD4-positive T cell, CD8 T cell, and macrophage infiltration signatures. Functional validation further confirmed that siRNA-mediated knockdown of *PSMB5* and *PSMB6* significantly impaired proliferation across multiple hepatocellular carcinoma cell lines. Collectively, this study prioritizes PSMB5 and PSMB6 as consistently associated functional biomarkers that integrate genetic dependency and immune context, providing a data-driven framework for stratifying proteasome-targeted therapeutic strategies across cancers.

## 1. Introduction

The ubiquitin–proteasome system (UPS) is responsible for the degradation of approximately 80% of intracellular proteins, playing a pivotal role in maintaining cellular proteostasis [1,2]. The ubiquitination process involves a cascade of enzymatic steps, namely, activation by E1 ubiquitin-activating enzymes, conjugation by E2 ubiquitin-conjugating enzymes, and ligation by E3 ubiquitin ligases, which confer substrate specificity [2]. Polyubiquitinated proteins are subsequently recognized and degraded by the 26S proteasome complex [1,3]. The 26S proteasome is a multi-subunit complex comprising a 20S catalytic core particle and one or two 19S regulatory particles. The 20S core particle consists of four stacked rings forming a barrel-like structure: two outer rings composed of seven α subunits (PSMA1–PSMA7) and two inner rings composed of seven β subunits (PSMB1–PSMB7). The β subunits harbor proteolytic activities, including chymotrypsin-like, trypsin-like, and caspase-like activities.

The 19S regulatory particle is subdivided into a base and a lid [4,5]. The base contains six AAA ATPases (PSMC1–PSMC6) and non-ATPase subunits such as PSMD1 and PSMD2, which are involved in substrate recognition and unfolding *(5).* The lid comprises non-ATPase subunits including PSMD3, PSMD6, PSMD7, PSMD8, PSMD11, PSMD12, PSMD13, PSMD14, and SHFM1 (also known as DSS1), which are implicated in deubiquitination and substrate processing. Upon recognition of polyubiquitinated substrates by ubiquitin receptors within the 19S regulatory particle, the substrates are deubiquitinated, unfolded by the ATPase activity of the base, and translocated into the 20S core particle for proteolytic degradation [2,3]. This orchestrated process ensures the regulated turnover of proteins, thereby maintaining cellular homeostasis.

Throughout evolution, the proteasome has diversified, with the emergence of tissue-specific subunits facilitating specialized functions across different cell types. Such variations enable adaptations to specific physiological demands, including immune surveillance, neuronal activity, and metabolic regulation [6]. A notable example is the immunoproteasome, predominantly expressed in immune cells. In response to pro-inflammatory cytokines like interferon-gamma, the standard catalytic subunits β1 (PSMB6), β2 (PSMB7), and β5 (PSMB5) are replaced by β1i (PSMB9), β2i (PSMB10), and β5i (PSMB8), respectively. This substitution enhances the generation of antigenic peptides optimized for presentation by major histocompatibility complex (MHC) class I molecules, thereby bolstering adaptive immune responses [7]. Proteasome composition is dynamic and can shift in response to cellular stress, aging, and pathological conditions such as neurodegenerative diseases and cancer [8]. Under oxidative stress, the 26S proteasome complex undergoes disassembly, leading to the activation of the 20S proteasome. Unlike the 26S proteasome, the 20S variant operates independently of ATP and ubiquitin, efficiently degrading oxidatively damaged or misfolded proteins [8,9]. This mechanism is crucial for cellular defense against oxidative damage and contributes to the maintenance of proteostasis under stress conditions.

The proteasome complex is integral to cancer biology, primarily through its role in protein degradation and regulation of various cellular processes [10]. Cancer cells often upregulate components of the ubiquitin–proteasome system (UPS) to degrade proteins that would otherwise inhibit cell cycle progression or promote apoptosis, thereby facilitating uncontrolled growth and survival [11]. Proteasome inhibitors, such as bortezomib and carfilzomib, have been developed and are utilized in treating multiple myeloma and other malignancies [12]. These inhibitors induce apoptosis in cancer cells by causing the accumulation of misfolded proteins, triggering endoplasmic reticulum (ER) stress and the unfolded protein response (UPR), leading to the activation of intrinsic apoptotic pathways [13]. Additionally, they can stabilize pro-apoptotic proteins and inhibit survival pathways such as NF-κB [12]. However, resistance to proteasome inhibitors can develop, necessitating a deeper understanding of the underlying mechanisms to enhance treatment strategies [14].

Different cancer types exhibit distinct patterns of genetic alterations, such as mutations, amplifications, overexpression, and co-expression of proteasome subunits [15]. These variations may be associated with proteasome structural diversity, differential assembly of its subunits, and variations in proteasome regulation, enabling cancer cells to adapt to diverse microenvironments. Notably, the functional consequences of proteasome perturbation are not universally cytotoxic. Previous studies have shown that partial inhibition or knockdown of specific 19S regulatory subunits can, under certain conditions, promote cellular adaptation or confer protective effects, depending on the cellular context and stress environment [16,17]. Moreover, the expression levels and genetic alterations of individual proteasome subunits have been linked to prognostic outcomes across various cancers [15]. For instance, adrenocortical carcinoma (ACC), lower-grade glioma (LGG), liver hepatocellular carcinoma (LIHC), and uveal melanoma (UVM) have shown consistently high proteasome activity due to overexpression of proteasome genes, suggesting that proteasome inhibition could be beneficial in these contexts [15]. Given the diversity of proteasome alterations among different cancer types, it is crucial to investigate the individual effects of proteasomal subunits within each cancer type to optimize proteasome-targeted therapies.

To investigate the individual contributions of proteasomal subunits to cancer cell viability, we analyzed dependency scores derived from genome-wide CRISPR-based and RNAi-based screens available through the DepMap portal. To ensure a comprehensive and biologically interpretable analysis, we systematically defined a curated set of proteasome-related genes. These components were categorized into four functional groups: (i) core structural subunits of the 20S core particle and 19S regulatory particle, (ii) regulatory or accessory factors (e.g., *PSMD5*, *PSMD9*, and *PSMD10*), (iii) proteasome assembly factors (e.g., *PSMG1–4* and *POMP*), and (iv) proteasome-associated enzymes, including deubiquitinating enzymes such as *USP14* and *UCH37*, as well as proteasome-associated stabilizing factors such as ECPAS. In particular, inclusion of assembly factors (*PSMG1–4* and *POMP*), regulatory particles (*PSME1–4*), and associated stabilizing or inhibitory proteins (e.g., *PSMF1* and *ECPAS*) was intended to capture both structural and regulatory layers of proteasome function in a unified analytical framework. In addition, proteasome-associated ubiquitin receptors such as *ADRM1* were included to better capture functional interactions within the proteasome complex. Components such as *PSME1–4* were incorporated to reflect regulatory proteasome variants, including alternative proteasome activators. This classification was introduced to avoid ambiguity in gene selection and to distinguish canonical subunits from functionally associated components. Importantly, this study does not treat all included genes as equivalent structural subunits but rather as components contributing to proteasome-related functional networks.

This analysis enabled comparative prioritization of proteasome-related genes across cancer types. *PSMB5* and *PSMB6* showed reproducible dependency-associated patterns across available CRISPR and RNAi datasets and were therefore selected for downstream analyses. However, these findings should be interpreted as a data-driven prioritization framework rather than definitive proof that *PSMB5* and *PSMB6* are functionally unique among proteasome subunits. Because proteasome components function within a multi-subunit complex and perturbation efficiency may vary across genes and platforms, direct side-by-side experimental comparison would be required to establish subunit-specific uniqueness. To support the development of subtype-specific therapeutic strategies, we further assessed contextual molecular characteristics associated with each proteasomal subunit, including somatic mutations, copy number alterations, transcriptomic overexpression, and associated signaling pathways. These integrated analyses provide a framework for prioritizing proteasome-targeted interventions tailored to the molecular landscape of individual cancer types.

## 2. Results

### 2.1. Pan-Cancer Analysis of the Dependency of Individual Proteasomal Subunits

To examine the effect of each subunit on each cancer type, we analyzed Chronos scores, which represent the effects of CRISPR-mediated knockouts, and DEMETER2 scores, which represent the effects of RNAi-mediated knockdowns, for each subunit in the DepMap portal. Chronos scores were available for 48 subunits across 54 cancer types (Supplementary Table S1), and DEMETER2 scores were available for 47 subunits across 46 cancer types. Subunits or cancer types lacking relevant data were excluded from the analysis. The inclusion of proteasome-associated components beyond canonical subunits, such as assembly factors (*PSMG1–4* and *POMP*), regulatory activators (*PSME1–4*), and associated proteins (e.g., *PSMF1* and *ECPAS*), was intended to capture functional diversity within proteasome regulation rather than to define structural equivalence among all included genes. In addition, structural flexibility of the proteasome should be considered when interpreting subunit dependency; for example, *PSMA7* has been reported to substitute for *PSMA4* under certain conditions [18].

**Figure 1:**
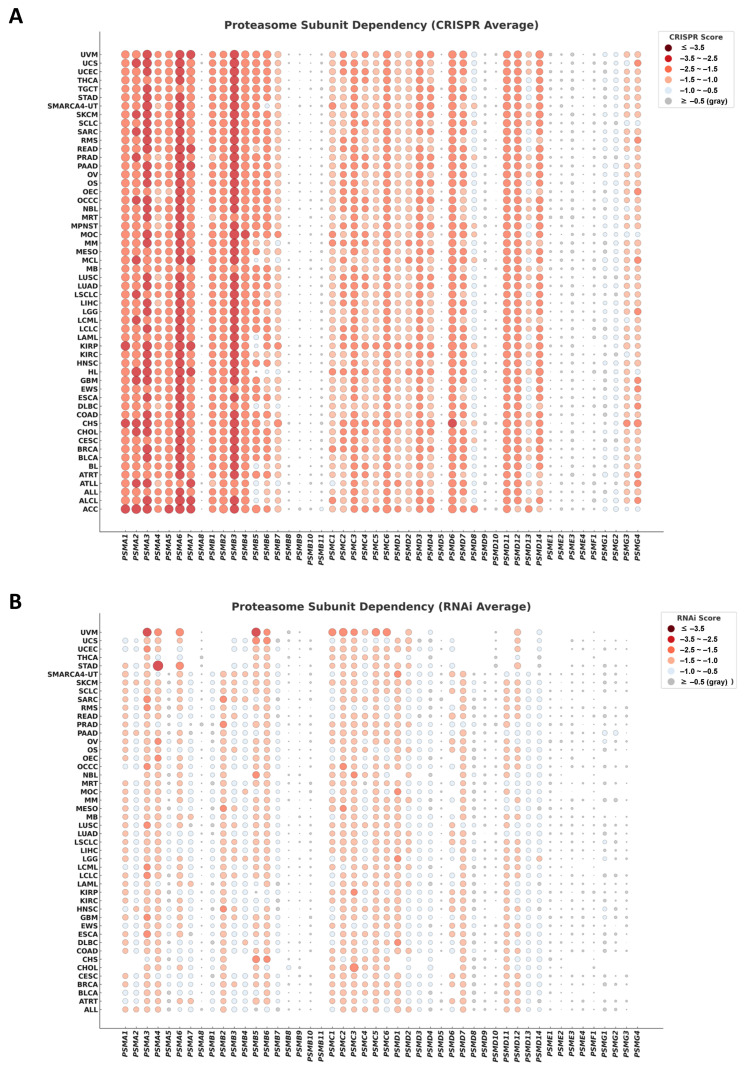
**Dependency status of proteasome-related components across cancer types based on CRISPR and RNAi perturbations.** (**A**) Bubble plot representing average CRISPR-based dependency scores (Chronos) per cancer type for each proteasome-related gene across 54 cancer types. (**B**) Bubble plot showing average RNAi-based dependency scores (DEMETER2) across 46 cancer types. The analyzed gene set includes not only canonical proteasome subunits but also proteasome-associated components, including regulatory/accessory factors (e.g., *PSMD5*, *PSMD9*, *PSMD10*), assembly factors (e.g., *PSMG1–4* and *POMP*), and associated enzymes such as deubiquitinating enzymes (*USP14* and *UCH37*). Dependency scores represent functional perturbation effects and do not directly measure complete gene knockout or knockdown efficiency. These effects may be influenced by genomic context, including copy number variation. Bubble size and color indicate dependency strength (red: stronger dependency; blue: weaker dependency).

In the Chronos score analysis, 33 subunits showed significant average dependency across cancer types (Chronos score < −1), while 15 subunits did not show significant dependency in any cancer type (Figure 1A). Although most subunits were classified as common essential genes (significant dependency in ≥90% of 1178 cell lines), 15 subunits (*PSMB5*, *PSMB6*, *PSMB7*, *PSMD1*, *PSMD10*, *PSMD13*, *PSMD4*, *PSMD8*, *PSMD9*, *PSME3*, *PSMF1*, *PSMG1*, *PSMG2*, *PSMG3*, and *PSMG4*) showed strong context-dependent dependency patterns (Figure 1A, Supplementary Table S2). Among these, *PSMB6* exhibited the strongest average dependency in malignant peripheral nerve sheath tumor (*MPNST*; score = −2.388).

In the DEMETER2 score analysis, 24 subunits showed significant average dependency across cancer types (DEMETER2 score < −1), whereas 23 others showed no significant dependency in any cancer type (Figure 1B). Only *PSMD1* was categorized as a common essential gene, showing significant dependency in ≥90% of the 707 analyzed cell lines (Supplementary Table S3). The remaining 31 subunits showed strongly selective dependency (Supplementary Table S3). Among these, PSMA4 displayed the strongest average dependency in stomach adenocarcinoma (STAD; score = −3.450). Notably, 9 subunits (*PSMB5*, *PSMB6*, *PSMB7*, *PSMD4*, *PSMD8*, *PSMD10*, *PSMD13*, *PSMG1*, and *PSMG2*) showed strongly selective dependency in both the Chronos and DEMETER2 analyses.

**Figure 2:**
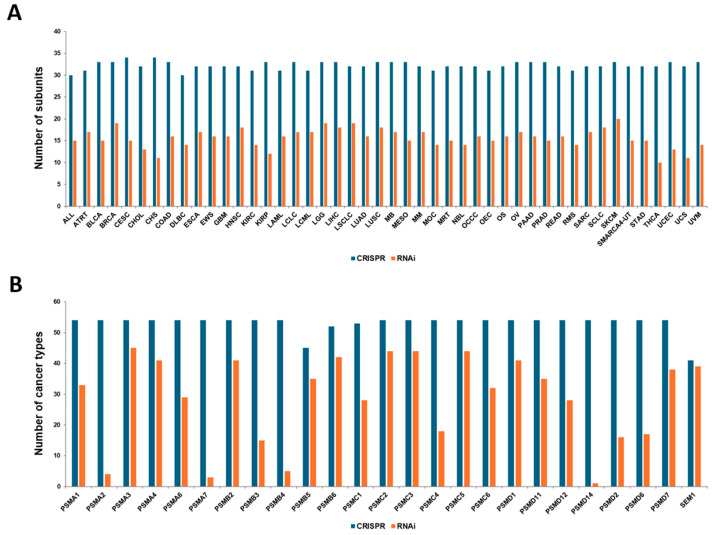
**Frequency of strong dependency by cancer type and proteasomal subunit.** (**A**) Bar plot displaying the number of proteasomal subunits exhibiting strong lethality (Chronos or DEMETER2 score < −1) in 54 cancer types. Each bar represents a cancer type, with blue indicating CRISPR-derived scores (Chronos) and orange indicating RNAi-derived scores (DEMETER2). (**B**) Bar plot showing the number of cancer types in which each individual subunit is strongly lethal (score < −1). Blue bars represent Chronos data; orange bars represent DEMETER2 data. Subunits or cancer types lacking dependency data were excluded prior to aggregation. Therefore, zero values indicate that dependency data were available but no subunits met the threshold, rather than representing missing information.

To assess the sensitivity of each cancer type to perturbation of proteasomal subunits, we counted the number of subunits with dependency scores < −1 per cancer type. Prior to this aggregation, subunits or cancer types lacking dependency data were excluded from the analysis to avoid conflating missing values with true absence of dependency. Therefore, values of zero represent cases in which data were available but no subunits met the dependency threshold, rather than cases of missing information. Most cancer types showed a similar number of dependent subunits. However, the average number of significantly dependent subunits per cancer type in DEMETER2 data (mean = 14.76) was approximately half of that in the Chronos data (mean = 31.24) (Figure 2A).

Next, we assessed how frequently each subunit exhibited dependency across cancer types. Based on Chronos scores, 28 subunits showed dependency in more than 50 cancer types, while 15 subunits did not show dependency in any type (Figure 2B). Based on DEMETER2 scores, only 8 subunits exhibited dependency in more than 40 cancer types. Among subunits classified as strongly selective in both datasets, *PSMB5* and *PSMB6* were among the most consistently prioritized genes across multiple cancer types (Figure 2B). Therefore, further analysis was mainly focused on *PSMB5* and *PSMB6,* as these subunits exhibited the most consistent and reproducible dependency patterns across both CRISPR and RNAi datasets and across multiple cancer types. This prioritization was based on a comparative assessment across all proteasome subunits, in which *PSMB5* and *PSMB6* consistently ranked among the top candidates in terms of cross-cancer dependency frequency and effect size (Figure 1 and Figure 2). Importantly, these subunits should not be interpreted as uniquely essential components of the proteasome, but rather represented the most consistently associated candidates across independent datasets, supporting their prioritization for downstream analyses.

It should be noted that dependency scores derived from CRISPR (Chronos) and RNAi (DEMETER2) datasets reflect functional perturbation effects rather than direct measures of complete gene knockout or knockdown efficiency. These effects may be influenced by underlying genomic features, including copy number variation and incomplete perturbation efficiency, which should be considered when interpreting dependency strength across cancer types.

Expression status of each proteasomal subunit were already reported [15], and we re-evaluated them (Supplementary Figure S1). Although most cancer types showed the overexpression of proteasomal subunits, some cancer types including uterine carcinosarcoma (UCS), pancreatic adenocarcinoma (PAAD) and OV, showed a relatively higher expression pattern (Supplementary Figure S1).

### 2.2. Functional Validation of PSMB5 and PSMB6 Dependency in Hepatocellular Carcinoma Cells

Based on the pan-cancer dependency analyses (Figure 1 and Figure 2), *PSMB5* and *PSMB6* were prioritized as reproducible dependency-associated candidates. Hepatocellular carcinoma (HCC) was selected for downstream functional validation and cohort-level analyses based on several considerations. First, our pan-cancer results indicated that proteasome dependency is closely linked to immune-related pathways, and HCC represents a tumor type in which immune context is highly relevant. Second, HCC provides a well-characterized panel of cell line models suitable for experimental validation. Third, the availability of multiple independent transcriptomic cohorts enabled robust meta-analysis of immune infiltration-associated features. Therefore, HCC was used as a representative model to examine whether depletion of these prioritized candidates affects cell proliferation and to evaluate their association with immune context in patient cohorts.

**Figure 3:**
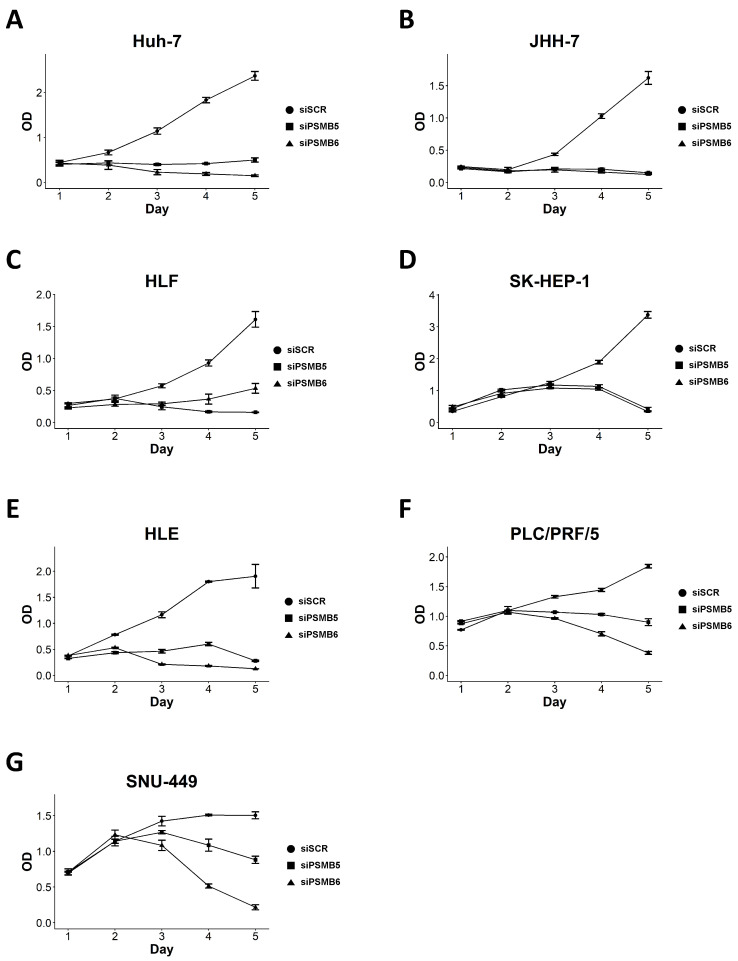
**Effects of proteasome subunit knockdown on cell proliferation in cancer cells.** Cell proliferation was assessed using the Ez-Cytox assay following siRNA-mediated knockdown of *PSMB5* and *PSMB6*. Cells were transfected with siRNAs targeting *PSMB5* or *PSMB6*, or with a non-targeting control (siSCR), and subsequently seeded for proliferation analysis. Cell viability was measured at the indicated time points (Day 1–5) and expressed as optical density (OD). (**A**) Huh7, (**B**) JHH7, (**C**) HLF, (**D**) SK-HEP-1, (**E**) HLE, (**F**) PLC/PRF/5, and (**G**) SNU-449 cells. Data are presented as mean ± SD from independent experiments.

To validate these findings, we performed siRNA-mediated knockdown in hepatocellular carcinoma (HCC) cells and monitored cell proliferation over time. *PSMB5* and *PSMB6* siRNA efficiently knocked down their expression in liver cancer cells (Supplementary Figure S2). Across all examined cell lines, including Huh7, JHH7, HLF, SK-HEP-1, HLE, PLC/PRF/5, and SNU-449, control cells (siSCR) showed a progressive increase in proliferation from Day 1 to Day 5, whereas knockdown of *PSMB5* or *PSMB6* consistently attenuated proliferative capacity (Figure 3A–G). Although the magnitude of inhibition varied among cell lines, the overall trend was uniform, with reduced growth relative to control conditions. These results provide functional support for the dependency-associated patterns observed in the in silico analyses and demonstrate that perturbation of *PSMB5* or *PSMB6* suppresses proliferative capacity in hepatocellular carcinoma cells. However, because these experiments measure proliferation/metabolic activity rather than endpoint-matched viability or dependency, they should not be interpreted as direct confirmation of DepMap-derived dependency scores or as proof of functional uniqueness relative to other proteasome subunits.

### 2.3. Systematic Identification of Molecular Features Associated with Proteasomal Subunit Dependency

To identify molecular features associated with proteasomal subunit dependency, we systematically analyzed correlations between dependency scores and gene-level features, including gene expression, copy number variation, and mutation status, across cancer cell lines. CRISPR-based associations were evaluated using Chronos scores, whereas RNAi-based associations were evaluated using DEMETER2 scores. Associations between each proteasome-related gene and its own molecular feature were excluded to avoid trivial self-correlations.

**Figure 4:**
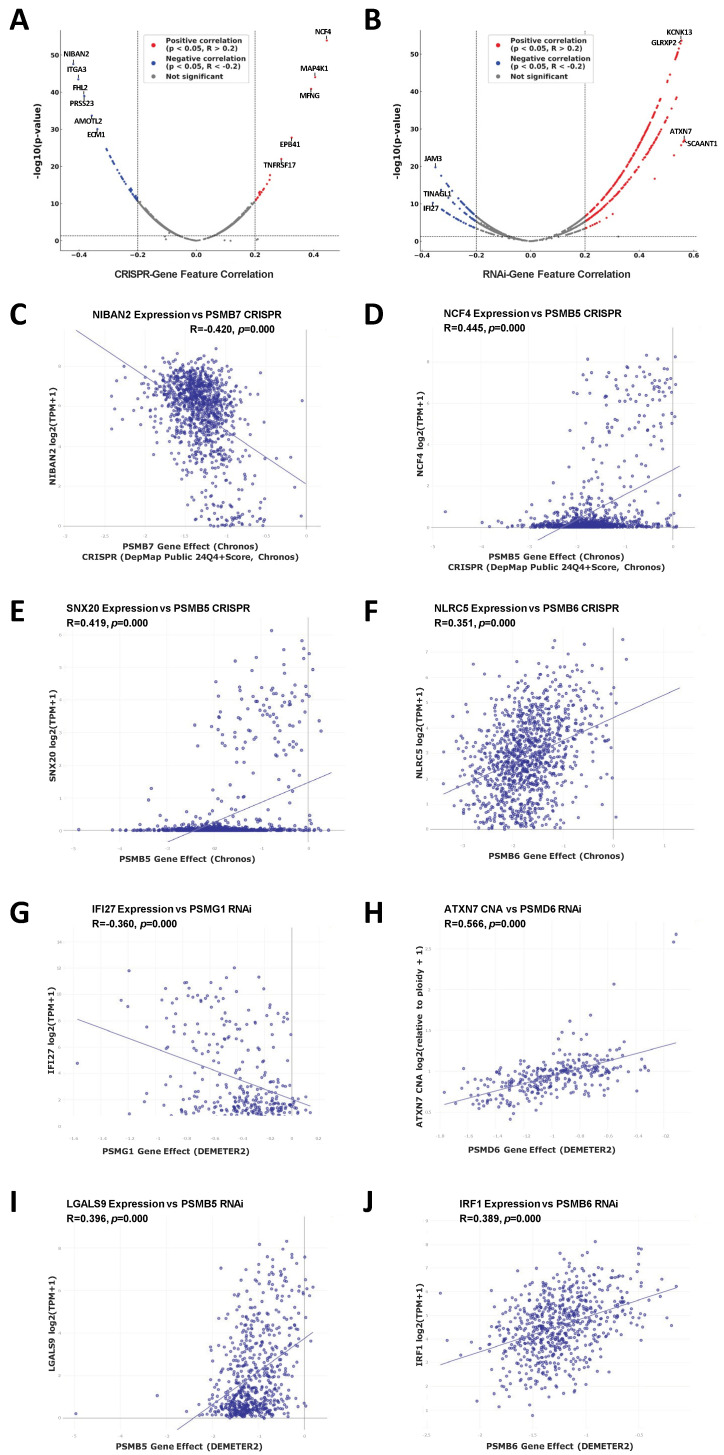
**Correlation between proteasomal subunit dependencies and gene-level features.** (**A**,**B**) Volcano plots illustrating correlations between dependency scores of each subunit (CRISPR, (**A**); RNAi, (**B**)) and other gene features. Red dots indicate significant correlations (*p* < 0.05 and |correlation| > 0.2); dashed lines denote ±0.2 thresholds. Labeled points highlight representative genes selected from the full set of significant associations (see Supplementary Tables S4 and S5). (**C**–**J**) Representative scatter plots with regression lines for selected gene-level associations: (**C**) *PFH11* expression–*PSMB5* (CRISPR), (**D**) NCF4 expression–*PSMB5* (CRISPR), (**E**) *IL3RA* expression and PSMB5 (CRISPR) (**F**) *NLRC5* expression–*PSMB6* (CRISPR), (**G**) *STAT6* expression–*PSMB5* (RNAi), (**H**) *ALOX15* copy number alteration–*PSMB6* (RNAi), (**I**) *LGALS9* expression–*PSMB5* (RNAi), (**J**) *IRF1* expression–*PSMB6* (RNAi). Pearson correlation coefficients, *p*-values and regression lines are presented inside each graph.

In the CRISPR-based analysis, volcano plot analysis revealed both negatively and positively correlated gene-level features associated with proteasomal subunit dependency (Figure 4A). Representative negatively correlated genes included *NIBAN2*, *TNFRSF12A*, *NCKAP1*, *GNG12*, *LMNA*, *FOSL1*, *ITGA3*, *FAM114A1*, and *LAPTM4B*. In contrast, representative positively correlated genes included *NCF4*, *SNX20*, *NLRC5*, *IL12RB1*, *WAS*, *MYO1G*, *TAP1*, *IKZF1*, *ZC3H12D*, and *ARHGAP30* (Figure 4A). Notably, many of the positively correlated genes are related to immune regulation, antigen presentation, cytokine signaling, or immune-cell-associated transcriptional programs. These results suggest that proteasomal subunit dependency is associated with immune-related molecular states rather than being linked only to proteostasis-related genes.

Representative scatter plots further illustrate these associations. These plots are provided for visualization of Pearson correlation patterns and do not imply a specific mechanistic or linear biological relationship. *PSMB5* dependency showed positive correlations with *PFH11* expression (R = 0.346, *p* = 0.000; Figure 4C), *NCF4* expression (R = 0.417, *p* = 0.000; Figure 4D), and *IL3RA* expression (R = 0.296, *p* = 0.000; Figure 4E). *PSMB6* dependency also correlated with *NLRC5* expression (R = 0.354, *p* = 0.000; Figure 4F). Because NLRC5 is a major regulator of MHC class I antigen presentation, this association supports a potential link between proteasome dependency and antigen-presentation-related immune programs.

In the RNAi-based analysis, DEMETER2-derived correlations also identified multiple gene-level features associated with proteasomal subunit dependency (Figure 4B). Representative negatively correlated genes included *EDA2R*, *CDKN1A*, *MAPK8IP1*, *SHISA4*, *ERRFI1*, *RPS27L*, *PTCHD4*, *JAM3*, and *HLA*-related genes. Representative positively correlated genes included *FAM185A*, *KCNK13*, *EFCAB11*, *NRDE2*, *TDP1*, *AP2M1*, *FBXL13*, and *TTC7B* (Figure 4B). These results indicate that dependency-associated molecular features are not restricted to CRISPR-based perturbation data but are also observed in an independent RNAi-based platform.

Consistent with this observation, representative DEMETER2 scatter plots showed positive correlations between *PSMB5* dependency and *STAT6* expression (R = 0.299, *p* = 0.000; Figure 4G), between *PSMB5* dependency and *LGALS9* expression (R = 0.395, *p* = 0.000; Figure 4H), and between *PSMB6* dependency and *IRF1* expression (R = 0.391, *p* = 0.000; Figure 4I). In addition, *PSMB6* dependency was associated with ALOX15 copy number alteration (R = 0.279, *p* = 0.000; Figure 4J). Importantly, *STAT6*, *LGALS9*, *IRF1*, and *NLRC5* are closely related to immune signaling, interferon response, antigen presentation, or immune regulatory pathways.

Taken together, these CRISPR- and RNAi-based analyses indicate that proteasome dependency is systematically associated with immune-related gene expression programs. The highlighted genes in Figure 4A,B are therefore not arbitrary examples, but representative features of broader gene-level associations summarized in Supplementary Tables S4 and S5.

### 2.4. Pathway-Level Characterization of Proteasomal Subunit Dependency

To determine whether the gene-level associations identified above reflect broader biological programs, we next analyzed correlations between proteasomal subunit dependency scores and ssGSEA-derived pathway activity scores across cancer cell lines (Supplementary Table S6). This analysis was performed separately using CRISPR-based Chronos scores and RNAi-based DEMETER2 scores.

**Figure 5:**
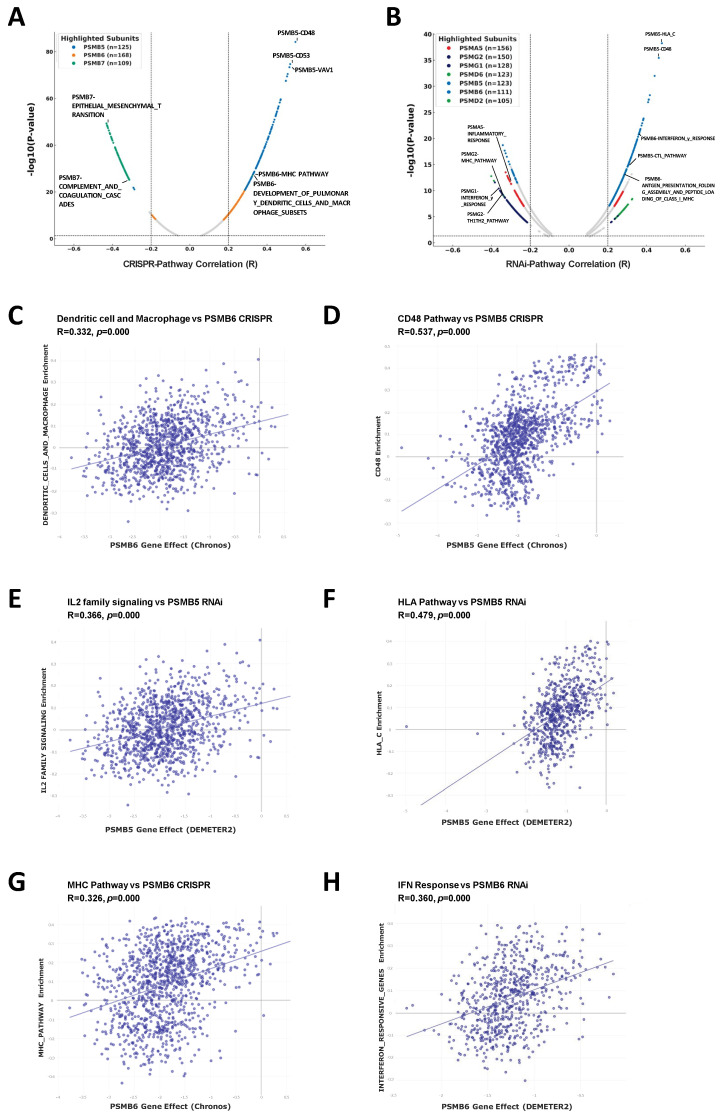
**Correlation between proteasomal subunit dependencies and pathway activity.** (**A**,**B**) Volcano plots showing correlations between ssGSEA pathway enrichment scores and dependency scores for each proteasomal subunit based on CRISPR (**A**) or RNAi (**B**) perturbations. Subunits are color-coded by family. Labeled points highlight representative genes selected from the full set of significant associations (see Supplementary Tables S7 and S8). (**C**–**H**) Representative scatter plots with regression lines for specific gene–pathway relationships: (**C**) Dendritic cell and macrophage Pathway–*PSMB6*(CRISPR), (**D**) CD48 Pathway–*PSMB5* (CRISPR), (**E**) IL2-*PSMB5* (RNAi), (**F**) HLA Pathway–*PSMB5* (RNAi), (**G**) MHC Pathway–*PSMB6* (CRISPR), (**H**) IFN Response–*PSMB6* (RNAi). Pearson correlation coefficients and *p*-values are presented inside each graph.

In the CRISPR-based pathway analysis, volcano plot analysis showed that several pathways were significantly associated with dependency on specific proteasome subunits (Figure 5A). Positively correlated pathways included *PSMB5*–CD48, *PSMB5*–CD53, *PSMB6*–MHC pathway, and *PSMB7*–primary immunodeficiency pathway associations (Figure 5A). These results indicate that proteasome dependency is linked not only to protein homeostasis but also to immune-related and cell–cell interaction pathways.

Representative scatter plots further supported these pathway-level associations. PSMB6 dependency was positively correlated with the dendritic cell and macrophage pathway (R = 0.332, *p* = 0.000; Figure 5C), while *PSMB5* dependency was strongly associated with the CD48 pathway (R = 0.537, *p* = 0.000; Figure 5D). In addition, *PSMB6* dependency showed a positive correlation with the MHC pathway (R = 0.326, *p* = 0.000; Figure 5G). Because CD48, dendritic cell/macrophage-related pathways, and MHC signaling are directly connected to immune regulation and antigen presentation, these results provide pathway-level support for the immune-associated gene-level findings shown in Figure 4.

In the RNAi-based DEMETER2 pathway analysis, multiple immune- and signaling-related pathways were again associated with proteasomal subunit dependency (Figure 5B). Positively correlated pathway associations included *PSMB5*–HLA-C, *PSMB5*–CD48, *PSMB6*–interferon response, *PSMA5*–APEX1, PSMD6–translation initiation reaction, and additional *PSMG1*- or PSMD2-associated pathways. Negatively correlated pathway associations included *PSMB5*–MAPT, *PSMB5*–RTN1, *PSMA5*–EGFR-related interactions, *PSMD6*–tight junction interactions, and *PSMG1*–interferon alpha response (Figure 5B). These findings show that immune-related pathways, including HLA signaling, CD48 signaling, and interferon response, are recurrently associated with proteasome dependency across perturbation platforms.

Representative DEMETER2 scatter plots showed that *PSMB5* dependency was correlated with IL2 family signaling (R = 0.366, *p* = 0.000; Figure 5E) and HLA pathway activity (R = 0.479, *p* = 0.000; Figure 5F). *PSMB6* dependency was also correlated with interferon response activity (R = 0.360, *p* = 0.000; Figure 5H). These results are consistent with the CRISPR-based findings and indicate that the association between proteasome dependency and immune-related pathway activity is reproducible across independent perturbation datasets.

Collectively, the pathway-level analysis demonstrates that proteasomal subunit dependency is embedded within broader immune-associated signaling networks, particularly those related to antigen presentation, HLA/MHC signaling, interferon response, cytokine signaling, and immune-cell-associated pathways. The full pathway-level results are provided in Supplementary Tables S7 and S8. These findings provide a functional rationale for the subsequent analysis of proteasome subunit expression and immune cell infiltration in patient tumor tissues.

### 2.5. Association of Proteasomal Subunit Activity with Immune Activity of Cancer Type

**Figure 6:**
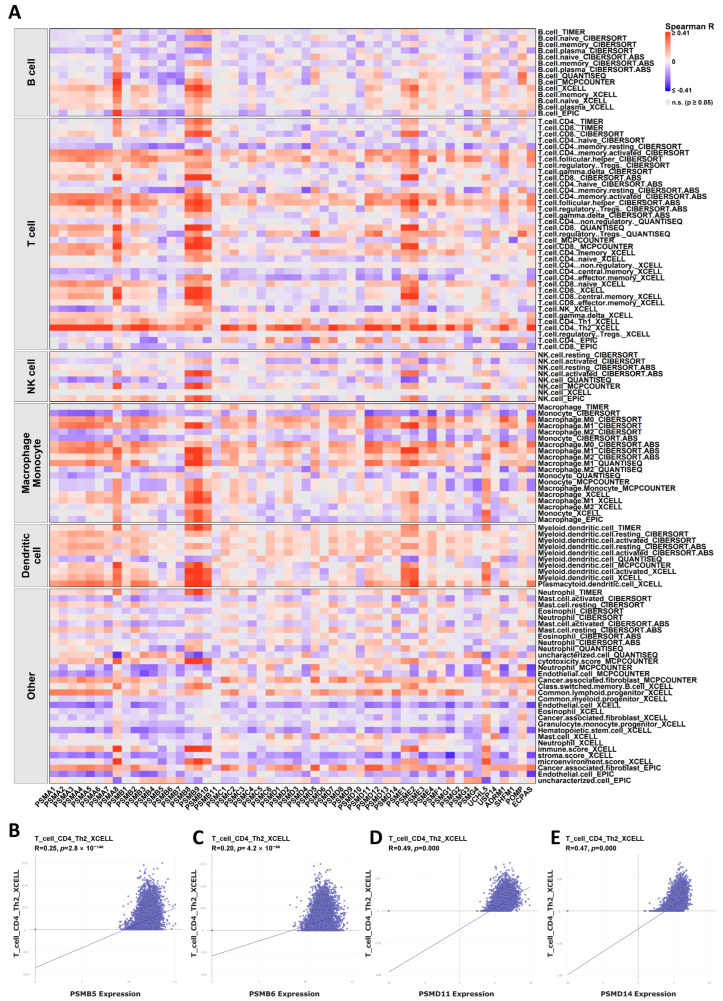
**Correlation between expression of proteasome subunits and immune cell infiltration across cancer types.** (**A**) Heatmap displaying Pearson correlation coefficients between the expression levels of individual proteasome subunits and infiltration scores of immune cell subsets estimated from TCGA pan-cancer data. Each column represents immune cell types grouped by lineage (B cell, T cell, NK cell, macrophage/monocyte, dendritic cell, others), and each row represents proteasome subunits. Red and blue indicate positive and negative correlations, respectively. (**B**–**E**) Representative scatter plots showing the correlation between selected proteasome subunits and T cell CD4 Th2 (xCELL) infiltration. Each dot represents an individual tumor sample, and the blue line indicates the linear regression fit (Pearson’s r and *p*-value shown). Outlier-robust and density-aware visualizations are provided in Supplementary Figures S3 and S4.

Because dependencies of *PSMB5* and *PSMB6* showed significant correlations with expression of immune genes (*NCF4*, *SNX20*, *NLRC5*, *LGALS9* and *IRF1*, in Figure 4) or immune signaling pathways (HLA pathway, MHC and IFN response signatures in Figure 5), we further examined their correlation. The analysis of the correlation between the expression of proteasomal subunits and immune cell infiltration in patient tumor tissues, which were calculated based on the expression pattern of immune genes, showed stronger associations of CD4-positive Th2 cell infiltration with subunit expression (Figure 6A). Notably *PSMB5*, *PSMB6*, *PSMD11* and *PSMD14* showed significant associations with CD4-positive Th2 cell infiltration (Figure 6B–E).

To address the potential influence of outliers, we systematically re-evaluated the associations between proteasome subunit expression and CD4^+^ Th2 infiltration after excluding extreme values using an interquartile range (IQR)-based filtering approach. Notably, the observed positive correlations remained statistically significant and directionally consistent after outlier removal (Supplementary Figure S3 and Table S9), indicating that these associations are not driven by a small number of extreme samples.

To further improve the visualization of data distribution and avoid misinterpretation caused by sparse points, we incorporated density-aware visualization using hexbin-based scatter plots (Supplementary Figure S4). These plots clearly demonstrate that the positive associations between proteasome subunit expression and CD4^+^ Th2 infiltration are supported by the overall distribution of tumor samples rather than isolated data points.

In addition, to determine whether these associations simply reflect general co-expression among proteasome subunits, we compared the strength of correlations between proteasome subunits with those between each subunit and CD4^+^ Th2 infiltration (Supplementary Figure S5). Inter-subunit correlations were heterogeneous (Pearson’s r ranging from 0.07 to 0.64), and importantly, the correlations with CD4^+^ Th2 infiltration were comparable to or in several cases stronger than baseline inter-subunit correlations, indicating that these associations are not solely explained by general co-expression among proteasome subunits.

Collectively, these analyses demonstrate that the association between proteasome subunit expression and CD4^+^ Th2 infiltration is robust, not driven by outliers, and not merely a byproduct of general co-expression patterns.

In addition, among proteasomal subunits, *PSMB8*, *PSMB9,* and *PSMB10* showed more frequent association with the immune cell infiltration (Figure 6A).

**Figure 7:**
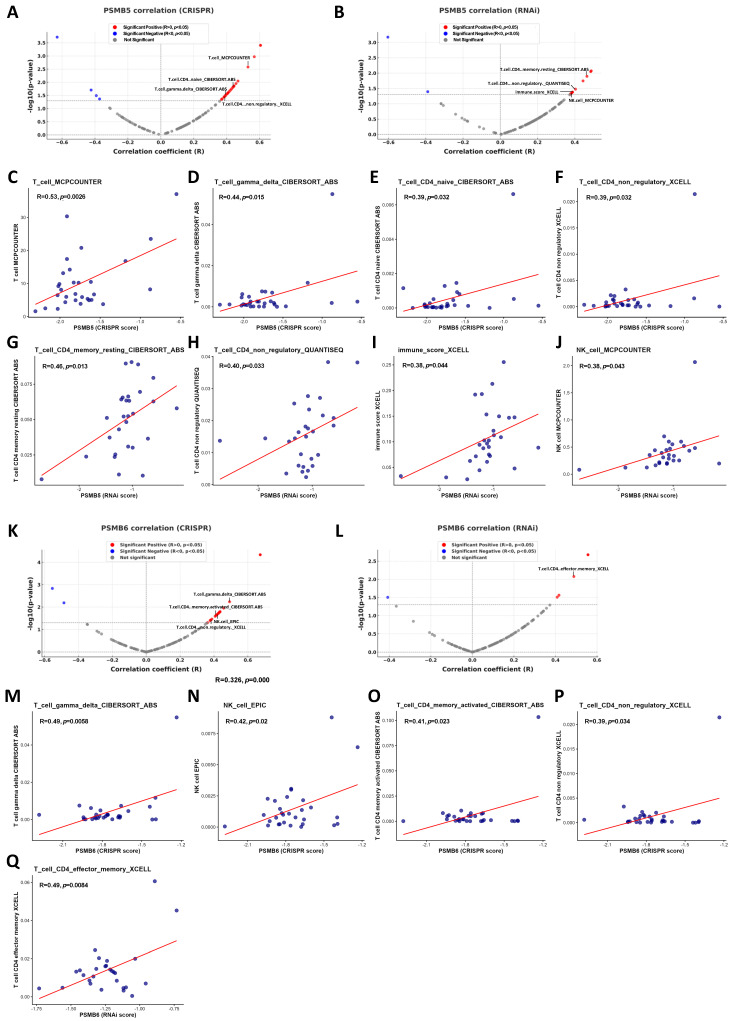
**Correlation between PSMB5 or PSMB6 dependency and immune cell infiltration across cancer types.** (**A**,**B**) Dependency scores of *PSMB5* from CRISPR (Chronos) (**A**) and RNAi (DEMETER2) (**B**) screens across cancer cell lines. Lower values represent stronger dependency. (**C**–**J**) Representative correlations between PSMB5 dependency scores and immune-cell infiltration estimates obtained from multiple algorithms (TIMER, CIBERSORT, xCell, QUANTISEQ, EPIC). (**K**,**L**) Dependency scores of *PSMB6* from CRISPR (Chronos) (**K**) and RNAi (DEMETER2) (**L**) datasets across cell lines, where negative scores indicate higher dependency. (**M**–**Q**) Representative correlations between PSMB6 dependency scores and immune-cell infiltration estimates obtained from multiple algorithms (TIMER, CIBERSORT, xCell, QUANTISEQ, EPIC). The *x*-axis shows dependency scores, and the *y*-axis shows infiltration levels. Red lines represent linear fits with Pearson’s r and *p* values.

Next, we examined whether the dependency of *PSMB5* and *PSMB6* in cancer cell lines is also correlated with immune cell infiltration in patient tumor tissues. These analyses revealed that the inverse relationship between immune activity and proteasome dependency observed at the pathway level was recapitulated in tumor tissue-based immune infiltration estimates. This analysis of both subunits revealed their frequent correlation with various kinds of immune cell infiltrations in patient tumor tissues (Figure 7A–Q). Interestingly CD4 T cell infiltration in patient tissues was frequently correlated with weaker dependency *PSMB5* (Figure 7E–H) or *PSMB6* (Figure 7O–Q). To further validate the generalizability of these findings, we next performed a meta-analysis across multiple independent hepatocellular carcinoma cohorts.

### 2.6. Meta-Analysis of Immune Infiltration-Associated Proteasome Subunits Across 24 Independent HCC Cohorts

Given the consistent associations between proteasome subunit expression and immune cell infiltration observed in the primary analyses (Figure 6 and Figure 7), we next evaluated the robustness of these relationships across 24 independent cohorts of hepatocellular carcinoma patients using a meta-analysis framework. To ensure that these associations were not cohort-specific or driven by dataset-specific biases, we systematically evaluated their reproducibility across multiple independent transcriptomic datasets using a meta-analysis framework.

**Figure 8:**
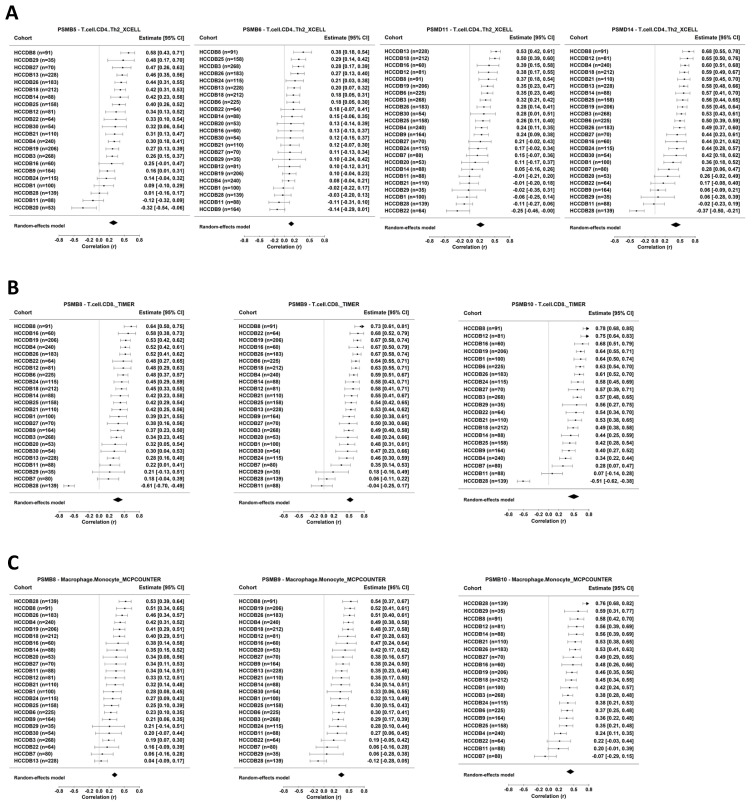
Meta-analysis of the associations between proteasome subunit expression and immune infiltration across 24 independent HCC cohorts. Forest plots summarizing cohort-wise correlation estimates and pooled effects for the associations between selected proteasome subunits and immune infiltration signatures across 24 independent HCC cohorts. Each plot shows the correlation coefficient with its corresponding confidence interval for each cohort, along with the pooled summary effect. (**A**) Meta-analysis for the CD4_Th2_XCELL signature. (**B**) Meta-analysis for the CD8_TIMER signature. (**C**) Meta-analysis for the Macrophage_monocyte_MCPCOUNTER signature. The pooled effect size is presented as the summary estimate across cohorts.

Across multiple HCC cohorts, the expression of proteasome subunits showed consistent positive correlations with immune infiltration signatures, including CD4 Th2 cells (XCELL), CD8 T cells (TIMER), and macrophage/monocyte populations (MCP-counter) (Figure 8A–C, Supplementary Figure S6). Importantly, the direction and magnitude of these associations were largely consistent across independent cohorts, supporting the robustness of the observed relationships beyond individual datasets. The pooled effect sizes indicated reproducible associations across cohorts, although the magnitude of correlation varied among subunits and immune cell types. Additional supporting analyses, including extended cohort-level results and alternative immune signatures, are provided in Supplementary Figure S6 and Tables S10–S12, further reinforcing the reproducibility of these findings. Several subunits, including *PSMB9* and *PSMD14*, exhibited relatively strong and consistent associations with immune infiltration, whereas others showed more moderate but still significant correlations (Supplementary Tables S10–S12). Despite inter-cohort heterogeneity, as reflected by I^2^ values, the overall directionality of associations was preserved across datasets. This heterogeneity likely reflects biological and technical variability across cohorts; however, the consistent directionality of associations suggests that the underlying relationship between proteasome subunit expression and immune infiltration is preserved. Collectively, these results demonstrate that the association between proteasome subunit expression and immune cell infiltration is not cohort-specific but represents a reproducible and robust feature across independent transcriptomic datasets. Taken together, these findings establish that the observed associations are reproducible across independent patient cohorts and are unlikely to be driven by dataset-specific artifacts, thereby providing strong support for the biological relevance of proteasome–immune interactions.

Although increased expression of immunoproteasome subunits such as *PSMB8*, *PSMB9*, and *PSMB10* in immune-rich tissues may reflect the presence of infiltrating immune cells, which are known to express these subunits at higher levels, the consistent correlation patterns observed across multiple subunits and independent datasets suggest that these associations reflect broader tumor immune context rather than a single gene- or cell-type-specific effect. However, we acknowledge that compositional effects from immune cell infiltration cannot be fully excluded.

### 2.7. Co-Essentiality Analysis of Proteasomal Subunits

**Figure 9:**
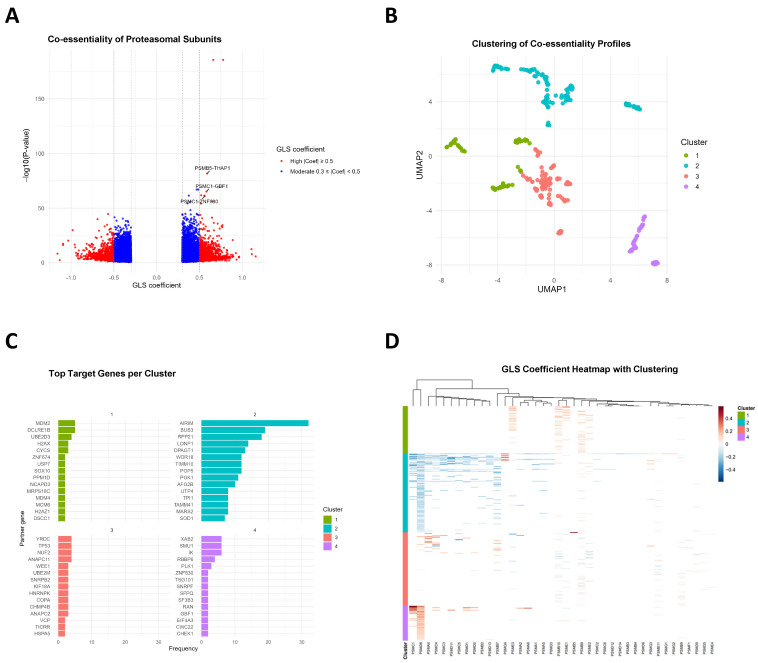
**Co-essentiality analysis of proteasomal subunits.** (**A**) Volcano plot displaying GLS coefficients for pairwise correlations among proteasomal subunits. Some significant pairs (|GLS coefficients| > 0.5, *p* < 0.05) are highlighted in red. (**B**) UMAP visualization of co-essentiality profiles. Proteasome subunits are distributed across multiple clusters rather than forming a single group, indicating heterogeneous and module-specific co-essentiality patterns. (**C**) Frequently co-correlated non-proteasomal genes within each cluster are listed. (**D**) Heatmap showing GLS coefficients across subunits with hierarchical clustering. Clusters are color-coded.

Since the proteasome consists of multiple core subunits as well as associated regulatory factors, we investigated the co-essentiality of each proteasome-related gene with non-proteasomal proteins. Using a generalized least squares (GLS) framework, we analyzed approximately 930,000 gene pairs across cancer cell lines and identified 721 significant co-essential pairs (FDR < 0.05, Benjamini–Hochberg correction; Supplementary Table S13, Figure 9A).

Notably, several high-confidence interactions exhibited strong positive co-essentiality, including pairs involving proteasome subunits and key regulatory proteins (Figure 9A). These associations suggest functional coupling between proteasome components and diverse cellular pathways.

Importantly, we further examined the distribution of proteasome subunits within the UMAP space (Figure 9B). Rather than forming a single compact cluster, proteasome subunits were distributed across multiple clusters, indicating heterogeneous co-essentiality patterns. This spatial separation suggests that individual proteasome components are associated with distinct functional modules.

Consistent with this observation, cluster-specific enrichment of co-essential partner genes revealed distinct functional signatures for each module (Figure 9C). For example, certain clusters were enriched for genes related to cell cycle regulation and oncogenic signaling, whereas others were associated with RNA processing or metabolic pathways, supporting functional specialization across clusters.

Furthermore, hierarchical clustering of GLS coefficients demonstrated that these modules exhibit distinct interaction patterns at the global level (Figure 9D). Strong co-essentiality signals were largely confined within specific clusters, while cross-cluster associations were comparatively weaker, reinforcing the modular organization of proteasome-associated dependencies.

Together, these results indicate that proteasome subunits are not functionally uniform but instead participate in context-dependent co-essentiality networks, as consistently supported across dimensional reduction (Figure 9B), partner enrichment (Figure 9C), and global interaction patterns (Figure 9D).

## 3. Discussion

In this study, we systematically characterized the dependency landscape of proteasome-related genes across cancers and prioritized *PSMB5* and *PSMB6* as reproducible, context-dependent biomarkers. By integrating CRISPR and RNAi datasets with transcriptomic, immune, and network-level analyses, our results highlight a reproducible association between proteasome dependency and tumor immune context, extend beyond descriptive proteasome biology, and provide a quantitative framework for biomarker-guided molecular targeting, rather than defining uniquely essential subunits. Notably, these findings position specific proteasome subunits as prioritized and context-associated therapeutic vulnerability candidates within the broader proteasome network. While proteasome dependency itself is a well-established feature of cancer cells, our study extends this concept by showing that specific subunits exhibit reproducible associations with immune-related molecular features across multiple independent datasets. Rather than representing uniquely essential proteasome components, *PSMB5* and *PSMB6* were prioritized based on their consistent and reproducible association with dependency and immune-related features across multiple datasets and analytical frameworks.

Importantly, proteasome subunits do not exert uniform functional effects across all contexts. Previous studies have shown that suppression of certain 19S regulatory subunits can, under specific conditions, confer a protective effect by reducing proteotoxic stress or modulating protein homeostasis pathways [16,17]. These findings highlight the complex and context-dependent roles of proteasome components. Consistent with this, our pan-cancer analysis showed heterogeneous dependency patterns among proteasome-related genes. *PSMB5* and *PSMB6* were prioritized because they showed reproducible dependency-associated patterns across both CRISPR and RNAi datasets, not because the present study experimentally establishes their uniqueness relative to all other proteasome components.

It should be noted that increased expression of immunoproteasome subunits (e.g., *PSMB8*, *PSMB9*, and *PSMB10*) in immune-infiltrated tumors may partially reflect the abundance of immune cells themselves, rather than tumor cell-intrinsic upregulation. Therefore, further studies using single-cell or spatial transcriptomic approaches will be required to distinguish tumor-intrinsic from immune cell-derived expression.

Specifically, perturbation of *PSMB5* resulted in consistent cytotoxic effects in many cancer types including acute lymphoblastic leukemia (ALL), acute myeloid leukemia (AML), uterine corpus endometrial carcinoma (UCEC), kidney renal clear cell carcinoma (KIRC), small cell lung cancer (SCLC), neuroblastoma (NBL), Ewing sarcoma (EWS), diffuse large B cell lymphoma (DLBC) and HCC. Previous studies have demonstrated that overexpression or mutation of *PSMB5* contributes to bortezomib resistance in hematologic malignancies such as ALL, AML, and DLBC, as well as in SCLC [19]. In solid tumors such as UCEC and KIRC, *PSMB5* is frequently overexpressed and associated with poor patient prognosis [15]. Additionally, in neuroblastoma, *PSMB5* has been implicated in protecting cells from oxidative stress, suggesting a role in cellular stress responses [20]. These findings support further investigation of PSMB5 as a dependency-associated biomarker in these cancer contexts.

Both CRISPR and RNAi-mediated perturbations of *PSMB6* demonstrated consistent cytotoxic effects across several cancer types, including KIRC, skin cutaneous melanoma (SKCM), ALL, colon adenocarcinoma (COAD), lower-grade glioma (LGG), glioblastoma multiforme (GBM), and HCC. Notably, *PSMB6* is overexpressed in LGG, GBM, and SKCM, and its elevated expression has been associated with poor patient prognosis in these cancers [21,22]. In contrast, although *PSMB6* expression is downregulated in KIRC, its expression levels still correlate with patient survival [23]. Furthermore, hypoxic conditions commonly observed in LGG and GBM have been linked to PSMB6 expression, suggesting that *PSMB6* may contribute to cellular adaptation under hypoxia [21]. In SKCM, *PSMB6* overexpression is associated with the downregulation of immune-related genes, possibly contributing to immune evasion in this cancer type [22]. These findings support further investigation of PSMB6 as a context-associated biomarker, particularly in relation to stress adaptation and immune-related transcriptional states.

Notably, these dependency patterns were experimentally validated, as siRNA-mediated knockdown of *PSMB5* and *PSMB6* led to reduced proliferative capacity across multiple hepatocellular carcinoma cell lines (Figure 3). This functional validation supports the biological relevance of the dependency signals observed in large-scale perturbation datasets.

Although functional validation was primarily performed in hepatocellular carcinoma models, the cross-cancer relevance of *PSMB5* and *PSMB6* is strongly supported by large-scale dependency datasets encompassing diverse cancer types. The consistent dependency patterns observed across CRISPR and RNAi platforms, together with reproducible associations with immune-related pathways across multiple cancer lineages, suggest that these subunits represent broadly conserved therapeutic vulnerabilities rather than HCC-specific phenomena. In this context, HCC was utilized as a representative model that enables integration of functional validation with extensive patient-level immune and transcriptomic data. Nevertheless, additional experimental validation in other tumor types will be valuable to further confirm the universality of these findings.

The perturbation effects of *PSMB5* and *PSMB6* are associated with the immune activity or the expression level of immune-related genes in various types of cancer cells. Specifically, the expression levels of *NCF4* and *LGALS9* show a negative correlation with the cytotoxic effects resulting from *PSMB5* perturbation (Figure 4D and 4I; R = 0.445, *p* = 0.000; R = 0.396, *p* = 0.000, respectively). Similarly, enrichment of CD48 and HLA also exhibits a negative relationship with *PSMB5* perturbation effects (Figure 5D and 5F; R = 0.547, *p* = 0.000; R = 0.479, *p* = 0.000, respectively). Moreover, *PSMB6* perturbation effects are negatively correlated with the enrichment of MHC and interferon response pathways (Figure 5G and 5H; R = 0.326, *p* = 0.000; R = 0.360, *p* = 0.000, respectively). These findings suggest that proteasome dependency is functionally linked to the immune regulatory state of tumor cells, particularly in contexts with reduced immune signaling activity. This relationship may indicate that cancer cells with diminished immune activity become more reliant on proteasome function, thereby increasing their vulnerability to proteasome perturbation. However, these associations should be interpreted as correlative and hypothesis-generating rather than causal evidence of immune-state-dependent therapeutic susceptibility. Additionally, the expression levels of *NLRC5* and *IRF1*, key regulators of MHC class I antigen presentation, are inversely related to the cytotoxic effects of *PSMB6* perturbation (Figure 4F and 4J; R = 0.351, *p* = 0.000; R = 0.389, *p* = 0.000, respectively). These findings suggest that cancer cells with low immune reactivity are more susceptible to *PSMB5* or *PSMB6* perturbation. Importantly, these associations with immune cell infiltration were consistently observed across 24 independent HCC cohorts, as confirmed by meta-analysis (Figure 8 and Figure S6, Supplementary Tables S10–S12), indicating the relationship between proteasome-related features and immune infiltration is reproducible across datasets.

Further analysis in patient cohorts showed that expression levels of many proteasomal subunits are more strongly correlated with CD4-positive Th2 cell infiltration. CD4-positive Th2 cells, which mainly secrete cytokines such as IL-4, IL-5, and IL-13, play an important role in regulating humoral immune responses and allergic reactions [24]. Interestingly, Th2 cells mainly induce an immunosuppressive state through the secretion of anti-inflammatory cytokines in the tumor microenvironment, which may inhibit the function of other anti-tumor immune cells. On the other hand, some studies suggest that Th2 cells may promote the terminal differentiation of tumor cells, thereby inhibiting cancer progression, indicating that their role is context-dependent. Th2 cells induce the polarization of M2-type tumor-associated macrophages (TAMs) in the tumor microenvironment by secreting cytokines such as IL-4 and IL-13 [25]. The activity of Th2 cells can prevent the infiltration and activation of cytotoxic T lymphocytes (CTLs) through cytokine secretion. For example, some studies have shown that high levels of Th2 cells are negatively correlated with CTL infiltration and function, which is associated with a decrease in the therapeutic effect of immune checkpoint blockade (ICB) [26]. On the other hand, cases have been reported in which Th2 cells contribute to cancer suppression under certain conditions. Some studies have revealed that Th2 cells can induce terminal differentiation of tumor cells, thereby inhibiting cancer growth and metastasis. For example, in a breast cancer model, Th2 cells were reported to induce terminal differentiation of tumor cells, thereby inhibiting tumor growth [27]. In limited environments, Th2 cells can induce inflammatory responses by converting to Th1 cells or by readjusting the Th1/Th2 balance, ultimately promoting anti-tumor immune responses [28]. Notably Th2 cell infiltration is closely associated with the overall survival rate (OS) and recurrence-free survival rate (PFS) of patients [29]. Some studies have shown that high infiltration of Th2 cells is associated with a poor response to immune checkpoint blockade (ICB) treatment, whereas high CTL infiltration is linked with better prognosis [30]. Given the critical role of the proteasome in intracellular antigen processing and presentation via MHC class I molecules [31,32], further mechanistic studies are warranted to explore this sensitivity.

These observations provide a hypothesis-generating framework linking proteasome dependency-associated patterns to tumor immune regulation. Given the central role of the proteasome in generating antigenic peptides for MHC class I presentation [33], alterations in PSMB5 and PSMB6 activity may directly affect the repertoire and efficiency of antigen presentation. The observed inverse association between *PSMB5*/*PSMB6* dependency and key regulators of antigen presentation, such as NLRC5 and IRF1, further supports the hypothesis that these subunits may modulate immune visibility of tumor cells. In addition, the consistent association with interferon response pathways suggests a potential role in shaping inflammatory signaling and immune activation states. Collectively, these findings indicate that *PSMB5* and *PSMB6* may influence tumor–immune interactions through coordinated regulation of antigen processing and immune signaling pathways, although direct experimental validation will be required to establish causality.

Furthermore, the observed association between proteasome dependency and immune signaling provides a rationale for considering combination strategies with immunotherapy, but this concept requires careful contextual interpretation. In the present study, stronger *PSMB5* and *PSMB6* dependency was associated with lower expression of immune regulators such as NLRC5 and IRF1, as well as lower enrichment of HLA/MHC and interferon-response pathways, suggesting that proteasome-targetable states may be enriched in relatively immune-low tumors. In this context, the most relevant immunotherapeutic partners are likely to be immune checkpoint inhibitors (ICIs), particularly PD-1/PD-L1 blockade and, in selected settings, CTLA-4-containing combinations.

This issue is especially relevant in hepatocellular carcinoma (HCC), where immunotherapy has become a major component of systemic treatment. Current first-line regimens for advanced HCC include atezolizumab plus bevacizumab and durvalumab plus tremelimumab, indicating that checkpoint-based therapy already represents the clinical backbone for this disease [34]. Therefore, if proteasome-targeted approaches are further developed for HCC, the most plausible translational strategy would be to evaluate them in combination with these checkpoint-based regimens, particularly in tumors with weak baseline immune activation or impaired antigen-presentation programs.

Mechanistically, such combinations may be relevant for at least two reasons. First, proteasome-targeted stress can increase tumor cell vulnerability and may enhance immunogenicity under some conditions, thereby potentially improving responsiveness to checkpoint blockade. Second, our data suggest that proteasome dependency is linked to an immune-low transcriptional state, raising the possibility that proteasome-targeted therapy could preferentially debulk tumor cells that are less responsive to immunotherapy alone. However, this concept should be interpreted with caution because the proteasome is also fundamentally involved in MHC class I antigen processing and presentation. Thus, depending on the degree and timing of inhibition, proteasome-directed therapy could either enhance antitumor immune priming or, conversely, impair antigen presentation and immune-cell function. For this reason, future studies should determine whether sequential, intermittent, or subunit-selective proteasome targeting is more compatible with immunotherapy than continuous broad proteasome inhibition.

Taken together, our findings do not establish a proteasome inhibitor–immunotherapy combination as a validated treatment strategy, but they do support it as a biologically grounded hypothesis, especially for immune-cold HCC. Future work should directly test combinations of *PSMB5*/*PSMB6*-targeted approaches with PD-1/PD-L1-based therapy, with or without anti-VEGF or CTLA-4-based components, in immunocompetent preclinical HCC models.

Based on GLS clustering, we identified four distinct clusters, suggesting that specific proteasomal subunits may play critical roles in the degradation of particular target proteins. Our analysis revealed both known and novel associations between proteasomal subunits and cancer-related genes. In Clusters 1, 3, and 4, notable oncogenes and tumor suppressors such as *MDM2*, *TP53*, and *KRAS*, which are substrates of the ubiquitin–proteasome system, were observed. Notably, the *PSME3*–*MDM2* pair exhibited a high GLS coefficient, supporting previous studies showing that *PSME3* (also known as PA28γ) enhances MDM2-mediated degradation of p53 by facilitating their interaction [35,36]. This interaction establishes a regulatory feedback loop that controls p53 stability and thereby influences cell cycle progression and apoptosis. Additionally, our results indicate that KRAS may interact with proteasomal subunits PSMD2 and PSMC3. PSMD2, a non-ATPase subunit of the 19S regulatory complex, has been implicated in the degradation of oncogenic regulators and is associated with cancer progression and poor clinical outcomes [37]. PSMC3, an ATPase subunit, is essential for 26S proteasome function and may also regulate oncogenic signaling cascades. These novel associations warrant further mechanistic investigation to elucidate the roles of proteasomal subunits in tumorigenesis and therapeutic response.

These findings have important implications for therapeutic strategies. Proteasome inhibitors, including bortezomib, carfilzomib, and ixazomib, have become pivotal in the treatment of hematological malignancies, particularly multiple myeloma. However, their clinical application faces several limitations. One of the major challenges is the development of resistance in cancer cells, which can be attributed to various mechanisms such as mutations in proteasome subunits (e.g., *PSMB5*), altered expression of drug targets, and activation of compensatory survival pathways (McConkey & Zhu, 2008 [14]). Although these inhibitors are effective in hematologic cancers, their therapeutic efficacy in solid tumors remains limited. The unique microenvironment of solid tumors—characterized by hypoxia and the presence of stromal cells—can hinder drug penetration and activity [38]. Additionally, proteasome inhibitors often lack specificity, resulting in off-target effects that damage normal cells in addition to cancer cells. This non-selective mechanism can lead to a range of adverse effects, including peripheral neuropathy, gastrointestinal toxicity, and cardiovascular complications. For example, bortezomib is well-documented to cause neurotoxicity, which may necessitate treatment discontinuation in some patients [39,40]. To address these limitations, therapeutic strategies targeting individual proteasomal subunits could provide more selective and tolerable alternatives. Based on subunit expression profiles and functional efficacy in different cancer types, rational combinations of subunit-specific perturbations may help to overcome resistance and reduce toxicity associated with conventional proteasome inhibitors. Furthermore, the observed association between proteasome dependency and immune signaling raises the possibility that proteasome-targeted therapies may be most effective in tumors with low immune activity, or could be rationally combined with immunotherapies to overcome immune resistance. Emerging strategies, including subunit-specific small molecules and RNA-based therapeutics, further support the feasibility of this approach.

Multi-kinase inhibitors such as sorafenib and regorafenib are standard treatments for advanced HCC; however, their clinical efficacy is often limited by intrinsic and acquired resistance [34]. Given the central role of the proteasome in maintaining proteostasis and regulating stress responses, targeting *PSMB5* or *PSMB6* may enhance the therapeutic effects of these agents. For example, multi-kinase inhibitors can induce cellular stress, including oxidative and endoplasmic reticulum stress, and concomitant inhibition of proteasome function may exacerbate proteotoxic stress, thereby promoting cancer cell death. In addition, proteasome activity is involved in the regulation of survival signaling pathways and degradation of pro-apoptotic factors, suggesting that inhibition of specific subunits may disrupt adaptive resistance mechanisms to kinase inhibitors. Furthermore, the observed association between proteasome dependency and immune signaling pathways raises the possibility that proteasome-targeted strategies could modulate the tumor immune microenvironment, potentially enhancing the efficacy of combination therapies involving targeted agents and immunotherapy. Although these hypotheses require further experimental validation, our findings support the potential utility of *PSMB5* and *PSMB6* as biomarkers for rational combination strategies in HCC.

The application of small interfering RNA (siRNA) in cancer therapeutics has garnered significant attention, particularly with advancements in delivery systems such as lipid nanoparticles (LNPs), cationic nanoparticles, polymer-based carriers, conjugate delivery systems, exosome-based platforms, and RNA-based nanostructures. These delivery vehicles have been extensively studied to enhance the stability and targeting efficiency of siRNA molecules in vivo. Notably, Atu027, an siRNA formulation targeting protein kinase N3 (*PKN3*), utilized LNPs for delivery and completed a Phase I clinical trial in patients with advanced solid tumors. The study demonstrated the feasibility and safety of systemic siRNA administration using LNPs [41]. Similarly, TKM-PLK1, an siRNA therapeutic targeting polo-like kinase 1 (*PLK1*), employed stable nucleic acid-lipid particles (*SNALPs*) and also completed a Phase I clinical trial, highlighting the potential of SNALP-based delivery systems in oncology [42]. Building upon these findings, the present study suggests that siRNA targeting specific proteasomal subunits, such as *PSMB5*, could be a viable approach in cancer therapy. Previous research has reported the successful application of *PSMB5*-targeted siRNA in multiple myeloma and lung cancer cells, resulting in significant anti-tumor effects [43,44,45]. Combining proteasomal subunit-specific siRNA with conventional proteasome inhibitors may offer synergistic benefits, potentially reducing adverse effects and overcoming resistance mechanisms associated with proteasome inhibitor therapies.

This study has several limitations. First, dependency analyses were based on in vitro cell line models, which may not fully capture tumor heterogeneity and microenvironmental influences in vivo. These associations should therefore be interpreted with caution, as they may be influenced by complex interactions between tumor-intrinsic and microenvironmental factors. Therefore, future studies using animal models or more advanced systems such as patient-derived xenografts or organoid-based co-culture models will be important to further validate the functional and immunological roles of *PSMB5* and *PSMB6* in a physiological context. Second, although immune associations were robustly validated across multiple cohorts, causal relationships between proteasome dependency and immune modulation remain to be elucidated. Third, functional validation was primarily performed in hepatocellular carcinoma models, and further validation across additional cancer types will be necessary to establish broader clinical applicability. Fourth, the efficiency of siRNA-mediated knockdown was not quantitatively validated across all experimental conditions, which may affect the interpretation of the observed functional effects and should be addressed in future studies. Importantly, because dependency scores reflect functional perturbation effects rather than absolute gene inactivation, these results should be interpreted in the context of underlying genomic features such as copy number variation and incomplete perturbation efficiency. Fifth, methodological heterogeneity exists between our computational and experimental analyses: the high-throughput dependency datasets (DepMap) assess cellular viability following perturbation, whereas our cell-based functional experiments (Figure 3) measure growth rate over time. While both endpoints are broadly consistent with reduced fitness following *PSMB5* or *PSMB6* knockdown, they are not formally equivalent, and this distinction should be considered when interpreting the experimental data as validation of the computational findings. Sixth, we acknowledge the fundamental biological constraint raised by the proteasome’s nature as a multi-subunit complex: in principle, loss of any single essential subunit can disrupt assembly and impair overall proteolytic function. The DepMap screens measure genome-wide dependency across thousands of cell lines, and the extent to which individual subunits were successfully perturbed varies across screens and cell lines. Because we cannot fully verify perturbation efficiency for all subunits in these large-scale datasets, the observed differential dependency of PSMB5 and PSMB6 should be interpreted with caution. Rather than asserting that these two subunits are uniquely essential in an absolute mechanistic sense, our conclusions are limited to the observation that they show the most consistent and reproducible dependency signal across both CRISPR and RNAi platforms, across the widest range of cancer types, and with the strongest association with immune-related molecular features. Direct experimental comparison of *PSMB5* and *PSMB6* knockdown against a panel of other proteasome subunits—using matched assays with verified perturbation efficiencies and including both viability and growth-rate endpoints—will be required in future studies to formally establish whether PSMB5 and PSMB6 are more critical than other individual subunits.

In conclusion, this study provides a data-driven framework for understanding proteasome dependency in relation to tumor immune context, highlighting *PSMB5* and *PSMB6* as consistently associated subunits across multiple datasets. By integrating cross-platform dependency data with immune and clinical context, our work provides a conceptual and translational framework for precision targeting of proteasome subunits, with potential implications for improving therapeutic selectivity and overcoming resistance in cancer treatment.

## 4. Materials & Methods

### 4.1. Dependency Analysis

CRISPR-Cas9 and RNA interference (RNAi) perturbation screening datasets were obtained from the DepMap portal (https://depmap.org/portal/, accessed on 24 June 2025). CRISPR dependency scores were derived from the Chronos model (1178 cell lines), while RNAi scores were taken from the DEMETER2-integrated dataset, which includes data from the Achilles, DRIVE, and Marcotte screens (707 cell lines). Cancer type annotations were assigned based on the sample metadata file provided by DepMap. For each proteasomal subunit, dependency scores were averaged across cell lines grouped by cancer type. Matched molecular features for each cell line—including gene expression (log2[TPM + 1]), copy number alterations (log2 relative to ploidy + 1), and mutation profiles—were also retrieved from the DepMap portal. Subunits or cancer types with missing dependency scores were excluded prior to downstream aggregation analyses. This preprocessing step ensured that zero counts in summary analyses reflect true absence of dependency rather than missing data.

### 4.2. Differential Expression Analysis

The expression data used in this study were retrieved from the TCGA Pan-Cancer Atlas project (https://gdc.cancer.gov/about-data/publications/pancanatlas/, accessed on 24 June 2025) and the UCSC Xena portal (https://xena.ucsc.edu/, accessed on 24 June 2025). Additional genetic data were downloaded directly from the cBioPortal (https://www.cbioportal.org/, accessed on 24 June 2025). Log2 fold change values were computed by comparing tumor sample expression levels with the average expression of matched normal tissues, based on the TCGA and GTEx datasets. The results were visualized using a clustered heatmap with cancer-type annotations. Pairwise Pearson correlation coefficients (r) and *p*-values were calculated across tumor samples.

### 4.3. Cox Regression Analysis

For all proteasomal subunits which are described before [15], we conducted univariate Cox regression analysis based on overall survival (OS) or progression-free interval (PFI) using Python (3.13).

### 4.4. Estimation of Immune Cell Infiltration

Immune cell infiltration estimates were obtained from the TIMER3.0 portal (http://compbio.cn/timer3/, accessed on 24 June 2025), which provides deconvolution-based immune infiltration scores derived from TCGA pan-cancer transcriptomic datasets. Multiple algorithms—including TIMER, CIBERSORT, quanTIseq, xCell, MCP-counter, and EPIC—were used to ensure robustness across estimation methods. Associations between proteasome subunit expression and immune cell infiltration scores were assessed using Pearson correlation analysis [46,47].

### 4.5. Coessentiality Analysis

Co-essentiality relationships were assessed using generalized least squares (GLS) regression to evaluate correlations between the perturbation profile of each proteasomal subunit and all other genes across cell lines. GLS was implemented to account for non-independence among cell lines and to improve estimation accuracy compared to simple correlation methods. Following computation of GLS coefficients, dimensionality reduction and clustering were performed using Uniform Manifold Approximation and Projection (UMAP) to identify modular gene clusters. Clustering parameters were selected based on default settings unless otherwise indicated.

### 4.6. Data Availability and Ethical Considerations

All datasets analyzed in this study were publicly available and accessed from established repositories (DepMap, TCGA, GDC, UCSC Xena, cBioPortal, and TIMER3.0). No new human or animal data were generated; therefore, institutional ethical approval was not required.

### 4.7. Cell Culture and siRNA Transfection

Human hepatocellular carcinoma cell lines (Huh7, JHH7, HLF, SK-HEP-1, HLE, PLC/PRF/5, and SNU-449) were cultured under standard conditions. Cells were seeded into 96-well plates and transfected with small interfering RNAs (siRNAs) targeting PSMB5 or PSMB6, or with a non-targeting control siRNA (siSCR), using DharmaFECT 1 (Horizon Discovery, Cambridge, UK) at a final concentration of 100 nM. siRNA sequences have been summarized in Supplementary Table S14. For siRNA transfection, scrambled (SCR) and PSMB5, PSMB6 siRNAs were purchased from Dharmacon (Lafayette, CO, USA) and Bioneer Corporation (Daejeon, Republic of Korea), respectively.

### 4.8. Real-Time PCR

Cells were seeded onto 6-well plates; 24 h later, cells were transfected with siRNA; 48 h later, cells were harvested and RNA was extracted. With this extracted RNA, cDNA synthesis was performed. Furthermore, real-time PCR was performed with conditions like Hold stage, PCR stage and Melt curve stage. Primers used in this study are summarized in Supplementary Table S15.

### 4.9. Cell Proliferation Assayh

Cell proliferation and viability were assessed using the Ez-Cytox cell viability assay (Daeil Lab Service, Seoul, Republic of Korea), a colorimetric assay based on the reduction of a water-soluble tetrazolium salt (WST) by metabolically active cells. In viable cells, mitochondrial dehydrogenases convert the tetrazolium salt into a water-soluble formazan dye, the amount of which is proportional to the number of living cells.

Hepatocellular carcinoma cell lines (Huh7, JHH7, HLF, SK-HEP-1, HLE, PLC/PRF/5, and SNU-449) were seeded into 96-well plates following siRNA transfection (PSMB5, PSMB6, or non-targeting control siSCR; final concentration 100 nM). Cells were cultured under standard conditions and monitored over a time course of 1–5 days.

At each indicated time point, Ez-Cytox reagent was added to each well according to the manufacturer’s instructions (typically 10 μL per 100 μL culture medium) and incubated for 1–2 h at 37 °C. Absorbance was measured at 450 nm using a microplate reader. Background absorbance from medium-only wells was subtracted from all measurements.

Optical density (OD) values were used as a proxy for viable cell numbers and were normalized to Day 1 or control (siSCR) conditions where indicated. All experiments were performed in at least triplicate wells and repeated independently. Data are presented as mean ± standard deviation (SD).

It should be noted that the Ez-Cytox assay measures metabolic activity rather than direct cell counts; therefore, the results reflect relative cell viability/proliferation rather than absolute cell numbers.

### 4.10. Meta-Analysis of Associations Between Proteasome Subunit Expression and Immune Cell Infiltration

Associations between proteasome subunit expression and immune cell infiltration were evaluated across 24 independent HCC cohorts (GSE22058, GSE25097, GSE36376, GSE14520, GSE10143, GSE9843, GSE19977, GSE46444, GSE54236, GSE63898, GSE43619, GSE64041, ICGC-LIRI-JP, GSE124751, GSE164760, GSE134568, GSE87630, GSE76427, OEP000321, GSE112790, GSE121248, GSE109211, GSE89377, GSE148355). For each cohort, Pearson correlation coefficients (r) were calculated between gene expression and immune cell infiltration scores estimated by XCELL (CD4 Th2 cells), TIMER (CD8 T cells), and MCP-counter (macrophage/monocyte). Correlation coefficients were integrated using meta-analysis to obtain pooled effect sizes and 95% confidence intervals. Heterogeneity was assessed using the I^2^ statistic. Statistical significance was evaluated using meta-analysis-derived *p*-values with false discovery rate (FDR) correction. Forest plots and summary tables were generated to present the results.

All assay conditions and reporting were designed to align with the general principles of Minimum Information About a Cellular Assay (MIACA) guidelines.

## Data Availability

Derived data supporting the findings of this study and materials are available from the corresponding author S.-O.O. on request.

## Supplementary Materials

The following supporting information can be downloaded at: https://www.mdpi.com/article/10.3390/molecules31111954/s1, Figure S1. Expression patterns of proteasome subunits across TCGA pan-cancer and 24 independent HCC cohorts. Figure S2. Validation of siRNA-mediated knockdown of PSMB5 and PSMB6 in HCC cell lines. Figure S3. Robustness analysis of proteasome subunit expression–T cell CD4 Th2 infiltration correlations after outlier removal. Figure S4. Joint distribution of proteasome subunit expression and CD4^+^ Th2 T cell infiltration. Figure S5. Comparison between inter-subunit expression correlations and immune signature correlations. Figure S6. Cohort-specific heatmaps of correlations between proteasome subunit expression and immune cell infiltration signatures in LIHC. Table S1. Cancer type classification for DepMap model cell lines. Table S2. Status of common or selective subunits based on the CRISPR effect. Table S3. Status of common or selective subunits based on the RNAi effect. Table S4. CRISPR–Immune Feature Correlations. Table S5. RNAi–Immune Feature Correlations. Table S6. Gene composition of ssGSEA pathways. Table S7. ssGSEA_CRISPR Correlations. Table S8. ssGSEA_RNAi Correlations. Table S9. Robustness analysis of correlations after outlier removal. Table S10. Meta-analysis summary of associations between proteasome subunit expression and CD4 Th2 cell infiltration (XCELL). Table S11. Meta-analysis summary of associations between proteasome subunit expression and CD8 cell infiltration (TIMER). Table S12. Meta-analysis summary of associations between proteasome subunit expression and macrophage monocyte cell infiltration (MCPCOUNTER). Table S13. List of gene pairs per cluster. Table S14. List of siRNA sequences in this study. Table S15. List of quantitative PCR primers in this study.

## Abbreviations

ACC	Adrenocortical Carcinoma
ALCL	Anaplastic Large-Cell Lymphoma
ALL	Acute Lymphoblastic Leukemia
ATLL	Adult T Cell Leukemia/Lymphoma
ATRT	Atypical Teratoid/Rhabdoid Tumor
BL	Burkitt Lymphoma
BLCA	Bladder Urothelial Carcinoma
BRCA	Breast Invasive Carcinoma
CESC	Cervical Squamous Cell Carcinoma and Endocervical Adenocarcinoma
CHOL	Cholangiocarcinoma
CHS	Chondrosarcoma
COAD	Colon Adenocarcinoma
DLBC	Diffuse Large B Cell Lymphoma
ESCA	Esophageal Carcinoma
EWS	Ewing Sarcoma
GBM	Glioblastoma
HL	Hodgkin Lymphoma
HNSC	Head and Neck Squamous Cell Carcinoma
KIRC	Kidney Renal Clear Cell Carcinoma
KIRP	Papillary Renal Cell Carcinoma
LAML	Acute Myeloid Leukemia
LCLC	Large Cell Lung Carcinoma
LCML	Chronic Myeloid Leukemia
LGG	Low Grade Glioma
LIHC	Hepatocellular Carcinoma
LSCLC	Non-Small Cell Lung Carcinoma
LUAD	Lung Adenocarcinoma
LUSC	Lung Squamous Cell Carcinoma
MB	Medulloblastoma
MCL	Mantle Cell Lymphoma
MESO	Mesothelioma
MM	Multiple Myeloma
MOC	Mucinous Ovarian Carcinoma
MPNST	Malignant Peripheral Nerve Sheath Tumor
MRT	Malignant Rhabdoid Tumor
NBL	Neuroblastoma
OCCC	Clear Cell Ovarian Carcinoma
OEC	Endometrioid Ovarian Carcinoma
OS	Osteosarcoma
OV	Ovarian Serous Cystadenocarcinoma
PAAD	Pancreatic Adenocarcinoma
PRAD	Prostate Adenocarcinoma
READ	Rectal Adenocarcinoma
RMS	Rhabdomyosarcoma
SARC	Sarcoma
SCLC	Small Cell Lung Cancer
SKCM	Skin Cutaneous Melanoma
SMARCA4-UT	SMARCA4-deficient undifferentiated tumor
STAD	Stomach Adenocarcinoma
TGCT	Testicular Germ Cell Tumor
THCA	Thyroid Carcinoma
UCEC	Uterine Corpus Endometrial Carcinoma
UCS	Uterine Carcinosarcoma
UVM	Uveal Melanoma
WT	Wilms Tumor
