# Supplementary material for: Integrative Pan-Cancer Mapping of Proteasome Dependency Prioritizes PSMB5 and PSMB6 as Context-Dependent Vulnerability Biomarkers Linked to Immune Context
Source: Molecules. 2026 Jun 4;31(11):1954. doi: 10.3390/molecules31111954 (PMC13258478; doi:10.3390/molecules31111954)
Supplement: Supplementary file 1 [file molecules-31-01954-s001.zip › molecules-4265658-supplementary.pdf]

Supplementary Fig. S1

A

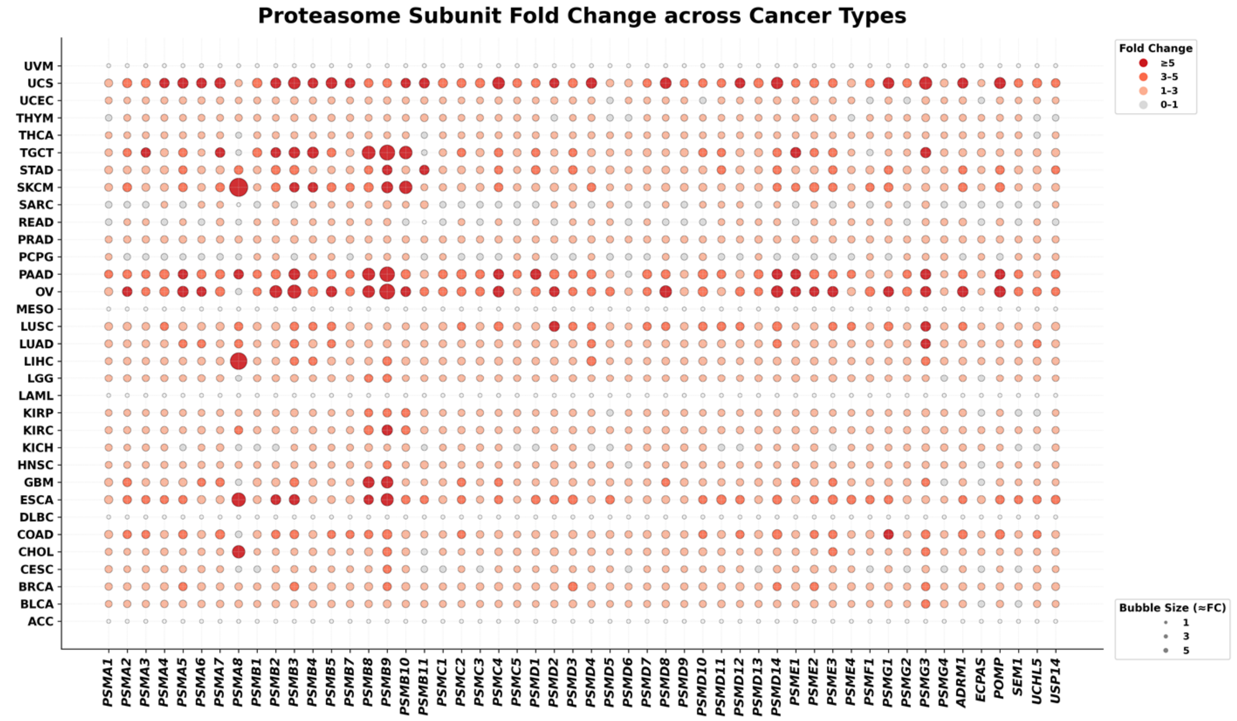

B

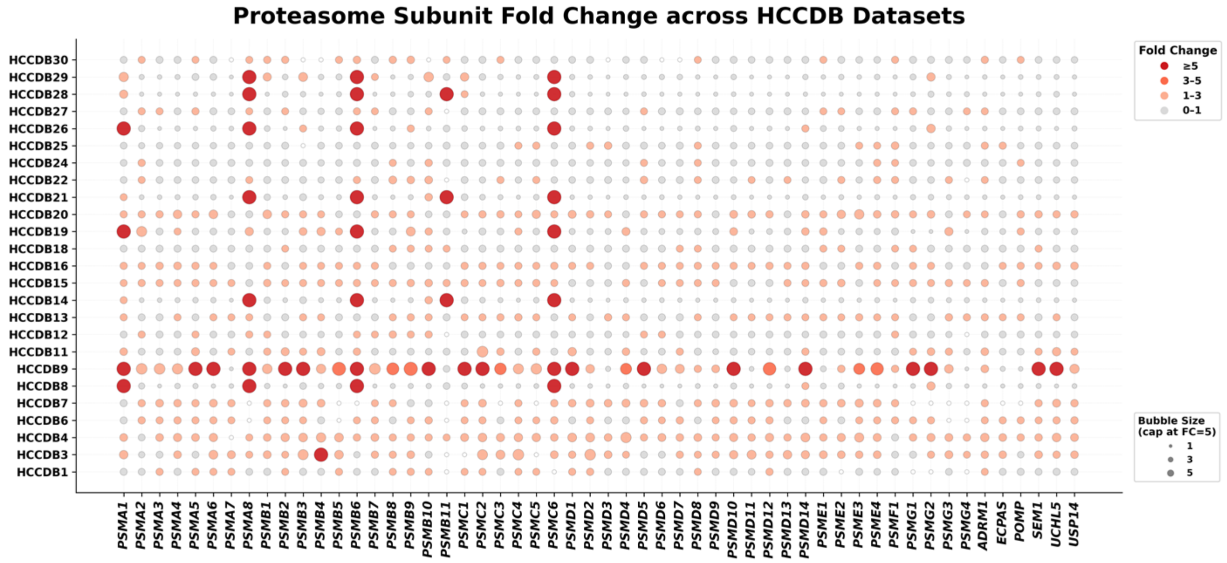

Supplementary Fig. S1. Expression patterns of proteasome subunits across TCGA pan-cancer and 24 independent HCC cohorts. Bubble plots illustrating the expression fold change of proteasome subunits across cancer types and independent cohorts. (A) Expression fold change of proteasome subunits across TCGA pan-cancer datasets. (B) Expression fold change of proteasome subunits across 24 independent HCC cohorts. Each circle represents the mean fold change of a subunit within a given cancer type or cohort. The size of the circle corresponds to the magnitude of the fold change, and the color intensity reflects the degree of expression change, with darker red indicating higher expression levels relative to normal tissues.

Supplementary Fig. S2

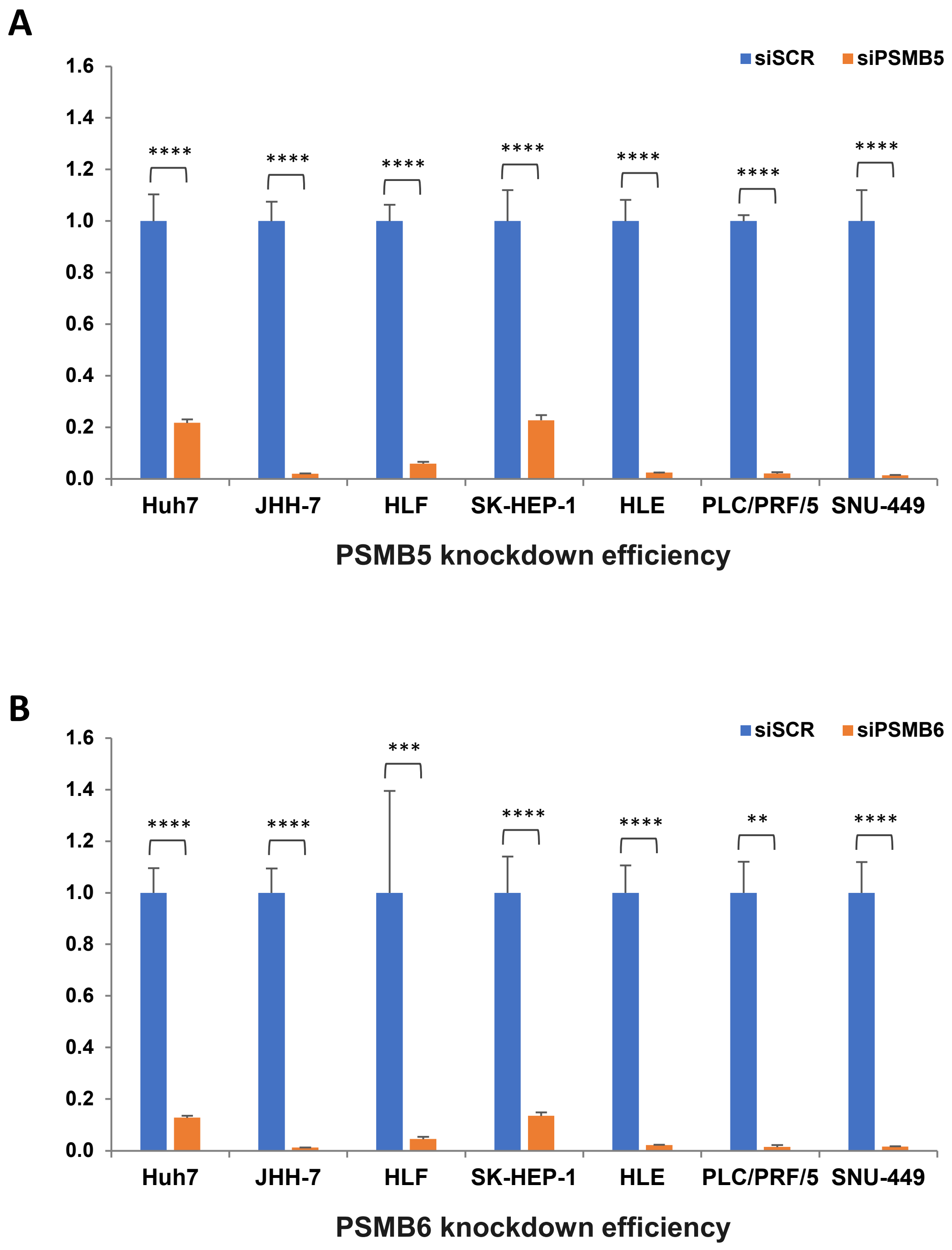

Supplementary Fig. S2. Validation of siRNA-mediated knockdown of PSMB5 and PSMB6 in HCC cell lines. (A) Relative expression levels of PSMB5 after siPSMB5 transfection in JHH7, HLE, SK-HEP-1, SNU449, HLF, and PLC cells. (B) Relative expression levels of PSMB6 after siPSMB6 transfection in the indicated HCC cell lines. Expression levels were normalized to the siSCR control, which was set to 1.0. Data are presented as mean ± SD.

Supplementary Fig. S3

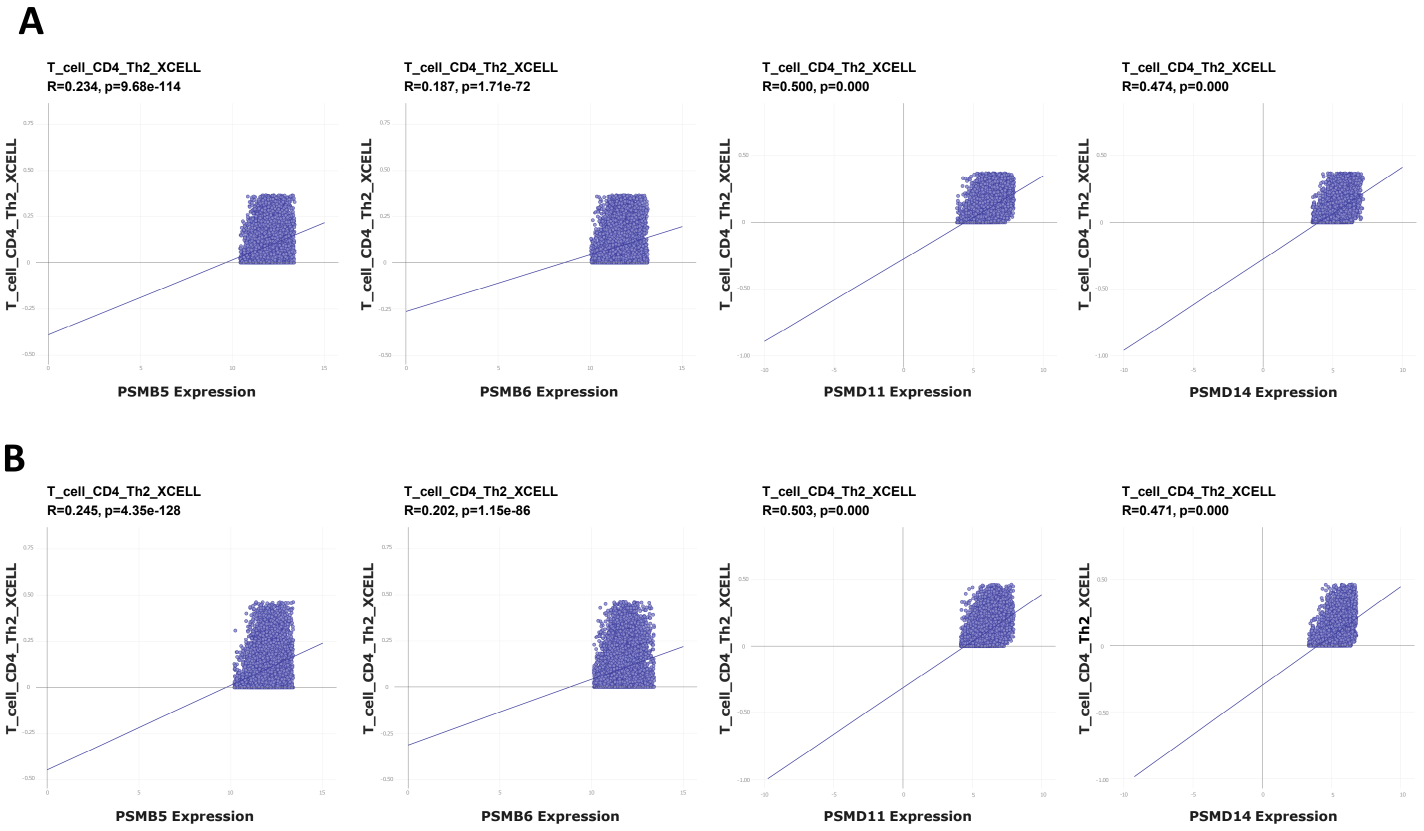

Supplementary Fig. S3. Robustness analysis of proteasome subunit expression–T cell CD4 Th2 infiltration correlations after outlier removal. Scatter plots show the correlations between proteasome subunit expression levels and T cell CD4 Th2 infiltration scores estimated by xCELL after outlier removal. PSMB5, PSMB6, PSMD11, and PSMD14 were analyzed because these subunits showed representative positive associations with T cell CD4 Th2 infiltration in the main analysis. The upper panels show correlations after 1.5× interquartile range-based outlier removal (A), and the lower panels show correlations after 1% trimming (B). Pearson correlation coefficients and P values are indicated in each plot.

Supplementary Fig. S4

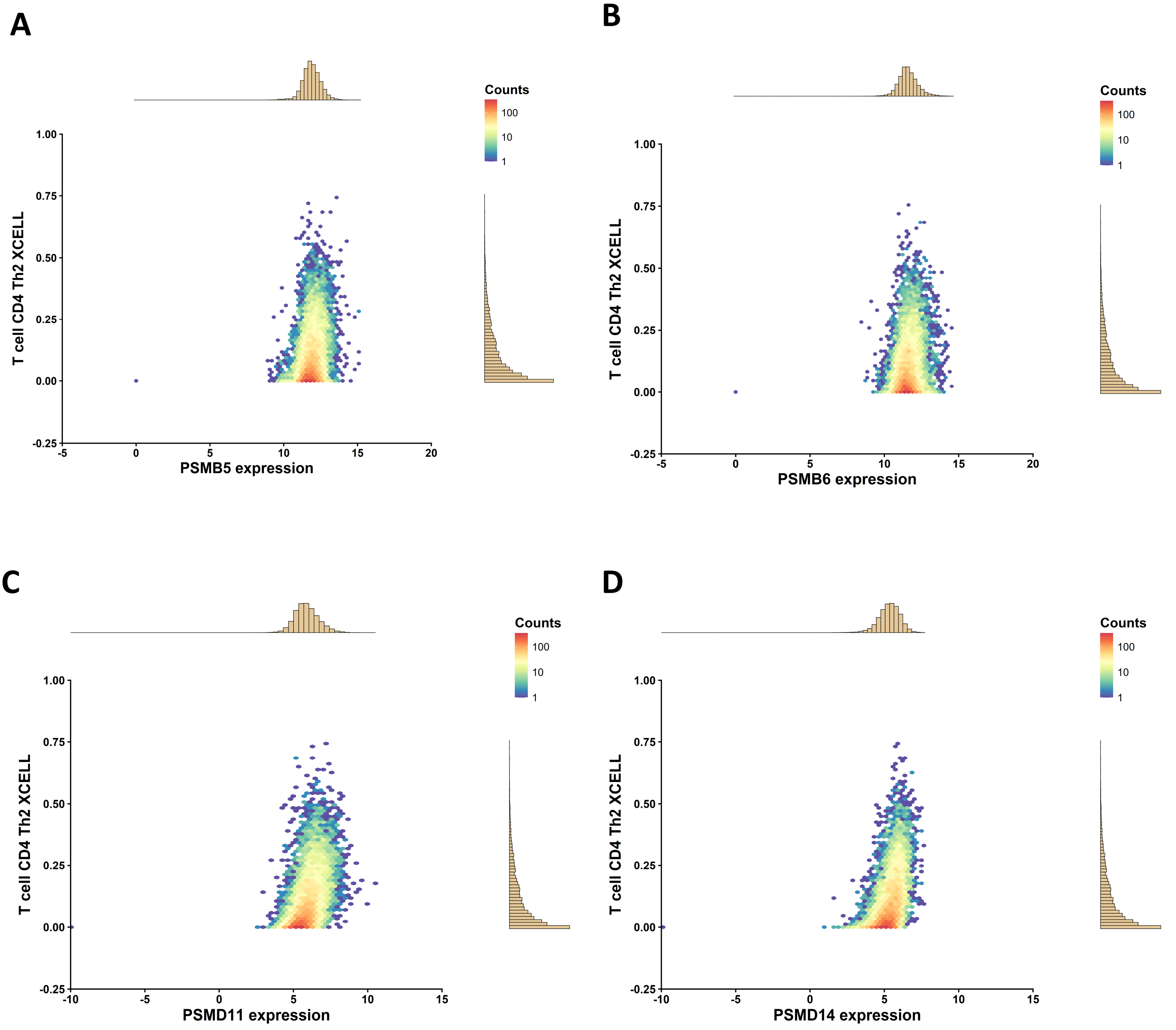

Supplementary Fig. S4. Joint distribution of proteasome subunit expression and $CD4^+$ Th2 T-cell infiltration. Density-colored scatter plots with marginal histograms illustrate the distribution of proteasome subunit expression levels and xCell-inferred $CD4^+$ Th2 T-cell infiltration scores. The analysis includes PSMB5(A), PSMB6(B), PSMD11(C), and PSMD14(D). Each point represents an individual tumor sample, and the color scale indicates local sample density. The marginal histograms display the distributions of gene expression values and immune infiltration scores, respectively. This visualization clarifies the sample distribution underlying the correlation analysis and enables assessment of whether the observed positive correlations are driven by a small number of outliers or reflect the overall distribution across tumor samples.

Supplementary Fig. S5

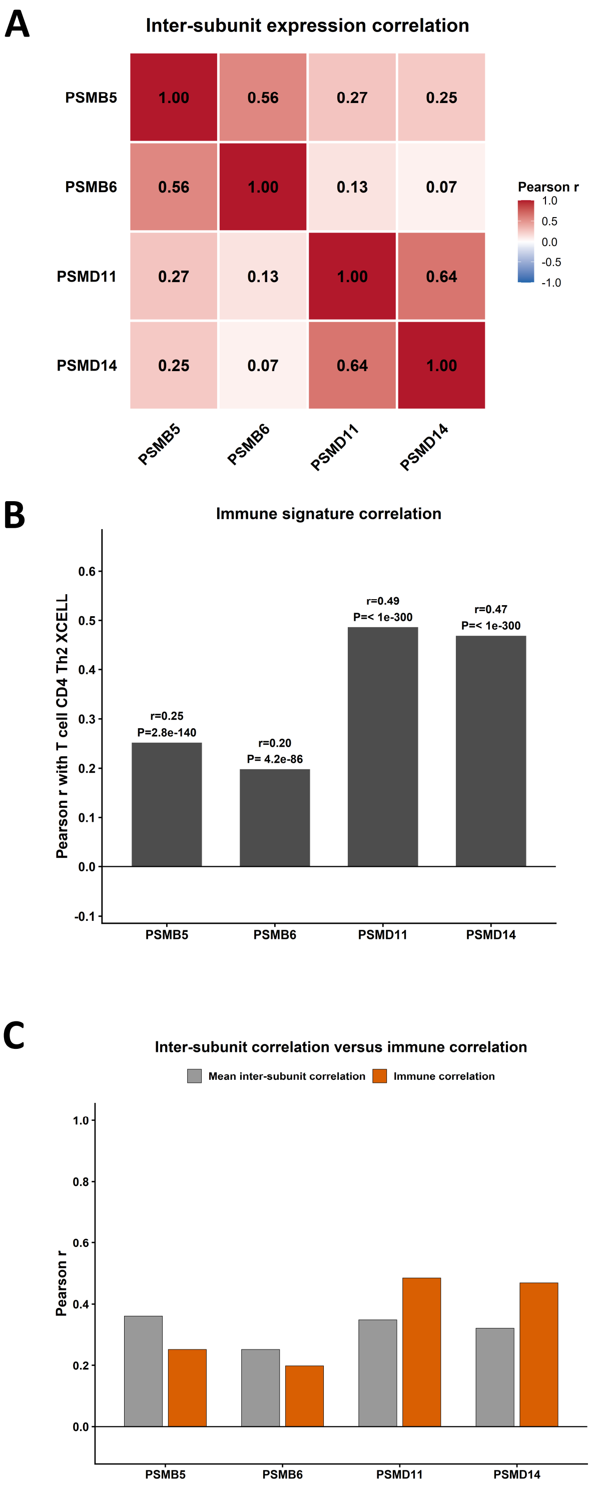

Supplementary Fig. S5. Comparison between inter-subunit expression correlations and immune signature correlations. The heatmap shows Pearson correlation coefficients among PSMB5, PSMB6, PSMD11, and PSMD14 expression levels across tumor samples. The middle bar plot shows Pearson correlation coefficients between each proteasome subunit and T cell CD4 Th2 infiltration scores estimated by xCELL. The lower bar plot compares the mean inter-subunit expression correlation of each gene with its correlation with the T cell CD4 Th2 immune signature. PSMB5 and PSMB6 showed moderate correlations with T cell CD4 Th2 infiltration, whereas PSMD11 and PSMD14 showed stronger immune signature correlations. These analyses indicate that the association between proteasome subunit expression and T cell CD4 Th2 infiltration is not merely a reflection of uniform co-expression among proteasome subunits.

Supplementary Fig. S6

A

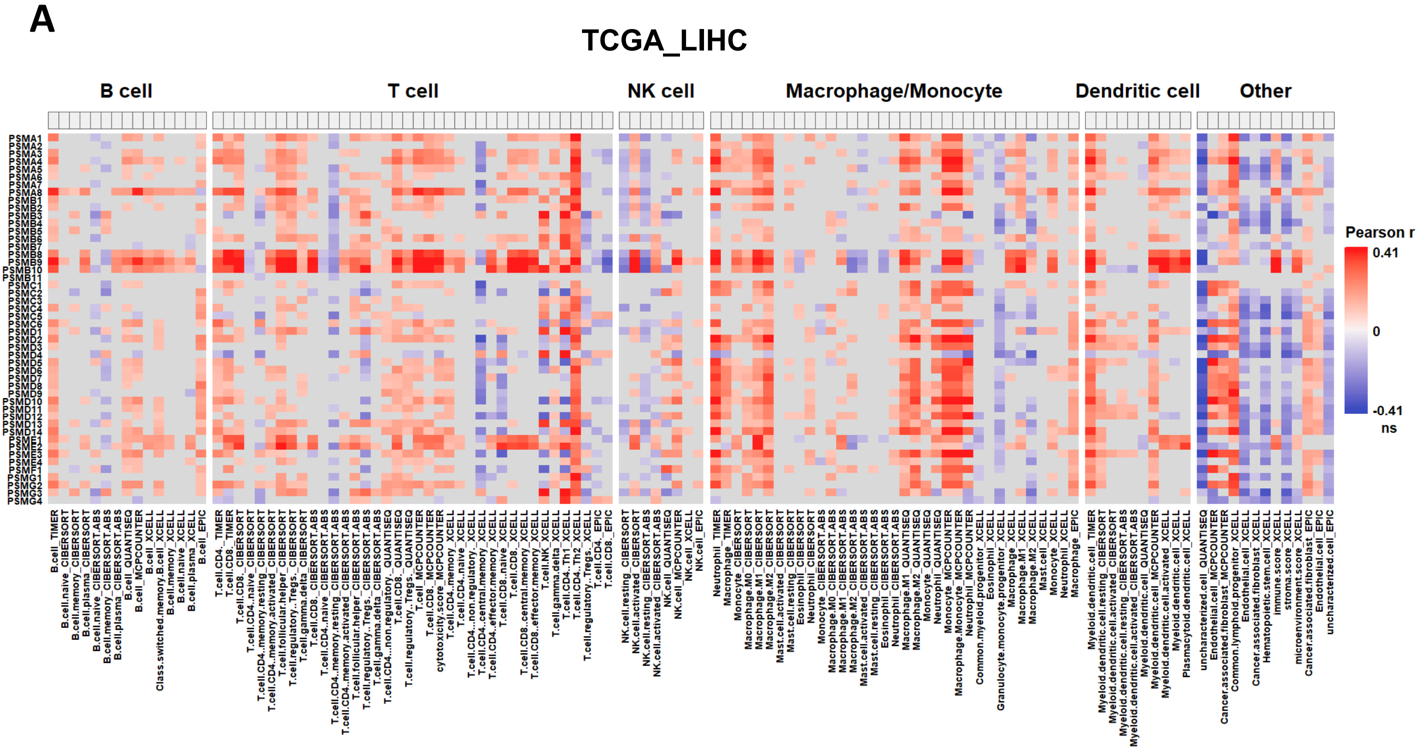

B

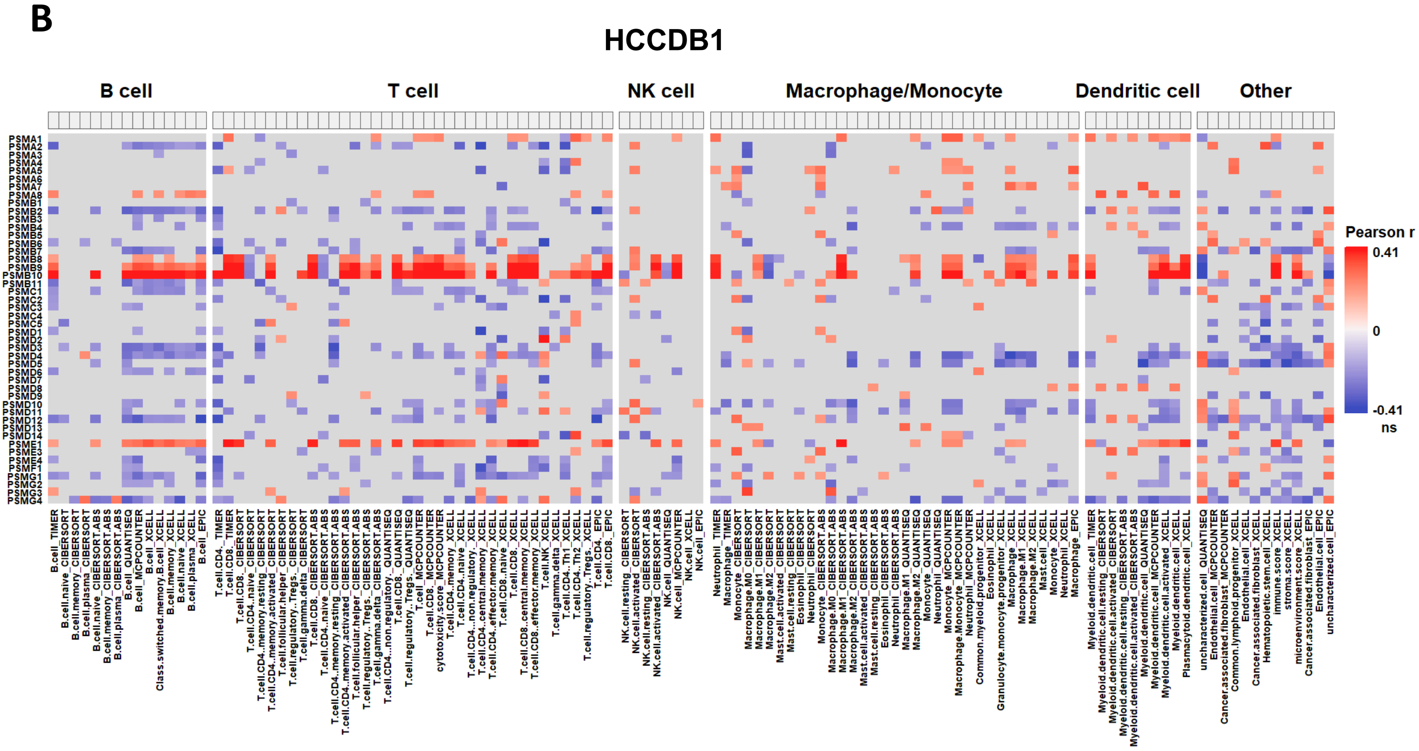

Supplementary Fig. S6. Cohort-specific heatmaps of correlations between proteasome subunit expression and immune cell infiltration signatures in LIHC. Heatmaps show Pearson correlation coefficients between the expression of individual proteasome subunits and immune cell infiltration signatures across TCGA_LIHC and 24 independent HCC cohorts. Rows represent proteasome subunits, and columns represent immune infiltration signatures categorized into B cells, T cells, NK cells, macrophage/monocyte, dendritic cells, and other immune/stromal signatures. Red indicates positive correlation, blue indicates negative correlation, and gray indicates non-significant associations. Color intensity reflects the magnitude of the Pearson correlation coefficient.

Supplementary Fig. S6_continued

C

HCCDB3

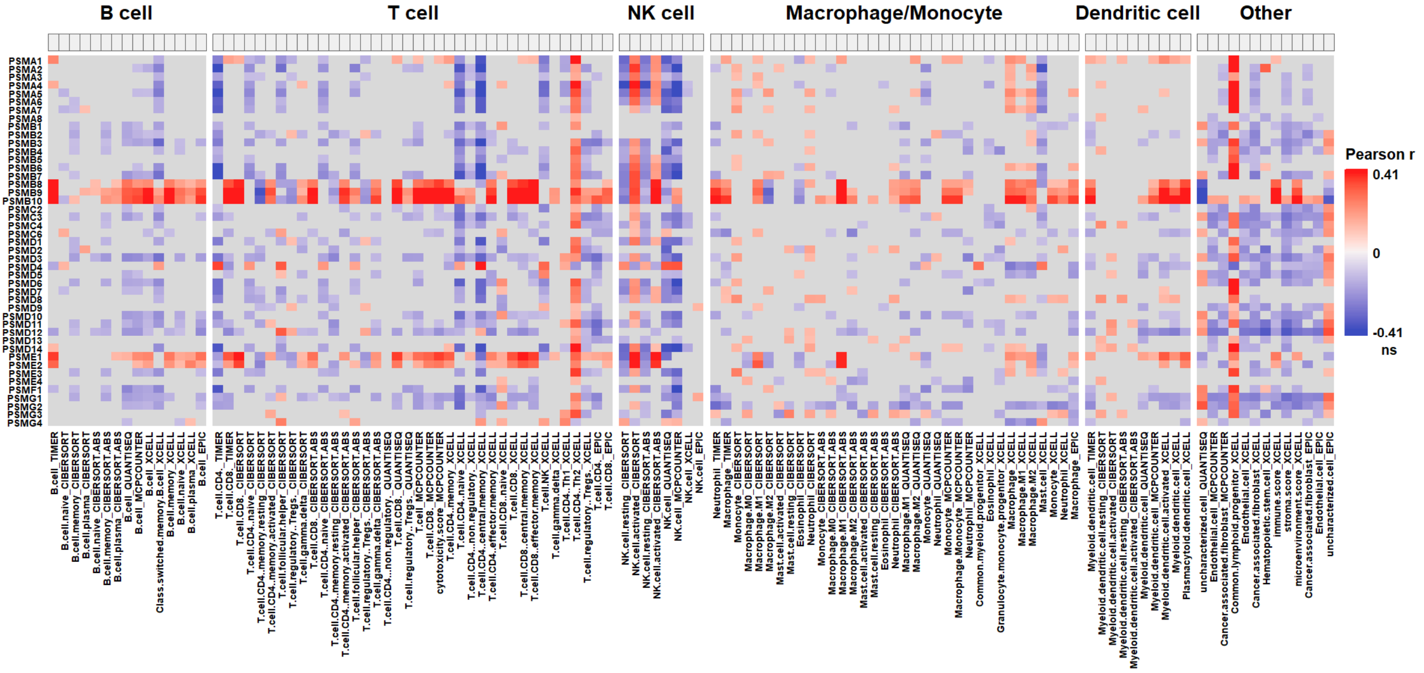

D

HCCDB4

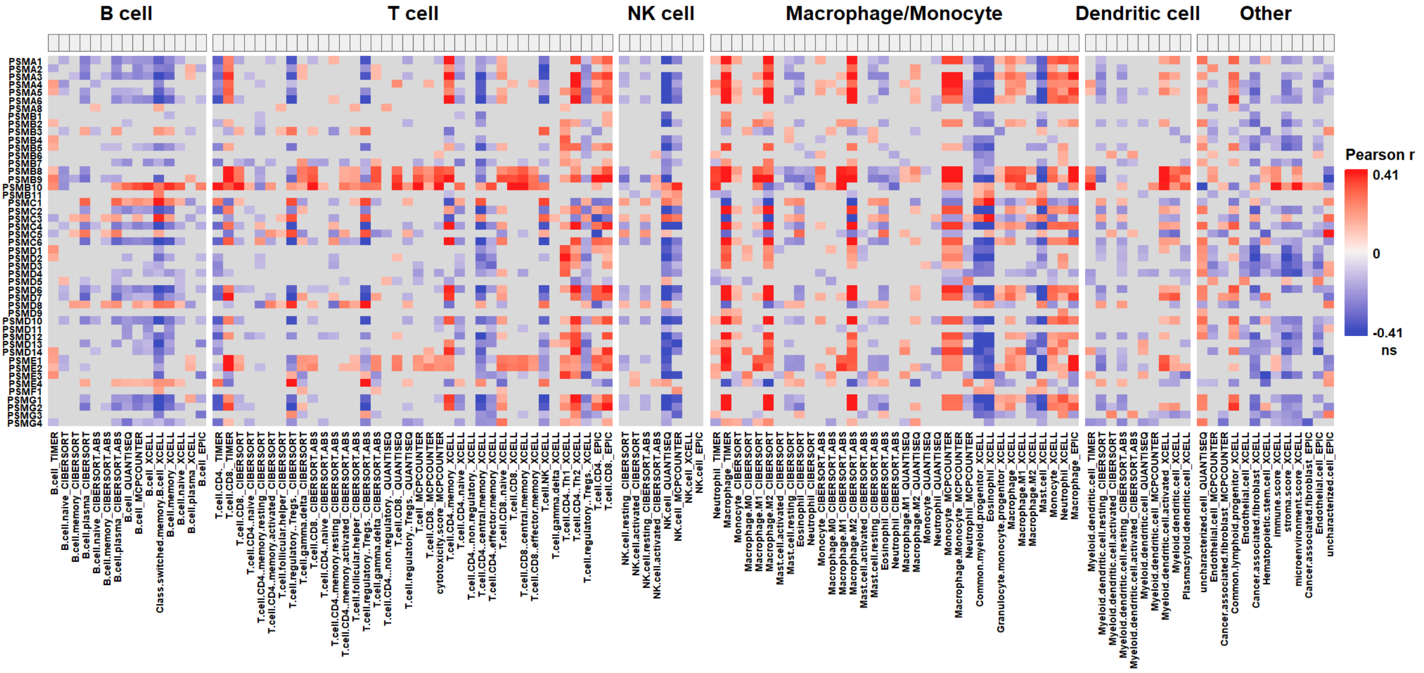

Supplementary Fig. S6_continued

E

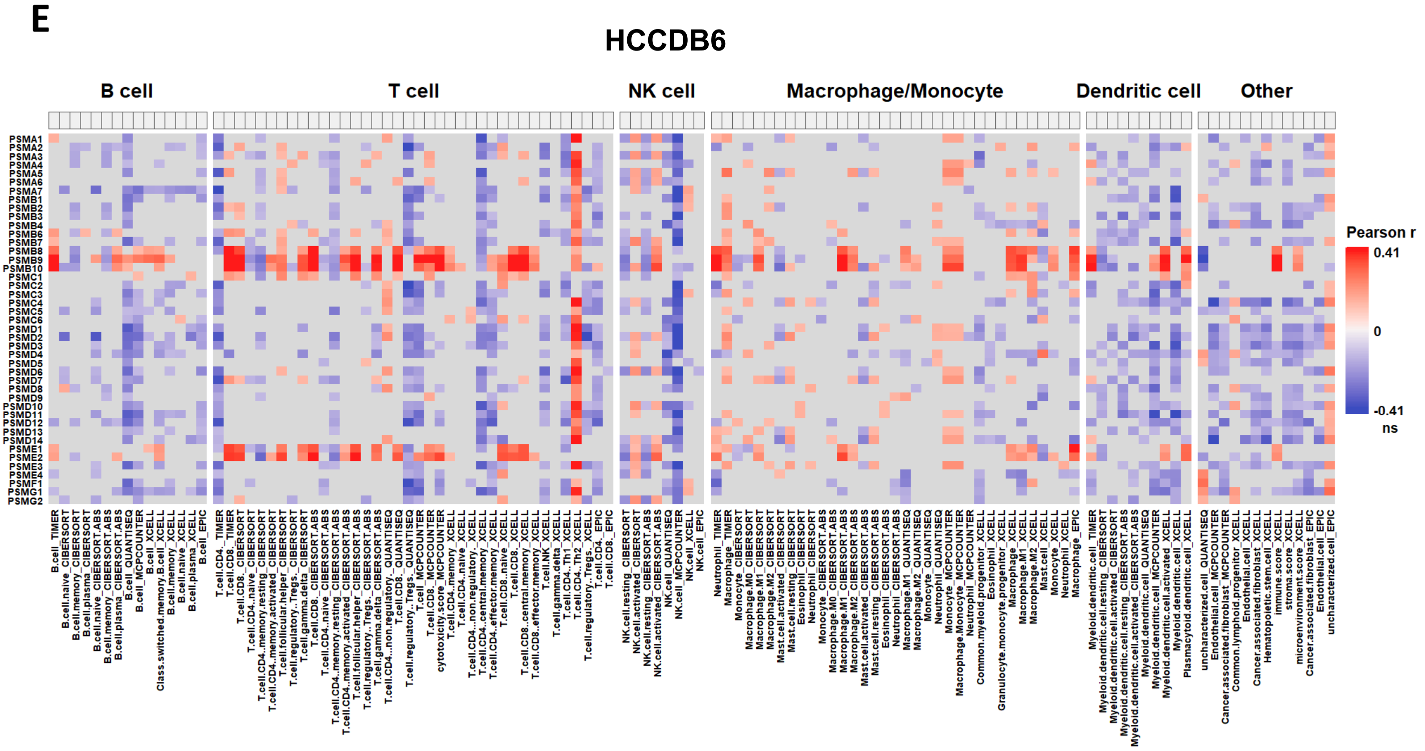

F

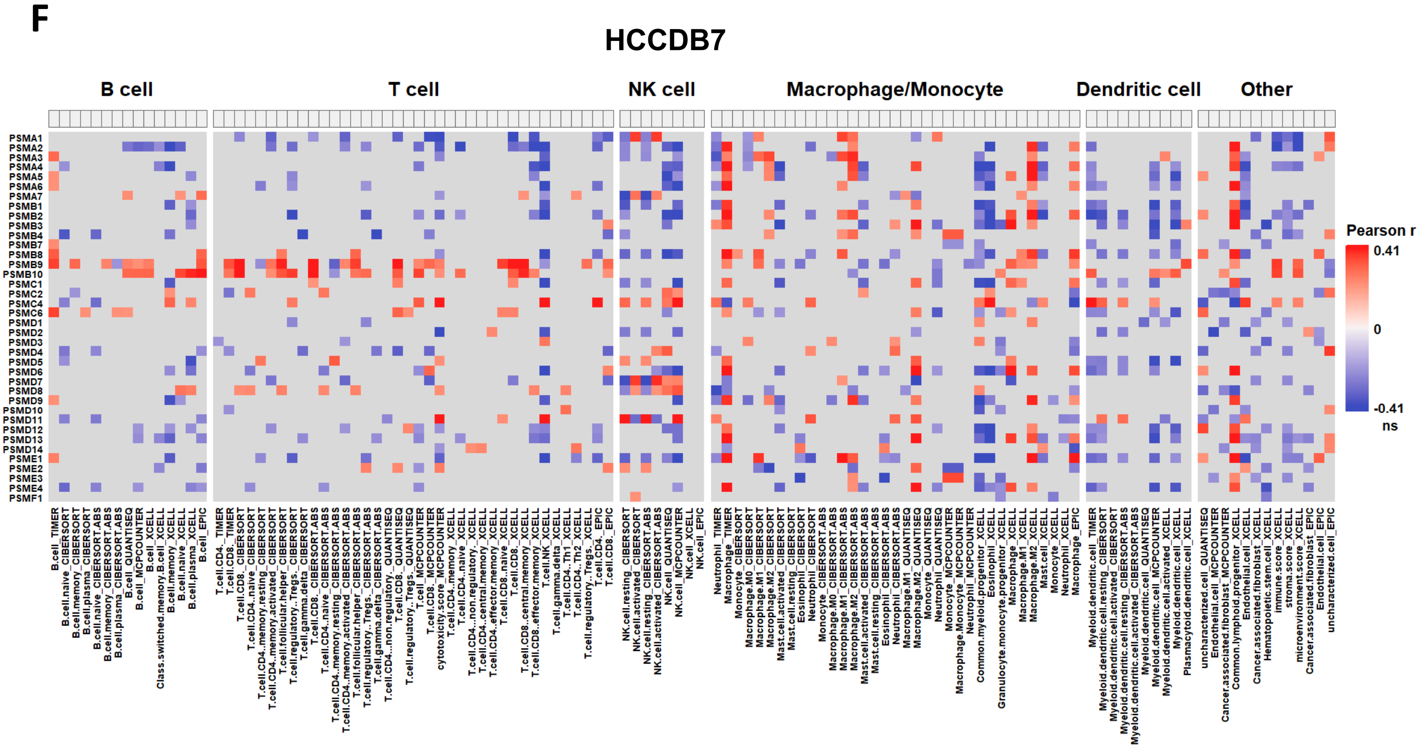

Supplementary Fig. S6_continued

G

HCCDB8

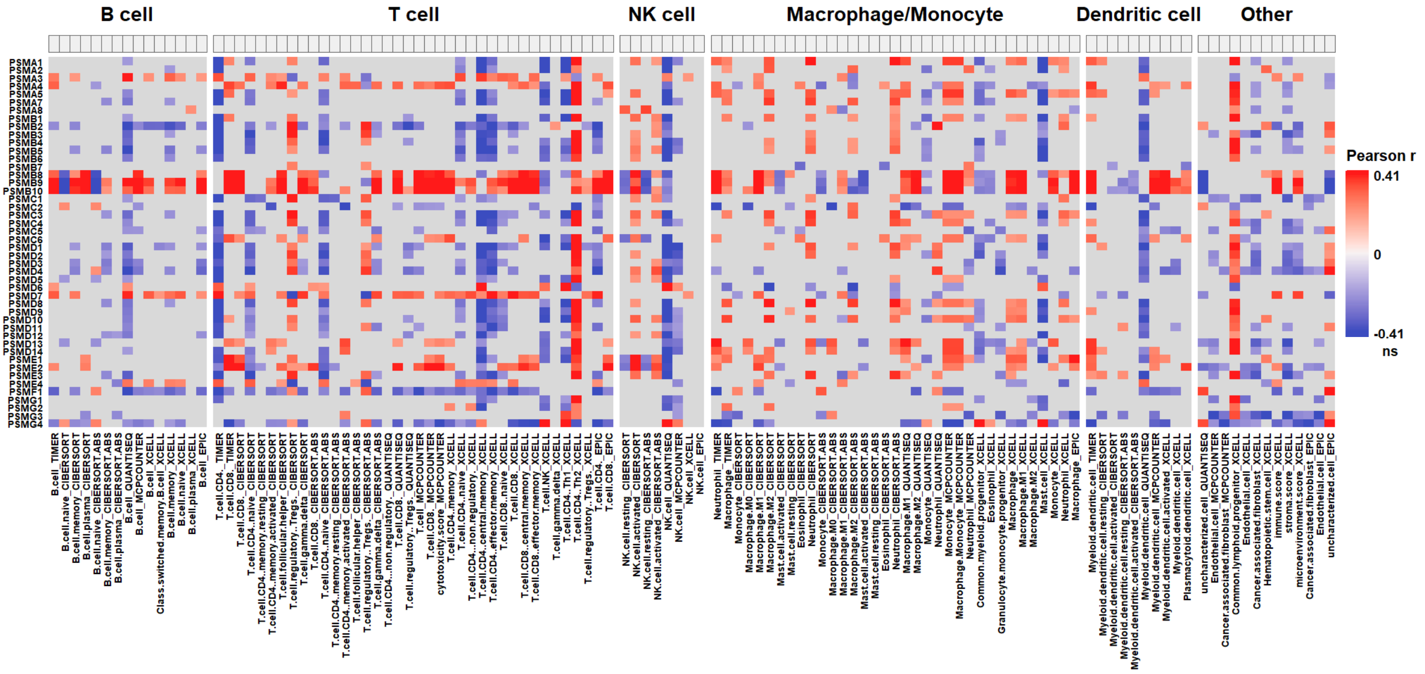

H

HCCDB9

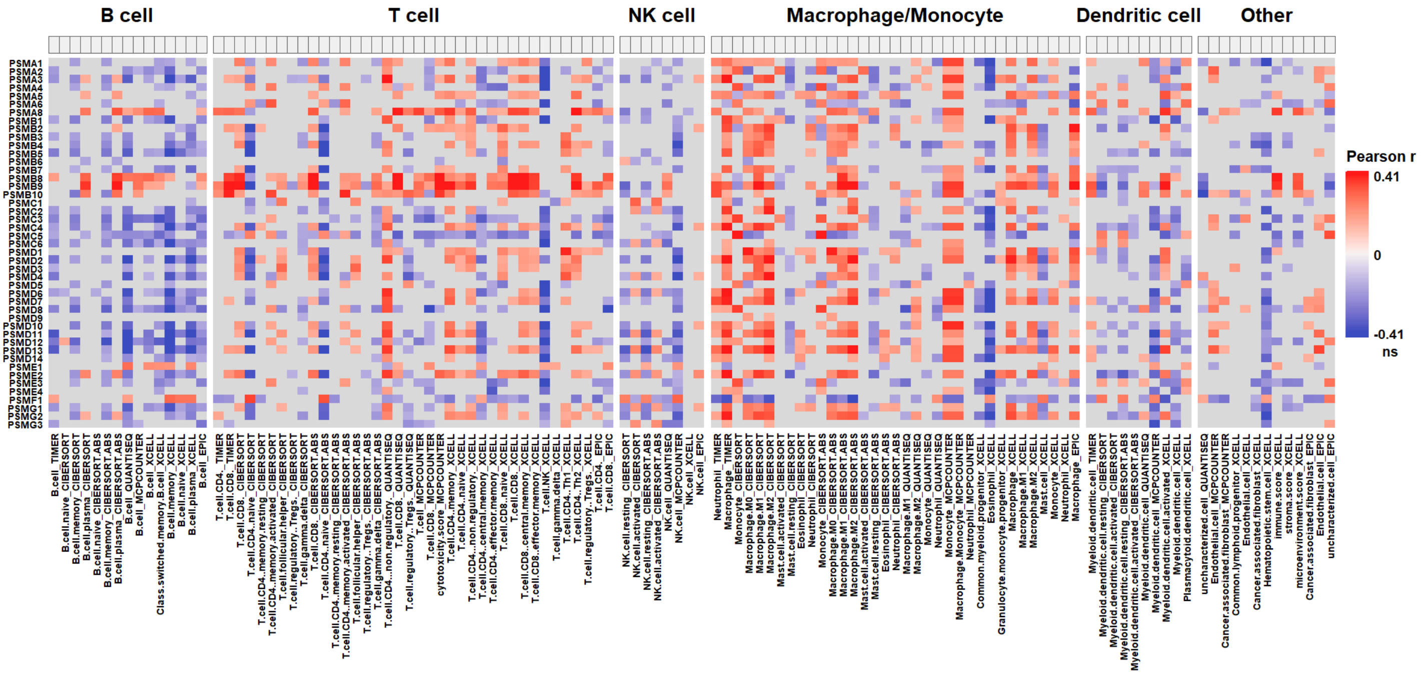

Supplementary Fig. S6_continued

I

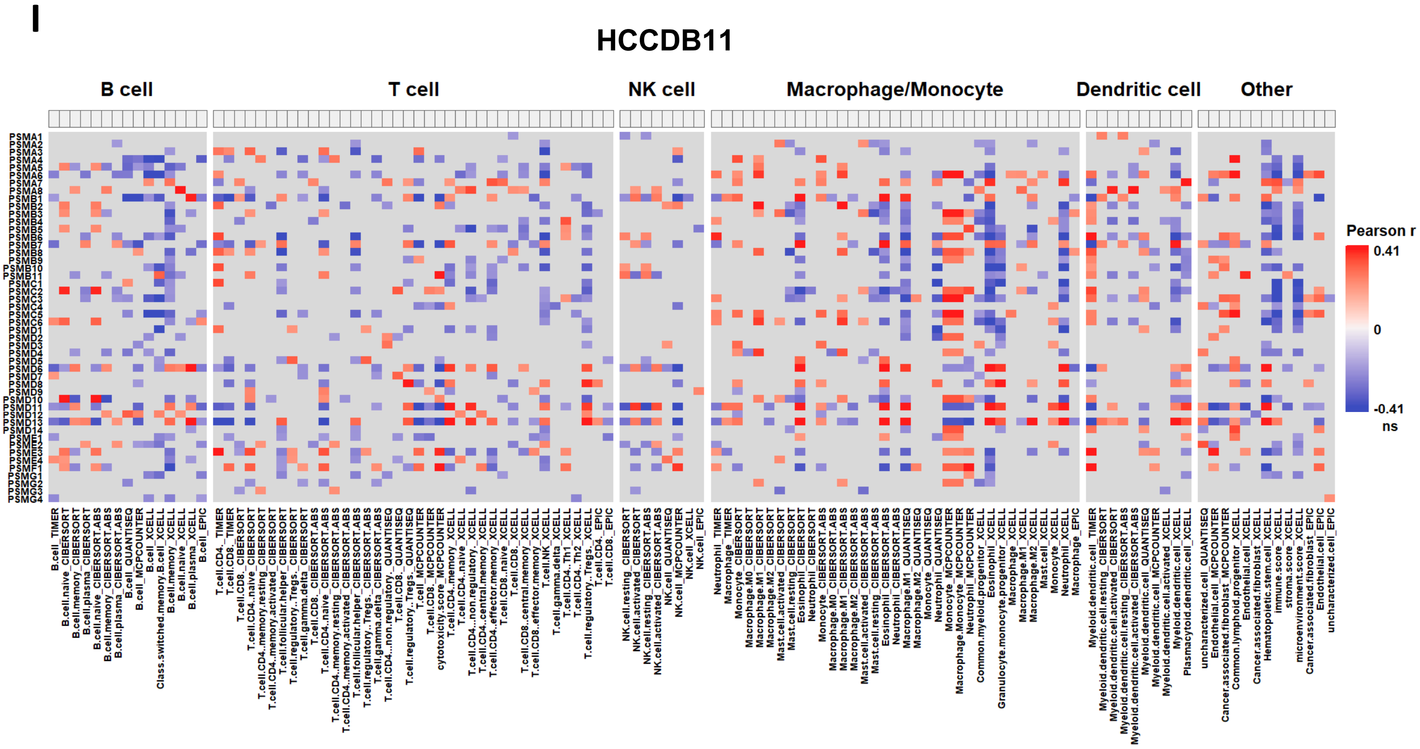

J

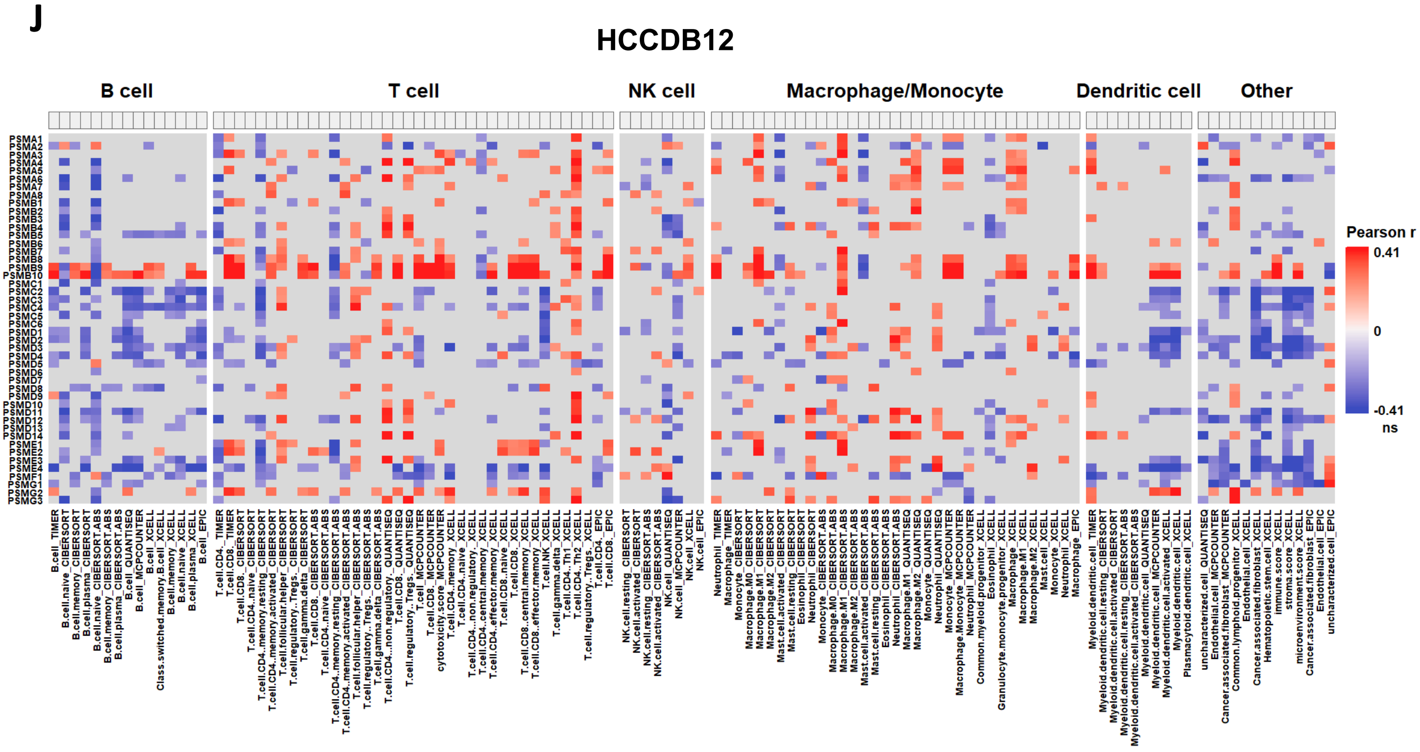

Supplementary Fig. S6_continued

K

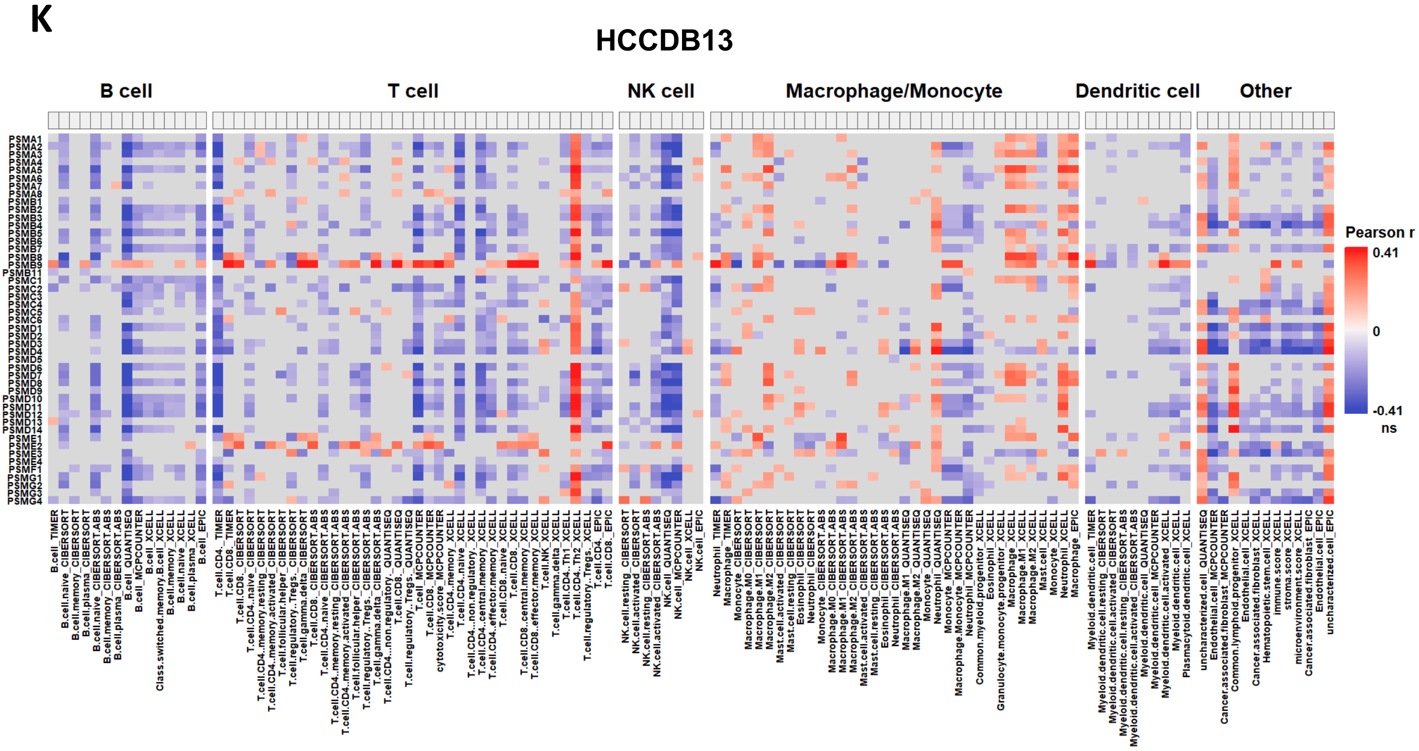

L

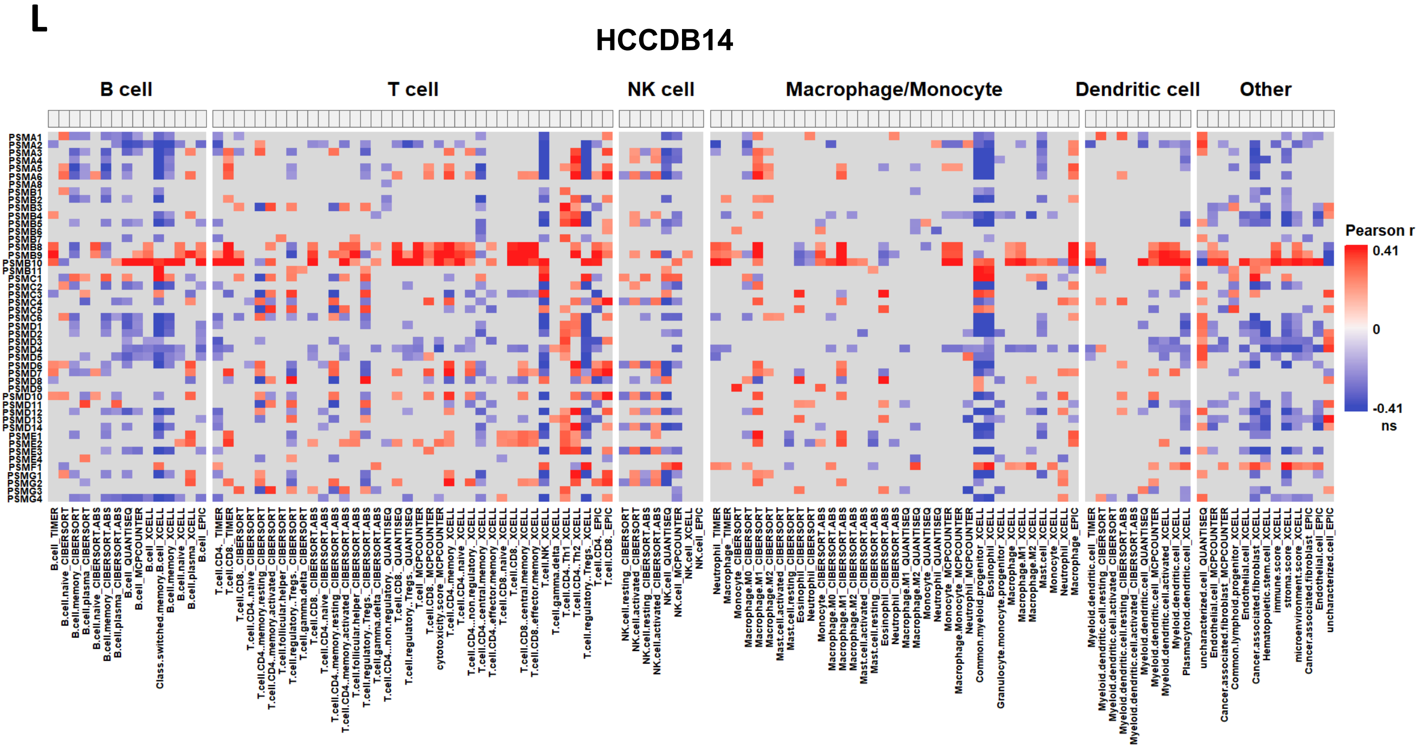

Supplementary Fig. S6_continued

M

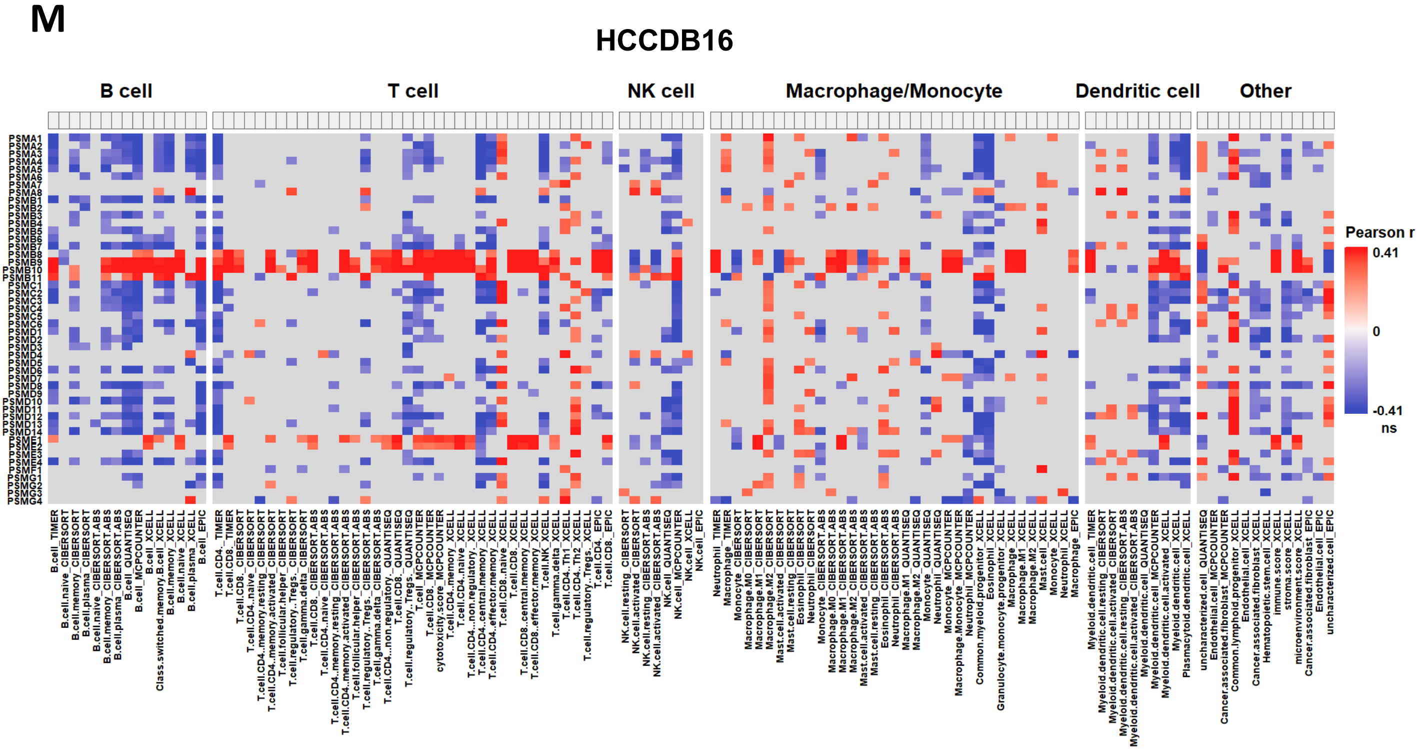

N

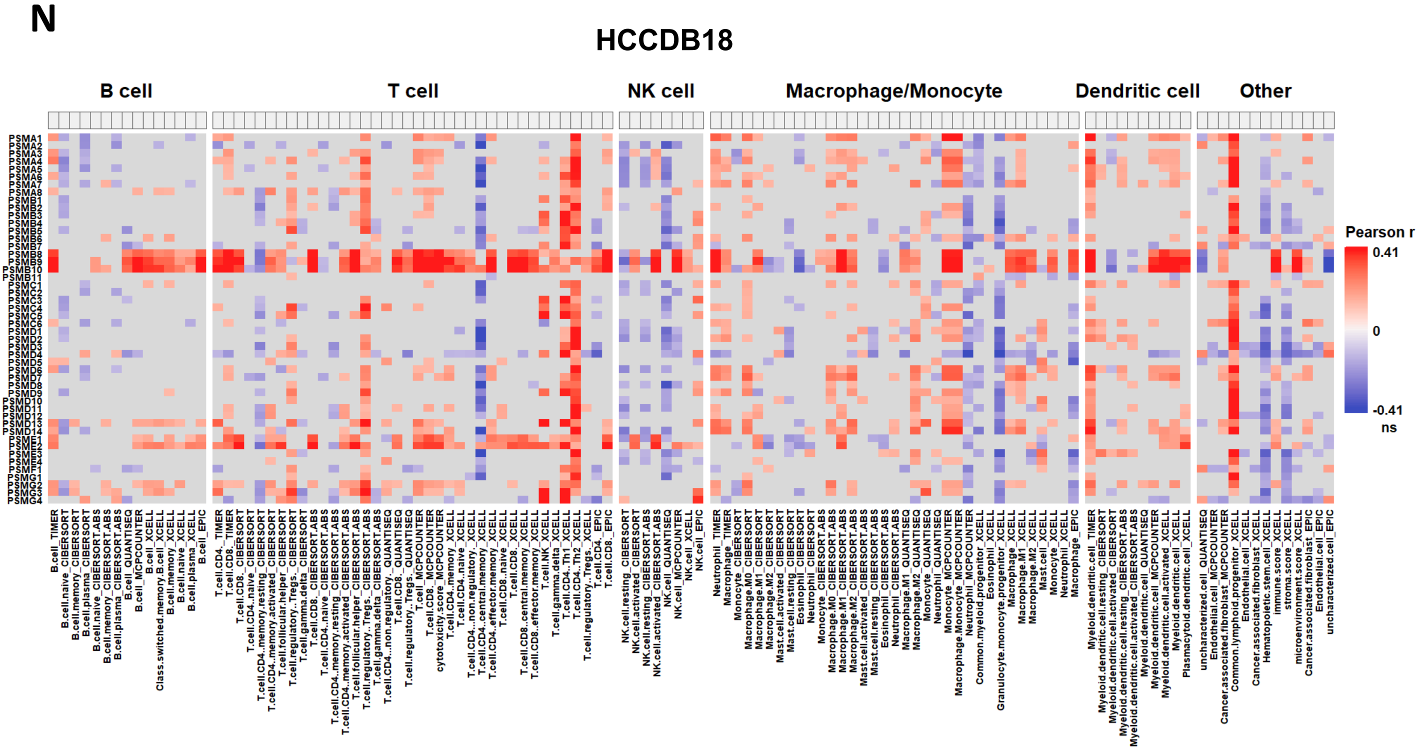

Supplementary Fig. S6_continued

O

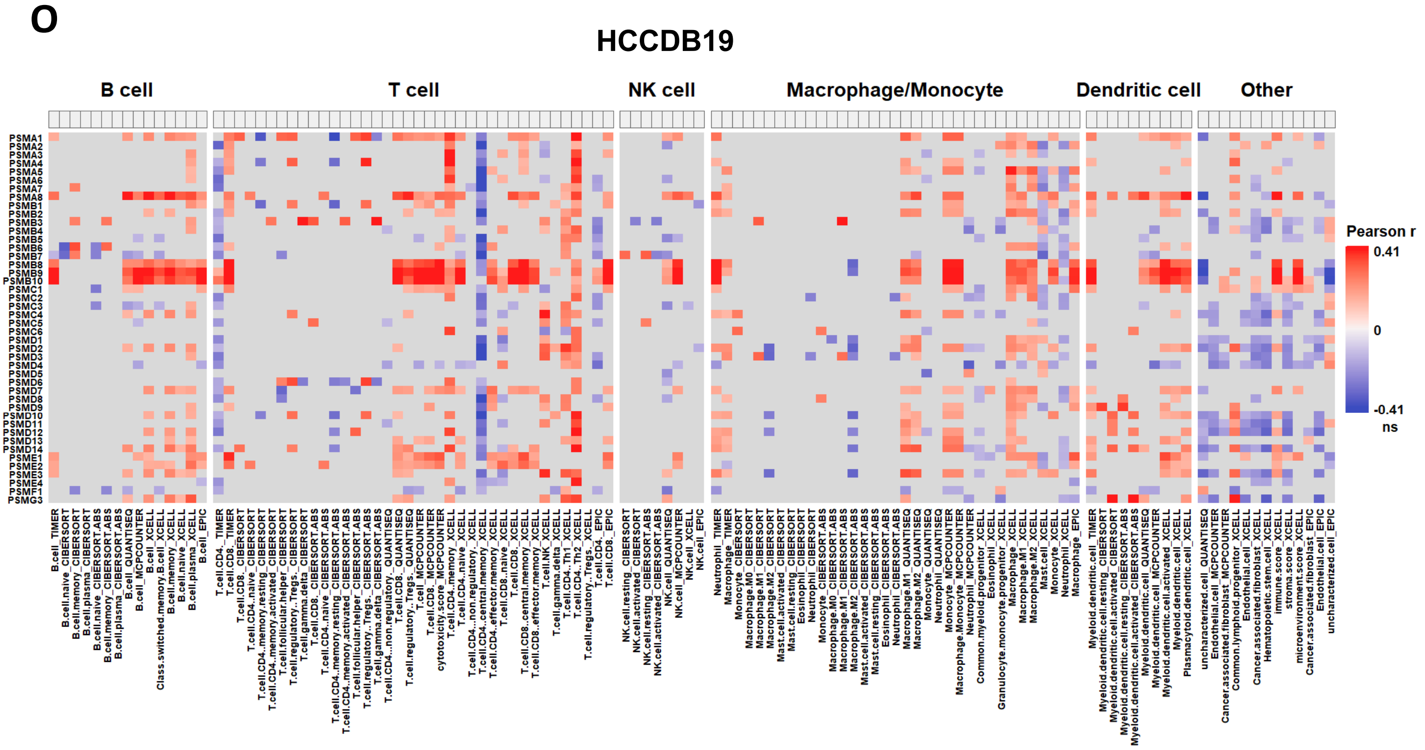

P

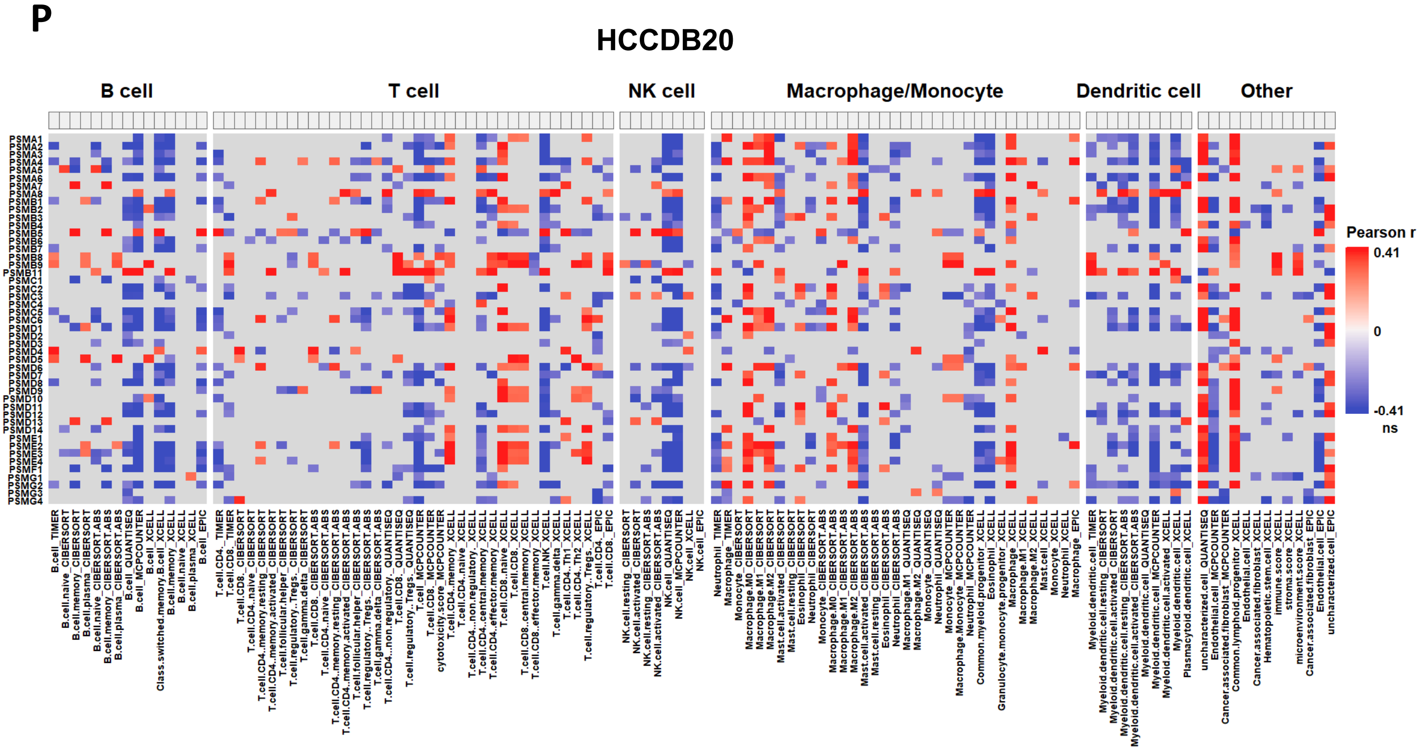

Supplementary Fig. S6_continued

Q

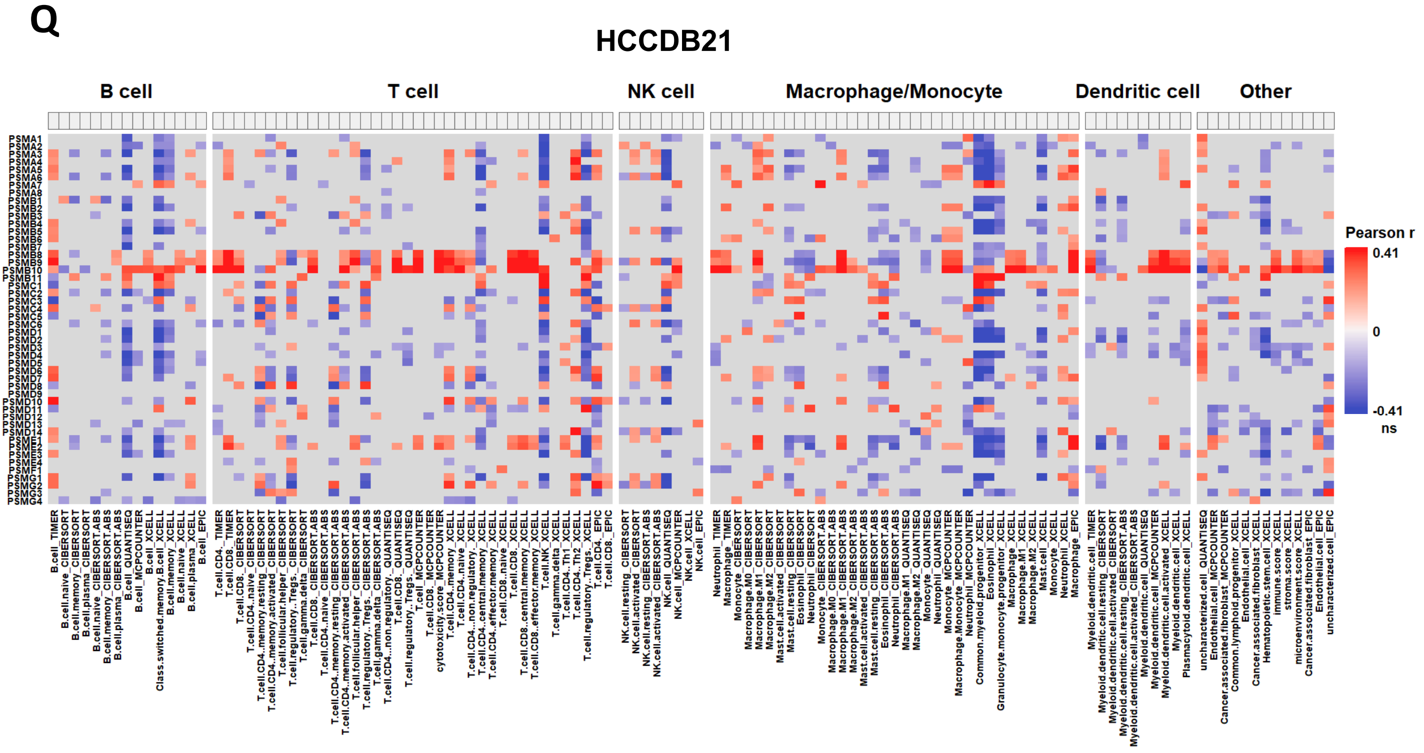

R

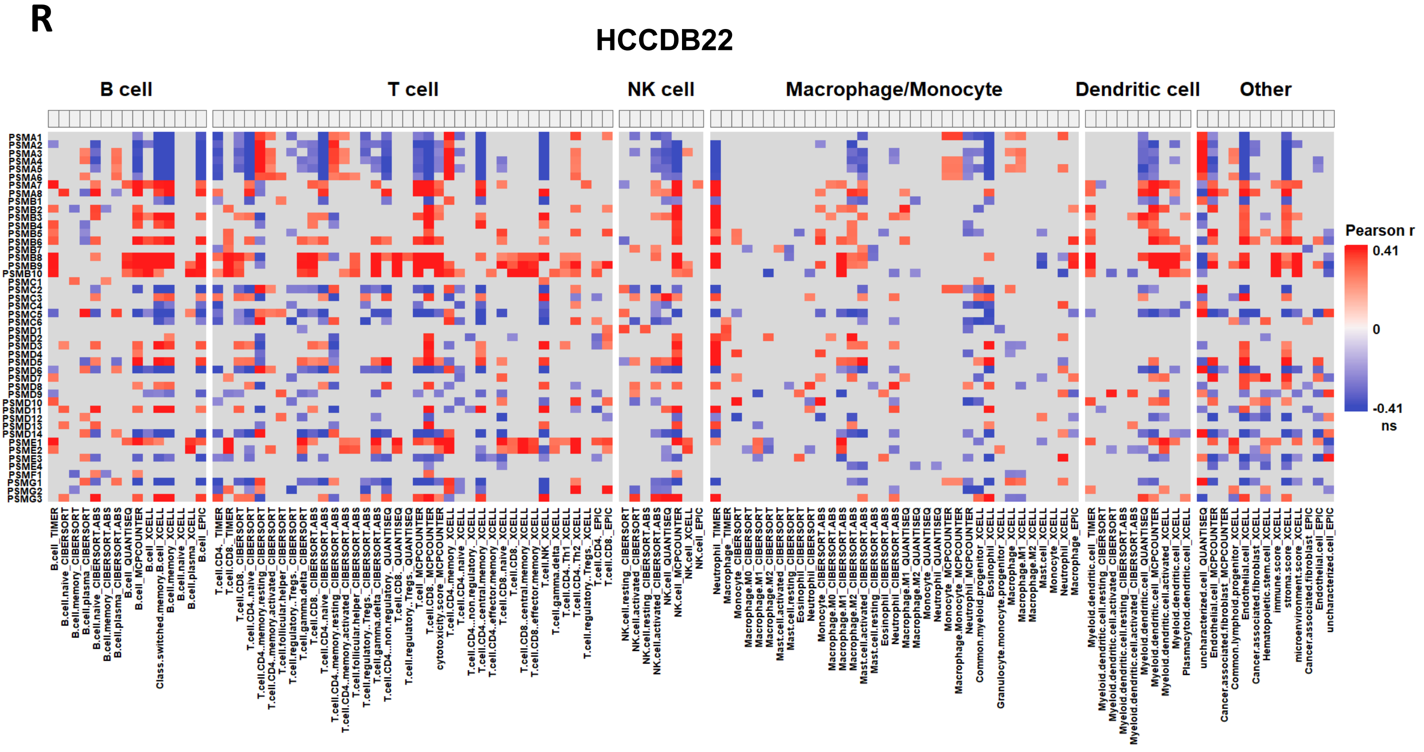

Supplementary Fig. S6_continued

S

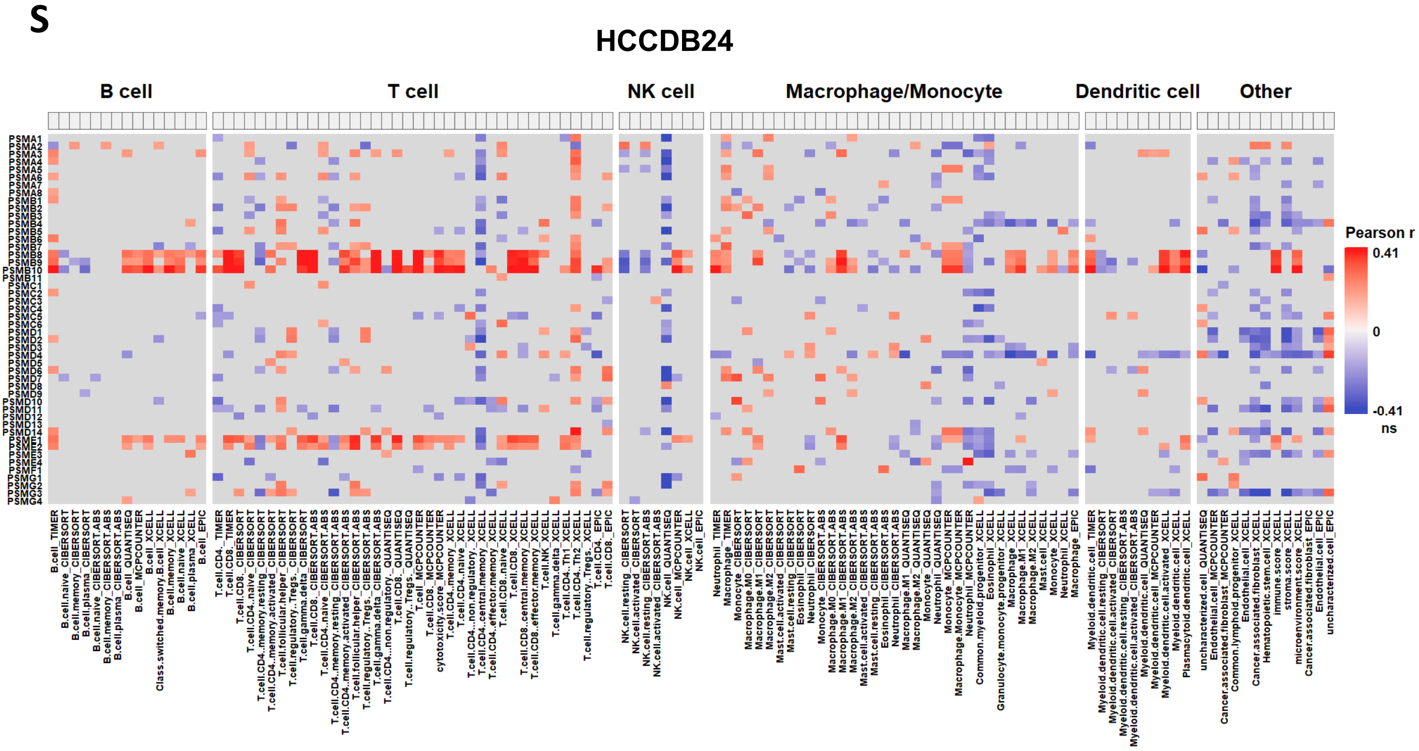

T

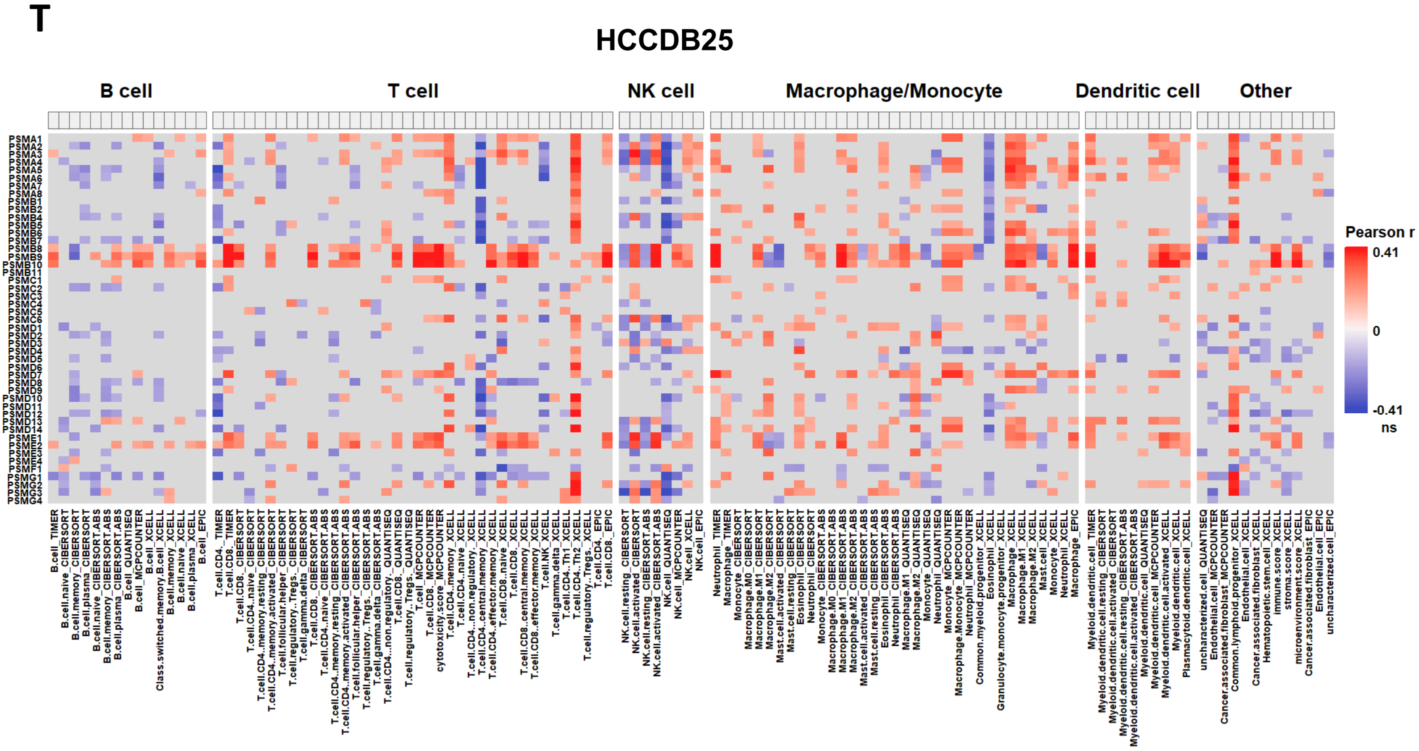

Supplementary Fig. S6_continued

U

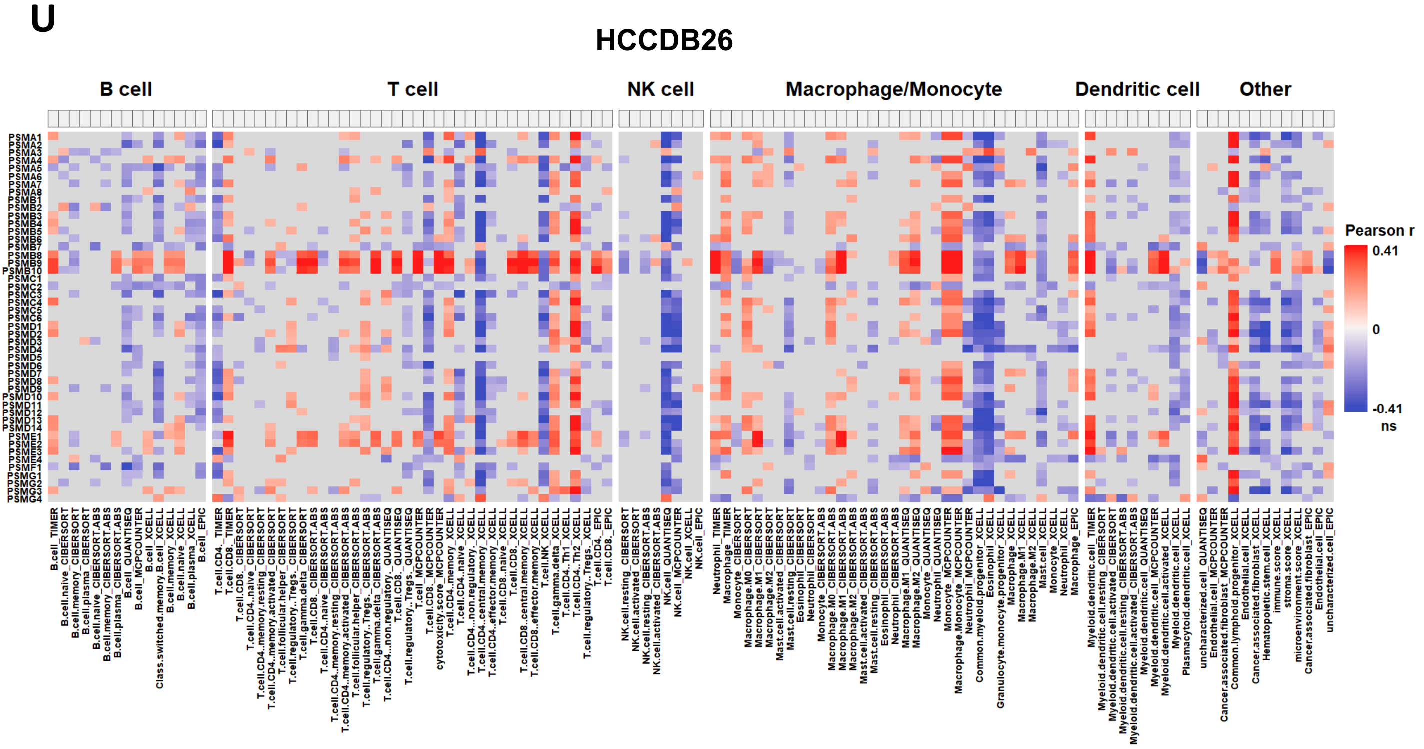

V

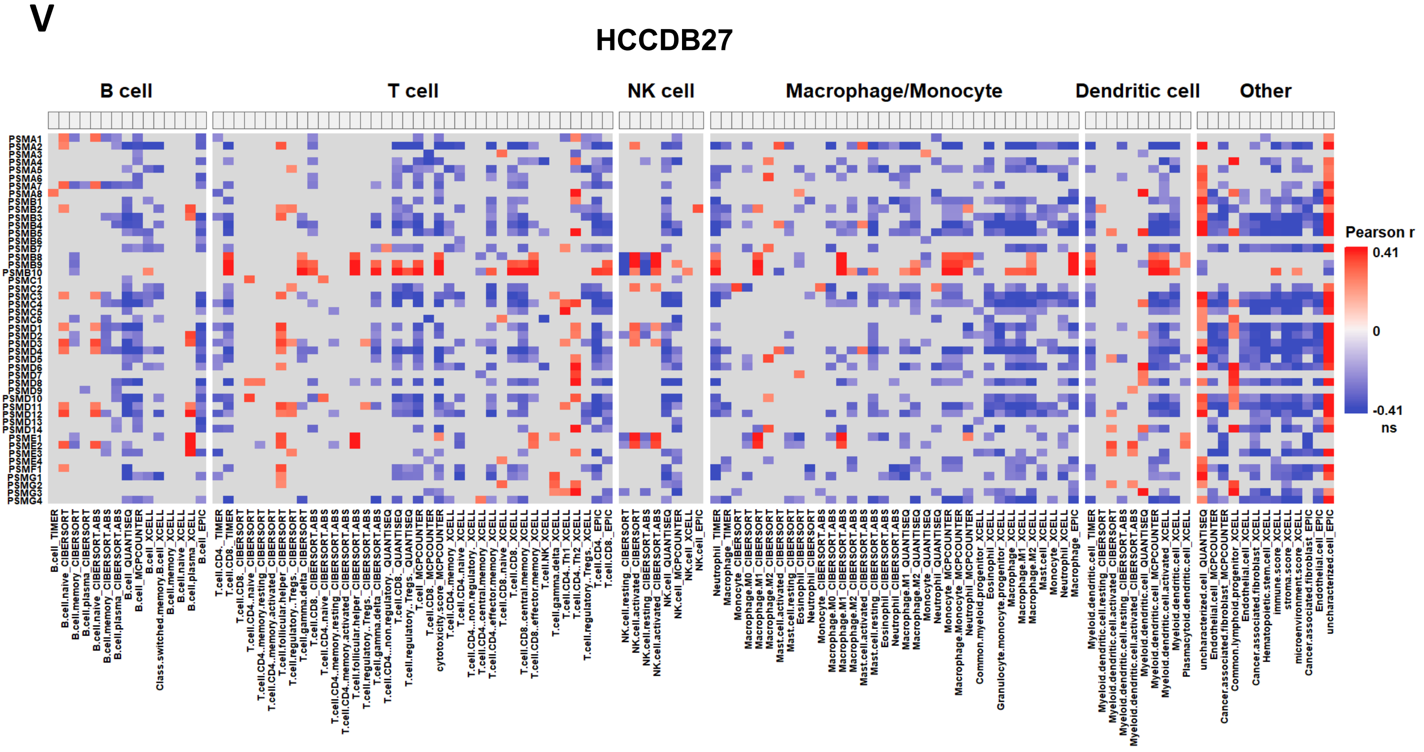

Supplementary Fig. S6_continued

W

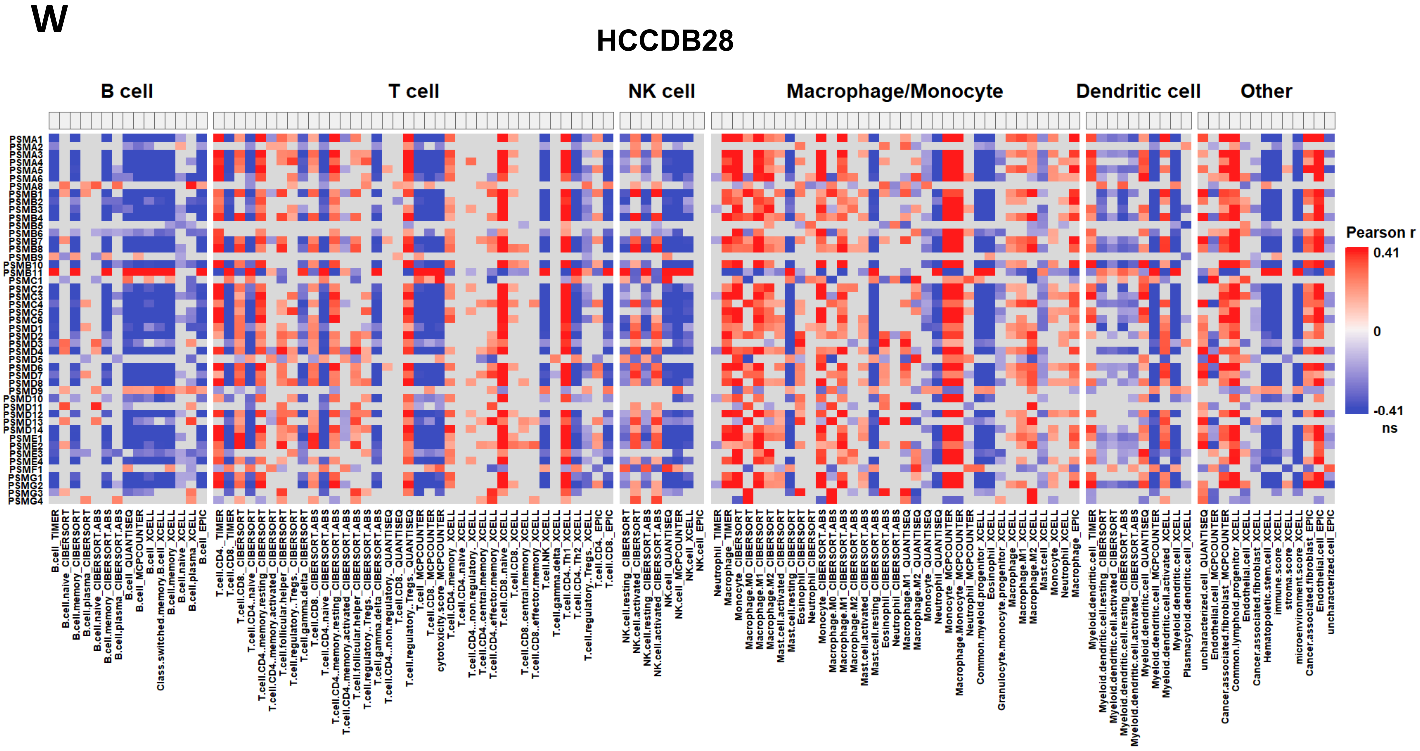

X

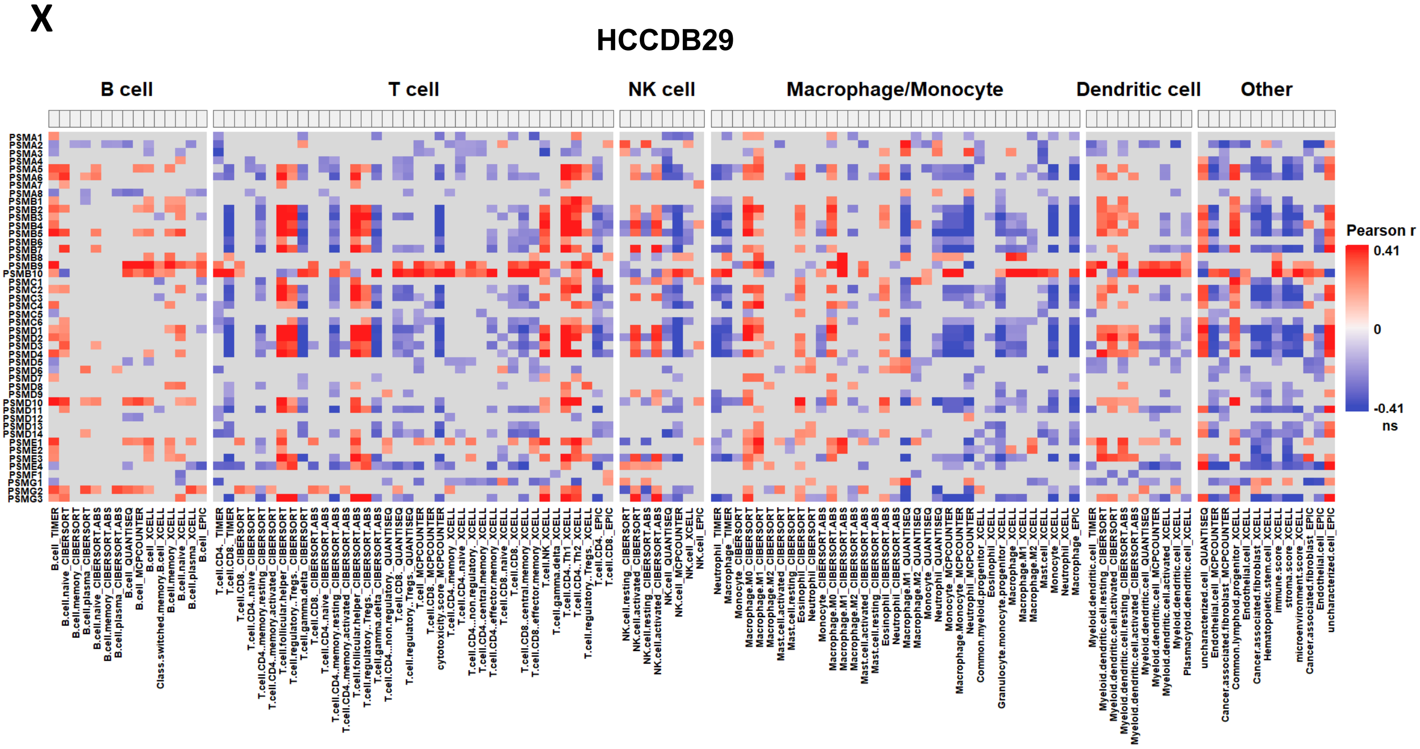

Supplementary Fig. S6_continued

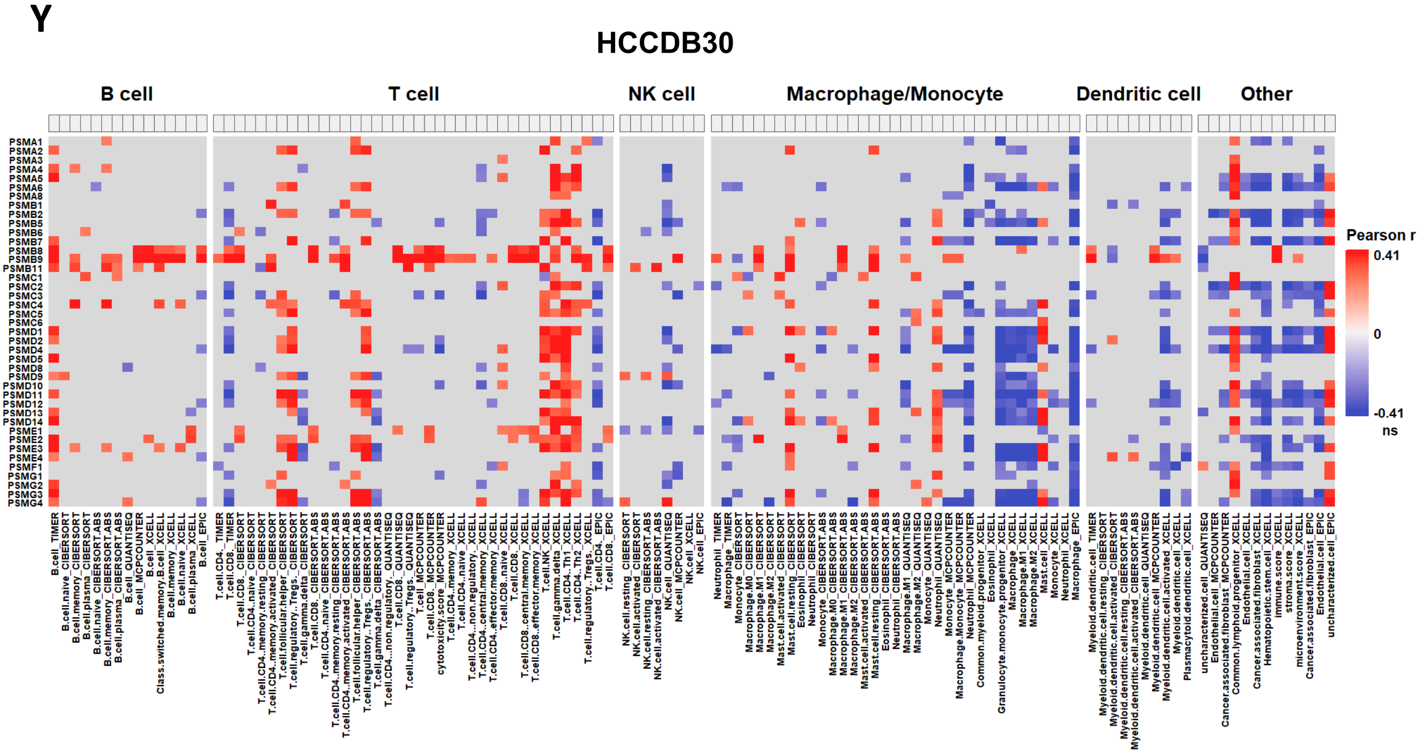

Supplementary Table S1. Cancer type classification of depmap model cell lines

DepMapID	subtype	primaryMet	growthPattern	displayName	Cancer Type
ACH-001598	Adrenocortical Carcinoma	Primary	Adherent	NCIH295R	ACC
ACH-001401	Adrenocortical Carcinoma	Primary	Adherent	SW13	ACC
ACH-001119	Anaplastic Large-Cell Lymphoma ALK Negative	Metastatic	Suspension	MAC2A	ALCL
ACH-001068	Anaplastic Large-Cell Lymphoma ALK Negative		Unknown	FEPD	ALCL
ACH-001060	Anaplastic Large-Cell Lymphoma ALK Negative		Suspension	DL40	ALCL
ACH-000233	Anaplastic Large-Cell Lymphoma ALK Positive		Suspension	DEL	ALCL
ACH-000338	Anaplastic Large-Cell Lymphoma ALK Positive	Primary	Suspension	SR786	ALCL
ACH-000773	Anaplastic Large-Cell Lymphoma ALK Positive		Suspension	KIJK	ALCL
ACH-000226	Anaplastic Large-Cell Lymphoma ALK Positive		Suspension	SUPM2	ALCL
ACH-000664	Anaplastic Large-Cell Lymphoma ALK Positive		Suspension	SUDHL1	ALCL
ACH-000053	Anaplastic Large-Cell Lymphoma ALK Positive		Suspension	KARPAS299	ALCL
ACH-001111	Anaplastic Large-Cell Lymphoma ALK Positive		Suspension	L82	ALCL
ACH-001520	B-Lymphoblastic Leukemia/Lymphoma	Primary	Suspension	HG3	ALL
ACH-001680	B-Lymphoblastic Leukemia/Lymphoma	Metastatic	Suspension	U698M	ALL
ACH-001669	B-Lymphoblastic Leukemia/Lymphoma	Primary	Suspension	TANOUE	ALL
ACH-001485	B-Lymphoblastic Leukemia/Lymphoma	Primary	Adherent	CII	ALL
ACH-002059	B-Lymphoblastic Leukemia/Lymphoma	Primary	Suspension	P30OHK	ALL
ACH-000938	B-Lymphoblastic Leukemia/Lymphoma	Primary	Suspension	NALM6	ALL
ACH-002643	B-Lymphoblastic Leukemia/Lymphoma		Suspension	GRANTA452	ALL
ACH-000151	B-Lymphoblastic Leukemia/Lymphoma	Primary	Suspension	JM1	ALL
ACH-000038	B-Lymphoblastic Leukemia/Lymphoma	Primary	Suspension	EHEB	ALL
ACH-000130	B-Lymphoblastic Leukemia/Lymphoma	Primary	Suspension	NALM19	ALL
ACH-000020	B-Lymphoblastic Leukemia/Lymphoma with Hypodiploidy	Primary	Suspension	MHHCALL2	ALL
ACH-001993	B-Lymphoblastic Leukemia/Lymphoma with Hypodiploidy	Primary	Suspension	NALM16	ALL
ACH-000728	hoblastic Leukemia/Lymphoma with t(1;19)(q23;p13.3);TCF3-PBX1		Adherent	KASUMI2	ALL
ACH-000070	hoblastic Leukemia/Lymphoma with t(1;19)(q23;p13.3);TCI	Primary	Suspension	697	ALL
ACH-000032	hoblastic Leukemia/Lymphoma with t(1;19)(q23;p13.3);TCI	Primary	Mixed	MHHCALL3	ALL
ACH-000922	hoblastic Leukemia/Lymphoma with t(1;19)(q23;p13.3);TCF3-PBX1		Suspension	RCHACV	ALL
ACH-000960	olastic Leukemia/Lymphoma with t(12;21)(p13.2;q22.1); ET	Primary	Suspension	REH	ALL
ACH-001736	olastic Leukemia/Lymphoma with t(12;21)(p13.2;q22.1); ET	Primary	Suspension	HB1119	ALL
ACH-001209	hoblastic Leukemia/Lymphoma with t(9;22)(q34.1;q11.2);BCR-ABL1		Mixed	TOM1	ALL
ACH-003101	hoblastic Leukemia/Lymphoma with t(9;22)(q34.1;q11.2);BCR-ABL1		Suspension	Z119	ALL
ACH-003103	hoblastic Leukemia/Lymphoma with t(9;22)(q34.1;q11.2);BCR-ABL1		Suspension	Z181	ALL
ACH-000059	hoblastic Leukemia/Lymphoma with t(9;22)(q34.1;q11.2);B(	Primary	Suspension	SUPB15	ALL
ACH-000874	oblastic Leukemia/Lymphoma with t(v;11q23.3);KMT2A Re	Primary	Suspension	RS411	ALL
ACH-001735	oblastic Leukemia/Lymphoma with t(v;11q23.3);KMT2A Re	Primary	Suspension	SEMK2	ALL
ACH-001106	oblastic Leukemia/Lymphoma with t(v;11q23.3);KMT2A Re	Primary	Suspension	KOPN8	ALL
ACH-000782	oblastic Leukemia/Lymphoma with t(v;11q23.3);KMT2A Re	Primary	Suspension	SEM	ALL
ACH-000156	B-Lymphoblastic Leukemia/Lymphoma, BCR-ABL1 Like	Primary	Adherent	MHHCALL4	ALL
ACH-000492	B-Lymphoblastic Leukemia/Lymphoma, BCR-ABL1 Like	Primary	Suspension	MUTZ5	ALL
ACH-000104	Early T-Cell Precursor Lymphoblastic Leukemia	Primary	Suspension	LOUCY	ALL
ACH-001134	T-Lymphoblastic Leukemia/Lymphoma		Suspension	MYLA	ALL
ACH-000937	T-Lymphoblastic Leukemia/Lymphoma		Suspension	PF382	ALL
ACH-000519	T-Lymphoblastic Leukemia/Lymphoma	Primary	Suspension	PEER	ALL
ACH-000942	T-Lymphoblastic Leukemia/Lymphoma	Primary	Suspension	HPBALL	ALL
ACH-000918	T-Lymphoblastic Leukemia/Lymphoma	Primary	Suspension	MOLT16	ALL
ACH-000636	T-Lymphoblastic Leukemia/Lymphoma	Primary	Suspension	RPMI8402	ALL
ACH-000372	T-Lymphoblastic Leukemia/Lymphoma	Primary	Suspension	P12ICHIKAWA	ALL
ACH-000206	T-Lymphoblastic Leukemia/Lymphoma		Suspension	C8166	ALL
ACH-000995	T-Lymphoblastic Leukemia/Lymphoma	Primary	Adherent	JURKAT	ALL
ACH-000953	T-Lymphoblastic Leukemia/Lymphoma	Metastatic	Suspension	SUPT1	ALL
ACH-000981	T-Lymphoblastic Leukemia/Lymphoma	Primary	Suspension	DND41	ALL
ACH-001737	Adult T-Cell Leukemia/Lymphoma	Primary	Suspension	CTV1DM	ATLL
ACH-000197	Adult T-Cell Leukemia/Lymphoma	Primary	Suspension	TALL1	ATLL
ACH-000964	Adult T-Cell Leukemia/Lymphoma	Primary	Suspension	MOLT3	ATLL
ACH-000101	Adult T-Cell Leukemia/Lymphoma	Primary	Suspension	KE37	ATLL
ACH-001097	Adult T-Cell Leukemia/Lymphoma		Suspension	KARPAS384	ATLL
ACH-000122	Adult T-Cell Leukemia/Lymphoma		Suspension	SUPT11	ATLL
ACH-000105	Adult T-Cell Leukemia/Lymphoma	Primary	Suspension	ALLSIL	ATLL
ACH-001289	Atypical Teratoid/Rhabdoid Tumor		Suspension	COGAR359	ATRT
ACH-001020	Atypical Teratoid/Rhabdoid Tumor		Adherent	BT16	ATRT
ACH-000160	Atypical Teratoid/Rhabdoid Tumor	Primary	Adherent	BT12	ATRT
ACH-001028	Atypical Teratoid/Rhabdoid Tumor		Mixed	CHLA06ATRT	ATRT
ACH-001031	Atypical Teratoid/Rhabdoid Tumor	Primary	Adherent	CHLA266	ATRT
ACH-001492	Burkitt Lymphoma	Primary	Suspension	DOGKIT	BL
ACH-001447	Burkitt Lymphoma	Primary	Adherent	BJAB	BL
ACH-001449	Burkitt Lymphoma	Primary	Suspension	BL2	BL
ACH-000877	Burkitt Lymphoma	Primary	Suspension	EB1	BL
ACH-001493	Burkitt Lymphoma	Primary	Suspension	DOGUM	BL
ACH-000944	Burkitt Lymphoma		Suspension	NAMALWA	BL
ACH-000440	Burkitt Lymphoma	Primary	Suspension	CA46	BL
ACH-001450	Burkitt Lymphoma	Primary	Suspension	BLUE1	BL

ACH-000707	Burkitt Lymphoma	Primary	Suspension	P3HR1	BL
ACH-001503	Burkitt Lymphoma	Primary	Suspension	GUMBUS	BL
ACH-000786	Burkitt Lymphoma	Primary	Suspension	DAUDI	BL
ACH-000654	Burkitt Lymphoma	Primary	Suspension	RAJI	BL
ACH-002055	Burkitt Lymphoma	Primary	Suspension	TL1	BL
ACH-000162	Burkitt Lymphoma		Suspension	GA10	BL
ACH-001636	Burkitt Lymphoma	Primary	Suspension	RAMOS	BL
ACH-000245	Burkitt Lymphoma		Suspension	BL41	BL
ACH-001064	Burkitt Lymphoma		Suspension	EB2	BL
ACH-000567	Burkitt Lymphoma	Metastatic	Suspension	ST486	BL
ACH-000402	Burkitt Lymphoma		Suspension	BL70	BL
ACH-001411	Bladder Squamous Cell Carcinoma	Primary	Adherent	UMUC5	BLCA
ACH-000839	Bladder Squamous Cell Carcinoma	Primary	Adherent	SCABER	BLCA
ACH-000242	Bladder Urothelial Carcinoma	Primary	Adherent	RT4	BLCA
ACH-001409	Bladder Urothelial Carcinoma	Primary	Adherent	UMUC16	BLCA
ACH-001414	Bladder Urothelial Carcinoma	Primary	Adherent	UMUC6	BLCA
ACH-000566	Bladder Urothelial Carcinoma	Primary	Adherent	SW1710	BLCA
ACH-001412	Bladder Urothelial Carcinoma	Primary	Adherent	UMUC10	BLCA
ACH-001407	Bladder Urothelial Carcinoma	Metastatic	Adherent	UMUC13	BLCA
ACH-000896	Bladder Urothelial Carcinoma	Primary	Adherent	647V	BLCA
ACH-001413	Bladder Urothelial Carcinoma	Primary	Adherent	UMUC11	BLCA
ACH-000545	Bladder Urothelial Carcinoma	Primary	Adherent	VMCUB1	BLCA
ACH-001415	Bladder Urothelial Carcinoma	Primary	Adherent	UMUC7	BLCA
ACH-000834	Bladder Urothelial Carcinoma	Metastatic	Adherent	UMUC1	BLCA
ACH-001183	Bladder Urothelial Carcinoma	Primary	Adherent	RT11284	BLCA
ACH-000486	Bladder Urothelial Carcinoma	Primary	Adherent	KU1919	BLCA
ACH-000396	Bladder Urothelial Carcinoma	Primary	Adherent	J82	BLCA
ACH-000724	Bladder Urothelial Carcinoma	Primary	Adherent	HT1376	BLCA
ACH-000522	Bladder Urothelial Carcinoma	Primary	Adherent	UMUC3	BLCA
ACH-000753	Bladder Urothelial Carcinoma	Metastatic	Adherent	JMSU1	BLCA
ACH-000547	Bladder Urothelial Carcinoma	Primary	Adherent	HT1197	BLCA
ACH-000018	Bladder Urothelial Carcinoma	Primary	Adherent	T24	BLCA
ACH-000142	Bladder Urothelial Carcinoma	Primary	Adherent	CAL29	BLCA
ACH-000741	Bladder Urothelial Carcinoma	Primary	Adherent	UBLC1	BLCA
ACH-000905	Bladder Urothelial Carcinoma	Primary	Mixed	5637	BLCA
ACH-000026	Bladder Urothelial Carcinoma	Metastatic	Mixed	253JBV	BLCA
ACH-000802	Bladder Urothelial Carcinoma	Primary	Adherent	BFTC905	BLCA
ACH-000011	Bladder Urothelial Carcinoma	Metastatic	Adherent	253J	BLCA
ACH-000473	Bladder Urothelial Carcinoma	Primary	Adherent	RT112	BLCA
ACH-001416	Bladder Urothelial Carcinoma	Primary	Adherent	UMUC9	BLCA
ACH-000862	Bladder Urothelial Carcinoma	Primary	Adherent	KMBC2	BLCA
ACH-000593	Bladder Urothelial Carcinoma	Primary	Adherent	BC3C	BLCA
ACH-001410	Bladder Urothelial Carcinoma	Metastatic	Adherent	UMUC4	BLCA
ACH-000720	Bladder Urothelial Carcinoma	Primary	Adherent	TCCSUP	BLCA
ACH-000384	Bladder Urothelial Carcinoma	Primary	Adherent	SW780	BLCA
ACH-000127	Bladder Urothelial Carcinoma	Primary	Adherent	SLR20	BLCA
ACH-000725	Breast Ductal Carcinoma In Situ	Primary	Adherent	HCC202	BRCA
ACH-000755	Breast Ductal Carcinoma In Situ	Primary	Suspension	HCC2218	BRCA
ACH-000859	Breast Ductal Carcinoma In Situ	Primary	Adherent	HCC1954	BRCA
ACH-000349	Breast Ductal Carcinoma In Situ	Primary	Adherent	HCC1500	BRCA
ACH-000624	Breast Ductal Carcinoma In Situ	Primary	Adherent	HCC1806	BRCA
ACH-001662	Breast Invasive Carcinoma, NOS	Primary	Adherent	SNU2372	BRCA
ACH-000148	Breast Invasive Carcinoma, NOS	Primary	Adherent	HS578T	BRCA
ACH-000573	Breast Invasive Carcinoma, NOS	Metastatic	Mixed	MDAMB436	BRCA
ACH-000288	Breast Invasive Carcinoma, NOS	Primary	Adherent	BT549	BRCA
ACH-000276	Breast Invasive Ductal Carcinoma	Primary	Adherent	HCC38	BRCA
ACH-000818	Breast Invasive Ductal Carcinoma	Primary	Adherent	BT483	BRCA
ACH-000223	Breast Invasive Ductal Carcinoma	Primary	Adherent	HCC1937	BRCA
ACH-000330	Breast Invasive Ductal Carcinoma	Metastatic	Adherent	EFM19	BRCA
ACH-000643	Breast Invasive Ductal Carcinoma	Primary	Adherent	HDQP1	BRCA
ACH-000711	Breast Invasive Ductal Carcinoma	Metastatic	Adherent	JIMT1	BRCA
ACH-000857	Breast Invasive Ductal Carcinoma	Primary	Adherent	CAL851	BRCA
ACH-002499	Breast Invasive Ductal Carcinoma	Metastatic	Adherent	UACC3199	BRCA
ACH-000196	Breast Invasive Ductal Carcinoma	Primary	Suspension	HCC1599	BRCA
ACH-000691	Breast Invasive Ductal Carcinoma	Primary	Suspension	HCC2157	BRCA
ACH-000374	Breast Invasive Ductal Carcinoma	Primary	Adherent	HCC1143	BRCA
ACH-001705	Breast Invasive Ductal Carcinoma	Primary	Adherent	VP267	BRCA
ACH-000668	Breast Invasive Ductal Carcinoma	Primary	Adherent	HCC70	BRCA
ACH-001419	Breast Invasive Ductal Carcinoma	Primary	Adherent	VP229	BRCA
ACH-001389	Breast Invasive Ductal Carcinoma	Metastatic	Adherent	SUM1315MO2	BRCA
ACH-000111	Breast Invasive Ductal Carcinoma	Primary	Mixed	HCC1187	BRCA
ACH-000621	Breast Invasive Ductal Carcinoma	Metastatic	Adherent	MDAMB157	BRCA
ACH-000277	Breast Invasive Ductal Carcinoma	Primary	Adherent	HCC1419	BRCA
ACH-000721	Breast Invasive Ductal Carcinoma	Metastatic	Adherent	HMC18	BRCA

ACH-000902	Breast Invasive Ductal Carcinoma	Metastatic	Mixed	CAL148	BRCA
ACH-000927	Breast Invasive Ductal Carcinoma	Primary	Adherent	BT474	BRCA
ACH-000759	Breast Invasive Ductal Carcinoma	Metastatic	Adherent	MDAMB175VII	BRCA
ACH-000147	Breast Invasive Ductal Carcinoma	Metastatic	Adherent	T47D	BRCA
ACH-000930	Breast Invasive Ductal Carcinoma	Primary	Adherent	HCC1569	BRCA
ACH-000699	Breast Invasive Ductal Carcinoma	Primary	Adherent	HCC1395	BRCA
ACH-001390	Breast Invasive Ductal Carcinoma	Primary	Adherent	SUM149PT	BRCA
ACH-001388	Breast Invasive Ductal Carcinoma	Primary	Suspension	SUM102PT	BRCA
ACH-000536	Breast Invasive Ductal Carcinoma	Primary	Adherent	BT20	BRCA
ACH-001819	Breast Invasive Ductal Carcinoma	Metastatic	Adherent	MFM223	BRCA
ACH-002401	Breast Invasive Ductal Carcinoma	Metastatic	Adherent	21MT2	BRCA
ACH-000097	Breast Invasive Ductal Carcinoma	Metastatic	Adherent	ZR751	BRCA
ACH-002399	Breast Invasive Ductal Carcinoma	Primary	Adherent	21NT	BRCA
ACH-000828	Breast Invasive Lobular Carcinoma	Metastatic	Adherent	ZR7530	BRCA
ACH-000554	Breast Invasive Lobular Carcinoma	Primary	Adherent	UACC893	BRCA
ACH-002921	Breast Invasive Lobular Carcinoma	Metastatic	Adherent	BCK4	BRCA
ACH-001683	Breast Invasive Lobular Carcinoma	Primary	Adherent	UACC3133	BRCA
ACH-000568	Breast Invasive Lobular Carcinoma	Primary	Mixed	UACC812	BRCA
ACH-000783	Breast Invasive Lobular Carcinoma	Metastatic	Adherent	CAMA1	BRCA
ACH-000044	Breast Invasive Lobular Carcinoma	Metastatic	Adherent	MDAMB134VI	BRCA
ACH-001395	Breast Invasive Lobular Carcinoma	Metastatic	Adherent	SUM44PE	BRCA
ACH-000117	Invasive Breast Carcinoma	Metastatic	Adherent	EFM192A	BRCA
ACH-000017	Invasive Breast Carcinoma	Metastatic	Mixed	SKBR3	BRCA
ACH-000856	Invasive Breast Carcinoma	Metastatic	Adherent	CAL51	BRCA
ACH-000019	Invasive Breast Carcinoma	Metastatic	Adherent	MCF7	BRCA
ACH-000248	Invasive Breast Carcinoma	Metastatic	Adherent	AU565	BRCA
ACH-000258	Invasive Breast Carcinoma	Metastatic	Suspension	DU4475	BRCA
ACH-001392	Invasive Breast Carcinoma	Metastatic	Adherent	SUM185PE	BRCA
ACH-000876	Invasive Breast Carcinoma	Metastatic	Adherent	MDAMB415	BRCA
ACH-001396	Invasive Breast Carcinoma	Metastatic	Adherent	SUM52PE	BRCA
ACH-000028	Invasive Breast Carcinoma	Metastatic	Adherent	KPL1	BRCA
ACH-001394	Invasive Breast Carcinoma	Metastatic	Adherent	SUM229PE	BRCA
ACH-000768	Invasive Breast Carcinoma	Metastatic	Adherent	MDAMB231	BRCA
ACH-001393	Invasive Breast Carcinoma	Primary	Suspension	SUM190PT	BRCA
ACH-000910	Invasive Breast Carcinoma	Metastatic	Adherent	MDAMB453	BRCA
ACH-000352	Invasive Breast Carcinoma	Metastatic	Adherent	HCC1428	BRCA
ACH-000212	Invasive Breast Carcinoma	Metastatic	Adherent	CAL120	BRCA
ACH-000849	Invasive Breast Carcinoma	Metastatic	Adherent	MDAMB468	BRCA
ACH-001391	Invasive Breast Carcinoma	Primary	Adherent	SUM159PT	BRCA
ACH-000934	Invasive Breast Carcinoma	Metastatic	Adherent	MDAMB361	BRCA
ACH-001525	Cervical Adenocarcinoma	Metastatic	Adherent	HT3	CESC
ACH-001650	Cervical Adenocarcinoma	Primary	Adherent	SISO	CESC
ACH-001652	Cervical Squamous Cell Carcinoma	Primary	Adherent	SKGII	CESC
ACH-001651	Cervical Squamous Cell Carcinoma	Primary	Adherent	SKGI	CESC
ACH-001336	Cervical Squamous Cell Carcinoma	Metastatic	Adherent	CASKI	CESC
ACH-001515	Cervical Squamous Cell Carcinoma	Metastatic	Adherent	HCS2	CESC
ACH-001360	Cervical Squamous Cell Carcinoma	Metastatic	Adherent	MS751	CESC
ACH-000556	Cervical Squamous Cell Carcinoma	Primary	Adherent	SIHA	CESC
ACH-000490	Cervical Squamous Cell Carcinoma	Metastatic	Adherent	SF767	CESC
ACH-001334	Cervical Squamous Cell Carcinoma	Primary	Adherent	C4I	CESC
ACH-001451	Cervical Squamous Cell Carcinoma	Primary	Adherent	BOKU	CESC
ACH-001335	Cervical Squamous Cell Carcinoma	Primary	Adherent	C4II	CESC
ACH-001333	Cervical Squamous Cell Carcinoma	Primary	Adherent	C33A	CESC
ACH-001402	Cervical Squamous Cell Carcinoma	Primary	Adherent	SW756	CESC
ACH-002020	Cervical Squamous Cell Carcinoma	Primary	Adherent	SKGIIIA	CESC
ACH-001086	Endocervical Adenocarcinoma	Primary	Adherent	HELA	CESC
ACH-001516	Endocervical Adenocarcinoma	Primary	Adherent	HCSC1	CESC
ACH-001513	Endocervical Adenocarcinoma	Metastatic	Adherent	HCA1	CESC
ACH-002647	Cholangiocarcinoma	Metastatic	Adherent	CCC5	CHOL
ACH-000461	Extrahepatic Cholangiocarcinoma	Primary	Adherent	SNU1196	CHOL
ACH-001536	Extrahepatic Cholangiocarcinoma	Primary	Adherent	KKU100	CHOL
ACH-000268	Extrahepatic Cholangiocarcinoma	Primary	Adherent	SNU245	CHOL
ACH-001673	Extrahepatic Cholangiocarcinoma	Primary	Adherent	TFK1	CHOL
ACH-001494	Extrahepatic Cholangiocarcinoma	Primary	Adherent	EGI1	CHOL
ACH-001997	Extrahepatic Cholangiocarcinoma	Primary	Adherent	ECC2	CHOL
ACH-001607	Gallbladder Adenocarcinoma, NOS	Metastatic	Adherent	NOZ	CHOL
ACH-001619	Gallbladder Adenocarcinoma, NOS	Metastatic	Adherent	OCUG1	CHOL
ACH-001850	Gallbladder Adenocarcinoma, NOS	Metastatic	Adherent	G415	CHOL
ACH-000141	Gallbladder Adenocarcinoma, NOS	Primary	Adherent	SNU308	CHOL
ACH-001961	Gallbladder Adenocarcinoma, NOS	Metastatic	Adherent	GB2	CHOL
ACH-001861	Gallbladder Cancer	Metastatic	Adherent	TGBC1TKB	CHOL
ACH-001538	Intrahepatic Cholangiocarcinoma	Primary	Adherent	KKU213	CHOL
ACH-001864	Intrahepatic Cholangiocarcinoma	Metastatic	Adherent	YSCCC	CHOL
ACH-001838	Intrahepatic Cholangiocarcinoma	Primary	Adherent	ICC12	CHOL

ACH-001846	Intrahepatic Cholangiocarcinoma	Primary	Adherent	ICC6	CHOL
ACH-001841	Intrahepatic Cholangiocarcinoma	Primary	Adherent	ICC15	CHOL
ACH-001959	Intrahepatic Cholangiocarcinoma	Primary	Adherent	CCLP1	CHOL
ACH-001537	Intrahepatic Cholangiocarcinoma	Primary	Adherent	KKU055	CHOL
ACH-000209	Intrahepatic Cholangiocarcinoma	Primary	Adherent	SNU1079	CHOL
ACH-000808	Intrahepatic Cholangiocarcinoma	Primary	Adherent	HUH28	CHOL
ACH-001848	Intrahepatic Cholangiocarcinoma	Primary	Adherent	ICC8	CHOL
ACH-001855	Intrahepatic Cholangiocarcinoma	Primary	Adherent	OZ	CHOL
ACH-001839	Intrahepatic Cholangiocarcinoma	Primary	Adherent	ICC137	CHOL
ACH-001852	Intrahepatic Cholangiocarcinoma	Primary	Adherent	HKGZCC	CHOL
ACH-001863	Intrahepatic Cholangiocarcinoma	Primary	Adherent	TKKK	CHOL
ACH-001996	Intrahepatic Cholangiocarcinoma	Primary	Adherent	ICC18	CHOL
ACH-001836	Intrahepatic Cholangiocarcinoma	Primary	Adherent	ICC108	CHOL
ACH-000976	Intrahepatic Cholangiocarcinoma	Metastatic	Adherent	HUCCT1	CHOL
ACH-001994	Intrahepatic Cholangiocarcinoma		Adherent	ICC16	CHOL
ACH-001843	Intrahepatic Cholangiocarcinoma	Primary	Adherent	ICC3	CHOL
ACH-001847	Intrahepatic Cholangiocarcinoma	Primary	Adherent	ICC7	CHOL
ACH-001842	Intrahepatic Cholangiocarcinoma	Primary	Adherent	ICC2	CHOL
ACH-001960	Intrahepatic Cholangiocarcinoma	Primary	Adherent	CCSW1	CHOL
ACH-001856	Intrahepatic Cholangiocarcinoma	Primary	Adherent	RBE	CHOL
ACH-001857	Intrahepatic Cholangiocarcinoma	Primary	Adherent	SG231	CHOL
ACH-001835	Intrahepatic Cholangiocarcinoma	Primary	Adherent	ICC106	CHOL
ACH-001834	Intrahepatic Cholangiocarcinoma	Primary	Adherent	ICC10	CHOL
ACH-001849	Intrahepatic Cholangiocarcinoma	Primary	Adherent	ICC9	CHOL
ACH-001845	Intrahepatic Cholangiocarcinoma	Primary	Adherent	ICC5	CHOL
ACH-001844	Intrahepatic Cholangiocarcinoma	Primary	Adherent	ICC4	CHOL
ACH-001858	Intrahepatic Cholangiocarcinoma	Primary	Adherent	SSP25	CHOL
ACH-001150	Chondrosarcoma	Primary	Adherent	OUMS27	CHS
ACH-000418	Chondrosarcoma	Primary	Adherent	SW1353	CHS
ACH-002781	Dedifferentiated Chondrosarcoma	Primary	Adherent	NCCDCS1C1	CHS
ACH-000516	Dedifferentiated Chondrosarcoma		Adherent	CAL78	CHS
ACH-000999	Colon Adenocarcinoma	Primary	Adherent	SNU1040	COAD
ACH-000552	Colon Adenocarcinoma	Primary	Adherent	HT29	COAD
ACH-000501	Colon Adenocarcinoma	Primary	Adherent	LS123	COAD
ACH-000798	Colon Adenocarcinoma	Primary	Adherent	CL40	COAD
ACH-000998	Colon Adenocarcinoma	Primary	Adherent	CW2	COAD
ACH-000971	Colon Adenocarcinoma	Primary	Adherent	HCT116	COAD
ACH-001454	Colon Adenocarcinoma	Primary	Adherent	C10	COAD
ACH-000926	Colon Adenocarcinoma	Primary	Adherent	HT55	COAD
ACH-000991	Colon Adenocarcinoma	Primary	Adherent	SNU81	COAD
ACH-000009	Colon Adenocarcinoma	Primary	Adherent	C2BBE1	COAD
ACH-000491	Colon Adenocarcinoma	Metastatic	Mixed	NCIH716	COAD
ACH-000342	Colon Adenocarcinoma	Primary	Adherent	CL14	COAD
ACH-000360	Colon Adenocarcinoma	Metastatic	Mixed	NCIH508	COAD
ACH-000249	Colon Adenocarcinoma	Primary	Adherent	CL11	COAD
ACH-000943	Colon Adenocarcinoma	Primary	Adherent	RKO	COAD
ACH-000722	Colon Adenocarcinoma	Metastatic	Mixed	SNUC1	COAD
ACH-000842	Colon Adenocarcinoma	Primary	Adherent	SW480	COAD
ACH-000997	Colon Adenocarcinoma	Primary	Adherent	HCT15	COAD
ACH-000467	Colon Adenocarcinoma	Primary	Adherent	HCC56	COAD
ACH-002539	Colon Adenocarcinoma	Primary	Adherent	SNU1460	COAD
ACH-000710	Colon Adenocarcinoma	Primary	Suspension	NCIH854	COAD
ACH-000403	Colon Adenocarcinoma	Metastatic	Adherent	NCIH747	COAD
ACH-000400	Colon Adenocarcinoma	Metastatic	Adherent	SKCO1	COAD
ACH-000381	Colon Adenocarcinoma	Metastatic	Adherent	T84	COAD
ACH-000296	Colon Adenocarcinoma	Metastatic	Adherent	OUMS23	COAD
ACH-000955	Colon Adenocarcinoma	Metastatic	Adherent	SNU407	COAD
ACH-000895	Colon Adenocarcinoma	Primary	Adherent	CL34	COAD
ACH-000680	Colon Adenocarcinoma	Primary	Mixed	SW948	COAD
ACH-000089	Colon Adenocarcinoma	Metastatic	Adherent	NCIH684	COAD
ACH-000007	Colon Adenocarcinoma	Primary	Adherent	LS513	COAD
ACH-000252	Colon Adenocarcinoma	Primary	Adherent	LS1034	COAD
ACH-002660	Colon Adenocarcinoma	Metastatic	Adherent	JVE187	COAD
ACH-000003	Colon Adenocarcinoma	Primary	Adherent	CACO2	COAD
ACH-000950	Colon Adenocarcinoma	Metastatic	Adherent	LOVO	COAD
ACH-000820	Colon Adenocarcinoma	Primary	Adherent	SW403	COAD
ACH-002538	Colon Adenocarcinoma		Adherent	SNU1235	COAD
ACH-000957	Colon Adenocarcinoma	Primary	Adherent	LS180	COAD
ACH-000935	Colon Adenocarcinoma	Primary	Adherent	MDST8	COAD
ACH-001460	Colon Adenocarcinoma	Primary	Mixed	C84	COAD
ACH-000202	Colon Adenocarcinoma	Primary	Adherent	COLO320	COAD
ACH-000963	Colon Adenocarcinoma	Metastatic	Adherent	CCK81	COAD
ACH-002886	Colon Adenocarcinoma		Adherent	VACO432	COAD
ACH-000969	Colon Adenocarcinoma	Primary	Mixed	KM12	COAD

ACH-002654	Colon Adenocarcinoma	Primary	Adherent	JVE015	COAD
ACH-002662	Colon Adenocarcinoma	Primary	Adherent	JVE207	COAD
ACH-000982	Colon Adenocarcinoma	Primary	Adherent	GP2D	COAD
ACH-000651	Colon Adenocarcinoma	Metastatic	Mixed	SW620	COAD
ACH-000236	Colon Adenocarcinoma	Primary	Suspension	SW1417	COAD
ACH-001345	Colon Adenocarcinoma	Primary	Adherent	GP5D	COAD
ACH-001061	Colon Adenocarcinoma	Primary	Adherent	DLD1	COAD
ACH-000412	Colon Adenocarcinoma	Primary	Adherent	SNU1197	COAD
ACH-001786	Colon Adenocarcinoma	Primary	Adherent	SNU1544	COAD
ACH-000253	Colon Adenocarcinoma	Metastatic	Mixed	COLO201	COAD
ACH-000958	Colon Adenocarcinoma	Primary	Mixed	SW48	COAD
ACH-002667	Colon Adenocarcinoma		Adherent	JVE528	COAD
ACH-000489	Colon Adenocarcinoma	Primary	Adherent	SW1116	COAD
ACH-000986	Colon Adenocarcinoma	Primary	Adherent	HT115	COAD
ACH-000985	Colon Adenocarcinoma	Primary	Adherent	LS411N	COAD
ACH-001399	Colon Adenocarcinoma	Metastatic	Adherent	SW626	COAD
ACH-000350	Colon Adenocarcinoma	Metastatic	Adherent	COLO678	COAD
ACH-000970	Colon Adenocarcinoma	Primary	Adherent	SNUC5	COAD
ACH-002543	Colon Adenocarcinoma	Primary	Suspension	SNU1406	COAD
ACH-001458	Colon Adenocarcinoma	Primary	Adherent	C75	COAD
ACH-000959	Colon Adenocarcinoma	Primary	Adherent	SNUC4	COAD
ACH-000967	Colon Adenocarcinoma	Primary	Adherent	SNUC2A	COAD
ACH-002669	Colon Adenocarcinoma	Primary	Adherent	KP363T	COAD
ACH-001456	Colorectal Adenocarcinoma	Primary	Adherent	C125PM	COAD
ACH-002932	Colorectal Adenocarcinoma	Metastatic	Mixed	CCLFNEURO0001T	COAD
ACH-002678	Diffuse Large B-Cell Lymphoma, NOS		Suspension	WILL1	DLBC
ACH-001616	Diffuse Large B-Cell Lymphoma, NOS	Primary	Suspension	OCILY18	DLBC
ACH-000914	Diffuse Large B-Cell Lymphoma, NOS	Metastatic	Suspension	HT	DLBC
ACH-003100	Diffuse Large B-Cell Lymphoma, NOS	Primary	Suspension	EJ1	DLBC
ACH-001703	Diffuse Large B-Cell Lymphoma, NOS	Metastatic	Suspension	VAL	DLBC
ACH-000124	Diffuse Large B-Cell Lymphoma, NOS	Primary	Suspension	OCILY19	DLBC
ACH-001709	Diffuse Large B-Cell Lymphoma, NOS	Primary	Mixed	WSUNHL	DLBC
ACH-000656	Diffuse Large B-Cell Lymphoma, NOS	Metastatic	Suspension	SUDHL8	DLBC
ACH-003460	Diffuse Large B-Cell Lymphoma, NOS	Primary	Suspension	CCLFHEME0001T	DLBC
ACH-000334	Diffuse Large B-Cell Lymphoma, NOS	Metastatic	Suspension	DB	DLBC
ACH-001617	Diffuse Large B-Cell Lymphoma, NOS	Primary	Suspension	OCILY7	DLBC
ACH-001148	Diffuse Large B-Cell Lymphoma, NOS		Suspension	OCILY132	DLBC
ACH-001686	Diffuse Large B-Cell Lymphoma, NOS	Primary	Suspension	ULA	DLBC
ACH-000371	Diffuse Large B-Cell Lymphoma, NOS	Metastatic	Suspension	RL	DLBC
ACH-000056	Diffuse Large B-Cell Lymphoma, NOS	Metastatic	Suspension	DOHH2	DLBC
ACH-002677	Diffuse Large B-Cell Lymphoma, NOS	Metastatic	Suspension	U2946	DLBC
ACH-001677	Diffuse Large B-Cell Lymphoma, NOS	Primary	Suspension	U2904	DLBC
ACH-000697	Diffuse Large B-Cell Lymphoma, NOS	Primary	Suspension	A3KAW	DLBC
ACH-000611	Diffuse Large B-Cell Lymphoma, NOS	Metastatic	Suspension	SUDHL6	DLBC
ACH-000315	Diffuse Large B-Cell Lymphoma, NOS	Metastatic	Suspension	KARPAS422	DLBC
ACH-001678	Diffuse Large B-Cell Lymphoma, NOS	Primary	Suspension	U2940	DLBC
ACH-000140	Diffuse Large B-Cell Lymphoma, NOS	Metastatic	Suspension	PFEIFFER	DLBC
ACH-000660	Diffuse Large B-Cell Lymphoma, NOS	Primary	Suspension	SUDHL5	DLBC
ACH-000388	Diffuse Large B-Cell Lymphoma, NOS	Metastatic	Suspension	NUDHL1	DLBC
ACH-000365	Diffuse Large B-Cell Lymphoma, NOS	Metastatic	Suspension	SUDHL4	DLBC
ACH-000157	Diffuse Large B-Cell Lymphoma, NOS	Primary	Suspension	A4FUK	DLBC
ACH-000534	Diffuse Large B-Cell Lymphoma, NOS	Metastatic	Suspension	WSUDLCL2	DLBC
ACH-000271	Diffuse Large B-Cell Lymphoma, NOS	Metastatic	Suspension	SUDHL10	DLBC
ACH-000285	Diffuse Large B-Cell Lymphoma, NOS	Primary	Suspension	TOLEDO	DLBC
ACH-000383	Esophageal Adenocarcinoma	Primary	Adherent	OE33	ESCA
ACH-001654	Esophageal Adenocarcinoma	Primary	Adherent	SKGT4	ESCA
ACH-002706	Esophageal Adenocarcinoma	Primary	Mixed	IS076A	ESCA
ACH-001368	Esophageal Adenocarcinoma	Metastatic	Suspension	OACM51	ESCA
ACH-003426	Esophageal Adenocarcinoma	Metastatic	Adherent	CCLFNEURO0046T	ESCA
ACH-002705	Esophageal Adenocarcinoma	Primary	Adherent	IS076P	ESCA
ACH-000216	Esophageal Adenocarcinoma		Adherent	JHESOAD1	ESCA
ACH-001500	Esophageal Adenocarcinoma	Primary	Adherent	FLO1	ESCA
ACH-001497	Esophageal Adenocarcinoma	Primary	Suspension	ESO51	ESCA
ACH-002703	Esophageal Adenocarcinoma	Primary	Adherent	OANC1	ESCA
ACH-000823	Esophageal Squamous Cell Carcinoma	Primary	Adherent	KYSE140	ESCA
ACH-000917	Esophageal Squamous Cell Carcinoma		Adherent	TE4	ESCA
ACH-000544	Esophageal Squamous Cell Carcinoma	Primary	Adherent	OE21	ESCA
ACH-000784	Esophageal Squamous Cell Carcinoma	Primary	Adherent	KYSE70	ESCA
ACH-000561	Esophageal Squamous Cell Carcinoma	Metastatic	Adherent	TDOTT	ESCA
ACH-000726	Esophageal Squamous Cell Carcinoma		Adherent	TE14	ESCA
ACH-000318	Esophageal Squamous Cell Carcinoma	Primary	Adherent	TE10	ESCA
ACH-000865	Esophageal Squamous Cell Carcinoma	Primary	Adherent	KYSE450	ESCA
ACH-000679	Esophageal Squamous Cell Carcinoma	Primary	Adherent	OE19	ESCA
ACH-000824	Esophageal Squamous Cell Carcinoma	Primary	Adherent	KYSE510	ESCA

ACH-000637	Esophageal Squamous Cell Carcinoma	Primary	Adherent	KYSE520	ESCA
ACH-000855	Esophageal Squamous Cell Carcinoma	Primary	Adherent	KYSE150	ESCA
ACH-000605	Esophageal Squamous Cell Carcinoma		Adherent	TE6	ESCA
ACH-000809	Esophageal Squamous Cell Carcinoma	Primary	Adherent	KYSE410	ESCA
ACH-000694	Esophageal Squamous Cell Carcinoma	Metastatic	Adherent	TE9	ESCA
ACH-000305	Esophageal Squamous Cell Carcinoma		Adherent	ECGI10	ESCA
ACH-000717	Esophageal Squamous Cell Carcinoma	Primary	Adherent	COLO680N	ESCA
ACH-000873	Esophageal Squamous Cell Carcinoma	Primary	Adherent	KYSE270	ESCA
ACH-000488	Esophageal Squamous Cell Carcinoma	Primary	Adherent	TE11	ESCA
ACH-000647	Esophageal Squamous Cell Carcinoma	Primary	Adherent	TE1	ESCA
ACH-000693	Esophageal Squamous Cell Carcinoma	Primary	Adherent	KYSE180	ESCA
ACH-000452	Esophageal Squamous Cell Carcinoma		Adherent	TE8	ESCA
ACH-000777	Esophageal Squamous Cell Carcinoma	Primary	Adherent	KYSE30	ESCA
ACH-000353	Esophageal Squamous Cell Carcinoma	Primary	Adherent	TE15	ESCA
ACH-000408	Esophageal Squamous Cell Carcinoma		Adherent	TE5	ESCA
ACH-001034	Ewing Sarcoma	Primary	Adherent	CHLA9	EWS
ACH-001283	Ewing Sarcoma	Metastatic	Adherent	TC106	EWS
ACH-002780	Ewing Sarcoma	Primary	Suspension	NCCES1C1	EWS
ACH-001029	Ewing Sarcoma		Adherent	CHLA10	EWS
ACH-000087	Ewing Sarcoma	Primary	Mixed	SKES1	EWS
ACH-001193	Ewing Sarcoma		Adherent	SKPNDW	EWS
ACH-000391	Ewing Sarcoma	Metastatic	Adherent	MHHES1	EWS
ACH-001430	Ewing Sarcoma	Primary	Mixed	TC138	EWS
ACH-001427	Ewing Sarcoma	Metastatic	Adherent	CCLFPEDS0007T	EWS
ACH-000210	Ewing Sarcoma	Metastatic	Suspension	CADOES1	EWS
ACH-000424	Ewing Sarcoma	Primary	Adherent	TC71	EWS
ACH-001192	Ewing Sarcoma	Metastatic	Suspension	SKNEP1	EWS
ACH-000279	Ewing Sarcoma	Primary	Adherent	EWS502	EWS
ACH-000499	Ewing Sarcoma	Primary	Adherent	EW8	EWS
ACH-000041	Ewing Sarcoma	Primary	Mixed	RDES	EWS
ACH-001022	Ewing Sarcoma	Primary	Unknown	CBAGPN	EWS
ACH-001032	Ewing Sarcoma	Primary	Adherent	CHLA32	EWS
ACH-001038	Ewing Sarcoma		Mixed	COGE352	EWS
ACH-001431	Ewing Sarcoma	Primary	Adherent	TC205	EWS
ACH-001030	Ewing Sarcoma	Primary	Mixed	CHLA218	EWS
ACH-000039	Ewing Sarcoma	Metastatic	Adherent	SKNMC	EWS
ACH-001035	Ewing Sarcoma	Primary	Suspension	CHLA99	EWS
ACH-001428	Ewing Sarcoma	Metastatic	Adherent	CCLFPEDS0010T	EWS
ACH-000052	Ewing Sarcoma	Primary	Adherent	A673	EWS
ACH-000570	Glioblastoma	Primary	Adherent	YKG1	GBM
ACH-000075	Glioblastoma	Primary	Adherent	U87MG	GBM
ACH-000269	Glioblastoma	Primary	Adherent	AM38	GBM
ACH-000376	Glioblastoma	Primary	Adherent	SF295	GBM
ACH-000887	Glioblastoma	Primary	Adherent	SF172	GBM
ACH-000368	Glioblastoma	Primary	Adherent	SNU1105	GBM
ACH-001611	Glioblastoma	Primary	Adherent	NP8	GBM
ACH-000098	Glioblastoma	Primary	Adherent	GAMG	GBM
ACH-000760	Glioblastoma	Primary	Adherent	LNZ308	GBM
ACH-001610	Glioblastoma	Primary	Adherent	NP5	GBM
ACH-002680	Glioblastoma	Primary	Adherent	170MGBA	GBM
ACH-000231	Glioblastoma	Primary	Adherent	KALS1	GBM
ACH-003438	Glioblastoma	Primary	Mixed	CCLFNEURO0005T	GBM
ACH-000819	Glioblastoma	Primary	Adherent	LN18	GBM
ACH-000571	Glioblastoma	Primary	Adherent	T98G	GBM
ACH-000027	Glioblastoma	Primary	Adherent	GOS3	GBM
ACH-000370	Glioblastoma	Primary	Adherent	SNU626	GBM
ACH-000328	Glioblastoma	Primary	Adherent	LN215	GBM
ACH-001623	Glioblastoma	Primary	Adherent	ONDA8	GBM
ACH-000543	Glioblastoma	Primary	Adherent	SNU489	GBM
ACH-000215	Glioblastoma	Primary	Adherent	LN382	GBM
ACH-000479	Glioblastoma	Primary	Adherent	KNS81	GBM
ACH-000455	Glioblastoma	Primary	Adherent	LN428	GBM
ACH-000623	Glioblastoma	Primary	Adherent	SNU201	GBM
ACH-000609	Glioblastoma	Primary	Adherent	SF126	GBM
ACH-000504	Glioblastoma	Primary	Adherent	SNB75	GBM
ACH-001605	Glioblastoma	Primary	Adherent	NO10	GBM
ACH-000673	Glioblastoma	Primary	Adherent	LN443	GBM
ACH-000323	Glioblastoma	Primary	Adherent	42MGBA	GBM
ACH-000738	Glioblastoma	Primary	Adherent	GB1	GBM
ACH-002681	Glioblastoma		Adherent	538MGBA	GBM
ACH-000102	Glioblastoma	Primary	Adherent	GMS10	GBM
ACH-000634	Glioblastoma	Primary	Adherent	LN340	GBM
ACH-001609	Glioblastoma	Primary	Adherent	NP3	GBM
ACH-000137	Glioblastoma	Primary	Adherent	8MGBA	GBM

ACH-000445	Glioblastoma	Primary	Adherent	KNS60	GBM
ACH-001329	Glioblastoma	Primary	Adherent	ANGMCSS	GBM
ACH-000289	Glioblastoma	Primary	Adherent	SNU466	GBM
ACH-001624	Glioblastoma	Primary	Adherent	ONDA9	GBM
ACH-000244	Glioblastoma	Primary	Adherent	DKMG	GBM
ACH-001608	Glioblastoma	Primary	Adherent	NP2	GBM
ACH-000464	Glioblastoma	Primary	Adherent	CAS1	GBM
ACH-000622	Glioblastoma	Primary	Adherent	KNS42	GBM
ACH-000558	Glioblastoma	Primary	Adherent	A172	GBM
ACH-000631	Glioblastoma	Primary	Adherent	KS1	GBM
ACH-000863	Glioblastoma	Primary	Adherent	DBTRG05MG	GBM
ACH-000283	Glioblastoma	Primary	Adherent	A1207	GBM
ACH-000152	Glioblastoma	Primary	Adherent	M059K	GBM
ACH-000595	Glioblastoma	Primary	Adherent	LN229	GBM
ACH-000676	Glioblastoma	Primary	Adherent	LN464	GBM
ACH-000469	Glioblastoma	Primary	Adherent	YH13	GBM
ACH-002409	Glioblastoma	Primary	Adherent	CCLFNEURO0006T	GBM
ACH-001622	Glioblastoma	Primary	Adherent	ONDA7	GBM
ACH-000200	Glioblastoma	Primary	Adherent	NMCG1	GBM
ACH-000036	Glioblastoma	Primary	Adherent	U343	GBM
ACH-000754	Hodgkin Lymphoma		Suspension	L428	HL
ACH-000702	Hodgkin Lymphoma		Suspension	L1236	HL
ACH-000815	Hodgkin Lymphoma	Metastatic	Suspension	KMH2	HL
ACH-000806	Hodgkin Lymphoma		Suspension	L540	HL
ACH-000069	Hodgkin Lymphoma		Suspension	HS611T	HL
ACH-000267	Hodgkin Lymphoma		Suspension	HDLM2	HL
ACH-001685	Hodgkin Lymphoma	Primary	Suspension	UHO1	HL
ACH-002711	Adenoid Cystic Carcinoma	Primary	Adherent	UMHACC2A	HNSC
ACH-000163	Head and Neck Squamous Cell Carcinoma	Primary	Adherent	SW579	HNSC
ACH-002042	Head and Neck Squamous Cell Carcinoma	Primary	Adherent	T3M5	HNSC
ACH-001641	Head and Neck Squamous Cell Carcinoma	Primary	Adherent	SAT	HNSC
ACH-000762	Head and Neck Squamous Cell Carcinoma		Adherent	YD38	HNSC
ACH-003475	Head and Neck Squamous Cell Carcinoma	Metastatic	Adherent	CCLFHNSC0003T	HNSC
ACH-003474	Head and Neck Squamous Cell Carcinoma	Primary	Adherent	CCLFHNSC0001T	HNSC
ACH-003465	Head and Neck Squamous Cell Carcinoma	Primary	Adherent	CCLFHNSC0004T	HNSC
ACH-003476	Head and Neck Squamous Cell Carcinoma	Primary	Adherent	CCLFHNSC0002T	HNSC
ACH-000415	Hypopharynx Squamous Cell Carcinoma	Primary	Adherent	BICR6	HNSC
ACH-000618	Hypopharynx Squamous Cell Carcinoma		Adherent	SNU1041	HNSC
ACH-000846	Hypopharynx Squamous Cell Carcinoma	Primary	Adherent	FADU	HNSC
ACH-000715	Larynx Squamous Cell Carcinoma		Adherent	SNU1214	HNSC
ACH-000692	Larynx Squamous Cell Carcinoma	Primary	Adherent	SNU899	HNSC
ACH-000682	Larynx Squamous Cell Carcinoma		Adherent	SNU1066	HNSC
ACH-000500	Larynx Squamous Cell Carcinoma		Adherent	SNU46	HNSC
ACH-000549	Larynx Squamous Cell Carcinoma		Adherent	SNU1076	HNSC
ACH-000606	Oral Cavity Squamous Cell Carcinoma	Primary	Adherent	PECAPJ34CLONEC12	HNSC
ACH-000723	Oral Cavity Squamous Cell Carcinoma		Adherent	YD10B	HNSC
ACH-002029	Oral Cavity Squamous Cell Carcinoma	Primary	Adherent	SAS	HNSC
ACH-001332	Oral Cavity Squamous Cell Carcinoma	Primary	Adherent	BICR78	HNSC
ACH-001227	Oral Cavity Squamous Cell Carcinoma	Primary	Adherent	UPCISCC090	HNSC
ACH-000832	Oral Cavity Squamous Cell Carcinoma	Primary	Adherent	CAL27	HNSC
ACH-001691	Oral Cavity Squamous Cell Carcinoma	Primary	Adherent	UPCISCC029A	HNSC
ACH-000732	Oral Cavity Squamous Cell Carcinoma	Primary	Adherent	PECAPJ41CLONED2	HNSC
ACH-001695	Oral Cavity Squamous Cell Carcinoma	Primary	Adherent	UPCISCC099	HNSC
ACH-001696	Oral Cavity Squamous Cell Carcinoma	Primary	Adherent	UPCISCC111	HNSC
ACH-001701	Oral Cavity Squamous Cell Carcinoma	Primary	Adherent	UPCISCC200	HNSC
ACH-001626	Oral Cavity Squamous Cell Carcinoma	Metastatic	Adherent	OSC20	HNSC
ACH-000619	Oral Cavity Squamous Cell Carcinoma	Primary	Adherent	PECAPJ15	HNSC
ACH-000546	Oral Cavity Squamous Cell Carcinoma	Metastatic	Adherent	HSC4	HNSC
ACH-001690	Oral Cavity Squamous Cell Carcinoma	Primary	Adherent	UPCISCC026	HNSC
ACH-001347	Oral Cavity Squamous Cell Carcinoma	Primary	Adherent	H157	HNSC
ACH-000836	Oral Cavity Squamous Cell Carcinoma		Adherent	YD15	HNSC
ACH-001692	Oral Cavity Squamous Cell Carcinoma	Primary	Adherent	UPCISCC040	HNSC
ACH-000503	Oral Cavity Squamous Cell Carcinoma	Primary	Adherent	BICR16	HNSC
ACH-002043	Oral Cavity Squamous Cell Carcinoma	Primary	Adherent	CA922	HNSC
ACH-000188	Oral Cavity Squamous Cell Carcinoma	Primary	Adherent	SCC25	HNSC
ACH-001625	Oral Cavity Squamous Cell Carcinoma	Metastatic	Adherent	OSC19	HNSC
ACH-000238	Oral Cavity Squamous Cell Carcinoma	Primary	Adherent	SCC4	HNSC
ACH-003099	Oral Cavity Squamous Cell Carcinoma		Adherent	UCSFOT1109	HNSC
ACH-000472	Oral Cavity Squamous Cell Carcinoma	Metastatic	Adherent	HSC2	HNSC
ACH-000518	Oral Cavity Squamous Cell Carcinoma	Primary	Adherent	CAL33	HNSC
ACH-000548	Oral Cavity Squamous Cell Carcinoma	Primary	Adherent	BHY	HNSC
ACH-000735	Oral Cavity Squamous Cell Carcinoma	Primary	Adherent	PECAPJ49	HNSC
ACH-001509	Oral Cavity Squamous Cell Carcinoma	Primary	Adherent	H357	HNSC
ACH-000254	Oral Cavity Squamous Cell Carcinoma		Adherent	SCC15	HNSC

ACH-001445	Oral Cavity Squamous Cell Carcinoma	Primary	Adherent	BICR3	HNSC
ACH-001694	Oral Cavity Squamous Cell Carcinoma	Primary	Adherent	UPCISCC074	HNSC
ACH-001542	Oral Cavity Squamous Cell Carcinoma	Metastatic	Adherent	KON	HNSC
ACH-001699	Oral Cavity Squamous Cell Carcinoma	Primary	Adherent	UPCISCC131	HNSC
ACH-001229	Oral Cavity Squamous Cell Carcinoma	Primary	Adherent	UPCISCC154	HNSC
ACH-000630	Oral Cavity Squamous Cell Carcinoma		Adherent	YD8	HNSC
ACH-000740	Oral Cavity Squamous Cell Carcinoma	Primary	Adherent	A253	HNSC
ACH-002045	Oral Cavity Squamous Cell Carcinoma	Primary	Adherent	HO1U1	HNSC
ACH-001693	Oral Cavity Squamous Cell Carcinoma	Primary	Adherent	UPCISCC072	HNSC
ACH-001697	Oral Cavity Squamous Cell Carcinoma	Primary	Adherent	UPCISCC114	HNSC
ACH-001508	Oral Cavity Squamous Cell Carcinoma	Primary	Adherent	H314	HNSC
ACH-000778	Oral Cavity Squamous Cell Carcinoma	Metastatic	Adherent	HSC3	HNSC
ACH-000228	Oral Cavity Squamous Cell Carcinoma	Primary	Adherent	BICR31	HNSC
ACH-001511	Oral Cavity Squamous Cell Carcinoma	Primary	Adherent	H413	HNSC
ACH-000771	Oral Cavity Squamous Cell Carcinoma	Primary	Adherent	BICR56	HNSC
ACH-001543	Oral Cavity Squamous Cell Carcinoma	Primary	Adherent	KOSC2	HNSC
ACH-000794	Oral Cavity Squamous Cell Carcinoma	Metastatic	Adherent	BICR22	HNSC
ACH-001698	Oral Cavity Squamous Cell Carcinoma	Primary	Adherent	UPCISCC116	HNSC
ACH-001510	Oral Cavity Squamous Cell Carcinoma	Primary	Adherent	H376	HNSC
ACH-001346	Oral Cavity Squamous Cell Carcinoma	Primary	Adherent	H103	HNSC
ACH-001228	Oral Cavity Squamous Cell Carcinoma		Adherent	UPCISCC152	HNSC
ACH-000181	Oral Cavity Squamous Cell Carcinoma	Primary	Adherent	SCC9	HNSC
ACH-000207	Oropharynx Squamous Cell Carcinoma	Metastatic	Adherent	DETROIT562	HNSC
ACH-001700	Oropharynx Squamous Cell Carcinoma	Primary	Adherent	UPCISCC172	HNSC
ACH-002044	Sinonasal Squamous Cell Carcinoma	Primary	Adherent	HSQ89	HNSC
ACH-000792	Renal Cell Carcinoma	Primary	Adherent	BFTC909	KIRC
ACH-000317	Renal Cell Carcinoma	Primary	Adherent	TUHR14TKB	KIRC
ACH-001398	Renal Cell Carcinoma	Primary	Adherent	SW156	KIRC
ACH-001688	Renal Cell Carcinoma	Primary	Adherent	UMRC7	KIRC
ACH-000428	Renal Cell Carcinoma	Primary	Adherent	UO31	KIRC
ACH-000429	Renal Cell Carcinoma	Primary	Adherent	A704	KIRC
ACH-002533	Renal Cell Carcinoma	Primary	Adherent	SNU482	KIRC
ACH-000484	Renal Cell Carcinoma	Primary	Adherent	VMRCRCW	KIRC
ACH-000375	Renal Cell Carcinoma	Primary	Adherent	G402	KIRC
ACH-001194	Renal Cell Carcinoma	Metastatic	Adherent	SKRC31	KIRC
ACH-000171	Renal Cell Carcinoma	Primary	Adherent	VMRCRCZ	KIRC
ACH-000189	Renal Cell Carcinoma	Primary	Adherent	RCC10RGB	KIRC
ACH-000457	Renal Cell Carcinoma	Metastatic	Adherent	CAL54	KIRC
ACH-000385	Renal Cell Carcinoma	Primary	Adherent	SKRC52	KIRC
ACH-000495	Renal Cell Carcinoma	Primary	Adherent	TUHR4TKB	KIRC
ACH-000555	Renal Cell Carcinoma	Primary	Adherent	A498	KIRC
ACH-000313	Renal Clear Cell Carcinoma	Primary	Adherent	KMRC3	KIRC
ACH-000684	Renal Clear Cell Carcinoma	Primary	Adherent	KMRC1	KIRC
ACH-000600	Renal Clear Cell Carcinoma	Primary	Adherent	SLR26	KIRC
ACH-000459	Renal Clear Cell Carcinoma	Primary	Adherent	TUHR10TKB	KIRC
ACH-000262	Renal Clear Cell Carcinoma	Primary	Adherent	UOK101	KIRC
ACH-000300	Renal Clear Cell Carcinoma	Metastatic	Adherent	SLR25	KIRC
ACH-000250	Renal Clear Cell Carcinoma	Primary	Adherent	KMRC20	KIRC
ACH-000649	Renal Clear Cell Carcinoma	Primary	Adherent	786O	KIRC
ACH-000016	Renal Clear Cell Carcinoma	Metastatic	Adherent	SLR21	KIRC
ACH-000411	Renal Clear Cell Carcinoma	Primary	Adherent	769P	KIRC
ACH-000433	Renal Clear Cell Carcinoma	Metastatic	Adherent	CAKI1	KIRC
ACH-002528	Renal Clear Cell Carcinoma	Primary	Adherent	SNU267	KIRC
ACH-000159	Renal Clear Cell Carcinoma	Primary	Adherent	OSRC2	KIRC
ACH-000907	Renal Clear Cell Carcinoma	Primary	Adherent	SNU349	KIRC
ACH-000709	Renal Clear Cell Carcinoma	Primary	Adherent	KMRC2	KIRC
ACH-000272	Renal Clear Cell Carcinoma	Primary	Adherent	SLR24	KIRC
ACH-000513	Renal Clear Cell Carcinoma	Primary	Adherent	SNU1272	KIRC
ACH-001687	Renal Clear Cell Carcinoma	Primary	Adherent	UMRC3	KIRC
ACH-000234	Papillary Renal Cell Carcinoma	Primary	Adherent	CAKI2	KIRP
ACH-000046	Papillary Renal Cell Carcinoma	Metastatic	Adherent	ACHN	KIRP
ACH-003273	Acute Megakaryoblastic Leukemia	Primary	Suspension	CHRF28811	LAML
ACH-000195	Acute Megakaryoblastic Leukemia		Suspension	SET2	LAML
ACH-000602	Acute Megakaryoblastic Leukemia		Suspension	M07E	LAML
ACH-000006	Acute Monoblastic/Monocytic Leukemia	Primary	Suspension	MONOMAC6	LAML
ACH-000112	Acute Monoblastic/Monocytic Leukemia		Suspension	SIGM5	LAML
ACH-001129	Acute Monoblastic/Monocytic Leukemia	Primary	Suspension	MONOMAC1	LAML
ACH-001647	Acute Monoblastic/Monocytic Leukemia	Metastatic	Suspension	SHI1	LAML
ACH-000080	Acute Monoblastic/Monocytic Leukemia		Mixed	BDCM	LAML
ACH-000168	Acute Monoblastic/Monocytic Leukemia	Primary	Suspension	NOMO1	LAML
ACH-000034	Acute Myeloid Leukemia	Primary	Suspension	PLB985	LAML
ACH-000406	Acute Myeloid Leukemia	Metastatic	Suspension	U937	LAML
ACH-001577	Acute Myeloid Leukemia	Primary	Suspension	MUTZ8	LAML
ACH-000002	Acute Myeloid Leukemia	Primary	Suspension	HL60	LAML

ACH-000751	Acute Myeloid Leukemia	Primary	Suspension	OCIM1	LAML
ACH-002675	Acute Myeloid Leukemia		Suspension	SH2	LAML
ACH-000166	Acute Myeloid Leukemia	Primary	Suspension	KASUMI6	LAML
ACH-002994	Acute Myeloid Leukemia		Suspension	WSUAML	LAML
ACH-000770	Acute Myeloid Leukemia		Suspension	P31FUJ	LAML
ACH-000498	Acute Myeloid Leukemia		Suspension	KO52	LAML
ACH-001618	Acute Myeloid Leukemia	Primary	Suspension	OCIM2	LAML
ACH-000218	Acute Myeloid Leukemia	Primary	Suspension	PL21	LAML
ACH-000439	Acute Myeloid Leukemia	Primary	Suspension	ME1	LAML
ACH-000263	Acute Myeloid Leukemia	Primary	Suspension	KASUMI1	LAML
ACH-000336	Acute Myeloid Leukemia	Primary	Suspension	OCIAML3	LAML
ACH-000146	Acute Myeloid Leukemia	Primary	Suspension	THP1	LAML
ACH-002290	Acute Myeloid Leukemia	Primary	Suspension	NKM1	LAML
ACH-000369	Acute Myeloid Leukemia	Primary	Suspension	MOLM16	LAML
ACH-000065	Acute Myeloid Leukemia	Primary	Suspension	OCIAML5	LAML
ACH-000386	Acute Myeloid Leukemia	Primary	Suspension	KG1	LAML
ACH-002946	Acute Myeloid Leukemia		Unknown	UKE1	LAML
ACH-000004	Acute Myeloid Leukemia	Primary	Suspension	HEL	LAML
ACH-000362	Acute Myeloid Leukemia	Primary	Suspension	MOLM13	LAML
ACH-000045	Acute Myeloid Leukemia	Primary	Suspension	MV411	LAML
ACH-000387	Acute Myeloid Leukemia		Suspension	TF1	LAML
ACH-001684	Acute Myeloid Leukemia	Metastatic	Suspension	UCSDAML1	LAML
ACH-000081	Acute Myeloid Leukemia	Primary	Suspension	GDM1	LAML
ACH-001613	Acute Myeloid Leukemia	Primary	Suspension	OCIAML4	LAML
ACH-000373	Acute Myeloid Leukemia	Primary	Suspension	SKM1	LAML
ACH-002651	Acute Myeloid Leukemia		Suspension	FKH1	LAML
ACH-000190	Acute Myeloid Leukemia		Adherent	HDMYZ	LAML
ACH-000299	Acute Myeloid Leukemia	Primary	Suspension	HNT34	LAML
ACH-000113	Acute Myeloid Leukemia	Primary	Suspension	OCIAML2	LAML
ACH-001574	Acute Myeloid Leukemia	Primary	Suspension	MOLM14	LAML
ACH-000005	Acute Myeloid Leukemia		Mixed	HEL9217	LAML
ACH-000557	Acute Myeloid Leukemia	Primary	Suspension	AML193	LAML
ACH-000294	Acute Myeloid Leukemia	Primary	Suspension	NB4	LAML
ACH-000084	with inv(3)(q21.3q26.2) or t(3;3)(q21.3;q26.2); GATA2, MECOM		Adherent	MUTZ3	LAML
ACH-001656	AML with Maturation	Metastatic	Suspension	SKNO1	LAML
ACH-002716	AML with Minimal Differentiation		Suspension	MO91	LAML
ACH-002668	AML with Minimal Differentiation		Suspension	KASUMI3	LAML
ACH-000627	Large Cell Lung Carcinoma	Metastatic	Adherent	LCLC103H	LCLC
ACH-000596	Large Cell Lung Carcinoma	Primary	Adherent	LCLC97TM1	LCLC
ACH-000672	Large Cell Lung Carcinoma	Metastatic	Adherent	IALM	LCLC
ACH-000662	Large Cell Lung Carcinoma	Metastatic	Adherent	CORL23	LCLC
ACH-000789	Large Cell Lung Carcinoma	Primary	Adherent	NCIH810	LCLC
ACH-000853	Large Cell Lung Carcinoma	Metastatic	Adherent	NCIH661	LCLC
ACH-000891	Large Cell Lung Carcinoma	Primary	Adherent	HCC1438	LCLC
ACH-000434	Large Cell Lung Carcinoma	Metastatic	Adherent	NCIH1915	LCLC
ACH-000980	Large Cell Lung Carcinoma	Metastatic	Adherent	NCIH1155	LCLC
ACH-000463	Large Cell Lung Carcinoma	Metastatic	Adherent	NCIH460	LCLC
ACH-000904	Large Cell Lung Carcinoma	Metastatic	Suspension	NCIH2106	LCLC
ACH-000813	Large Cell Lung Carcinoma	Metastatic	Adherent	T3M10	LCLC
ACH-000510	Large Cell Lung Carcinoma	Metastatic	Adherent	NCIH1299	LCLC
ACH-000015	Large Cell Lung Carcinoma	Primary	Adherent	NCIH1581	LCLC
ACH-002475	Chronic Myeloid Leukemia, BCR-ABL1+		Adherent	HAP1	LCML
ACH-000076	Chronic Myeloid Leukemia, BCR-ABL1+	Primary	Suspension	NCO2	LCML
ACH-000326	Chronic Myeloid Leukemia, BCR-ABL1+	Primary	Suspension	JURLMK1	LCML
ACH-000551	Chronic Myeloid Leukemia, BCR-ABL1+	Metastatic	Suspension	K562	LCML
ACH-000983	Chronic Myeloid Leukemia, BCR-ABL1+		Suspension	KCL22	LCML
ACH-000432	Chronic Myeloid Leukemia, BCR-ABL1+	Primary	Suspension	BV173	LCML
ACH-001573	Chronic Myeloid Leukemia, BCR-ABL1+	Metastatic	Suspension	MOLM1	LCML
ACH-000604	Chronic Myeloid Leukemia, BCR-ABL1+	Primary	Suspension	KYO1	LCML
ACH-000241	Chronic Myeloid Leukemia, BCR-ABL1+	Primary	Suspension	JK1	LCML
ACH-000301	Chronic Myeloid Leukemia, BCR-ABL1+	Primary	Suspension	LAMA84	LCML
ACH-000072	Chronic Myeloid Leukemia, BCR-ABL1+		Suspension	MEG01	LCML
ACH-000295	Chronic Myeloid Leukemia, BCR-ABL1+		Suspension	EM2	LCML
ACH-000074	Chronic Myeloid Leukemia, BCR-ABL1+	Primary	Adherent	KU812	LCML
ACH-002061	Chronic Myeloid Leukemia, BCR-ABL1+	Primary	Suspension	P2URK562	LCML
ACH-000462	Chronic Myeloid Leukemia, BCR-ABL1+	Primary	Suspension	NALM1	LCML
ACH-000321	Chronic Myeloid Leukemia, BCR-ABL1+	Primary	Suspension	MOLM6	LCML
ACH-002676	Chronic Myeloid Leukemia, BCR-ABL1+		Suspension	TK6	LCML
ACH-000920	Chronic Myeloid Leukemia, BCR-ABL1+	Primary	Suspension	CMLT1	LCML
ACH-000208	Astrocytoma	Primary	Adherent	U178	LGG
ACH-001016	Astrocytoma	Primary	Adherent	BECKER	LGG
ACH-000329	Astrocytoma	Primary	Adherent	CCFSTTG1	LGG
ACH-000040	Astrocytoma	Primary	Adherent	U118MG	LGG
ACH-000437	Astrocytoma	Primary	Adherent	SW1088	LGG

ACH-000655	Astrocytoma	Primary	Adherent	SF268	LGG
ACH-000389	Astrocytoma	Primary	Adherent	H4	LGG
ACH-000232	Astrocytoma	Primary	Adherent	U251MG	LGG
ACH-000592	Astrocytoma	Primary	Adherent	TM31	LGG
ACH-001172	Astrocytoma	Primary	Adherent	U251MGDM	LGG
ACH-000128	Astrocytoma	Primary	Adherent	LN319	LGG
ACH-000591	Astrocytoma	Primary	Adherent	LN235	LGG
ACH-000807	Oligodendroglioma	Primary	Adherent	SNU738	LGG
ACH-000067	Oligodendroglioma	Primary	Adherent	HS683	LGG
ACH-000126	Oligodendroglioma	Primary	Adherent	KG1C	LGG
ACH-000671	Hepatoblastoma	Primary	Adherent	HUH6	LIHC
ACH-000739	Hepatoblastoma	Primary	Adherent	HEPG2	LIHC
ACH-000625	Hepatocellular Carcinoma	Primary	Mixed	HEP3B217	LIHC
ACH-000848	Hepatocellular Carcinoma	Primary	Adherent	JHH7	LIHC
ACH-000686	Hepatocellular Carcinoma	Primary	Adherent	SNU878	LIHC
ACH-002523	Hepatocellular Carcinoma	Primary	Adherent	SNU739	LIHC
ACH-000361	Hepatocellular Carcinoma	Metastatic	Adherent	SKHEP1	LIHC
ACH-000420	Hepatocellular Carcinoma	Primary	Adherent	SNU449	LIHC
ACH-000476	Hepatocellular Carcinoma	Primary	Adherent	JHH4	LIHC
ACH-000393	Hepatocellular Carcinoma	Primary	Adherent	HLF	LIHC
ACH-000483	Hepatocellular Carcinoma	Primary	Adherent	SNU182	LIHC
ACH-000537	Hepatocellular Carcinoma	Primary	Adherent	SNU761	LIHC
ACH-000577	Hepatocellular Carcinoma	Metastatic	Adherent	JHH2	LIHC
ACH-000734	Hepatocellular Carcinoma	Primary	Adherent	JHH5	LIHC
ACH-000480	Hepatocellular Carcinoma	Primary	Adherent	HUH7	LIHC
ACH-000316	Hepatocellular Carcinoma	Primary	Adherent	SNU886	LIHC
ACH-001318	Hepatocellular Carcinoma	Primary	Adherent	PLCPRF5	LIHC
ACH-000493	Hepatocellular Carcinoma	Primary	Adherent	SNU423	LIHC
ACH-000221	Hepatocellular Carcinoma	Primary	Adherent	SNU398	LIHC
ACH-000475	Hepatocellular Carcinoma	Primary	Adherent	HUH1	LIHC
ACH-000620	Hepatocellular Carcinoma	Primary	Adherent	JHH1	LIHC
ACH-000471	Hepatocellular Carcinoma	Primary	Adherent	LI7	LIHC
ACH-000478	Hepatocellular Carcinoma	Primary	Adherent	SNU387	LIHC
ACH-000217	Hepatocellular Carcinoma	Primary	Adherent	JHH6	LIHC
ACH-000422	Hepatocellular Carcinoma	Primary	Adherent	SNU475	LIHC
ACH-001853	patocellular Carcinoma plus Intrahepatic Cholangiocarcin	Primary	Adherent	KMCH1	LIHC
ACH-000035	Non-Small Cell Lung Cancer	Metastatic	Adherent	NCIH1650	LSCLC
ACH-000186	Non-Small Cell Lung Cancer	Primary	Adherent	NCIH2444	LSCLC
ACH-000924	Non-Small Cell Lung Cancer	Primary	Adherent	NCIH2172	LSCLC
ACH-000603	Non-Small Cell Lung Cancer	Metastatic	Adherent	BEN	LSCLC
ACH-000251	Non-Small Cell Lung Cancer	Primary	Adherent	NCIH2887	LSCLC
ACH-000826	Non-Small Cell Lung Cancer	Primary	Adherent	CAL12T	LSCLC
ACH-002996	Non-Small Cell Lung Cancer	Metastatic	Mixed	CI34601	LSCLC
ACH-000867	Non-Small Cell Lung Cancer	Metastatic	Adherent	CHAGOK1	LSCLC
ACH-000929	Non-Small Cell Lung Cancer	Metastatic	Adherent	NCIH2110	LSCLC
ACH-000825	Non-Small Cell Lung Cancer	Primary	Mixed	HOP92	LSCLC
ACH-002035	Poorly Differentiated Non-Small Cell Lung Cancer	Primary	Adherent	LCAM1	LSCLC
ACH-000327	Lung Adenocarcinoma	Primary	Adherent	NCIH1395	LUAD
ACH-000528	Lung Adenocarcinoma	Metastatic	Adherent	ABC1	LUAD
ACH-000587	Lung Adenocarcinoma	Primary	Adherent	NCIH1975	LUAD
ACH-000785	Lung Adenocarcinoma	Metastatic	Adherent	NCIH2126	LUAD
ACH-000392	Lung Adenocarcinoma	Metastatic	Adherent	CALU3	LUAD
ACH-000638	Lung Adenocarcinoma	Metastatic	Adherent	NCIH441	LUAD
ACH-000578	Lung Adenocarcinoma	Primary	Adherent	HCC1171	LUAD
ACH-000033	Lung Adenocarcinoma	Metastatic	Adherent	NCIH1819	LUAD
ACH-000787	Lung Adenocarcinoma	Primary	Adherent	LXF289	LUAD
ACH-000677	Lung Adenocarcinoma	Primary	Adherent	SW1573	LUAD
ACH-000012	Lung Adenocarcinoma	Primary	Adherent	HCC827	LUAD
ACH-000945	Lung Adenocarcinoma	Metastatic	Suspension	NCIH650	LUAD
ACH-000282	Lung Adenocarcinoma	Metastatic	Mixed	NCIH1755	LUAD
ACH-000681	Lung Adenocarcinoma	Primary	Adherent	A549	LUAD
ACH-000379	Lung Adenocarcinoma	Metastatic	Adherent	NCIH1781	LUAD
ACH-000109	Lung Adenocarcinoma	Primary	Mixed	NCIH3255	LUAD
ACH-000712	Lung Adenocarcinoma	Primary	Adherent	HCC1833	LUAD
ACH-000562	Lung Adenocarcinoma	Metastatic	Adherent	HCC78	LUAD
ACH-000066	Lung Adenocarcinoma	Metastatic	Adherent	HCC4006	LUAD
ACH-000314	Lung Adenocarcinoma	Primary	Adherent	HCC2108	LUAD
ACH-000893	Lung Adenocarcinoma	Primary	Adherent	NCIH1651	LUAD
ACH-002650	Lung Adenocarcinoma	Metastatic	Adherent	ETCC016	LUAD
ACH-000590	Lung Adenocarcinoma	Primary	Adherent	NCIH2073	LUAD
ACH-000339	Lung Adenocarcinoma	Primary	Adherent	HCC461	LUAD
ACH-000744	Lung Adenocarcinoma	Metastatic	Adherent	NCIH1623	LUAD
ACH-000675	Lung Adenocarcinoma	Primary	Adherent	NCIH1734	LUAD
ACH-000851	Lung Adenocarcinoma	Primary	Adherent	MORCPR	LUAD

ACH-000414	Lung Adenocarcinoma	Metastatic	Adherent	NCIH1944	LUAD
ACH-002522	Lung Adenocarcinoma	Metastatic	Adherent	SNU2292	LUAD
ACH-000706	Lung Adenocarcinoma	Primary	Adherent	EKVX	LUAD
ACH-002526	Lung Adenocarcinoma	Metastatic	Adherent	SNU1327	LUAD
ACH-000951	Lung Adenocarcinoma	Primary	Mixed	NCIH2342	LUAD
ACH-000589	Lung Adenocarcinoma	Metastatic	Adherent	NCIH1437	LUAD
ACH-000264	Lung Adenocarcinoma	Metastatic	Adherent	CALU6	LUAD
ACH-000667	Lung Adenocarcinoma	Primary	Adherent	HCC44	LUAD
ACH-000718	Lung Adenocarcinoma	Metastatic	Adherent	NCIH2291	LUAD
ACH-000021	Lung Adenocarcinoma	Metastatic	Adherent	NCIH1693	LUAD
ACH-000845	Lung Adenocarcinoma	Primary	Adherent	NCIH1373	LUAD
ACH-000852	Lung Adenocarcinoma	Primary	Mixed	NCIH1435	LUAD
ACH-000448	Lung Adenocarcinoma	Metastatic	Mixed	NCIH1666	LUAD
ACH-000766	Lung Adenocarcinoma	Metastatic	Adherent	NCIH1648	LUAD
ACH-000343	Lung Adenocarcinoma	Primary	Adherent	NCIH522	LUAD
ACH-000774	Lung Adenocarcinoma	Primary	Adherent	RERFLCAD2	LUAD
ACH-000733	Lung Adenocarcinoma	Primary	Adherent	NCIH1838	LUAD
ACH-003012	Lung Adenocarcinoma	Primary	Adherent	A549CRAFKD	LUAD
ACH-000482	Lung Adenocarcinoma	Primary	Adherent	RERFLCKJ	LUAD
ACH-000779	Lung Adenocarcinoma	Primary	Adherent	PC9	LUAD
ACH-000575	Lung Adenocarcinoma	Primary	Adherent	HCC364	LUAD
ACH-000451	Lung Adenocarcinoma	Primary	Adherent	NCIH2085	LUAD
ACH-000161	Lung Adenocarcinoma	Metastatic	Adherent	CORL105	LUAD
ACH-000916	Lung Adenocarcinoma	Metastatic	Adherent	NCIH1573	LUAD
ACH-000837	Lung Adenocarcinoma	Primary	Adherent	NCIH322	LUAD
ACH-000861	Lung Adenocarcinoma	Primary	Adherent	HOP62	LUAD
ACH-000757	Lung Adenocarcinoma	Primary	Adherent	A427	LUAD
ACH-000447	Lung Adenocarcinoma	Primary	Adherent	NCIH2228	LUAD
ACH-000892	Lung Adenocarcinoma	Primary	Adherent	NCIH1563	LUAD
ACH-000416	Lung Adenocarcinoma	Metastatic	Adherent	NCIH838	LUAD
ACH-000875	Lung Adenocarcinoma	Primary	Mixed	NCIH2347	LUAD
ACH-000841	Lung Adenocarcinoma	Metastatic	Adherent	NCIH2087	LUAD
ACH-000860	Lung Adenocarcinoma	Primary	Adherent	NCIH358	LUAD
ACH-000311	Lung Adenocarcinoma	Metastatic	Adherent	NCIH2122	LUAD
ACH-000496	Lung Adenocarcinoma	Metastatic	Adherent	NCIH1792	LUAD
ACH-000062	Lung Adenocarcinoma	Primary	Adherent	RERFLCMS	LUAD
ACH-000521	Lung Adenocarcinoma	Metastatic	Adherent	NCIH2030	LUAD
ACH-000029	Lung Adenocarcinoma	Primary	Adherent	HCC827GR5	LUAD
ACH-000150	Lung Adenocarcinoma	Primary	Adherent	HCC2935	LUAD
ACH-002531	Lung Adenocarcinoma	Metastatic	Adherent	SNU2535	LUAD
ACH-000869	Lung Adenocarcinoma	Metastatic	Adherent	NCIH1568	LUAD
ACH-000337	Lung Adenocarcinoma	Primary	Adherent	NCIH3122	LUAD
ACH-000886	Lung Adenocarcinoma	Metastatic	Adherent	NCIH2009	LUAD
ACH-000888	Lung Adenocarcinoma	Primary	Adherent	NCIH1793	LUAD
ACH-000030	Lung Adenocarcinoma	Primary	Adherent	PC14	LUAD
ACH-002497	Lung Adenocarcinoma	Primary	Suspension	NCIH920	LUAD
ACH-000925	Lung Adenocarcinoma	Metastatic	Adherent	DV90	LUAD
ACH-000791	Lung Adenocarcinoma	Primary	Adherent	RERFLCAD1	LUAD
ACH-000900	Lung Adenocarcinoma	Primary	Adherent	NCIH23	LUAD
ACH-000666	Lung Adenocarcinoma	Metastatic	Suspension	NCIH1355	LUAD
ACH-000872	Lung Adenocarcinoma	Primary	Adherent	HCC515	LUAD
ACH-000121	Lung Adenocarcinoma	Metastatic	Mixed	NCIH2405	LUAD
ACH-000781	Lung Adenocarcinoma	Metastatic	Adherent	NCIH2023	LUAD
ACH-000868	Lung Adenosquamous Carcinoma	Primary	Adherent	HCC1195	LUAD
ACH-000628	Lung Adenosquamous Carcinoma	Primary	Adherent	NCIH596	LUAD
ACH-000840	Lung Adenosquamous Carcinoma	Primary	Adherent	HCC366	LUAD
ACH-000378	Lung Adenosquamous Carcinoma	Metastatic	Adherent	NCIH647	LUAD
ACH-000731	Lung Adenosquamous Carcinoma	Primary	Adherent	HCC2279	LUAD
ACH-001113	Lung Squamous Cell Carcinoma	Primary	Adherent	LC1SQSF	LUSC
ACH-000705	Lung Squamous Cell Carcinoma	Primary	Mixed	LC1F	LUSC
ACH-000769	Lung Squamous Cell Carcinoma	Primary	Adherent	LK2	LUSC
ACH-000700	Lung Squamous Cell Carcinoma	Primary	Adherent	NCIH2882	LUSC
ACH-000176	Lung Squamous Cell Carcinoma	Primary	Adherent	LOUNH91	LUSC
ACH-000585	Lung Squamous Cell Carcinoma	Primary	Adherent	EPLC272H	LUSC
ACH-000894	Lung Squamous Cell Carcinoma	Metastatic	Adherent	NCIH1869	LUSC
ACH-000481	Lung Squamous Cell Carcinoma	Primary	Adherent	NCIH2170	LUSC
ACH-000690	Lung Squamous Cell Carcinoma	Primary	Adherent	HCC2814	LUSC
ACH-000858	Lung Squamous Cell Carcinoma	Metastatic	Adherent	KNS62	LUSC
ACH-000261	Lung Squamous Cell Carcinoma	Metastatic	Adherent	RERFLCAI	LUSC
ACH-000511	Lung Squamous Cell Carcinoma	Metastatic	Adherent	CALU1	LUSC
ACH-000975	Lung Squamous Cell Carcinoma	Primary	Adherent	HCC2450	LUSC
ACH-000553	Lung Squamous Cell Carcinoma	Primary	Adherent	SQ1	LUSC
ACH-000665	Lung Squamous Cell Carcinoma	Metastatic	Adherent	SKMES1	LUSC
ACH-000737	Lung Squamous Cell Carcinoma	Metastatic	Suspension	NCIH1385	LUSC

ACH-000669	Lung Squamous Cell Carcinoma	Primary	Adherent	SW900	LUSC
ACH-000843	Lung Squamous Cell Carcinoma	Metastatic	Adherent	HARA	LUSC
ACH-000747	Lung Squamous Cell Carcinoma	Primary	Adherent	NCIH1703	LUSC
ACH-000921	Lung Squamous Cell Carcinoma	Metastatic	Adherent	NCIH157DM	LUSC
ACH-000454	Lung Squamous Cell Carcinoma	Metastatic	Adherent	HCC95	LUSC
ACH-000395	Lung Squamous Cell Carcinoma	Primary	Adherent	NCIH520	LUSC
ACH-000563	Lung Squamous Cell Carcinoma	Metastatic	Adherent	EBC1	LUSC
ACH-000442	Lung Squamous Cell Carcinoma	Primary	Adherent	RERFLCSQ1	LUSC
ACH-000390	Lung Squamous Cell Carcinoma	Primary	Adherent	LUDLU1	LUSC
ACH-000878	Lung Squamous Cell Carcinoma	Primary	Adherent	HCC15	LUSC
ACH-001489	Lung Squamous Cell Carcinoma	Metastatic	Suspension	CORL32	LUSC
ACH-000055	Medulloblastoma	Metastatic	Mixed	D283MED	MB
ACH-001054	Medulloblastoma	Primary	Mixed	D458	MB
ACH-001232	Medulloblastoma	Primary	Adherent	UW228	MB
ACH-000095	Medulloblastoma	Primary	Mixed	D341Med	MB
ACH-000211	Medulloblastoma	Primary	Adherent	DAOY	MB
ACH-000776	Medulloblastoma	Primary	Adherent	ONS76	MB
ACH-001053	Medulloblastoma	Primary	Mixed	D425	MB
ACH-001201	Medulloblastoma	Primary	Adherent	SUMB002	MB
ACH-003107	Mantle Cell Lymphoma		Suspension	JMP1	MCL
ACH-000106	Mantle Cell Lymphoma		Suspension	JVM2	MCL
ACH-000220	Mantle Cell Lymphoma		Suspension	MINO	MCL
ACH-000073	Mantle Cell Lymphoma	Primary	Suspension	GRANTA519	MCL
ACH-002500	Mantle Cell Lymphoma		Suspension	Z138	MCL
ACH-002485	Mantle Cell Lymphoma		Suspension	MAVER1	MCL
ACH-000068	Mantle Cell Lymphoma		Suspension	REC1	MCL
ACH-000357	Mantle Cell Lymphoma		Suspension	JEKO1	MCL
ACH-001561	Pleural Mesothelioma, Biphasic Type	Primary	Adherent	MERO84	MESO
ACH-000092	Pleural Mesothelioma, Biphasic Type	Metastatic	Adherent	NCIH2452	MESO
ACH-001556	Pleural Mesothelioma, Biphasic Type	Primary	Adherent	MERO25	MESO
ACH-001558	Pleural Mesothelioma, Biphasic Type	Primary	Adherent	MERO48A	MESO
ACH-000335	Pleural Mesothelioma, Biphasic Type	Metastatic	Adherent	MSTO211H	MESO
ACH-001560	Pleural Mesothelioma, Biphasic Type	Primary	Adherent	MERO83	MESO
ACH-001547	Pleural Mesothelioma, Epithelioid Type	Primary	Adherent	LO68	MESO
ACH-002033	Pleural Mesothelioma, Epithelioid Type	Primary	Adherent	HMMME	MESO
ACH-001559	Pleural Mesothelioma, Epithelioid Type	Primary	Adherent	MERO82	MESO
ACH-000331	Pleural Mesothelioma, Epithelioid Type		Adherent	ISTMES2	MESO
ACH-001977	Pleural Mesothelioma, Epithelioid Type	Primary	Adherent	NO36	MESO
ACH-000569	Pleural Mesothelioma, Epithelioid Type	Primary	Adherent	ISTMES1	MESO
ACH-001557	Pleural Mesothelioma, Epithelioid Type	Primary	Adherent	MERO41	MESO
ACH-000648	Pleural Mesothelioma, Epithelioid Type	Metastatic	Adherent	NCIH28	MESO
ACH-000367	Pleural Mesothelioma, Epithelioid Type	Metastatic	Adherent	NCIH226	MESO
ACH-000645	Pleural Mesothelioma, Epithelioid Type	Primary	Adherent	JL1	MESO
ACH-000086	Pleural Mesothelioma, Epithelioid Type		Adherent	ACCMESO1	MESO
ACH-001555	Pleural Mesothelioma, Epithelioid Type	Primary	Adherent	MERO14	MESO
ACH-001992	Pleural Mesothelioma, Epithelioid Type	Primary	Adherent	ONE58	MESO
ACH-000319	Pleural Mesothelioma, Epithelioid Type		Adherent	MPP89	MESO
ACH-001562	Pleural Mesothelioma, Epithelioid Type	Primary	Adherent	MERO95	MESO
ACH-000153	Pleural Mesothelioma, Sarcomatoid Type	Metastatic	Adherent	NCIH2052	MESO
ACH-000167	Plasma Cell Myeloma	Metastatic	Mixed	KE97	MM
ACH-001541	Plasma Cell Myeloma	Primary	Suspension	KMS28PE	MM
ACH-000576	Plasma Cell Myeloma	Primary	Suspension	KMS27	MM
ACH-000380	Plasma Cell Myeloma	Primary	Suspension	KMS12BM	MM
ACH-000204	Plasma Cell Myeloma	Primary	Mixed	LP1	MM
ACH-000714	Plasma Cell Myeloma	Primary	Suspension	KMS11	MM
ACH-000763	Plasma Cell Myeloma	Primary	Mixed	MM1S	MM
ACH-000889	Plasma Cell Myeloma	Primary	Mixed	KMM1	MM
ACH-000854	Plasma Cell Myeloma		Mixed	OCIMY5	MM
ACH-000024	Plasma Cell Myeloma	Primary	Suspension	OPM2	MM
ACH-000193	Plasma Cell Myeloma	Primary	Suspension	KARPAS620	MM
ACH-000541	Plasma Cell Myeloma	Primary	Suspension	KMS34	MM
ACH-000419	Plasma Cell Myeloma	Primary	Suspension	KMS28BM	MM
ACH-000821	Plasma Cell Myeloma	Primary	Mixed	EJM	MM
ACH-000050	Plasma Cell Myeloma	Metastatic	Suspension	NCIH929	MM
ACH-000564	Plasma Cell Myeloma	Primary	Suspension	KHM1B	MM
ACH-000512	Plasma Cell Myeloma		Suspension	INA6	MM
ACH-000838	Plasma Cell Myeloma	Primary	Suspension	AMO1	MM
ACH-000653	Plasma Cell Myeloma	Primary	Suspension	JJN3	MM
ACH-000829	Plasma Cell Myeloma		Suspension	HUNS1	MM
ACH-000817	Plasma Cell Myeloma	Primary	Mixed	RPMI8226	MM
ACH-000745	Plasma Cell Myeloma	Primary	Suspension	MOLP8	MM
ACH-000626	Plasma Cell Myeloma		Suspension	U266B1	MM
ACH-000588	Plasma Cell Myeloma	Primary	Suspension	KMS26	MM
ACH-000436	Plasma Cell Myeloma		Suspension	OCIMY7	MM

ACH-000598	Plasma Cell Myeloma	Primary	Suspension	KMS21BM	MM
ACH-000057	Plasma Cell Myeloma		Suspension	OPM1	MM
ACH-000363	Plasma Cell Myeloma	Primary	Suspension	SKMM2	MM
ACH-000183	Plasma Cell Myeloma	Primary	Suspension	L363	MM
ACH-000453	Plasma Cell Myeloma	Primary	Suspension	MOLP2	MM
ACH-000608	Mucinous Ovarian Cancer	Primary	Adherent	COV644	MOC
ACH-000237	Mucinous Ovarian Cancer	Primary	Adherent	JHOM1	MOC
ACH-000936	Mucinous Ovarian Cancer	Metastatic	Adherent	EFO27	MOC
ACH-000333	Mucinous Ovarian Cancer	Primary	Adherent	JHOM2B	MOC
ACH-000796	Mucinous Ovarian Cancer	Primary	Adherent	MCAS	MOC
ACH-000701	Mucinous Ovarian Cancer	Primary	Adherent	RMUGS	MOC
ACH-002066	Malignant Peripheral Nerve Sheath Tumor	Primary	Adherent	HSSCH2	MPNST
ACH-002695	Malignant Peripheral Nerve Sheath Tumor	Unknown	Adherent	STS26T	MPNST
ACH-002693	Malignant Peripheral Nerve Sheath Tumor	Unknown	Adherent	S462	MPNST
ACH-002710	Malignant Peripheral Nerve Sheath Tumor	Primary	Adherent	MPNST724	MPNST
ACH-002799	Malignant Peripheral Nerve Sheath Tumor	Unknown	Adherent	NCCMPNST1C1	MPNST
ACH-002800	Malignant Peripheral Nerve Sheath Tumor	Unknown	Adherent	NCCMPNST2C1	MPNST
ACH-002801	Malignant Peripheral Nerve Sheath Tumor	Unknown	Adherent	NCCMPNST3C1	MPNST
ACH-002065	Malignant Peripheral Nerve Sheath Tumor	Metastatic	Adherent	HSPSS	MPNST
ACH-002802	Malignant Peripheral Nerve Sheath Tumor		Adherent	NCCMPNST3X2C1	MPNST
ACH-001200	Rhabdoid Cancer	Metastatic	Adherent	STM9101	MRT
ACH-000597	Rhabdoid Cancer		Adherent	TTC709	MRT
ACH-001099	Rhabdoid Cancer		Adherent	KD	MRT
ACH-000533	Rhabdoid Cancer		Adherent	NCIH2004RT	MRT
ACH-000172	Rhabdoid Cancer	Metastatic	Adherent	TM87	MRT
ACH-000201	Rhabdoid Cancer	Primary	Adherent	A204	MRT
ACH-001210	Rhabdoid Cancer	Metastatic	Adherent	TTC1240	MRT
ACH-000096	Rhabdoid Cancer	Primary	Adherent	G401	MRT
ACH-001211	Rhabdoid Cancer		Adherent	TTC549	MRT
ACH-001109	Rhabdoid Cancer		Adherent	KPMRTRY	MRT
ACH-001059	Rhabdoid Cancer		Unknown	DL	MRT
ACH-001532	Rhabdoid Cancer	Metastatic	Adherent	JMURTK2	MRT
ACH-001128	Rhabdoid Cancer		Adherent	MON	MRT
ACH-000260	Neuroblastoma	Metastatic	Adherent	SKNAS	NBL
ACH-001302	Neuroblastoma	Unknown	Mixed	COGN305	NBL
ACH-001481	Neuroblastoma	Metastatic	Adherent	CHLA90	NBL
ACH-000310	Neuroblastoma	Metastatic	Adherent	IMR32	NBL
ACH-000203	Neuroblastoma	Metastatic	Adherent	NH6	NBL
ACH-000136	Neuroblastoma	Primary	Adherent	CHP126	NBL
ACH-002080	Neuroblastoma	Primary	Adherent	TN2	NBL
ACH-001301	Neuroblastoma	Unknown	Adherent	COGN278	NBL
ACH-001338	Neuroblastoma	Primary	Adherent	CHP134	NBL
ACH-000446	Neuroblastoma	Metastatic	Adherent	KPNSI9S	NBL
ACH-000078	Neuroblastoma	Metastatic	Mixed	MHHNB11	NBL
ACH-000099	Neuroblastoma	Primary	Adherent	SIMA	NBL
ACH-000149	Neuroblastoma	Metastatic	Adherent	SKNSH	NBL
ACH-000227	Neuroblastoma	Metastatic	Adherent	KPNYN	NBL
ACH-001303	Neuroblastoma	Unknown	Adherent	NB1643	NBL
ACH-001300	Neuroblastoma	Primary	Mixed	CHLA15	NBL
ACH-000341	Neuroblastoma	Metastatic	Adherent	SKNFI	NBL
ACH-000120	Neuroblastoma	Primary	Adherent	CHP212	NBL
ACH-001366	Neuroblastoma	Metastatic	Adherent	NGP	NBL
ACH-001354	Neuroblastoma	Primary	Adherent	LAN2	NBL
ACH-001188	Neuroblastoma	Metastatic	Adherent	SHSY5Y	NBL
ACH-000804	Neuroblastoma	Metastatic	Adherent	NB1	NBL
ACH-001603	Neuroblastoma	Primary	Adherent	NH12	NBL
ACH-001548	Neuroblastoma	Metastatic	Adherent	LS	NBL
ACH-001465	Neuroblastoma	Metastatic	Suspension	CHLA136	NBL
ACH-002431	Neuroblastoma	Primary	Adherent	CCLFPEDS0014T1	NBL
ACH-000366	Neuroblastoma	Metastatic	Adherent	SKNDZ	NBL
ACH-002922	Neuroblastoma	Metastatic	Adherent	SKNMM	NBL
ACH-000345	Neuroblastoma	Metastatic	Mixed	KPNRTBM1	NBL
ACH-001344	Neuroblastoma	Metastatic	Adherent	GIMEN	NBL
ACH-000259	Neuroblastoma	Primary	Adherent	KELLY	NBL
ACH-001716	Neuroblastoma	Primary	Adherent	GOTO	NBL
ACH-000312	Neuroblastoma	Metastatic	Adherent	SKNBE2	NBL
ACH-002283	Neuroblastoma	Metastatic	Adherent	NB5	NBL
ACH-001674	Neuroblastoma	Primary	Adherent	TGW	NBL
ACH-001367	Neuroblastoma	Metastatic	Adherent	NMB	NBL
ACH-000324	Clear Cell Ovarian Cancer	Primary	Adherent	JHOC5	OCCC
ACH-000646	Clear Cell Ovarian Cancer	Primary	Adherent	OVMANA	OCCC
ACH-000719	Clear Cell Ovarian Cancer	Metastatic	Adherent	RMGI	OCCC
ACH-000906	Clear Cell Ovarian Cancer	Primary	Adherent	ES2	OCCC
ACH-001719	Clear Cell Ovarian Cancer	Primary	Adherent	OCIC4P	OCCC

ACH-000527	Clear Cell Ovarian Cancer	Metastatic	Adherent	OVISE	OCCC
ACH-001369	Clear Cell Ovarian Cancer	Primary	Adherent	OCIC5X	OCCC
ACH-000663	Clear Cell Ovarian Cancer	Metastatic	Adherent	OVTOKO	OCCC
ACH-000885	Clear Cell Ovarian Cancer	Primary	Adherent	TOV21G	OCCC
ACH-001991	Endometrioid Ovarian Cancer	Primary	Mixed	NZOV9	OEC
ACH-000947	Endometrioid Ovarian Cancer	Metastatic	Adherent	OVK18	OEC
ACH-002524	Endometrioid Ovarian Cancer	Metastatic	Adherent	SNU251	OEC
ACH-000048	Endometrioid Ovarian Cancer	Primary	Adherent	TOV112D	OEC
ACH-000966	Endometrioid Ovarian Cancer	Primary	Adherent	IGROV1	OEC
ACH-000657	Endometrioid Ovarian Cancer	Primary	Adherent	A2780	OEC
ACH-001814	Osteosarcoma	Primary	Adherent	OS252	OS
ACH-002471	Osteosarcoma	Primary	Adherent	OS052	OS
ACH-001817	Osteosarcoma	Metastatic	Adherent	C393	OS
ACH-002834	Osteosarcoma	Primary	Adherent	OS384	OS
ACH-002067	Osteosarcoma	Primary	Adherent	NOS1	OS
ACH-000748	Osteosarcoma	Primary	Adherent	SJSA1	OS
ACH-002778	Osteosarcoma	Unknown	Adherent	NCCOS1X2C1	OS
ACH-001715	Osteosarcoma	Primary	Adherent	CAL72	OS
ACH-001001	Osteosarcoma	Primary	Adherent	143B	OS
ACH-000613	Osteosarcoma	Primary	Adherent	HOS	OS
ACH-000359	Osteosarcoma	Primary	Adherent	MG63	OS
ACH-001712	Osteosarcoma	Primary	Adherent	HS860T	OS
ACH-002689	Osteosarcoma	Metastatic	Adherent	OS525	OS
ACH-002470	Osteosarcoma	Metastatic	Adherent	OS186	OS
ACH-000410	Osteosarcoma	Primary	Adherent	SAOS2	OS
ACH-000364	Osteosarcoma	Primary	Adherent	U2OS	OS
ACH-002069	Osteosarcoma	Primary	Adherent	HSOS1	OS
ACH-001275	Osteosarcoma		Adherent	OSA1777	OS
ACH-000082	Osteosarcoma	Primary	Adherent	G292CLONEA141B1	OS
ACH-002433	Osteosarcoma	Primary	Adherent	CCLFPEDS0019T	OS
ACH-001526	Osteosarcoma	Primary	Adherent	HUO9	OS
ACH-001813	Osteosarcoma	Primary	Adherent	C242	OS
ACH-002688	Osteosarcoma	Primary	Adherent	OS457	OS
ACH-001818	Osteosarcoma	Metastatic	Adherent	C396	OS
ACH-002690	Osteosarcoma	Primary	Adherent	OS526	OS
ACH-000520	High-Grade Serous Ovarian Cancer	Metastatic	Adherent	59M	OV
ACH-000116	High-Grade Serous Ovarian Cancer	Metastatic	Adherent	OAW28	OV
ACH-000584	High-Grade Serous Ovarian Cancer	Primary	Adherent	JHOS4	OV
ACH-000574	High-Grade Serous Ovarian Cancer	Primary	Adherent	FUOV1	OV
ACH-000430	High-Grade Serous Ovarian Cancer	Primary	Adherent	TYKNU	OV
ACH-000713	High-Grade Serous Ovarian Cancer	Primary	Adherent	CAOV3	OV
ACH-000132	High-Grade Serous Ovarian Cancer	Primary	Adherent	JHOS2	OV
ACH-000409	High-Grade Serous Ovarian Cancer	Metastatic	Adherent	OVSAHO	OV
ACH-000001	High-Grade Serous Ovarian Cancer	Metastatic	Adherent	NIHOVCAR3	OV
ACH-000524	High-Grade Serous Ovarian Cancer	Metastatic	Adherent	KURAMOCHI	OV
ACH-000278	High-Grade Serous Ovarian Cancer	Metastatic	Adherent	COV362	OV
ACH-000696	High-Grade Serous Ovarian Cancer	Primary	Adherent	OVCAR8	OV
ACH-000635	High-Grade Serous Ovarian Cancer	Metastatic	Adherent	SNU119	OV
ACH-000103	High-Grade Serous Ovarian Cancer	Metastatic	Adherent	CAOV4	OV
ACH-000617	High-Grade Serous Ovarian Cancer	Metastatic	Adherent	OVCAR4	OV
ACH-001628	High-Grade Serous Ovarian Cancer	Metastatic	Adherent	PEA1	OV
ACH-000013	High-Grade Serous Ovarian Cancer	Metastatic	Adherent	ONCODG1	OV
ACH-000443	High-Grade Serous Ovarian Cancer	Primary	Adherent	OVKATE	OV
ACH-000256	High-Grade Serous Ovarian Cancer	Metastatic	Adherent	COV318	OV
ACH-001151	High-Grade Serous Ovarian Cancer	Metastatic	Adherent	OVCAR5	OV
ACH-000542	High-Grade Serous Ovarian Cancer	Metastatic	Adherent	HEYA8	OV
ACH-000704	Serous Ovarian Cancer	Metastatic	Adherent	OAW42	OV
ACH-001403	Serous Ovarian Cancer	Metastatic	Adherent	TO14	OV
ACH-001340	Serous Ovarian Cancer	Metastatic	Adherent	COV413A	OV
ACH-001630	Serous Ovarian Cancer	Metastatic	Adherent	PEO1	OV
ACH-002486	Serous Ovarian Cancer	Primary	Adherent	MESOV	OV
ACH-001418	Serous Ovarian Cancer	Primary	Adherent	UWB1289	OV
ACH-001145	Serous Ovarian Cancer	Metastatic	Adherent	OC316	OV
ACH-001373	Serous Ovarian Cancer	Metastatic	Adherent	OV17R	OV
ACH-000811	Serous Ovarian Cancer	Metastatic	Adherent	SKOV3	OV
ACH-000308	Serous Ovarian Cancer	Metastatic	Adherent	EFO21	OV
ACH-000962	Serous Ovarian Cancer	Metastatic	Adherent	OC314	OV
ACH-001370	Serous Ovarian Cancer	Primary	Adherent	OCIP5X	OV
ACH-000091	Serous Ovarian Cancer	Metastatic	Adherent	OV56	OV
ACH-000460	Serous Ovarian Cancer	Metastatic	Adherent	SNU8	OV
ACH-000291	Serous Ovarian Cancer	Metastatic	Adherent	OV90	OV
ACH-001632	Serous Ovarian Cancer	Metastatic	Adherent	PEO4	OV
ACH-001048	Serous Ovarian Cancer	Metastatic	Adherent	COV504	OV
ACH-000685	Adenosquamous Carcinoma of the Pancreas	Metastatic	Mixed	L33	PAAD

ACH-000108	Adenosquamous Carcinoma of the Pancreas	Metastatic	Adherent	KP3	PAAD
ACH-001098	Pancreatic Adenocarcinoma	Primary	Adherent	KCIMOH1	PAAD
ACH-001375	Pancreatic Adenocarcinoma	Primary	Adherent	PACADD119	PAAD
ACH-001377	Pancreatic Adenocarcinoma	Primary	Adherent	PACADD137	PAAD
ACH-000138	Pancreatic Adenocarcinoma	Metastatic	Adherent	CFPAC1	PAAD
ACH-000222	Pancreatic Adenocarcinoma	Metastatic	Adherent	ASPC1	PAAD
ACH-000031	Pancreatic Adenocarcinoma	Primary	Adherent	PANC0213	PAAD
ACH-000332	Pancreatic Adenocarcinoma	Metastatic	Adherent	YAPC	PAAD
ACH-001382	Pancreatic Adenocarcinoma	Metastatic	Adherent	PACADD188	PAAD
ACH-000213	Pancreatic Adenocarcinoma	Metastatic	Adherent	HUPT4	PAAD
ACH-000354	Pancreatic Adenocarcinoma	Metastatic	Adherent	CAPAN1	PAAD
ACH-001378	Pancreatic Adenocarcinoma	Primary	Adherent	PACADD159	PAAD
ACH-000164	Pancreatic Adenocarcinoma	Primary	Adherent	PANC1	PAAD
ACH-000517	Pancreatic Adenocarcinoma	Metastatic	Adherent	SNU410	PAAD
ACH-000022	Pancreatic Adenocarcinoma	Metastatic	Adherent	PATU8988S	PAAD
ACH-000235	Pancreatic Adenocarcinoma	Primary	Adherent	PANC0403	PAAD
ACH-000094	Pancreatic Adenocarcinoma	Metastatic	Adherent	HPAFII	PAAD
ACH-000599	Pancreatic Adenocarcinoma	Primary	Adherent	PATU8902	PAAD
ACH-000139	Pancreatic Adenocarcinoma	Primary	Adherent	PANC0327	PAAD
ACH-000155	Pancreatic Adenocarcinoma	Primary	Adherent	SW1990	PAAD
ACH-000270	Pancreatic Adenocarcinoma	Primary	Adherent	HPAC	PAAD
ACH-002039	Pancreatic Adenocarcinoma	Metastatic	Adherent	PK8	PAAD
ACH-000118	Pancreatic Adenocarcinoma	Metastatic	Adherent	HUPT3	PAAD
ACH-000265	Pancreatic Adenocarcinoma	Metastatic	Adherent	KP4	PAAD
ACH-003433	Pancreatic Adenocarcinoma	Metastatic	Adherent	CCLFPANC0019T	PAAD
ACH-000652	Pancreatic Adenocarcinoma	Metastatic	Adherent	SUIT2	PAAD
ACH-000093	Pancreatic Adenocarcinoma	Primary	Adherent	PANC0504	PAAD
ACH-000060	Pancreatic Adenocarcinoma	Primary	Adherent	PANC1005	PAAD
ACH-000243	Pancreatic Adenocarcinoma	Primary	Adherent	DANG	PAAD
ACH-001379	Pancreatic Adenocarcinoma	Metastatic	Adherent	PACADD161	PAAD
ACH-000417	Pancreatic Adenocarcinoma	Primary	Adherent	PANC0813	PAAD
ACH-000535	Pancreatic Adenocarcinoma	Primary	Adherent	BXPC3	PAAD
ACH-000178	Pancreatic Adenocarcinoma	Metastatic	Adherent	HS766T	PAAD
ACH-002672	Pancreatic Adenocarcinoma	Primary	Adherent	MAPACHS77	PAAD
ACH-000107	Pancreatic Adenocarcinoma	Primary	Adherent	CAPAN2	PAAD
ACH-000601	Pancreatic Adenocarcinoma	Primary	Adherent	MIAPACA2	PAAD
ACH-000042	Pancreatic Adenocarcinoma	Primary	Adherent	PANC0203	PAAD
ACH-000205	Pancreatic Adenocarcinoma	Metastatic	Adherent	PK59	PAAD
ACH-000468	Pancreatic Adenocarcinoma	Primary	Adherent	PK45H	PAAD
ACH-001380	Pancreatic Adenocarcinoma	Metastatic	Adherent	PACADD165	PAAD
ACH-001376	Pancreatic Adenocarcinoma	Metastatic	Adherent	PACADD135	PAAD
ACH-000266	Pancreatic Adenocarcinoma	Primary	Adherent	SNU213	PAAD
ACH-001999	Pancreatic Adenocarcinoma	Primary	Adherent	9505BIK	PAAD
ACH-000085	Pancreatic Adenocarcinoma	Primary	Adherent	T3M4	PAAD
ACH-000023	Pancreatic Adenocarcinoma	Metastatic	Adherent	PATU8988T	PAAD
ACH-000281	Pancreatic Adenocarcinoma	Primary	Adherent	KP2	PAAD
ACH-000307	Pancreatic Adenocarcinoma	Metastatic	Adherent	PK1	PAAD
ACH-001353	Pancreatic Adenocarcinoma	Primary	Adherent	JOPACA1	PAAD
ACH-000502	Pancreatic Adenocarcinoma	Metastatic	Adherent	TCCPAN2	PAAD
ACH-000933	Pancreatic Adenocarcinoma	Primary	Mixed	SNU324	PAAD
ACH-000320	Pancreatic Adenocarcinoma	Primary	Adherent	PSN1	PAAD
ACH-000114	Pancreatic Adenocarcinoma	Metastatic	Adherent	SU8686	PAAD
ACH-000952	Prostate Adenocarcinoma	Metastatic	Adherent	MDAPCA2B	PRAD
ACH-000956	Prostate Adenocarcinoma	Primary	Adherent	22RV1	PRAD
ACH-000090	Prostate Adenocarcinoma	Metastatic	Adherent	PC3	PRAD
ACH-000979	Prostate Adenocarcinoma	Metastatic	Adherent	DU145	PRAD
ACH-000977	Prostate Adenocarcinoma	Metastatic	Adherent	LNCAPCLONEFGC	PRAD
ACH-000115	Prostate Adenocarcinoma	Metastatic	Adherent	VCAP	PRAD
ACH-000708	Rectal Adenocarcinoma	Metastatic	Adherent	SNU283	READ
ACH-000421	Rectal Adenocarcinoma	Primary	Adherent	SW837	READ
ACH-002024	Rectal Adenocarcinoma	Primary	Adherent	ECC4	READ
ACH-000532	Rectal Adenocarcinoma	Metastatic	Adherent	SNU61	READ
ACH-001461	Rectal Adenocarcinoma	Primary	Adherent	C99	READ
ACH-000565	Rectal Adenocarcinoma	Primary	Adherent	RCM1	READ
ACH-000286	Rectal Adenocarcinoma	Primary	Adherent	SNU1033	READ
ACH-000683	Rectal Adenocarcinoma	Metastatic	Adherent	SNU503	READ
ACH-000470	Rectal Adenocarcinoma	Primary	Adherent	SW1463	READ
ACH-002535	Rectal Adenocarcinoma	Primary	Adherent	SNU254	READ
ACH-001459	Rectal Adenocarcinoma	Primary	Adherent	C80	READ
ACH-002532	Rectal Adenocarcinoma	Primary	Adherent	SNU1411	READ
ACH-002025	Rectal Adenocarcinoma	Primary	Adherent	TT1TKB	READ
ACH-002501	Alveolar Rhabdomyosarcoma	Metastatic	Adherent	RMZ	RMS
ACH-001184	Alveolar Rhabdomyosarcoma	Metastatic	Adherent	SCMCRM2	RMS
ACH-001745	Alveolar Rhabdomyosarcoma		Adherent	RHJT	RMS

ACH-002936	Alveolar Rhabdomyosarcoma	Metastatic	Mixed	CCLFPEDS0043T	RMS
ACH-000100	Alveolar Rhabdomyosarcoma	Metastatic	Adherent	RH41	RMS
ACH-001096	Alveolar Rhabdomyosarcoma		Adherent	JR	RMS
ACH-001740	Alveolar Rhabdomyosarcoma	Metastatic	Adherent	RH28	RMS
ACH-001050	Alveolar Rhabdomyosarcoma		Adherent	CW9019	RMS
ACH-000833	Alveolar Rhabdomyosarcoma	Metastatic	Adherent	RH30	RMS
ACH-001765	Alveolar Rhabdomyosarcoma	Metastatic	Adherent	RH4	RMS
ACH-001743	Alveolar Rhabdomyosarcoma	Metastatic	Adherent	RC2	RMS
ACH-000689	Embryonal Rhabdomyosarcoma		Adherent	RH18	RMS
ACH-003473	Embryonal Rhabdomyosarcoma	Primary	Adherent	CCLFPEDS0013T	RMS
ACH-002048	Embryonal Rhabdomyosarcoma	Primary	Adherent	RMSYM	RMS
ACH-001751	Embryonal Rhabdomyosarcoma		Adherent	RH36	RMS
ACH-001196	Embryonal Rhabdomyosarcoma		Adherent	SMSCTR	RMS
ACH-001750	Embryonal Rhabdomyosarcoma		Adherent	TTC442	RMS
ACH-000607	Embryonal Rhabdomyosarcoma	Primary	Suspension	KYM1	RMS
ACH-000169	Embryonal Rhabdomyosarcoma	Primary	Adherent	RD	RMS
ACH-002787	Pleomorphic Rhabdomyosarcoma	Primary	Adherent	NCCPRMS1C1	RMS
ACH-000051	Rhabdomyosarcoma	Primary	Unknown	TE617T	RMS
ACH-001804	Dedifferentiated Liposarcoma		Suspension	LPS510	SARC
ACH-001799	Dedifferentiated Liposarcoma		Adherent	LPS141	SARC
ACH-001802	Dedifferentiated Liposarcoma		Adherent	LPS853	SARC
ACH-001807	Dedifferentiated Liposarcoma	Primary	Adherent	LPS067	SARC
ACH-003184	Leiomyosarcoma	Primary	Mixed	NRHLMS2	SARC
ACH-003267	Leiomyosarcoma	Metastatic	Adherent	CCLFRARE0009T	SARC
ACH-003266	Leiomyosarcoma	Primary	Adherent	CCLFRARE0021T	SARC
ACH-000145	Leiomyosarcoma	Primary	Adherent	SKLMS1	SARC
ACH-002785	Leiomyosarcoma	Metastatic	Adherent	NCCLMS1C1	SARC
ACH-003181	Leiomyosarcoma	Metastatic	Mixed	NRHLMS1	SARC
ACH-003269	Leiomyosarcoma	Metastatic	Adherent	CCLFRARE0019T	SARC
ACH-003265	Leiomyosarcoma	Metastatic	Adherent	CCLFRARE0010T	SARC
ACH-002942	Leiomyosarcoma	Primary	Adherent	CCLFRARE0004T	SARC
ACH-003268	Leiomyosarcoma	Metastatic	Adherent	CCLFRARE0022T	SARC
ACH-000505	Leiomyosarcoma	Primary	Adherent	RKN	SARC
ACH-001793	Liposarcoma		Adherent	LPS27	SARC
ACH-001791	Liposarcoma		Adherent	LPS6	SARC
ACH-003464	Liposarcoma	Primary	Adherent	CCLFNPDX0008T	SARC
ACH-001540	Liposarcoma	Primary	Adherent	KMLS1	SARC
ACH-003183	Myxofibrosarcoma	Primary	Mixed	NRHMFS3	SARC
ACH-002794	Myxofibrosarcoma	Primary	Adherent	NCCMFS1C1	SARC
ACH-000037	Sarcoma, NOS		Adherent	S117	SARC
ACH-002790	Synovial Sarcoma	Primary	Adherent	NCCSS2C1	SARC
ACH-001270	Synovial Sarcoma		Adherent	127399	SARC
ACH-001322	Synovial Sarcoma		Adherent	CME1	SARC
ACH-001280	Synovial Sarcoma		Adherent	SCS214	SARC
ACH-001274	Synovial Sarcoma		Adherent	SW982	SARC
ACH-001277	Synovial Sarcoma	Metastatic	Adherent	YAMATO	SARC
ACH-003180	rphic Sarcoma/Malignant Fibrous Histiocytoma/High-Gra	Primary	Mixed	NRHUPS2	SARC
ACH-003177	rphic Sarcoma/Malignant Fibrous Histiocytoma/High-Gra	Metastatic	Mixed	NRHUPS1	SARC
ACH-002782	rphic Sarcoma/Malignant Fibrous Histiocytoma/High-Gra	Primary	Adherent	NCCUPS1C1	SARC
ACH-001164	rphic Sarcoma/Malignant Fibrous Histiocytoma/High-Grade Spindle Cel		Adherent	CCLFPEDS0003T	SARC
ACH-002784	rphic Sarcoma/Malignant Fibrous Histiocytoma/High-Gra	Primary	Adherent	NCCUPS2C1	SARC
ACH-000835	rphic Sarcoma/Malignant Fibrous Histiocytoma/High-Gra	Metastatic	Adherent	GCT	SARC
ACH-003111	rphic Sarcoma/Malignant Fibrous Histiocytoma/High-Grade Spindle Cel		Adherent	HS856T	SARC
ACH-001655	Uterine Leiomyosarcoma	Primary	Adherent	SKN	SARC
ACH-000939	Uterine Leiomyosarcoma	Primary	Adherent	SKUT1	SARC
ACH-000449	Uterine Sarcoma/Mesenchymal	Primary	Adherent	MESSA	SARC
ACH-001794	Well-Differentiated Liposarcoma	Primary	Adherent	93T449	SARC
ACH-001796	Well-Differentiated Liposarcoma		Adherent	95T1000	SARC
ACH-001795	Well-Differentiated Liposarcoma		Adherent	94T778	SARC
ACH-002075	Small Cell Lung Cancer	Primary	Mixed	LU138	SCLC
ACH-000800	Small Cell Lung Cancer	Metastatic	Adherent	NCIH446	SCLC
ACH-000729	Small Cell Lung Cancer	Primary	Suspension	NCIH1963	SCLC
ACH-001046	Small Cell Lung Cancer		Suspension	CORL26	SCLC
ACH-000179	Small Cell Lung Cancer	Metastatic	Suspension	NCIH1618	SCLC
ACH-000871	Small Cell Lung Cancer	Metastatic	Mixed	NCIH510	SCLC
ACH-000749	Small Cell Lung Cancer	Metastatic	Adherent	DMS273	SCLC
ACH-000358	Small Cell Lung Cancer	Metastatic	Suspension	NCIH69	SCLC
ACH-000594	Small Cell Lung Cancer	Metastatic	Mixed	DMS153	SCLC
ACH-003120	Small Cell Lung Cancer	Metastatic	Adherent	NCIH1607	SCLC
ACH-002074	Small Cell Lung Cancer	Primary	Suspension	LU143	SCLC
ACH-000866	Small Cell Lung Cancer	Metastatic	Adherent	NCIH1048	SCLC
ACH-000586	Small Cell Lung Cancer	Metastatic	Adherent	NCIH1876	SCLC
ACH-000394	Small Cell Lung Cancer	Metastatic	Suspension	NCIH2081	SCLC
ACH-000816	Small Cell Lung Cancer	Metastatic	Suspension	NCIH524	SCLC

ACH-002077	Small Cell Lung Cancer	Metastatic	Suspension	LU165	SCLC
ACH-000523	Small Cell Lung Cancer	Metastatic	Mixed	NCIH1184	SCLC
ACH-000399	Small Cell Lung Cancer	Metastatic	Adherent	NCIH2196	SCLC
ACH-000187	Small Cell Lung Cancer	Primary	Suspension	CORL311	SCLC
ACH-000639	Small Cell Lung Cancer	Metastatic	Mixed	NCIH211	SCLC
ACH-002170	Small Cell Lung Cancer	Metastatic	Adherent	NCIH1688	SCLC
ACH-000670	Small Cell Lung Cancer	Metastatic	Adherent	SBC5	SCLC
ACH-000525	Small Cell Lung Cancer	Metastatic	Suspension	NCIH2171	SCLC
ACH-002490	Small Cell Lung Cancer	Metastatic	Adherent	NCIH1882	SCLC
ACH-000506	Small Cell Lung Cancer	Metastatic	Suspension	NCIH146	SCLC
ACH-001386	Small Cell Lung Cancer	Metastatic	Suspension	SCLC22H	SCLC
ACH-000514	Small Cell Lung Cancer	Metastatic	Suspension	NCIH1092	SCLC
ACH-003071	Small Cell Lung Cancer	Primary	Suspension	NCIH748	SCLC
ACH-000803	Small Cell Lung Cancer	Metastatic	Suspension	COLO668	SCLC
ACH-001549	Small Cell Lung Cancer	Primary	Suspension	LU135	SCLC
ACH-003121	Small Cell Lung Cancer	Metastatic	Adherent	NCIH2679	SCLC
ACH-002051	Small Cell Lung Cancer	Primary	Suspension	LU134A	SCLC
ACH-000297	Small Cell Lung Cancer	Metastatic	Suspension	NCIH889	SCLC
ACH-002052	Small Cell Lung Cancer	Primary	Suspension	LU139	SCLC
ACH-000912	Small Cell Lung Cancer	Primary	Adherent	NCIH2286	SCLC
ACH-000508	Small Cell Lung Cancer	Metastatic	Mixed	CORL88	SCLC
ACH-000698	Small Cell Lung Cancer	Primary	Adherent	DMS53	SCLC
ACH-000780	Small Cell Lung Cancer	Metastatic	Suspension	NCIH1105	SCLC
ACH-000790	Small Cell Lung Cancer	Primary	Mixed	SHP77	SCLC
ACH-000870	Small Cell Lung Cancer	Metastatic	Mixed	NCIH1930	SCLC
ACH-000290	Small Cell Lung Cancer	Metastatic	Suspension	NCIH209	SCLC
ACH-000767	Small Cell Lung Cancer	Metastatic	Suspension	NCIH526	SCLC
ACH-000355	Small Cell Lung Cancer	Metastatic	Mixed	NCIH82	SCLC
ACH-000610	Small Cell Lung Cancer	Primary	Mixed	NCIH2227	SCLC
ACH-000515	Small Cell Lung Cancer	Metastatic	Suspension	HCC33	SCLC
ACH-003119	Small Cell Lung Cancer	Metastatic	Adherent	NCIH1450	SCLC
ACH-000743	Small Cell Lung Cancer	Primary	Suspension	CORL95	SCLC
ACH-000257	Small Cell Lung Cancer	Metastatic	Mixed	CORL279	SCLC
ACH-003061	Small Cell Lung Cancer	Primary	Suspension	NCIH774	SCLC
ACH-000431	Small Cell Lung Cancer	Metastatic	Suspension	NCIH1694	SCLC
ACH-000703	Small Cell Lung Cancer	Metastatic	Suspension	DMS79	SCLC
ACH-000298	Small Cell Lung Cancer	Metastatic	Adherent	NCIH2029	SCLC
ACH-000659	Small Cell Lung Cancer	Metastatic	Suspension	SCLC21H	SCLC
ACH-000559	Small Cell Lung Cancer	Primary	Suspension	NCIH1836	SCLC
ACH-000844	Small Cell Lung Cancer	Primary	Adherent	DMS454	SCLC
ACH-001591	Small Cell Lung Cancer	Primary	Adherent	NCIH1417	SCLC
ACH-000382	Small Cell Lung Cancer	Metastatic	Suspension	CORL24	SCLC
ACH-000695	Small Cell Lung Cancer	Metastatic	Suspension	CORL47	SCLC
ACH-000830	Small Cell Lung Cancer	Metastatic	Suspension	NCIH1436	SCLC
ACH-002508	Acral Melanoma	Metastatic	Adherent	WM3211	SKCM
ACH-002846	Acral Melanoma	Metastatic	Suspension	YUSEEPM163517	SKCM
ACH-002928	Acral Melanoma	Metastatic	Unknown	MM100511	SKCM
ACH-002510	Acral Melanoma	Metastatic	Adherent	M040416	SKCM
ACH-002509	Acral Melanoma	Metastatic	Adherent	WM4235	SKCM
ACH-002512	Acral Melanoma	Metastatic	Adherent	MM160113	SKCM
ACH-001339	Cutaneous Melanoma	Primary	Adherent	COLO794	SKCM
ACH-000632	Cutaneous Melanoma	Metastatic	Adherent	HS944T	SKCM
ACH-000805	Cutaneous Melanoma	Metastatic	Adherent	COLO679	SKCM
ACH-002005	Cutaneous Melanoma	Primary	Adherent	SKMEL19	SKCM
ACH-000404	Cutaneous Melanoma	Primary	Adherent	K029AX	SKCM
ACH-000730	Cutaneous Melanoma	Metastatic	Adherent	SKMEL5	SKCM
ACH-000810	Cutaneous Melanoma	Metastatic	Adherent	SKMEL30	SKCM
ACH-000423	Cutaneous Melanoma	Metastatic	Adherent	SKMEL3	SKCM
ACH-000915	Cutaneous Melanoma	Primary	Adherent	IPC298	SKCM
ACH-000881	Cutaneous Melanoma	Primary	Adherent	MELJUSO	SKCM
ACH-000401	Cutaneous Melanoma	Metastatic	Adherent	COLO800	SKCM
ACH-000615	Cutaneous Melanoma	Primary	Adherent	SKMEL28	SKCM
ACH-000987	Cutaneous Melanoma	Metastatic	Adherent	MEWO	SKCM
ACH-000644	Cutaneous Melanoma	Primary	Adherent	COLO829	SKCM
ACH-000640	Cutaneous Melanoma	Primary	Adherent	SKMEL31	SKCM
ACH-001328	Cutaneous Squamous Cell Carcinoma	Primary	Adherent	A431	SKCM
ACH-001521	Cutaneous Squamous Cell Carcinoma	Primary	Adherent	HKA1	SKCM
ACH-001523	Cutaneous Squamous Cell Carcinoma	Primary	Adherent	HSC1	SKCM
ACH-001524	Cutaneous Squamous Cell Carcinoma	Primary	Adherent	HSC5	SKCM
ACH-001442	Cutaneous Squamous Cell Carcinoma	Metastatic	Adherent	A388	SKCM
ACH-001979	Melanoma	Metastatic	Adherent	NZM11	SKCM
ACH-000765	Melanoma	Metastatic	Adherent	WM983B	SKCM
ACH-000008	Melanoma	Primary	Adherent	A101D	SKCM
ACH-000465	Melanoma	Metastatic	Suspension	SKMEL1	SKCM

ACH-000550	Melanoma	Primary	Adherent	IGR39	SKCM
ACH-000322	Melanoma	Metastatic	Adherent	HT144	SKCM
ACH-000441	Melanoma	Metastatic	Adherent	SH4	SKCM
ACH-001981	Melanoma	Metastatic	Adherent	NZM2	SKCM
ACH-001975	Melanoma	Metastatic	Adherent	MM576	SKCM
ACH-001704	Melanoma	Primary	Adherent	VMRCMELG	SKCM
ACH-000580	Melanoma	Primary	Adherent	C32	SKCM
ACH-001568	Melanoma	Metastatic	Adherent	MM386	SKCM
ACH-002847	Melanoma	Metastatic	Adherent	YUHOIN	SKCM
ACH-002458	Melanoma	Metastatic	Adherent	HT144SKINFV1	SKCM
ACH-001569	Melanoma	Metastatic	Adherent	MM415	SKCM
ACH-000814	Melanoma	Primary	Adherent	HS939T	SKCM
ACH-001973	Melanoma	Metastatic	Adherent	MM485	SKCM
ACH-001949	Melanoma		Adherent	MAMEL46	SKCM
ACH-001980	Melanoma	Metastatic	Adherent	NZM12	SKCM
ACH-003456	Melanoma	Metastatic	Adherent	CCLFMELM0006T	SKCM
ACH-002003	Melanoma	Primary	Adherent	A375SKINCJ3	SKCM
ACH-002461	Melanoma	Metastatic	Adherent	RVH421SKINFV1	SKCM
ACH-001041	Melanoma	Metastatic	Adherent	CHL1DM	SKCM
ACH-001988	Melanoma	Metastatic	Mixed	NZM5	SKCM
ACH-000799	Melanoma	Metastatic	Adherent	HS695T	SKCM
ACH-001983	Melanoma	Metastatic	Adherent	NZM30	SKCM
ACH-002511	Melanoma	Primary	Adherent	M140325	SKCM
ACH-003455	Melanoma	Primary	Adherent	CCLFMELM0004T	SKCM
ACH-001990	Melanoma	Metastatic	Adherent	NZM7	SKCM
ACH-003458	Melanoma	Metastatic	Adherent	CCLFMELM0010T	SKCM
ACH-001985	Melanoma	Metastatic	Mixed	NZM40	SKCM
ACH-002460	Melanoma	Metastatic	Adherent	HT144SKINFV2	SKCM
ACH-000614	Melanoma	Metastatic	Adherent	RVH421	SKCM
ACH-000014	Melanoma	Metastatic	Adherent	HS294T	SKCM
ACH-000348	Melanoma	Metastatic	Adherent	RPMI7951	SKCM
ACH-001566	Melanoma	Metastatic	Adherent	MM370	SKCM
ACH-000572	Melanoma	Primary	Adherent	G361	SKCM
ACH-001239	Melanoma	Metastatic	Adherent	WM2664	SKCM
ACH-001970	Melanoma	Metastatic	Adherent	MM253	SKCM
ACH-000425	Melanoma	Primary	Adherent	UACC62	SKCM
ACH-000882	Melanoma	Metastatic	Adherent	IGR1	SKCM
ACH-000458	Melanoma	Metastatic	Adherent	CJM	SKCM
ACH-002084	Melanoma	Metastatic	Adherent	MMAC	SKCM
ACH-001987	Melanoma	Metastatic	Adherent	NZM43	SKCM
ACH-001986	Melanoma	Metastatic	Adherent	NZM42	SKCM
ACH-001522	Melanoma	Metastatic	Adherent	HMY1	SKCM
ACH-000650	Melanoma	Metastatic	Adherent	IGR37	SKCM
ACH-002404	Melanoma	Metastatic	Adherent	CCLFMELM0002T	SKCM
ACH-001971	Melanoma	Metastatic	Adherent	MM466	SKCM
ACH-001978	Melanoma	Metastatic	Adherent	NZM1	SKCM
ACH-001984	Melanoma	Metastatic	Mixed	NZM4	SKCM
ACH-001563	Melanoma	Metastatic	Adherent	MM127	SKCM
ACH-000822	Melanoma	Metastatic	Adherent	SKMEL24	SKCM
ACH-000812	Melanoma	Metastatic	Adherent	COLO783	SKCM
ACH-001982	Melanoma	Metastatic	Adherent	NZM3	SKCM
ACH-001567	Melanoma	Metastatic	Adherent	MM383	SKCM
ACH-001976	Melanoma	Metastatic	Adherent	MM603	SKCM
ACH-000661	Melanoma	Primary	Adherent	WM1799	SKCM
ACH-001190	Melanoma	Metastatic	Adherent	SKMEL2	SKCM
ACH-002459	Melanoma	Metastatic	Adherent	HT144SKINFV3	SKCM
ACH-001645	Melanoma	Primary	Suspension	SEKI	SKCM
ACH-000304	Melanoma	Metastatic	Adherent	WM115	SKCM
ACH-000931	Melanoma	Primary	Adherent	HMCB	SKCM
ACH-001972	Melanoma	Metastatic	Adherent	MM473	SKCM
ACH-001953	Melanoma		Adherent	MAMEL27	SKCM
ACH-003105	Melanoma		Adherent	VMM917	SKCM
ACH-002001	Melanoma	Primary	Adherent	A375SKINCJ1	SKCM
ACH-000450	Melanoma	Primary	Adherent	MELHO	SKCM
ACH-000899	Melanoma	Metastatic	Unknown	WM88	SKCM
ACH-000884	Melanoma	Metastatic	Adherent	MDAMB435S	SKCM
ACH-003459	Melanoma	Metastatic	Adherent	CCLFMELM0011T	SKCM
ACH-001989	Melanoma	Metastatic	Mixed	NZM6	SKCM
ACH-000477	Melanoma	Metastatic	Adherent	MALME3M	SKCM
ACH-000801	Melanoma	Primary	Adherent	HS936T	SKCM
ACH-002004	Melanoma	Primary	Adherent	UACC62SKINCJ1	SKCM
ACH-000827	Melanoma	Primary	Adherent	WM793	SKCM
ACH-000274	Melanoma	Primary	Adherent	HS852T	SKCM
ACH-000219	Melanoma	Primary	Adherent	A375	SKCM

ACH-000582	Melanoma	Metastatic	Adherent	COLO741	SKCM
ACH-001570	Melanoma	Metastatic	Adherent	MM426	SKCM
ACH-002403	Melanoma	Metastatic	Adherent	CCLFMELM0001T	SKCM
ACH-003457	Melanoma	Metastatic	Adherent	CCLFMELM0038T	SKCM
ACH-002002	Melanoma	Primary	Adherent	A375SKINCJ2	SKCM
ACH-000579	Melanoma	Primary	Adherent	UACC257	SKCM
ACH-000788	Melanoma	Metastatic	Adherent	A2058	SKCM
ACH-002405	Melanoma	Metastatic	Adherent	CCLFMELM0003T	SKCM
ACH-001974	Melanoma	Metastatic	Adherent	MM537	SKCM
ACH-000968	Melanoma	Metastatic	Adherent	COLO792	SKCM
ACH-000750	Melanoma	Metastatic	Adherent	LOXIMVI	SKCM
ACH-000530	SMARCA4-deficient undifferentiated tumor	Primary	Adherent	DMS114	SMARCA4-UT
ACH-000890	SMARCA4-deficient undifferentiated tumor	Primary	Adherent	SW1271	SMARCA4-UT
ACH-000129	SMARCA4-deficient undifferentiated tumor	Metastatic	Suspension	NCIH1341	SMARCA4-UT
ACH-000292	SMARCA4-deficient undifferentiated tumor	Metastatic	Adherent	NCIH841	SMARCA4-UT
ACH-000752	SMARCA4-deficient undifferentiated tumor	Metastatic	Adherent	NCIH196	SMARCA4-UT
ACH-000351	Adenosquamous Carcinoma of the Stomach	Metastatic	Adherent	MKN1	STAD
ACH-000633	Diffuse Type Stomach Adenocarcinoma	Primary	Adherent	FU97	STAD
ACH-000344	Diffuse Type Stomach Adenocarcinoma	Metastatic	Adherent	SNU668	STAD
ACH-000674	Diffuse Type Stomach Adenocarcinoma	Metastatic	Mixed	NUGC4	STAD
ACH-000247	Diffuse Type Stomach Adenocarcinoma	Metastatic	Mixed	OCUM1	STAD
ACH-000356	Diffuse Type Stomach Adenocarcinoma	Metastatic	Adherent	MKN45	STAD
ACH-000764	Mucinous Stomach Adenocarcinoma	Primary	Adherent	SH10TC	STAD
ACH-000736	Signet Ring Cell Carcinoma of the Stomach	Metastatic	Adherent	SNU601	STAD
ACH-000793	Signet Ring Cell Carcinoma of the Stomach	Metastatic	Mixed	KATOIII	STAD
ACH-000880	Stomach Adenocarcinoma	Primary	Adherent	AGS	STAD
ACH-000847	Stomach Adenocarcinoma	Metastatic	Adherent	HGC27	STAD
ACH-000485	Stomach Adenocarcinoma	Metastatic	Mixed	GSU	STAD
ACH-000949	Stomach Adenocarcinoma	Metastatic	Suspension	TGBC11TKB	STAD
ACH-000507	Stomach Adenocarcinoma	Metastatic	Adherent	KE39	STAD
ACH-000908	Stomach Adenocarcinoma	Primary	Adherent	SNU520	STAD
ACH-000303	Stomach Adenocarcinoma	Metastatic	Suspension	SNU5	STAD
ACH-001664	Stomach Adenocarcinoma	Metastatic	Adherent	SNU638	STAD
ACH-000616	Stomach Adenocarcinoma	Metastatic	Adherent	HS746T	STAD
ACH-000761	Stomach Adenocarcinoma	Metastatic	Adherent	NUGC2	STAD
ACH-001653	Stomach Adenocarcinoma	Primary	Adherent	SKGT2	STAD
ACH-000932	Stomach Adenocarcinoma	Metastatic	Suspension	SNU1	STAD
ACH-002446	Stomach Adenocarcinoma	Metastatic	Adherent	CCLFUPGI0005T	STAD
ACH-000110	Stomach Adenocarcinoma	Primary	Suspension	NCCSTCK140	STAD
ACH-000144	Stomach Adenocarcinoma	Metastatic	Adherent	RERFGC1B	STAD
ACH-000746	Stomach Adenocarcinoma	Metastatic	Suspension	GSS	STAD
ACH-000255	Stomach Adenocarcinoma	Metastatic	Adherent	LMSU	STAD
ACH-000948	Stomach Adenocarcinoma	Primary	Adherent	2313287	STAD
ACH-000325	Stomach Adenocarcinoma	Metastatic	Suspension	SNU620	STAD
ACH-000919	Stomach Adenocarcinoma	Primary	Mixed	IM95	STAD
ACH-000581	Stomach Adenocarcinoma	Metastatic	Suspension	SNU16	STAD
ACH-000911	Stomach Adenocarcinoma	Metastatic	Adherent	NUGC3	STAD
ACH-003437	Stomach Adenocarcinoma	Metastatic	Adherent	CCLFUPGI0078T	STAD
ACH-000678	Tubular Stomach Adenocarcinoma	Metastatic	Adherent	MKN7	STAD
ACH-002544	Tubular Stomach Adenocarcinoma	Primary	Adherent	SNU1750	STAD
ACH-000898	Tubular Stomach Adenocarcinoma	Primary	Mixed	SNU719	STAD
ACH-000758	Tubular Stomach Adenocarcinoma	Metastatic	Adherent	MKN74	STAD
ACH-000239	Tubular Stomach Adenocarcinoma	Metastatic	Mixed	HUG1N	STAD
ACH-000427	Tubular Stomach Adenocarcinoma	Metastatic	Adherent	NCIN87	STAD
ACH-000466	Tubular Stomach Adenocarcinoma	Metastatic	Adherent	SNU216	STAD
ACH-001671	Embryonal Carcinoma	Metastatic	Adherent	TERA1	TGCT
ACH-001672	Embryonal Carcinoma	Metastatic	Adherent	TERA2	TGCT
ACH-001438	Embryonal Carcinoma	Metastatic	Adherent	1777NRPMET	TGCT
ACH-001437	Embryonal Carcinoma	Primary	Adherent	1618K	TGCT
ACH-002291	Embryonal Carcinoma	Metastatic	Adherent	NTERA2CLD1	TGCT
ACH-001435	Embryonal Carcinoma	Primary	Adherent	1156QE8	TGCT
ACH-001440	Embryonal Carcinoma	Primary	Adherent	833KE	TGCT
ACH-001578	Embryonal Carcinoma	Primary	Adherent	NCCIT	TGCT
ACH-001502	Teratoma	Primary	Adherent	GCT27	TGCT
ACH-001668	Teratoma	Primary	Adherent	SUSA	TGCT
ACH-003469	Anaplastic Thyroid Cancer	Metastatic	Adherent	CCLFTHYR0004T	THCA
ACH-003468	Anaplastic Thyroid Cancer	Metastatic	Adherent	CCLFTHYR0003T	THCA
ACH-001350	Anaplastic Thyroid Cancer	Metastatic	Adherent	HTCC3	THCA
ACH-001443	Anaplastic Thyroid Cancer	Primary	Adherent	ASH3	THCA
ACH-001307	Anaplastic Thyroid Cancer	Primary	Adherent	8505C	THCA
ACH-001306	Anaplastic Thyroid Cancer	Primary	Adherent	8305C	THCA
ACH-003470	Anaplastic Thyroid Cancer	Metastatic	Adherent	CCLFTHYR0005T	THCA
ACH-000191	Anaplastic Thyroid Cancer	Metastatic	Adherent	BHT101	THCA
ACH-000174	Anaplastic Thyroid Cancer	Primary	Adherent	CAL62	THCA

ACH-002041	Anaplastic Thyroid Cancer	Primary	Adherent	HOTHC	THCA
ACH-001356	Anaplastic Thyroid Cancer	Primary	Adherent	MB1	THCA
ACH-000897	Follicular Thyroid Cancer	Metastatic	Adherent	FTC238	THCA
ACH-000716	Follicular Thyroid Cancer	Primary	Adherent	TT2609C02	THCA
ACH-000903	Follicular Thyroid Cancer	Metastatic	Adherent	FTC133	THCA
ACH-000058	Follicular Thyroid Cancer	Primary	Adherent	ML1	THCA
ACH-001384	Follicular Thyroid Cancer	Metastatic	Adherent	RO82W1	THCA
ACH-001321	Medullary Thyroid Cancer	Primary	Adherent	TT	THCA
ACH-003472	Medullary Thyroid Cancer	Metastatic	Adherent	CCLFTHYR0009T	THCA
ACH-001528	Papillary Thyroid Cancer	Metastatic	Adherent	IHH4	THCA
ACH-002440	Papillary Thyroid Cancer	Metastatic	Adherent	CCLFTHYR0008T	THCA
ACH-003471	Papillary Thyroid Cancer	Metastatic	Adherent	CCLFTHYR0006T	THCA
ACH-000456	Poorly Differentiated Thyroid Cancer	Primary	Adherent	BCPAP	THCA
ACH-002021	Choriocarcinoma	Primary	Adherent	T3M3	UCEC
ACH-001530	Choriocarcinoma	Metastatic	Adherent	JEG3	UCEC
ACH-001529	Choriocarcinoma	Primary	Adherent	JAR	UCEC
ACH-000941	Endometrial Carcinoma	Primary	Adherent	HEC1B	UCEC
ACH-000946	Endometrial Carcinoma	Primary	Adherent	HEC265	UCEC
ACH-000978	Endometrial Carcinoma	Primary	Adherent	EN	UCEC
ACH-001517	Endometrial Carcinoma	Primary	Adherent	HEC1	UCEC
ACH-000993	Endometrial Carcinoma	Primary	Adherent	JHUEM7	UCEC
ACH-000293	Endometrial Carcinoma	Primary	Adherent	KLE	UCEC
ACH-000954	Endometrial Carcinoma	Primary	Adherent	HEC1A	UCEC
ACH-000864	Endometrial Carcinoma	Primary	Suspension	COLO684	UCEC
ACH-000928	Endometrial Carcinoma	Primary	Adherent	JHUEM1	UCEC
ACH-002027	Endometrial Carcinoma	Primary	Adherent	HOUAI	UCEC
ACH-001518	Endometrial Carcinoma	Primary	Adherent	HEC116	UCEC
ACH-000831	Endometrial Carcinoma	Metastatic	Adherent	HEC50B	UCEC
ACH-000990	Endometrial Carcinoma	Primary	Adherent	HEC108	UCEC
ACH-000974	Endometrial Carcinoma	Metastatic	Adherent	SNGM	UCEC
ACH-000173	Endometrial Carcinoma	Primary	Adherent	JHUEM3	UCEC
ACH-000994	Endometrial Carcinoma	Primary	Adherent	HEC59	UCEC
ACH-000435	Endometrial Carcinoma	Metastatic	Adherent	EFE184	UCEC
ACH-002026	Endometrial Carcinoma	Primary	Adherent	HHUA	UCEC
ACH-000879	Endometrial Carcinoma	Primary	Adherent	MFE296	UCEC
ACH-000192	Endometrial Carcinoma	Primary	Adherent	MFE280	UCEC
ACH-000940	Endometrial Carcinoma	Metastatic	Adherent	AN3CA	UCEC
ACH-000961	Endometrial Carcinoma	Primary	Unknown	ISHIKAWAHERAKLIO02ER	UCEC
ACH-000909	Endometrial Carcinoma	Primary	Mixed	JHUEM2	UCEC
ACH-000972	Endometrial Carcinoma	Primary	Adherent	HEC151	UCEC
ACH-000996	Endometrial Carcinoma	Primary	Adherent	HEC251	UCEC
ACH-000407	ne Carcinosarcoma/Uterine Malignant Mixed Mullerian T	Primary	Adherent	SNU685	UCS
ACH-001495	ne Carcinosarcoma/Uterine Malignant Mixed Mullerian T	Primary	Adherent	EMTOKA	UCS
ACH-002046	ne Carcinosarcoma/Uterine Malignant Mixed Mullerian T	Primary	Adherent	HTMMT	UCS
ACH-002419	ne Carcinosarcoma/Uterine Malignant Mixed Mullerian T	Primary	Suspension	CCLFOVPA0001T	UCS
ACH-000302	ne Carcinosarcoma/Uterine Malignant Mixed Mullerian T	Primary	Adherent	SNU1077	UCS
ACH-002648	ne Carcinosarcoma/Uterine Malignant Mixed Mullerian Tumor		Adherent	CX03	UCS
ACH-002047	ne Carcinosarcoma/Uterine Malignant Mixed Mullerian T	Metastatic	Adherent	HIRSBM	UCS
ACH-002687	Uveal Melanoma	Metastatic	Adherent	WM3772F	UVM
ACH-001554	Uveal Melanoma	Primary	Adherent	MEL202	UVM
ACH-001441	Uveal Melanoma	Metastatic	Adherent	921	UVM
ACH-002018	Uveal Melanoma	Metastatic	Adherent	OMM25	UVM
ACH-002013	Uveal Melanoma	Metastatic	Adherent	MM28	UVM
ACH-002017	Uveal Melanoma	Metastatic	Adherent	OMM1	UVM
ACH-002925	Uveal Melanoma	Primary	Adherent	UPMM3	UVM
ACH-002927	Uveal Melanoma	Primary	Adherent	UPMD2	UVM
ACH-002924	Uveal Melanoma	Primary	Adherent	UPMM2	UVM
ACH-002926	Uveal Melanoma	Primary	Unknown	UPMD1	UVM
ACH-002016	Uveal Melanoma	Primary	Adherent	MEL290	UVM
ACH-002015	Uveal Melanoma	Primary	Adherent	MEL285	UVM
ACH-002010	Uveal Melanoma	Primary	Adherent	MP41	UVM
ACH-002014	Uveal Melanoma	Primary	Adherent	MEL270	UVM
ACH-002011	Uveal Melanoma	Primary	Adherent	MP46	UVM

Supplementary Table S2. Status of common or selective subunit based on the CRISPR effect

Subunit	Number of Strongly Lethal Cell Lines*	Total Cell Lines	% Strongly Lethal Cell Lines
PSMA3	1178	1178	100
PSMA6	1178	1178	100
PSMB2	1178	1178	100
PSMB3	1178	1178	100
PSMB4	1178	1178	100
PSMA2	1177	1178	99.92
PSMD6	1177	1178	99.92
PSMC6	1175	1178	99.75
PSMD12	1175	1178	99.75
PSMA1	1174	1178	99.66
PSMA5	1173	1178	99.58
PSMB1	1171	1178	99.41
PSMD11	1171	1178	99.41
PSMD3	1168	1178	99.15
PSMC3	1164	1178	98.81
PSMA4	1157	1178	98.22
PSMC4	1151	1178	97.71
PSMC2	1140	1178	96.77
PSMD14	1120	1178	95.08
PSMD2	1104	1178	93.72
PSMD7	1085	1178	92.11
PSMC1	1082	1178	91.85
PSMC5	1081	1178	91.77
PSMD4	1056	1178	89.64
PSMB6	1045	1178	88.71
PSMB7	1031	1178	87.52
PSMB5	1025	1178	87.01
PSMD1	1013	1178	85.99
PSMG4	900	1178	76.4
PSMG3	819	1178	69.52
PSMD13	565	1178	47.96
PSMD8	339	1178	28.78
PSMG2	214	1178	18.17
PSMG1	120	1178	10.19
PSMD10	5	1178	0.42
PSME3	5	1178	0.42
PSMF1	5	1178	0.42
PSMD9	1	1178	0.08
PSMA8	0	1178	0
PSMB10	0	1178	0
PSMB11	0	1178	0
PSMB8	0	1178	0
PSMB9	0	1178	0
PSMC3IP	0	1178	0
PSMD5	0	1178	0
PSME1	0	1178	0
PSME2	0	1178	0
PSME3IP1	0	1178	0
PSME4	0	1178	0

* "Strongly Lethal Cell Lines" indicates "Chronos Score < -1"

Supplementary Table S3. Status of common or selective subunit based on the RNAi effect

Subunit	Number of Strongly Lethal Cell Lines*	Total Cell Lines	% Strongly Lethal Cell Lines
PSMD1	533	542	98.34
PSMA3	632	707	89.39
PSMB2	471	542	86.9
PSMC5	608	705	86.24
PSMD7	457	542	84.32
PSMA4	589	705	83.55
PSMB6	560	705	79.43
PSMC2	542	703	77.1
PSMC3	538	703	76.53
PSMD11	393	542	72.51
PSMB5	441	703	62.73
PSMC6	426	703	60.6
PSMA1	322	542	59.41
PSMD12	408	705	57.87
PSMA6	406	705	57.59
PSMC1	395	705	56.03
PSMB3	269	542	49.63
PSMD2	295	705	41.84
PSMC4	286	705	40.57
PSMA2	177	542	32.66
PSMB4	100	542	18.45
PSMD14	121	703	17.21
PSMB1	50	542	9.23
PSMD13	37	542	6.83
PSMD4	30	703	4.27
PSMB7	22	542	4.06
PSMA5	18	542	3.32
PSMG1	11	339	3.24
PSMD3	11	542	2.03
PSMD8	8	542	1.48
PSMG2	4	339	1.18
PSMD10	2	705	0.28
PSME3	0	542	0
PSMA8	0	705	0
PSME4	0	339	0
PSMC3IP	0	596	0
PSMB8	0	705	0
PSMB9	0	705	0
PSMB10	0	542	0
PSMD5	0	542	0
PSMD9	0	542	0
PSME1	0	542	0
PSME2	0	542	0
PSME3IP1	0	339	0
PSMG3	0	339	0

* "Strongly Lethal Cell Lines" indicates "DEMETER Score < -1"

Supplementary Table S4. CRISPR-Immune Feature Correlations

Positively correlated immune-related genes (n = 56)					
Subunit	Feature gene	Pearson r	P value	Q value	Feature type
**PSMB5**	NCF4	0.417	3.05×10^{-49}	1.95×10^{-45}	Expression
**PSMB5**	IL12RB1	0.411	1.13×10^{-47}	4.35×10^{-44}	Expression
**PSMB5**	NLRC5	0.408	5.28×10^{-47}	1.69×10^{-43}	Expression
**PSMB5**	TAP1	0.390	8.39×10^{-43}	1.24×10^{-39}	Expression
**PSMB5**	CD53	0.384	2.26×10^{-41}	2.42×10^{-38}	Expression
**PSMB5**	HLA-B	0.373	6.51×10^{-39}	5.44×10^{-36}	Expression
**PSMB5**	PLEK	0.369	5.09×10^{-38}	3.35×10^{-35}	Expression
**PSMB5**	CD74	0.366	1.49×10^{-37}	8.17×10^{-35}	Expression
**PSMB5**	CD48	0.361	1.87×10^{-36}	8.75×10^{-34}	Expression
**PSMB5**	IL2RG	0.360	3.32×10^{-36}	1.52×10^{-33}	Expression
**PSMB5**	LY86	0.355	4.05×10^{-35}	1.66×10^{-32}	Expression
**PSMB6**	NLRC5	0.354	5.27×10^{-35}	5.06×10^{-31}	Expression
**PSMB5**	ITGAL	0.353	7.98×10^{-35}	3.01×10^{-32}	Expression
**PSMB5**	TRIM38	0.351	2.34×10^{-34}	8.31×10^{-32}	Expression
**PSMB7**	CD48	0.351	2.56×10^{-34}	3.51×10^{-31}	Expression
**PSMB5**	TRIM22	0.351	2.56×10^{-34}	8.95×10^{-32}	Expression
**PSMB5**	TRIM21	0.351	2.71×10^{-34}	9.30×10^{-32}	Expression
**PSMB5**	NCF1	0.347	1.63×10^{-33}	5.04×10^{-31}	Expression
**PSMB5**	CD86	0.344	6.00×10^{-33}	1.72×10^{-30}	Expression
**PSMB5**	SPI1	0.343	8.93×10^{-33}	2.49×10^{-30}	Expression
**PSMB5**	PTPRC	0.342	1.40×10^{-32}	3.78×10^{-30}	Expression
**PSMB5**	BTK	0.337	1.32×10^{-31}	3.24×10^{-29}	Expression
**PSMB6**	TAP1	0.336	1.46×10^{-31}	9.33×10^{-28}	Expression
**PSMB5**	OAS2	0.334	5.01×10^{-31}	1.09×10^{-28}	Expression
**PSMB5**	HLA-DRA	0.332	1.10×10^{-30}	2.29×10^{-28}	Expression
**PSMB5**	HLA-E	0.331	1.56×10^{-30}	3.15×10^{-28}	Expression
**PSMB5**	SP110	0.330	2.62×10^{-30}	5.19×10^{-28}	Expression
**PSMB7**	CD53	0.327	8.59×10^{-30}	2.58×10^{-27}	Expression
**PSMB5**	HLA-F	0.326	1.20×10^{-29}	2.24×10^{-27}	Expression
**PSMB5**	IRF2	0.326	1.22×10^{-29}	2.26×10^{-27}	Expression
**PSMB5**	TAGAP	0.326	1.40×10^{-29}	2.56×10^{-27}	Expression
**PSMB5**	LAIR1	0.325	1.59×10^{-29}	2.85×10^{-27}	Expression
**PSMB5**	HLA-DMA	0.324	3.04×10^{-29}	5.25×10^{-27}	Expression
**PSMB5**	TNF	0.324	3.50×10^{-29}	6.00×10^{-27}	Expression
**PSMB5**	JAK3	0.321	8.24×10^{-29}	1.39×10^{-26}	Expression
**PSMB5**	HLA-DPA1	0.319	1.90×10^{-28}	3.06×10^{-26}	Expression
**PSMB5**	CIITA	0.319	2.02×10^{-28}	3.21×10^{-26}	Expression
**PSMB5**	IRF1	0.319	2.32×10^{-28}	3.66×10^{-26}	Expression
**PSMB5**	HLA-DOB	0.319	2.61×10^{-28}	4.04×10^{-26}	Expression
**PSMB5**	ERAP1	0.318	2.85×10^{-28}	4.38×10^{-26}	Expression
**PSMB5**	CCL5	0.318	3.59×10^{-28}	5.42×10^{-26}	Expression
**PSMB7**	CD79A	0.315	1.05×10^{-27}	1.98×10^{-25}	Expression
**PSMB7**	CD19	0.314	2.02×10^{-27}	3.50×10^{-25}	Expression
**PSMB5**	LILRB1	0.312	3.80×10^{-27}	5.29×10^{-25}	Expression
**PSMB5**	PLCG2	0.312	4.06×10^{-27}	5.60×10^{-25}	Expression
**PSMB5**	LST1	0.311	4.72×10^{-27}	6.48×10^{-25}	Expression
**PSMB7**	TNFRSF13C	0.311	5.12×10^{-27}	8.20×10^{-25}	Expression
**PSMB5**	TNFSF14	0.310	8.91×10^{-27}	1.20×10^{-24}	Expression
**PSMB7**	IL2RG	0.309	1.07×10^{-26}	1.55×10^{-24}	Expression
**PSMB6**	IL12RB1	0.309	1.45×10^{-26}	5.56×10^{-23}	Expression
**PSMB6**	HLA-DRA	0.202	6.50×10^{-12}	9.39×10^{-10}	Expression
**PSMD6**	DOK2	0.201	2.86×10^{-9}	2.95×10^{-5}	Copy Number
**PSMB5**	HLA-DQA1	0.201	8.04×10^{-12}	1.55×10^{-10}	Expression
**PSMB6**	IFI16	0.201	8.10×10^{-12}	1.12×10^{-9}	Expression
**PSMG1**	LY86	0.201	3.10×10^{-9}	1.92×10^{-5}	Copy Number
**PSMB6**	BATF3	0.200	8.55×10^{-12}	1.16×10^{-9}	Expression

Negatively correlated immune-related genes (n = 44)					
Subunit	Feature gene	Pearson r	P value	Q value	Feature type
**PSMB7**	TNFRSF12A	−0.392	4.55×10^{-43}	4.37×10^{-39}	Expression
**PSMB7**	CD63	−0.348	7.00×10^{-34}	7.91×10^{-31}	Expression
**PSMB7**	CD276	−0.340	3.31×10^{-32}	2.17×10^{-29}	Expression
**PSMB7**	CD59	−0.336	1.48×10^{-31}	7.67×10^{-29}	Expression
**PSMB7**	CD151	−0.328	6.05×10^{-30}	1.91×10^{-27}	Expression
**PSMB7**	IL13RA1	−0.323	4.67×10^{-29}	1.14×10^{-26}	Expression
**PSMB7**	CD109	−0.311	6.59×10^{-27}	1.03×10^{-24}	Expression
**PSMD9**	IL31	−0.308	2.39×10^{-20}	2.12×10^{-17}	Copy Number
**PSMB7**	NT5E	−0.304	7.71×10^{-26}	9.49×10^{-24}	Expression
**PSMB7**	TNFRSF1A	−0.304	1.00×10^{-25}	1.21×10^{-23}	Expression
**PSMB5**	CD276	−0.297	1.38×10^{-24}	1.36×10^{-22}	Expression
**PSMB7**	KIRREL1	−0.286	6.91×10^{-23}	5.45×10^{-21}	Expression
**PSMB7**	IL17RC	−0.277	1.38×10^{-21}	8.61×10^{-20}	Expression
**PSMB7**	SOCS3	−0.273	6.60×10^{-21}	3.71×10^{-19}	Expression
**PSMB7**	IFITM3	−0.272	7.55×10^{-21}	4.18×10^{-19}	Expression
**PSMB11**	IRF9	−0.266	2.14×10^{-15}	1.17×10^{-12}	Copy Number
**PSMB7**	CD44	−0.262	2.63×10^{-19}	1.18×10^{-17}	Expression
**PSMB11**	IL25	−0.257	1.85×10^{-14}	5.14×10^{-12}	Copy Number
**PSMB3**	CD63	−0.257	1.07×10^{-18}	1.03×10^{-14}	Expression
**PSMB7**	CD68	−0.255	1.95×10^{-18}	7.70×10^{-17}	Expression
**PSMB7**	IL6ST	−0.255	2.36×10^{-18}	9.16×10^{-17}	Expression
**PSMB11**	CTSG	−0.252	6.51×10^{-14}	1.44×10^{-11}	Copy Number
**PSMB7**	IL11	−0.251	7.45×10^{-18}	2.67×10^{-16}	Expression
**PSMB11**	GZMH	−0.246	2.74×10^{-13}	5.43×10^{-11}	Copy Number
**PSMB5**	CMTM4	−0.246	3.70×10^{-17}	1.53×10^{-15}	Expression
**PSMB7**	TNFSF12	−0.245	4.27×10^{-17}	1.37×10^{-15}	Expression
**PSMB11**	GZMB	−0.240	1.10×10^{-12}	2.02×10^{-10}	Copy Number
**PSMB6**	CMTM4	−0.239	2.69×10^{-16}	1.45×10^{-13}	Expression
**PSMB3**	CD151	−0.236	7.26×10^{-16}	3.49×10^{-12}	Expression
**PSMD10**	IGHG1	−0.234	1.94×10^{-16}	3.31×10^{-12}	Damaging Mutation
**PSMB7**	C1R	−0.232	2.05×10^{-15}	5.01×10^{-14}	Expression
**PSMB7**	CXCL8	−0.232	2.19×10^{-15}	5.33×10^{-14}	Expression
**PSMB7**	TGFB2	−0.230	3.82×10^{-15}	9.04×10^{-14}	Expression
**PSMB7**	C1S	−0.227	9.35×10^{-15}	2.07×10^{-13}	Expression
**PSMB3**	TNFRSF12A	−0.225	1.46×10^{-14}	2.54×10^{-11}	Expression
**PSMB7**	ULBP2	−0.221	4.08×10^{-14}	8.02×10^{-13}	Expression
**PSMA3**	CD59	−0.220	5.16×10^{-14}	4.14×10^{-10}	Expression
**PSMB5**	IL17RD	−0.220	5.90×10^{-14}	1.56×10^{-12}	Expression
**PSMA3**	CD63	−0.220	6.47×10^{-14}	4.14×10^{-10}	Expression
**PSMB5**	IL17RC	−0.218	1.00×10^{-13}	2.58×10^{-12}	Expression
**PSMC4**	HLA-G	−0.211	1.23×10^{-13}	2.62×10^{-10}	Damaging Mutation
**PSMB3**	CD59	−0.206	2.14×10^{-12}	9.70×10^{-10}	Expression
**PSMB6**	CMTM8	−0.205	3.05×10^{-12}	5.04×10^{-10}	Expression
**PSME3**	SIRPA	−0.200	8.70×10^{-12}	4.78×10^{-9}	Expression

Supplementary Table S5. RNAi-Immune Feature Correlations

Positively correlated immune-related genes (n = 59)					
**Subunit**	**Feature gene**	**Pearson r**	**P value**	**Q value**	**Feature type**
**PSMC1**	BCL11B	0.459	2.55×10^{-28}	6.31×10^{-26}	Copy Number
**PSMC2**	TRIM56	0.454	1.53×10^{-27}	6.32×10^{-25}	Copy Number
**PSMD6**	TLR9	0.443	7.00×10^{-13}	1.89×10^{-10}	Copy Number
**PSMC1**	TRAF3	0.433	4.50×10^{-25}	6.98×10^{-23}	Copy Number
**SEM1**	TRIM56	0.430	9.92×10^{-25}	2.22×10^{-22}	Copy Number
**SEM1**	CD36	0.430	1.21×10^{-24}	2.61×10^{-22}	Copy Number
**PSMD6**	CCR3	0.428	4.87×10^{-12}	7.13×10^{-10}	Copy Number
**PSMD6**	CCR1	0.424	7.85×10^{-12}	9.94×10^{-10}	Copy Number
**SEM1**	CCL24	0.423	6.49×10^{-24}	1.14×10^{-21}	Copy Number
**SEM1**	CCL26	0.423	6.70×10^{-24}	1.17×10^{-21}	Copy Number
**PSMD6**	XCR1	0.414	2.61×10^{-11}	2.59×10^{-9}	Copy Number
**PSMB1**	CCR6	0.410	1.41×10^{-17}	5.96×10^{-15}	Copy Number
**PSMD6**	CCR9	0.409	4.54×10^{-11}	4.22×10^{-9}	Copy Number
**PSMD7**	PLCG2	0.409	1.80×10^{-17}	3.98×10^{-15}	Copy Number
**PSMD11**	CCL11	0.407	2.60×10^{-17}	6.05×10^{-14}	Copy Number
**PSMB1**	ULBP2	0.406	3.32×10^{-17}	1.29×10^{-14}	Copy Number
**PSMD11**	CCL7	0.406	3.33×10^{-17}	6.89×10^{-14}	Copy Number
**PSMD6**	CCR2	0.404	8.57×10^{-11}	7.46×10^{-9}	Copy Number
**PSMB1**	TAGAP	0.401	7.73×10^{-17}	2.71×10^{-14}	Copy Number
**PSMD6**	CCR5	0.401	1.19×10^{-10}	1.01×10^{-8}	Copy Number
**PSMD6**	CCRL2	0.401	1.21×10^{-10}	1.02×10^{-8}	Copy Number
**PSMD2**	BCL6	0.400	2.50×10^{-21}	7.38×10^{-19}	Copy Number
**PSMD11**	CCL8	0.399	1.13×10^{-16}	1.35×10^{-13}	Copy Number
**PSMB1**	ULBP1	0.399	1.30×10^{-16}	3.91×10^{-14}	Copy Number
**PSMD11**	CCL2	0.395	2.76×10^{-16}	2.86×10^{-13}	Copy Number
**PSMB1**	ULBP3	0.389	8.41×10^{-16}	1.91×10^{-13}	Copy Number
**PSMD11**	CCL1	0.388	9.84×10^{-16}	9.64×10^{-13}	Copy Number
**PSMB5**	NLRC5	0.382	2.77×10^{-24}	8.85×10^{-21}	Expression
**PSMA7**	CD40	0.381	3.12×10^{-15}	1.12×10^{-12}	Copy Number
**PSMC3**	SPI1	0.378	6.55×10^{-19}	9.53×10^{-16}	Copy Number
**PSMD11**	CCL13	0.375	9.27×10^{-15}	6.90×10^{-12}	Copy Number
**PSMB6**	TAP1	0.370	8.95×10^{-23}	4.30×10^{-19}	Expression
**PSMA4**	CD276	0.361	2.75×10^{-17}	5.81×10^{-15}	Copy Number
**PSMA6**	NFKBIA	0.358	5.03×10^{-17}	8.44×10^{-15}	Copy Number
**PSMD6**	CCR8	0.350	2.79×10^{-8}	1.75×10^{-6}	Copy Number
**PSMB1**	THEMIS	0.348	8.78×10^{-13}	1.40×10^{-10}	Copy Number
**PSMB4**	RORC	0.346	1.23×10^{-12}	9.68×10^{-10}	Copy Number
**PSMB5**	TRIM38	0.346	6.55×10^{-20}	6.28×10^{-17}	Expression
**PSMC6**	BATF	0.335	6.27×10^{-15}	7.29×10^{-13}	Copy Number
**PSMC1**	TGFB3	0.333	7.44×10^{-15}	6.35×10^{-13}	Copy Number
**PSMA7**	ZBTB46	0.332	1.02×10^{-11}	1.24×10^{-9}	Copy Number
**PSMA3**	ARG2	0.330	1.15×10^{-14}	1.48×10^{-12}	Copy Number
**PSMC1**	BATF	0.329	1.52×10^{-14}	1.27×10^{-12}	Copy Number
**PSMA3**	BATF	0.325	3.42×10^{-14}	3.88×10^{-12}	Copy Number
**PSMA2**	NOD1	0.324	3.45×10^{-11}	7.83×10^{-9}	Copy Number
**PSMB5**	TAP1	0.324	1.76×10^{-17}	1.26×10^{-14}	Expression
**PSMC6**	ARG2	0.323	5.54×10^{-14}	5.54×10^{-12}	Copy Number
**PSMC2**	CCL26	0.322	6.31×10^{-14}	4.94×10^{-12}	Copy Number
**PSMD4**	RORC	0.322	6.35×10^{-14}	1.32×10^{-11}	Copy Number
**PSMD4**	CD160	0.321	8.01×10^{-14}	1.52×10^{-11}	Copy Number
**PSMC4**	NFKBIB	0.321	7.43×10^{-14}	1.15×10^{-10}	Copy Number
**PSMA7**	ADA	0.318	8.23×10^{-11}	8.37×10^{-9}	Copy Number
**PSMB1**	ARG1	0.315	1.27×10^{-10}	1.58×10^{-8}	Copy Number
**PSMD6**	CCR4	0.315	6.93×10^{-7}	3.94×10^{-5}	Copy Number
**PSMC2**	CD36	0.314	2.76×10^{-13}	1.84×10^{-11}	Copy Number
**PSMC6**	TGFB3	0.312	4.21×10^{-13}	3.58×10^{-11}	Copy Number
**PSMC2**	CCL24	0.311	4.80×10^{-13}	3.01×10^{-11}	Copy Number
**PSMB4**	CD160	0.308	3.50×10^{-10}	6.65×10^{-8}	Copy Number
**PSMD2**	TNFSF10	0.307	9.29×10^{-13}	1.04×10^{-10}	Copy Number

Negatively correlated immune-related genes (n = 41)					
**Subunit**	**Feature gene**	**Pearson r**	**P value**	**Q value**	**Feature type**
**PSMG1**	OASL	−0.341	5.85×10^{-10}	4.03×10^{-6}	Expression
**PSMD6**	S100A9	−0.329	2.59×10^{-9}	5.71×10^{-7}	Expression
**PSMG1**	ADAM8	−0.322	6.12×10^{-9}	1.47×10^{-5}	Expression
**PSMG1**	TAP2	−0.319	7.96×10^{-9}	1.70×10^{-5}	Expression
**PSMA3**	CCL7	−0.312	2.38×10^{-16}	6.54×10^{-13}	Expression
**PSMD6**	TNFSF10	−0.309	2.45×10^{-8}	3.02×10^{-6}	Expression
**PSMA3**	CCL2	−0.308	5.49×10^{-16}	1.14×10^{-12}	Expression
**PSMG2**	TNF	−0.305	1.60×10^{-6}	6.77×10^{-4}	Copy Number
**PSMD6**	S100A8	−0.304	4.21×10^{-8}	4.76×10^{-6}	Expression
**PSMG2**	NCR3	−0.301	2.14×10^{-6}	7.00×10^{-4}	Copy Number
**PSMG1**	OAS2	−0.299	7.43×10^{-8}	7.13×10^{-5}	Expression
**PSMD6**	CD24	−0.297	9.07×10^{-8}	8.79×10^{-6}	Expression
**PSMD6**	TNFSF13	−0.297	9.18×10^{-8}	8.81×10^{-6}	Expression
**PSMG1**	MX1	−0.297	9.41×10^{-8}	8.21×10^{-5}	Expression
**PSMA5**	BIRC3	−0.291	1.78×10^{-11}	5.16×10^{-9}	Expression
**PSMA5**	CASP4	−0.285	4.93×10^{-11}	1.10×10^{-8}	Expression
**PSMG2**	ADAM8	−0.285	3.07×10^{-7}	4.54×10^{-4}	Expression
**PSMG1**	OAS3	−0.281	4.33×10^{-7}	2.52×10^{-4}	Expression
**PSMG2**	MICB	−0.281	1.07×10^{-5}	1.67×10^{-3}	Copy Number
**PSMG1**	SP110	−0.280	4.81×10^{-7}	2.64×10^{-4}	Expression
**PSMG1**	SP100	−0.278	6.32×10^{-7}	3.11×10^{-4}	Expression
**PSMG2**	MICB	−0.277	6.51×10^{-7}	8.93×10^{-4}	Expression
**PSMG2**	TAP1	−0.274	9.18×10^{-7}	1.07×10^{-3}	Expression
**PSMG2**	STING1	−0.272	2.02×10^{-5}	2.46×10^{-3}	Copy Number
**PSMG2**	TAP2	−0.270	1.25×10^{-6}	1.22×10^{-3}	Expression
**PSMA3**	CD151	−0.269	1.94×10^{-12}	6.41×10^{-10}	Expression
**PSMA5**	AREG	−0.269	5.97×10^{-10}	7.60×10^{-8}	Expression
**PSMA5**	C3	−0.264	1.30×10^{-9}	1.40×10^{-7}	Expression
**PSMA5**	TNFRSF21	−0.263	1.51×10^{-9}	1.58×10^{-7}	Expression
**PSMA5**	NT5E	−0.263	1.59×10^{-9}	1.61×10^{-7}	Expression
**PSMG2**	CASP8	−0.262	2.62×10^{-6}	1.87×10^{-3}	Expression
**PSMG1**	TAP1	−0.262	2.71×10^{-6}	9.14×10^{-4}	Expression
**PSMA3**	CD302	−0.262	8.21×10^{-12}	2.05×10^{-9}	Expression
**PSMG2**	C2	−0.261	4.41×10^{-5}	4.23×10^{-3}	Copy Number
**PSMG1**	AREG	−0.261	3.09×10^{-6}	9.73×10^{-4}	Expression
**PSMG1**	CASP8	−0.258	3.73×10^{-6}	1.02×10^{-3}	Expression
**PSMA5**	VSIR	−0.258	2.98×10^{-9}	2.53×10^{-7}	Expression
**PSMD6**	PGLYRP4	−0.257	4.26×10^{-6}	1.83×10^{-4}	Expression
**PSMC6**	A2M	−0.257	2.35×10^{-11}	2.26×10^{-8}	Expression
**PSMG2**	TSLP	−0.257	5.92×10^{-5}	5.35×10^{-3}	Copy Number
**PSMG2**	OAS2	−0.256	4.51×10^{-6}	2.62×10^{-3}	Expression

Supplementary Table S6. Gene composition of ssGSEA pathways

Gene composition of pathways shown in Fig. 5

Pathway	Source	Gene Composition	N
**MHC_PATHWAY**	BioCarta	B2M, CD74, HLA-A, HLA-DRA, HLA-DRB1, HLA-DRB3, HLA-DRB4, HLA-DRB5, PSMB5, PSMB6, PSMB8, PSMB9, TAP1, TAP2	14
**CD48**	GNF2	ARHGDIB, CD48, CD53, CORO1A, CORO7, CSK, DOCK2, GRK6, HCLS1, HLA-A, HLA-B, HLA-C, HLA-E, HLA-F, INPP5D, LAPTM5, LIMD2, MBD2, PHF11, PSMB10, PSMB8, PTPN6, PTPRC, RAC2, SASH3, SIPA1, SP110, STAT6, TAPBP, TNFAIP8, TRIM38, VAV1	32
**HLA_C**	GNF2	ARF6, ARHGAP45, ARHGDIB, B2M, CD48, CD53, CORO1A, CORO7, CYBA, CYBC1, CYTIP, ELF4, FXYD5, GPSM3, GRK6, HCLS1, HLA-A, HLA-B, HLA-C, HLA-E, HLA-F, HLA-G, INPP5D, IRF1, LAPTM5, LRCH4, MAX, PHF11, PNRC1, PRR13, PSD4, PSMB10, PTPN6, PTPRC, RAC2, RIN3, SASH3, SERP1, SIPA1, STAT6, SUSD6, TAP1, TAPBP, TRIM22, TSC22D3, VAV1, WAS	47
**INTERFERON_GAMMA_RESPONSE**	Hallmark	ADAR, APOL6, ARID5B, ARL4A, AUTS2, B2M, BANK1, BATF2, BPGM, BST2, BTG1, C1R, C1S, CASP1, CASP3, CASP4, CASP7, CASP8, CCL2, CCL5, CCL7, CD274, CD38, CD40, CD69, CD74, CD86, CDKN1A, CFB, CFH, CIITA, CMKLR1, CMPK2, CMTR1, CSF2RB, CXCL10, CXCL11, CXCL9, DDX60, DHX58, EIF2AK2, EIF4E3, EPSTI1, FAS, FCGR1A, FGL2, FPR1, GBP4, GBP6, GCH1, GPR18, GZMA, HELZ2, HERC6, HIF1A, HLA-A, HLA-B, HLA-DMA, HLA-DQA1, HLA-DRB1, HLA-G, ICAM1, IDO1, IFI27, IFI30, IFI35, IFI44, IFI44L, IFIH1, IFIT1, IFIT2, IFIT3, IFITM2, IFITM3, IFNAR2, IL10RA, IL15, IL15RA, IL18BP, IL2RB, IL4R, IL6, IL7, IRF1, IRF2, IRF4, IRF5, IRF7, IRF8, IRF9, ISG15, ISG20, ISOC1, ITGB7, JAK2, KLRK1, LAP3, LATS2, LCP2, LGALS3BP, LY6E, LYSMD2, MARCHF1, MT2A, MTHFD2, MVP, MX1, MX2, MYD88, NAMPT, NCOA3, NFKB1, NFKBIA, NLRC5, NMI, NOD1, NUP93, OAS2, OAS3, OASL, OGFR, P2RY14, PARP12, PARP14, PDE4B, PELI1, PFKP, PIM1, PLA2G4A, PLSCR1, PML, PNP, PNPT1, PSMA2, PSMA3, PSMB10, PSMB2, PSMB8, PSMB9, PSME1, PSME2, PTGS2, PTPN1, PTPN2, PTPN6, RAPGEF6, RBCK1, RIGI, RIPK1, RIPK2, RNF213, RNF31, RSAD2, RTP4, SAMD9L, SAMHD1, SECTM1, SELP, SERPING1, SLAMF7, SLC25A28, SOCS1, SOCS3, SOD2, SP110, SPPL2A, SRI, SSPN, ST3GAL5, ST8SIA4, STAT1, STAT2, STAT3, STAT4, TAP1, TAPBP, TDRD7, TMT1B, TNFAIP2, TNFAIP3, TNFAIP6, TNFSF10, TOR1B, TRAFD1, TRIM14, TRIM21, TRIM25, TRIM26, TXNIP, UBE2L6, UPP1, USP18, VAMP5, VAMP8, VCAM1, WARS1, XAF1, XCL1, ZBP1, ZNFX1	200
**INTERLEUKIN_2_FAMILY_SIGNALING**	Reactome	CSF2, CSF2RA, CSF2RB, GAB2, GRB2, HAVCR2, IL15, IL15RA, IL2, IL21, IL21R, IL2RA, IL2RB, IL2RG, IL3, IL3RA, IL5, IL5RA, IL9, IL9R, INPP5D, INPPL1, JAK1, JAK2, JAK3, LCK, LGALS9, PIK3CA, PIK3CB, PIK3CD, PIK3R1, PIK3R2, PIK3R3, PTK2B, PTPN6, SHC1, SOS1, SOS2, STAT1, STAT3, STAT4, STAT5A, STAT5B, SYK	44
**DEVELOPMENT_OF_PULMONARY_DENDRITIC_CELLS_AND_MACF**	WP	BATF3, CSF1, CSF2, FLT3LG, ID2, IKZF1, IRF4, IRF8, RUNX2, SPI1, STAT3, TCF4, TPO	13

Gene composition was retrieved from MSigDB gene sets used for ssGSEA analysis.

Supplementary Table S7. ssGSEA_CRISPR Correlations

Positively correlated pathways with CRISPR dependency scores (Top 50)

Subunit	Pathway	Pearson r	P value
**PSMB5**	GNF2_CD48	0.547	6.54×10^{-85}
**PSMB5**	GNF2_CD53	0.520	2.00×10^{-75}
**PSMB5**	GNF2_VAV1	0.516	4.24×10^{-74}
**PSMB5**	GNF2_HLA C	0.507	3.32×10^{-71}
**PSMB5**	GNF2_INPP5D	0.503	3.33×10^{-70}
**PSMB5**	GNF2_PTPN6	0.497	2.54×10^{-68}
**PSMB5**	GNF2_PTPRC	0.497	3.09×10^{-68}
**PSMB5**	GNF2_CD97	0.469	5.50×10^{-60}
**PSMB5**	GNF2_ICAM3	0.466	5.52×10^{-59}
**PSMB5**	KEGG_ALLOGRAFT REJECTION	0.460	2.26×10^{-57}
**PSMB5**	HALLMARK_ALLOGRAFT REJECTION	0.457	1.15×10^{-56}
**PSMB5**	GNF2_ITGAL	0.456	2.53×10^{-56}
**PSMB5**	KEGG_INTESTINAL IMMUNE NETWORK FOR IGA PRODUCTION	0.451	5.92×10^{-55}
**PSMB5**	KEGG_GRAFT VERSUS HOST DISEASE	0.448	2.96×10^{-54}
**PSMB5**	GNF2_SELL	0.446	9.80×10^{-54}
**PSMB5**	KEGG_PRIMARY IMMUNODEFICIENCY	0.445	2.23×10^{-53}
**PSMB5**	REACTOME_INTERLEUKIN 2 FAMILY SIGNALING	0.444	3.40×10^{-53}
**PSMB5**	GNF2_ITGB2	0.438	1.10×10^{-51}
**PSMB5**	BIOCARTA_MHC PATHWAY	0.434	1.06×10^{-50}
**PSMB5**	GNF2_STAT6	0.429	1.70×10^{-49}
**PSMB5**	KEGG_AUTOIMMUNE THYROID DISEASE	0.424	3.34×10^{-48}
**PSMB5**	GNF2_TNFRSF1B	0.423	7.31×10^{-48}
**PSMB5**	KEGG_ANTIGEN PROCESSING AND PRESENTATION	0.421	2.08×10^{-47}
**PSMB5**	WP_TYROBP CAUSAL NETWORK	0.417	1.28×10^{-46}
**PSMB5**	WP_MICROGLIA PATHOGEN PHAGOCYTOSIS PATHWAY	0.417	1.41×10^{-46}
**PSMB5**	BIOCARTA_CTL PATHWAY	0.414	9.57×10^{-46}
**PSMB5**	GNF2_MYD88	0.413	1.25×10^{-45}
**PSMB5**	REACTOME_INTERLEUKIN 2 SIGNALING	0.407	3.09×10^{-44}
**PSMB5**	GNF2_LYN	0.405	1.01×10^{-43}
**PSMB5**	REACTOME_IMMUNOREGULATORY INTERACTIONS BETWEEN A LYMPHOID	0.405	1.16×10^{-43}
**PSMB5**	BIOCARTA_BLYMPHOCYTE PATHWAY	0.404	1.65×10^{-43}
**PSMB5**	WP_ALLOGRAFT REJECTION	0.403	3.04×10^{-43}
**PSMB5**	GNF2_TNFSF10	0.398	4.44×10^{-42}
**PSMB5**	GNF2_FGR	0.394	3.17×10^{-41}
**PSMB5**	GNF2_JAK1	0.388	4.85×10^{-40}
**PSMB5**	REACTOME_INTERLEUKIN 21 SIGNALING	0.388	6.38×10^{-40}
**PSMB5**	WP_TYPE II INTERFERON SIGNALING IFNG	0.386	1.66×10^{-39}
**PSMB5**	GNF2_HCK	0.385	1.94×10^{-39}
**PSMB5**	REACTOME_INTERFERON GAMMA SIGNALING	0.381	1.49×10^{-38}
**PSMB5**	BIOCARTA_GRANULOCYTES PATHWAY	0.381	1.83×10^{-38}
**PSMB5**	REACTOME_PD 1 SIGNALING	0.380	2.81×10^{-38}
**PSMB5**	GNF2_SPI1	0.377	1.10×10^{-37}
**PSMB5**	BIOCARTA_ASBCELL PATHWAY	0.372	1.11×10^{-36}
**PSMB5**	GNF2_CD1D	0.372	1.13×10^{-36}
**PSMB5**	GNF2_ZAP70	0.371	2.27×10^{-36}
**PSMB5**	GNF2_CD7	0.369	4.52×10^{-36}
**PSMB5**	REACTOME_INTERLEUKIN 9 SIGNALING	0.367	1.38×10^{-35}
**PSMB5**	HALLMARK_INTERFERON GAMMA RESPONSE	0.366	2.30×10^{-35}
**PSMB5**	REACTOME_ANTIGEN ACTIVATES B CELL RECEPTOR BCR LEADING TO	0.365	3.02×10^{-35}
**PSMB5**	GNF2_CARD15	0.364	4.50×10^{-35}

Negatively correlated pathways with CRISPR dependency scores (Top 50)

Subunit	Pathway	Pearson r	P value
**PSMB1**	REACTOME_INTERFERON GAMMA SIGNALING	−0.198	5.37×10^{-11}
**PSMB1**	WP_TYPE II INTERFERON SIGNALING IFNG	−0.196	9.69×10^{-11}
**PSMG1**	BIOCARTA_EOSINOPHILS PATHWAY	−0.180	2.57×10^{-9}
**PSMB1**	WP_CANCER IMMUNOTHERAPY BY PD1 BLOCKADE	−0.178	4.30×10^{-9}
**PSMB1**	GNF2_CD53	−0.177	5.04×10^{-9}
**PSMB1**	BIOCARTA_MHC PATHWAY	−0.175	7.82×10^{-9}
**PSMB1**	GNF2_INPP5D	−0.174	8.87×10^{-9}
**PSMB1**	HALLMARK_INTERFERON GAMMA RESPONSE	−0.173	1.13×10^{-8}
**POMP**	REACTOME_INTERLEUKIN 6 FAMILY SIGNALING	−0.172	1.18×10^{-8}
**PSMB1**	WP_DEVELOPMENT OF PULMONARY DENDRITIC CELLS AND MACROPHAGE	−0.172	1.37×10^{-8}
**PSMB1**	WP_MIRNAS INVOLVEMENT IN THE IMMUNE RESPONSE IN SEPSIS	−0.171	1.75×10^{-8}
**PSMG1**	WP_NANOMATERIALINDUCED INFLAMMASOME ACTIVATION	−0.170	1.88×10^{-8}
**PSMB1**	GNF2_ITGAL	−0.170	1.92×10^{-8}
**PSMB1**	GNF2_JAK1	−0.170	2.01×10^{-8}
**PSMB8**	KEGG_ALLOGRAFT REJECTION	−0.170	2.09×10^{-8}
**PSMB1**	REACTOME_INTERFERON ALPHA BETA SIGNALING	−0.169	2.35×10^{-8}
**PSMB1**	REACTOME_ANTIGEN PRESENTATION FOLDING ASSEMBLY AND PEPTIDE	−0.169	2.39×10^{-8}
**PSMB1**	WP_THE HUMAN IMMUNE RESPONSE TO TUBERCULOSIS	−0.169	2.50×10^{-8}
**PSMB1**	KEGG_GRAFT VERSUS HOST DISEASE	−0.168	3.23×10^{-8}
**PSMB8**	KEGG_INTESTINAL IMMUNE NETWORK FOR IGA PRODUCTION	−0.167	3.36×10^{-8}
**PSMG1**	KEGG_ANTIGEN PROCESSING AND PRESENTATION	−0.166	4.08×10^{-8}
**PSMB1**	GNF2_PTPRC	−0.166	4.11×10^{-8}
**PSMG1**	REACTOME_INTERLEUKIN 10 SIGNALING	−0.165	4.83×10^{-8}
**PSMG1**	REACTOME_CLEC7A INFLAMMASOME PATHWAY	−0.164	6.84×10^{-8}
**PSMB1**	GNF2_HLA C	−0.163	7.17×10^{-8}
**POMP**	REACTOME_INTERLEUKIN 6 SIGNALING	−0.163	7.11×10^{-8}
**PSMG1**	BIOCARTA_MHC PATHWAY	−0.163	7.87×10^{-8}
**PSMB8**	GNF2_VAV1	−0.162	8.39×10^{-8}
**PSMB9**	REACTOME_PD 1 SIGNALING	−0.162	8.48×10^{-8}
**SEM1**	WP_DEVELOPMENT OF PULMONARY DENDRITIC CELLS AND MACROPHAGE	−0.162	8.34×10^{-8}
**PSMB1**	HALLMARK_INTERFERON ALPHA RESPONSE	−0.162	9.90×10^{-8}
**PSMB1**	KEGG_ALLOGRAFT REJECTION	−0.161	1.03×10^{-7}
**PSMB1**	KEGG_ANTIGEN PROCESSING AND PRESENTATION	−0.160	1.23×10^{-7}
**PSMG1**	REACTOME_INTERLEUKIN 1 PROCESSING	−0.160	1.42×10^{-7}
**POMP**	REACTOME_IL 6 TYPE CYTOKINE RECEPTOR LIGAND INTERACTIONS	−0.159	1.34×10^{-7}
**PSMB1**	GNF2_PTPN6	−0.159	1.61×10^{-7}
**PSMB1**	GNF2_CD48	−0.158	1.78×10^{-7}
**PSMG1**	KEGG_CYTOKINE CYTOKINE RECEPTOR INTERACTION	−0.156	2.54×10^{-7}
**PSMB8**	GNF2_INPP5D	−0.156	2.66×10^{-7}
**PSMB8**	GNF2_PTPN6	−0.154	3.91×10^{-7}
**PSMG1**	REACTOME_INTERLEUKIN 4 AND INTERLEUKIN 13 SIGNALING	−0.154	3.96×10^{-7}
**PSMB1**	GNF2_MYD88	−0.154	4.05×10^{-7}
**PSMB8**	GNF2_CD48	−0.153	4.75×10^{-7}
**PSMG2**	WP_SELECTIVE EXPRESSION OF CHEMOKINE RECEPTORS DURING TCELL	−0.153	4.76×10^{-7}
**PSMG1**	BIOCARTA_BBCELL PATHWAY	−0.153	4.79×10^{-7}
**PSMG1**	WP_ALLOGRAFT REJECTION	−0.153	4.85×10^{-7}
**PSMG3**	BIOCARTA_MHC PATHWAY	−0.152	5.06×10^{-7}
**PSMB8**	WP_TOLLLIKE RECEPTOR SIGNALING RELATED TO MYD88	−0.152	5.60×10^{-7}
**POMP**	BIOCARTA_IFNA PATHWAY	−0.152	5.35×10^{-7}
**PSMG1**	WP_ACTIVATION OF NLRP3 INFLAMMASOME BY SARSCOV2	−0.152	5.84×10^{-7}

Supplementary Table S8. ssGSEA_RNAi Correlations

Positively correlated pathways with RNAi dependency scores (Top 50)			
**Subunit**	**Pathway**	**Pearson r**	**P value**
**PSMB5**	GNF2_HLA C	0.479	5.67×10^{-39}
**PSMB5**	GNF2_CD48	0.463	3.59×10^{-36}
**PSMB5**	GNF2_INPP5D	0.442	1.02×10^{-32}
**PSMB5**	WP_TYROBP CAUSAL NETWORK	0.417	1.28×10^{-46}
**PSMB5**	GNF2_CD53	0.417	5.11×10^{-29}
**PSMB5**	GNF2_VAV1	0.411	3.35×10^{-28}
**PSMB5**	GNF2_PTPRC	0.411	3.93×10^{-28}
**PSMB5**	GNF2_CD97	0.407	1.14×10^{-27}
**PSMB5**	WP_TYPE II INTERFERON SIGNALING IFNG	0.386	1.66×10^{-39}
**PSMB5**	GNF2_ICAM3	0.384	1.49×10^{-24}
**PSMB5**	BIOCARTA_MHC PATHWAY	0.383	2.18×10^{-24}
**PSMB5**	HALLMARK_INTERFERON GAMMA RESPONSE	0.382	2.86×10^{-24}
**PSMB5**	GNF2_PTPN6	0.382	3.25×10^{-24}
**PSMB5**	REACTOME_INTERFERON GAMMA SIGNALING	0.377	1.22×10^{-23}
**PSMB5**	HALLMARK_INTERFERON ALPHA RESPONSE	0.374	3.36×10^{-23}
**PSMB5**	GNF2_STAT6	0.368	1.48×10^{-22}
**PSMB5**	REACTOME_INTERFERON ALPHA BETA SIGNALING	0.368	1.64×10^{-22}
**PSMB5**	WP_MICROGLIA PATHOGEN PHAGOCYTOSIS PATHWAY	0.368	1.84×10^{-22}
**PSMB5**	GNF2_SELL	0.367	2.10×10^{-22}
**PSMB5**	GNF2_HCK	0.366	3.23×10^{-22}
**PSMB5**	REACTOME_INTERLEUKIN 2 FAMILY SIGNALING	0.366	3.35×10^{-22}
**PSMB5**	GNF2_CARD15	0.363	5.92×10^{-22}
**PSMB5**	BIOCARTA_NKCELLS PATHWAY	0.362	1.02×10^{-21}
**PSMB6**	HALLMARK_INTERFERON GAMMA RESPONSE	0.359	1.78×10^{-21}
**PSMB5**	HALLMARK_ALLOGRAFT REJECTION	0.352	1.39×10^{-20}
**PSMB5**	WP_SELECTIVE EXPRESSION OF CHEMOKINE RECEPTORS DURING TCELL	0.348	4.64×10^{-32}
**PSMB5**	GNF2_CD1D	0.348	3.62×10^{-20}
**PSMB5**	WP_TYPE II INTERFERON SIGNALING IFNG	0.345	8.13×10^{-20}
**PSMB5**	REACTOME_CROSS PRESENTATION OF PARTICULATE EXOGENOUS ANTIGENS	0.345	9.16×10^{-20}
**PSMB5**	GNF2_ITGAL	0.343	1.35×10^{-19}
**PSMB5**	GNF2_PECAM1	0.342	1.66×10^{-19}
**PSMB5**	GNF2_ITGB2	0.342	1.96×10^{-19}
**PSMB6**	REACTOME_INTERFERON ALPHA BETA SIGNALING	0.342	1.79×10^{-19}
**PSMB5**	GNF2_MYD88	0.341	2.34×10^{-19}
**PSMB6**	HALLMARK_INTERFERON ALPHA RESPONSE	0.341	2.13×10^{-19}
**PSMB6**	BIOCARTA_MHC PATHWAY	0.341	2.26×10^{-19}
**PSMB5**	GNF2_TNFRSF1B	0.334	1.52×10^{-18}
**PSMB5**	KEGG_GRAFT VERSUS HOST DISEASE	0.333	1.86×10^{-18}
**PSMB5**	GNF2_TNFSF10	0.333	1.94×10^{-18}
**PSMB5**	GNF2_SPI1	0.329	4.45×10^{-18}
**PSMB5**	WP_TYROBP CAUSAL NETWORK	0.327	8.58×10^{-18}
**PSMB5**	KEGG_PRIMARY IMMUNODEFICIENCY	0.324	1.62×10^{-17}
**PSMB5**	KEGG_ALLOGRAFT REJECTION	0.321	3.18×10^{-17}
**PSMB5**	GNF2_LYN	0.319	4.67×10^{-17}
**PSMB5**	WP_MACROPHAGE MARKERS	0.319	5.19×10^{-17}
**PSMB5**	REACTOME_INTERLEUKIN 18 SIGNALING	0.319	5.68×10^{-17}
**PSMB5**	KEGG_ANTIGEN PROCESSING AND PRESENTATION	0.318	6.71×10^{-17}
**PSMB5**	GNF2_FGR	0.318	6.95×10^{-17}
**PSMB6**	REACTOME_INTERFERON GAMMA SIGNALING	0.317	7.89×10^{-17}
**PSMB5**	REACTOME_IMMUNOREGULATORY INTERACTIONS BETWEEN A LYMPHOID	0.315	1.34×10^{-16}

Negatively correlated pathways with RNAi dependency scores (Top 50)			
**Subunit**	**Pathway**	**Pearson r**	**P value**
**PSMG1**	HALLMARK_INTERFERON ALPHA RESPONSE	−0.383	2.71×10^{-12}
**PSMG1**	HALLMARK_INTERFERON GAMMA RESPONSE	−0.363	4.02×10^{-11}
**PSMG1**	REACTOME_INTERFERON ALPHA BETA SIGNALING	−0.356	9.88×10^{-11}
**PSMG2**	REACTOME_INTERFERON ALPHA BETA SIGNALING	−0.351	1.79×10^{-10}
**PSMG2**	REACTOME_INTERFERON GAMMA SIGNALING	−0.342	6.03×10^{-10}
**PSMG2**	HALLMARK_INTERFERON GAMMA RESPONSE	−0.341	6.52×10^{-10}
**PSMG2**	HALLMARK_INTERFERON ALPHA RESPONSE	−0.333	1.68×10^{-9}
**PSMG2**	BIOCARTA_MHC PATHWAY	−0.331	2.22×10^{-9}
**PSMG1**	REACTOME_INTERFERON GAMMA SIGNALING	−0.330	2.41×10^{-9}
**PSMG2**	KEGG_ANTIGEN PROCESSING AND PRESENTATION	−0.330	2.47×10^{-9}
**PSMG2**	KEGG_ALLOGRAFT REJECTION	−0.322	6.26×10^{-9}
**PSMG2**	BIOCARTA_EOSINOPHILS PATHWAY	−0.319	9.11×10^{-9}
**PSMG2**	GNF2_HLA C	−0.316	1.21×10^{-8}
**PSMG2**	KEGG_GRAFT VERSUS HOST DISEASE	−0.314	1.48×10^{-8}
**PSMA5**	KEGG_CYTOKINE CYTOKINE RECEPTOR INTERACTION	−0.312	4.88×10^{-13}
**PSMG2**	REACTOME_ANTIGEN PRESENTATION FOLDING ASSEMBLY AND PEPTIDE	−0.309	2.69×10^{-8}
**PSMG2**	KEGG_AUTOIMMUNE THYROID DISEASE	−0.299	7.72×10^{-8}
**PSMG1**	WP_TYPE II INTERFERON SIGNALING IFNG	−0.298	8.21×10^{-8}
**PSMG2**	GNF2_INPP5D	−0.294	1.30×10^{-7}
**PSMG2**	GNF2_CD48	−0.291	1.73×10^{-7}
**PSMG2**	REACTOME_INTERLEUKIN 1 PROCESSING	−0.290	1.95×10^{-7}
**PSMG2**	WP_ALLOGRAFT REJECTION	−0.287	2.67×10^{-7}
**PSMG2**	BIOCARTA_BLYMPHOCYTE PATHWAY	−0.285	3.16×10^{-7}
**PSMG2**	REACTOME_INTERFERON SIGNALING	−0.280	4.97×10^{-7}
**PSMG2**	GNF2_STAT6	−0.278	6.30×10^{-7}
**PSMG1**	REACTOME_INTERLEUKIN 1 PROCESSING	−0.278	6.43×10^{-7}
**PSMA5**	REACTOME_INTERLEUKIN 10 SIGNALING	−0.277	1.74×10^{-10}
**PSMG1**	GNF2_HLA C	−0.277	6.69×10^{-7}
**PSMG2**	WP_TYPE II INTERFERON SIGNALING IFNG	−0.275	8.39×10^{-7}
**PSMG2**	WP_MACROPHAGE MARKERS	−0.275	8.80×10^{-7}
**PSMG2**	REACTOME_DDX58 IFIH1 MEDIATED INDUCTION OF INTERFERON ALPHA	−0.274	9.21×10^{-7}
**PSMG2**	GNF2_CD53	−0.273	1.02×10^{-6}
**PSMG2**	HALLMARK_ALLOGRAFT REJECTION	−0.273	1.04×10^{-6}
**PSMG2**	GNF2_VAV1	−0.271	1.20×10^{-6}
**PSMA5**	WP_CYTOKINES AND INFLAMMATORY RESPONSE	−0.269	6.06×10^{-10}
**PSMG2**	GNF2_CARD15	−0.268	1.64×10^{-6}
**PSMA5**	REACTOME_INTERLEUKIN 36 PATHWAY	−0.267	8.13×10^{-10}
**PSMG1**	WP_TOLLLIKE RECEPTOR SIGNALING RELATED TO MYD88	−0.267	1.81×10^{-6}
**PSMG1**	WP_SIMPLIFIED DEPICTION OF MYD88 DISTINCT INPUTOUTPUT PATHWAY	−0.266	1.88×10^{-6}
**PSMG1**	BIOCARTA_MHC PATHWAY	−0.265	2.19×10^{-6}
**PSMG1**	REACTOME_INTERFERON SIGNALING	−0.265	2.20×10^{-6}
**PSMG2**	GNF2_PECAM1	−0.265	2.24×10^{-6}
**PSMG2**	WP_TOLLLIKE RECEPTOR SIGNALING RELATED TO MYD88	−0.264	2.29×10^{-6}
**PSMG2**	GNF2_PTPRC	−0.262	2.81×10^{-6}
**PSMG2**	GNF2_HCK	−0.261	3.01×10^{-6}
**PSMG1**	REACTOME_ANTIGEN PRESENTATION FOLDING ASSEMBLY AND PEPTIDE	−0.261	3.06×10^{-6}
**PSMG2**	KEGG_CYTOKINE CYTOKINE RECEPTOR INTERACTION	−0.261	3.20×10^{-6}
**PSMG2**	GNF2_PTPN6	−0.261	3.22×10^{-6}
**PSMA3**	REACTOME_IL 6 TYPE CYTOKINE RECEPTOR LIGAND INTERACTIONS	−0.260	1.09×10^{-11}
**PSMA3**	REACTOME_INTERLEUKIN 6 FAMILY SIGNALING	−0.260	1.11×10^{-11}

Supplementary Table S9. Robustness analysis of correlations after outlier removal

Robustness analysis of correlations after outlier removal

Gene	Condition	N	Removed n	Remaining (%)	Pearson r	P value
**PSMB5**	Original	9669	0	100.00	0.252	2.82e-140
	1.5×IQR	9110	559	94.22	0.234	9.68e-114
	1% trimming	9385	284	97.06	0.245	4.35e-128
**PSMB6**	Original	9669	0	100.00	0.198	4.18e-86
	1.5×IQR	9091	578	94.02	0.187	1.71e-72
	1% trimming	9382	287	97.03	0.202	1.15e-86
**PSMD11**	Original	9669	0	100.00	0.486	<1.0e-300
	1.5×IQR	9228	441	95.44	0.500	<1.0e-300
	1% trimming	9382	287	97.03	0.503	<1.0e-300
**PSMD14**	Original	9669	0	100.00	0.469	<1.0e-300
	1.5×IQR	9188	481	95.03	0.474	<1.0e-300
	1% trimming	9384	285	97.05	0.471	<1.0e-300

Pearson correlation coefficients were calculated between proteasome subunit expression and T cell CD4 Th2 xCell scores.

Supplementary Table S10. Meta-analysis summary of associations between proteasome subunit expression and CD4 Th2 cell infiltration (XCELL)

CD4_Th2_table

Meta-analysis summary

Gene	Cell type	Meta r	95% CI	Meta p	Meta FDR	I²	k	N
PSMD14	T.cell.CD4..Th2_XCELL	0.418	[0.317, 0.510]	8.36e-14	3.44e-12	90.4	24	3112
PSMB9	T.cell.CD4..Th2_XCELL	0.308	[0.233, 0.379]	1.39e-14	6.61e-13	79.5	24	3112
PSMA4	T.cell.CD4..Th2_XCELL	0.304	[0.203, 0.398]	1.12e-08	1.89e-07	88.6	24	3112
PSMD10	T.cell.CD4..Th2_XCELL	0.286	[0.208, 0.361]	4.44e-12	1.43e-10	80.7	24	3112
PSMB5	T.cell.CD4..Th2_XCELL	0.285	[0.200, 0.366]	2.32e-10	5.53e-09	81.9	22	2807
PSMA1	T.cell.CD4..Th2_XCELL	0.278	[0.162, 0.385]	4.07e-06	3.59e-05	91.0	24	3112
PSME3	T.cell.CD4..Th2_XCELL	0.260	[0.176, 0.341]	4.10e-09	7.49e-08	83.0	24	3112
PSMA6	T.cell.CD4..Th2_XCELL	0.245	[0.160, 0.327]	4.10e-08	6.10e-07	82.7	23	3021
PSMG3	T.cell.CD4..Th2_XCELL	0.240	[0.147, 0.329]	7.51e-07	8.27e-06	84.2	22	2807
PSMD1	T.cell.CD4..Th2_XCELL	0.238	[0.157, 0.315]	1.44e-08	2.40e-07	80.9	24	3112
PSMD2	T.cell.CD4..Th2_XCELL	0.231	[0.131, 0.327]	9.38e-06	7.33e-05	87.8	24	3112
PSMC4	T.cell.CD4..Th2_XCELL	0.218	[0.123, 0.310]	9.02e-06	7.08e-05	86.2	24	3112
PSMA5	T.cell.CD4..Th2_XCELL	0.216	[0.093, 0.333]	6.70e-04	2.94e-03	91.8	24	3112
PSMD11	T.cell.CD4..Th2_XCELL	0.215	[0.134, 0.294]	3.74e-07	4.43e-06	81.1	24	3112
PSMG1	T.cell.CD4..Th2_XCELL	0.207	[0.102, 0.308]	1.31e-04	7.31e-04	87.4	22	2826
PSMG2	T.cell.CD4..Th2_XCELL	0.207	[0.099, 0.309]	1.83e-04	9.65e-04	87.9	22	2826
PSMD6	T.cell.CD4..Th2_XCELL	0.204	[0.082, 0.320]	1.18e-03	4.76e-03	91.4	23	3058
PSMA7	T.cell.CD4..Th2_XCELL	0.190	[0.115, 0.262]	8.27e-07	8.95e-06	69.8	19	2427
PSMC6	T.cell.CD4..Th2_XCELL	0.184	[0.092, 0.274]	1.10e-04	6.28e-04	84.5	23	3012
PSMB4	T.cell.CD4..Th2_XCELL	0.181	[0.059, 0.298]	3.92e-03	1.29e-02	91.4	23	3058
PSMD13	T.cell.CD4..Th2_XCELL	0.172	[0.085, 0.256]	1.25e-04	7.03e-04	83.0	24	3112
PSMB8	T.cell.CD4..Th2_XCELL	0.171	[0.083, 0.257]	1.70e-04	9.14e-04	83.5	24	3112
PSMA8	T.cell.CD4..Th2_XCELL	0.170	[0.089, 0.249]	4.54e-05	2.96e-04	78.0	22	2807
PSMB10	T.cell.CD4..Th2_XCELL	0.165	[0.070, 0.257]	7.00e-04	3.05e-03	84.0	21	2777
PSMD7	T.cell.CD4..Th2_XCELL	0.161	[0.071, 0.248]	4.65e-04	2.14e-03	83.5	23	3058
PSMD9	T.cell.CD4..Th2_XCELL	0.160	[0.096, 0.223]	1.07e-06	1.12e-05	67.6	24	3112
PSMB3	T.cell.CD4..Th2_XCELL	0.160	[0.069, 0.247]	5.53e-04	2.48e-03	82.8	22	2900
PSMD12	T.cell.CD4..Th2_XCELL	0.159	[0.046, 0.269]	6.05e-03	1.88e-02	90.0	24	3112
PSME2	T.cell.CD4..Th2_XCELL	0.146	[0.066, 0.224]	3.53e-04	1.69e-03	78.5	23	3012
PSMD3	T.cell.CD4..Th2_XCELL	0.142	[0.073, 0.208]	5.20e-05	3.32e-04	71.1	23	3058
PSMB1	T.cell.CD4..Th2_XCELL	0.137	[0.021, 0.249]	2.12e-02	5.32e-02	90.4	24	3112
PSMA3	T.cell.CD4..Th2_XCELL	0.137	[0.023, 0.247]	1.86e-02	4.78e-02	90.0	24	3112
PSMB6	T.cell.CD4..Th2_XCELL	0.137	[0.082, 0.191]	1.24e-06	1.27e-05	54.5	23	3032

Supplementary Table S11. Meta-analysis summary of associations between proteasome subunit expression and CD8 cell infiltration (TIMER)

CD8_TIMER_table

Meta-analysis summary

Gene	Cell type	Meta r	95% CI	Meta p	Meta FDR	I^2	k	N
PSMB9	T.cell.CD8._TIMER	0.522	[0.451, 0.587]	2.70e-34	6.43e-32	84.6	24	3112
PSMB10	T.cell.CD8._TIMER	0.501	[0.384, 0.603]	1.58e-13	6.26e-12	93.2	21	2777
PSMB8	T.cell.CD8._TIMER	0.376	[0.276, 0.468]	4.83e-12	1.55e-10	89.5	24	3112
PSME1	T.cell.CD8._TIMER	0.242	[0.125, 0.352]	6.69e-05	4.11e-04	91.1	24	3112
PSME2	T.cell.CD8._TIMER	0.221	[0.121, 0.317]	1.95e-05	1.39e-04	87.1	23	3012
PSMD7	T.cell.CD8._TIMER	0.106	[0.017, 0.193]	1.97e-02	5.01e-02	83.0	23	3058
PSMA3	T.cell.CD8._TIMER	0.092	[0.001, 0.182]	4.79e-02	1.04e-01	84.0	24	3112
PSMB6	T.cell.CD8._TIMER	0.089	[0.035, 0.142]	1.30e-03	5.16e-03	52.0	23	3032

Supplementary Table S12. Meta-analysis summary of associations between proteasome subunit expression and Macrophage monocyte cell infiltration (MCPCOUNTER)

Macrophage_monocyte_MCPCOUNTER

Meta-analysis summary

Gene	Cell type	Meta r	95% CI	Meta p	Meta FDR	I²	k	N
PSMB10	Macrophage.Monocyte_MCPCOUNTER	0.433	[0.355, 0.504]	4.60e-23	5.26e-21	82.3	21	2777
PSMB9	Macrophage.Monocyte_MCPCOUNTER	0.347	[0.283, 0.407]	9.09e-24	1.15e-21	72.7	24	3112
PSMB8	Macrophage.Monocyte_MCPCOUNTER	0.309	[0.253, 0.362]	3.57e-25	5.23e-23	62.4	24	3112
PSMA1	Macrophage.Monocyte_MCPCOUNTER	0.236	[0.158, 0.311]	6.19e-09	1.09e-07	79.6	24	3112
PSMD14	Macrophage.Monocyte_MCPCOUNTER	0.228	[0.149, 0.305]	3.21e-08	4.99e-07	80.2	24	3112
PSMA5	Macrophage.Monocyte_MCPCOUNTER	0.203	[0.112, 0.292]	1.66e-05	1.21e-04	84.9	24	3112
PSMA4	Macrophage.Monocyte_MCPCOUNTER	0.203	[0.122, 0.282]	1.35e-06	1.36e-05	80.8	24	3112
PSMD7	Macrophage.Monocyte_MCPCOUNTER	0.151	[0.087, 0.215]	4.56e-06	3.94e-05	67.5	23	3058
PSMA6	Macrophage.Monocyte_MCPCOUNTER	0.141	[0.038, 0.242]	7.65e-03	2.28e-02	87.5	23	3021
PSME1	Macrophage.Monocyte_MCPCOUNTER	0.139	[0.064, 0.213]	3.12e-04	1.53e-03	76.8	24	3112
PSME3	Macrophage.Monocyte_MCPCOUNTER	0.135	[0.060, 0.208]	4.58e-04	2.12e-03	76.4	24	3112
PSMC4	Macrophage.Monocyte_MCPCOUNTER	0.128	[0.064, 0.190]	8.97e-05	5.24e-04	67.0	24	3112
PSMA3	Macrophage.Monocyte_MCPCOUNTER	0.127	[0.029, 0.222]	1.09e-02	3.07e-02	86.2	24	3112
PSMG2	Macrophage.Monocyte_MCPCOUNTER	0.125	[0.027, 0.221]	1.26e-02	3.45e-02	84.9	22	2826
PSMC6	Macrophage.Monocyte_MCPCOUNTER	0.121	[0.026, 0.213]	1.24e-02	3.41e-02	84.8	23	3012
PSME2	Macrophage.Monocyte_MCPCOUNTER	0.111	[0.052, 0.169]	2.21e-04	1.13e-03	59.3	23	3012
PSMD2	Macrophage.Monocyte_MCPCOUNTER	0.108	[0.039, 0.177]	2.23e-03	8.07e-03	72.1	24	3112
PSMB6	Macrophage.Monocyte_MCPCOUNTER	0.106	[0.036, 0.174]	2.88e-03	1.00e-02	71.4	23	3032
PSMD6	Macrophage.Monocyte_MCPCOUNTER	0.104	[0.009, 0.198]	3.22e-02	7.55e-02	85.3	23	3058
PSMD1	Macrophage.Monocyte_MCPCOUNTER	0.103	[0.046, 0.158]	3.76e-04	1.79e-03	57.4	24	3112

Supplementary Table S13. List of gene pairs per cluster

Cluster_C1	Gene1_C1	Gene2_C1	Cluster_C2	Gene1_C2	Gene2_C2	Cluster_C2	Gene1_C2	Gene2_C2	Cluster_C2	Gene1_C2	Gene2_C2	Cluster_C2	Gene1_C2	Gene2_C2	Cluster_C3	Gene1_C3	Gene2_C3	Cluster_C3	Gene1_C3	Gene2_C3	Cluster_C4	Gene1_C4	Gene2_C4
1	PSMB10	ACTG1	2	PSMD8	ADAT2	2	PSMC4	DHX33	2	PSMD1	NIP7	2	PSMB11	SOD2	3	PSMB10	ACO2	3	PSMB8	PPP1R15B	4	PSMD8	AKIRIN2
1	PSMB10	ACTR8	2	PSMB7	ADSL	2	PSMD13	DHX33	2	PSMD8	NIP7	2	PSMC5	SOD2	3	PSMB9	ACO2	3	PSMB7	PPP2R1A	4	PSMD8	ANAPC4
1	PSME2	AHCY	2	PSMB8	ADSL	2	PSMD8	DHX33	2	PSMB7	NMNAT1	2	PSMD8	SOD2	3	PSMB10	ALG11	3	PSMC5	PRMT5	4	PSMD8	ATP6V0B
1	PSMB10	APOLD1	2	PSME2	ADSL	2	PSMC1	DKC1	2	PSMD8	NOL9	2	PSMD9	SOD2	3	PSMA4	ANAPC11	3	PSME3	PRPF19	4	PSMD13	BCL2L1
1	PSME3	BRAF	2	PSMA4	AFG2B	2	PSMC4	DKC1	2	PSMD8	NOP10	2	PSMB2	SS18L2	3	PSMB8	ANAPC11	3	PSMB11	PSTK	4	PSMD8	BCL2L1
1	PSMB8	BRD2	2	PSMB10	AFG2B	2	PSMD8	DKC1	2	PSMD8	OSGEP	2	PSMD1	SS18L2	3	PSMB9	ANAPC11	3	PSMB7	PTK2	4	PSMD8	CCDC174
1	PSMB10	C4orf46	2	PSMB8	AFG2B	2	PSMD8	DNAJC11	2	PSMD9	OSGEP	2	PSMD8	SS18L2	3	PSME3	ANAPC11	3	PSME2	PTK2	4	PSMD8	CDCA5
1	PSMB8	CCNT1	2	PSMC1	AFG2B	2	PSMD8	DNLZ	2	PSMB7	PAICS	2	PSMA1	TAMM41	3	PSMA4	ANAPC2	3	PSMC4	PUF60	4	PSMC1	CHEK1
1	PSMB10	CEP43	2	PSMC3	AFG2B	2	PSMB2	DPAGT1	2	PSMB11	PAK1IP1	2	PSMA4	TAMM41	3	PSMB9	ANAPC2	3	PSMA4	RBM22	4	PSMD8	CHEK1
1	PSMB8	COX7C	2	PSMC4	AFG2B	2	PSMC1	DPAGT1	2	PSMD8	PAK1IP1	2	PSMC1	TAMM41	3	PSMC4	ANAPC2	3	PSMB8	RBM22	4	PSMD8	COPB1
1	PSMB10	CYC1	2	PSMC5	AFG2B	2	PSMC3	DPAGT1	2	PSMA4	PGK1	2	PSMC3	TAMM41	3	PSMC4	ARCN1	3	PSMB8	RPL18	4	PSMC1	CWC22
1	PSMB8	CYCS	2	PSMD1	AFG2B	2	PSMC4	DPAGT1	2	PSMB2	PGK1	2	PSMD1	TAMM41	3	PSMB9	ATP1A1	3	PSMB8	RPL5	4	PSMD8	CWC22
1	PSME1	CYCS	2	PSMD13	AFG2B	2	PSMC5	DPAGT1	2	PSMC1	PGK1	2	PSMD13	TAMM41	3	PSMB10	ATP6V1B2	3	PSMB8	RRM1	4	PSMD8	DHX8
1	PSME2	CYCS	2	PSMD8	AFG2B	2	PSMD1	DPAGT1	2	PSMC4	PGK1	2	PSMD2	TAMM41	3	PSMB8	CAD	3	PSMB9	RUVBL1	4	PSMC1	EIF4A3
1	PSMB10	DCLRE1B	2	PSMD8	AIFM1	2	PSMD11	DPAGT1	2	PSMC5	PGK1	2	PSMD8	TAMM41	3	PSMB7	CCND1	3	PSMB10	SAP30BP	4	PSMD8	EIF4A3
1	PSMB9	DCLRE1B	2	PSMA1	AIRIM	2	PSMD13	DPAGT1	2	PSMD1	PGK1	2	PSMB7	TFRC	3	PSMD8	CCND1	3	PSMC5	SAP30BP	4	PSMC1	GBF1
1	PSME1	DCLRE1B	2	PSMA2	AIRIM	2	PSMD2	DPAGT1	2	PSMD11	PGK1	2	PSMB8	TFRC	3	PSMA4	CDC23	3	PSMB8	SCAP	4	PSMD8	GBF1
1	PSME2	DCLRE1B	2	PSMA4	AIRIM	2	PSMD8	DPAGT1	2	PSMD13	PGK1	2	PSMA4	TIMM10	3	PSMC4	CDC6	3	PSMD8	SCAP	4	PSMD8	HMGCR
1	PSME3	DCLRE1B	2	PSMA5	AIRIM	2	PSME3	DPAGT1	2	PSMD2	PGK1	2	PSMB2	TIMM10	3	PSMB10	CDK4	3	PSMB9	SLC35B2	4	PSMD8	HMGCS1
1	PSME3	DDX21	2	PSMA6	AIRIM	2	PSMG3	DPAGT1	2	PSMD8	PGK1	2	PSMB9	TIMM10	3	PSME3	CDKN1A	3	PSMB11	SNAP23	4	PSMB11	IK
1	PSMB8	DLD	2	PSMB11	AIRIM	2	PSMG4	DPAGT1	2	PSME2	PGK1	2	PSMC1	TIMM10	3	PSMB9	CFLAR	3	PSME2	SNAPC3	4	PSMC1	IK
1	PSMB8	DSCC1	2	PSMB2	AIRIM	2	PSMD8	DPH6	2	PSMD8	PGS1	2	PSMC4	TIMM10	3	PSMB10	CHMP4B	3	PSMB10	SNRPB2	4	PSMC4	IK
1	PSME3	DSCC1	2	PSMB3	AIRIM	2	PSMB7	DTYMK	2	PSMD8	PKM	2	PSMC5	TIMM10	3	PSMB11	CHMP4B	3	PSMC5	SNRPB2	4	PSMD1	IK
1	PSMB8	EMC6	2	PSMB4	AIRIM	2	PSMA4	EARS2	2	PSMD8	POLR1C	2	PSMD1	TIMM10	3	PSMB8	CHMP4B	3	PSMD5	SNRPB2	4	PSMD2	IK
1	PSMB10	EXOSC4	2	PSMB7	AIRIM	2	PSMB8	EARS2	2	PSMB10	POLR1E	2	PSMD11	TIMM10	3	PSMB10	COPA	3	PSMB11	SNRPD1	4	PSMD8	IK
1	PSMB8	GTF3C6	2	PSMB8	AIRIM	2	PSMB7	EP300	2	PSMD8	POLR1E	2	PSMD13	TIMM10	3	PSMD9	COPA	3	PSME3	SNRPD1	4	PSMD8	KIN
1	PSMB10	H2AX	2	PSMC1	AIRIM	2	PSMB2	FARSB	2	PSMC1	POLR3H	2	PSMD2	TIMM10	3	PSME2	COPA	3	PSME3	SPC24	4	PSMD8	NAPA
1	PSME1	H2AX	2	PSMC2	AIRIM	2	PSMC1	FARSB	2	PSMC4	POLR3H	2	PSMD8	TIMM10	3	PSMB7	COPG1	3	PSMB10	STX5	4	PSMD8	NEDD1
1	PSME2	H2AX	2	PSMC3	AIRIM	2	PSMD8	FARSB	2	PSMD11	POLR3H	2	PSMG4	TIMM10	3	PSMD8	COPG1	3	PSMB8	TADA1	4	PSMD8	NKAP
1	PSMB8	H2AZ1	2	PSMC4	AIRIM	2	PSMC1	FDXR	2	PSMD2	POLR3H	2	PSMD8	TONSL	3	PSMB7	CRKL	3	PSMB8	TAF5L	4	PSMD8	NUP160
1	PSME2	H2AZ1	2	PSMC5	AIRIM	2	PSMC5	FDXR	2	PSMD8	POLR3H	2	PSMA4	TPI1	3	PSMC4	DAD1	3	PSMB10	TARS1	4	PSMD8	NUP205
1	PSMB8	H2BC11	2	PSMC6	AIRIM	2	PSMD13	FDXR	2	PSMA4	POP5	2	PSMB10	TPI1	3	PSMG3	DAD1	3	PSMB5	THAP1	4	PSMD8	NUS1
1	PSMB8	H2BC4	2	PSMD1	AIRIM	2	PSMD8	FDXR	2	PSMA5	POP5	2	PSMB8	TPI1	3	PSMB11	DDX3X	3	PSMC4	TICRR	4	PSMC1	PLK1
1	PSMB8	H4C9	2	PSMD11	AIRIM	2	PSMB11	FEN1	2	PSMC1	POP5	2	PSMC5	TPI1	3	PSMB10	DNAJC9	3	PSMD8	TICRR	4	PSMD8	PLK1
1	PSMB10	HAMP	2	PSMD12	AIRIM	2	PSMD8	FEN1	2	PSMC3	POP5	2	PSMD11	TPI1	3	PSME2	DNAJC9	3	PSMB10	TINF2	4	PSME3	PLK1
1	PSME3	HAPSTR1	2	PSMD13	AIRIM	2	PSMB7	FPGS	2	PSMC4	POP5	2	PSMD13	TPI1	3	PSMB9	DNM2	3	PSMD5	TP53	4	PSMD8	POLE
1	PSMB10	HEATR1	2	PSMD14	AIRIM	2	PSMD8	GAPDH	2	PSMC5	POP5	2	PSMD8	TPI1	3	PSMB10	DYNC1H1	3	PSME1	TP53	4	PSMB9	POLR2I
1	PSMB8	HUS1	2	PSMD2	AIRIM	2	PSMD8	GEMIN6	2	PSMD1	POP5	2	PSME2	TPI1	3	PSMD9	EEF1B2	3	PSME3	TP53	4	PSMD8	POLR2I
1	PSME3	IPO9	2	PSMD3	AIRIM	2	PSMD8	GET1	2	PSMD11	POP5	2	PSMC3	TRIAP1	3	PSME2	EEF1B2	3	PSME4	TP53	4	PSMD8	PPP1R12A
1	PSMB8	IRF4	2	PSMD6	AIRIM	2	PSMD8	GET3	2	PSMD13	POP5	2	PSMD1	TRIAP1	3	PSMB11	EEFSEC	3	PSMC4	TP53RK	4	PSMC1	RAN
1	PSMB9	IRF4	2	PSMD8	AIRIM	2	PSMC4	GGPS1	2	PSMD3	POP5	2	PSMD8	TRIAP1	3	PSMB11	EIF2S2	3	PSMB8	TSC2	4	PSMD8	RAN
1	PSMB10	JAZF1	2	PSME2	AIRIM	2	PSMD11	GGPS1	2	PSMD8	POP5	2	PSMC1	TRMT112	3	PSMB11	EIF6	3	PSMB8	TTK	4	PSMB10	RBBP6
1	PSME3	KIF18B	2	PSME3	AIRIM	2	PSMD8	GGPS1	2	PSMG3	POP5	2	PSMD8	TRMT112	3	PSMB10	ESPL1	3	PSME1	TUBB	4	PSMB8	RBBP6
1	PSME3	MAPK1	2	PSMG1	AIRIM	2	PSMD8	GINS2	2	PSMC4	PPA1	2	PSMD8	TRMT61A	3	PSMB11	FAM133B	3	PSME4	TUBB	4	PSMD8	RBBP6
1	PSMB10	MARS1	2	PSMG2	AIRIM	2	PSMB7	GMPS	2	PSMD2	PPA1	2	PSMD8	TUBE1	3	PSMB8	FCF1	3	PSMB9	TXNL4A	4	PSME1	RBBP6
1	PSMB8	MBNL1	2	PSMG3	AIRIM	2	PSME2	GMPS	2	PSMD8	PPA1	2	PSMD8	TWNK	3	PSMB7	FERMT2	3	PSMB10	UBE2M	4	PSMD8	RBM25
1	PSMB10	MCM6	2	PSMG4	AIRIM	2	PSMD8	GNB1L	2	PSMB8	PPA2	2	PSMB7	UMPS	3	PSMF1	FERMT2	3	PSMD9	UBE2M	4	PSMD8	RBM8A
1	PSMB8	MCM6	2	PSMC1	ALG13	2	PSMD8	GTF2H4	2	PSMD8	PPA2	2	PSMB7	UROD	3	PSME2	GPN1	3	PSME2	UBE2M	4	PSMD8	RPA1
1	PSMB10	MDM2	2	PSMD2	ALG13	2	PSMB7	GUK1	2	PSMB11	PPP4C	2	PSMA4	UTP4	3	PSME1	GPN2	3	PSMB8	UBL5	4	PSMD8	RRM2
1	PSME1	MDM2	2	PSMD8	ALG13	2	PSMD8	GUK1	2	PSMD8	PPP4C	2	PSMB11	UTP4	3	PSMB11	GPX4	3	PSMB9	UBL5	4	PSMD8	SF3A1
1	PSME2	MDM2	2	PSMD8	ARMC7	2	PSMD8	HARS2	2	PSMB2	PPWD1	2	PSMB2	UTP4	3	PSMB11	GRB2	3	PSMA2	VCP	4	PSMD8	SF3A3
1	PSME3	MDM2	2	PSMB7	ATIC	2	PSMD8	HSCB	2	PSMC4	PPWD1	2	PSMB9	UTP4	3	PSMB7	GRB2	3	PSMC3	VCP	4	PSMD8	SF3B1
1	PSME4	MDM2	2	PSMB7	ATP1B3	2	PSMD8	HSD17B10	2	PSMD8	PPWD1	2	PSMD1	UTP4	3	PSMB11	GSPT1	3	PSMC4	VPS28	4	PSMC1	SF3B3
1	PSME1	MDM4	2	PSMB8	ATP1B3	2	PSMD8	HSPA14	2	PSMA4	PTPMT1	2	PSMD11	UTP4	3	PSMB10	GTF2E2	3	PSMA4	WEE1	4	PSMD8	SF3B3
1	PSME3	MDM4	2	PSMA1	BUB3	2	PSME3	HSPA14	2	PSMD8	PTPMT1	2	PSMD13	UTP4	3	PSMC4	HJURP	3	PSMB10	WEE1	4	PSMD8	SF3B5
1	PSMB8	MRPL15	2	PSMA2	BUB3	2	PSMD8	HSPD1	2	PSMD8	PWP2	2	PSMD8	UTP4	3	PSMB11	HNRNPH1	3	PSME2	WEE1	4	PSMC1	SFPQ
1	PSMB10	MRPS18C	2	PSMA4	BUB3	2	PSMD8	KDM8	2	PSMB11	RAD9A	2	PSMA4	WDR18	3	PSMD9	HNRNPH1	3	PSMB9	WNK1	4	PSMD8	SFPQ
1	PSMB8	MRPS18C	2	PSMA5	BUB3	2	PSMD8	LARS2	2	PSMD8	RAD9A	2	PSMB7	WDR18	3	PSMA4	HNRNPK	3	PSMB7	YRDC	4	PSMD8	SLU7
1	PSME3	MRTO4	2	PSMB2	BUB3	2	PSMD8	LETM1	2	PSMD8	RARS2	2	PSMB9	WDR18	3	PSMB8	HNRNPK	3	PSMD2	YRDC	4	PSMA2	SMU1
1	PSMB10	NCAPD3	2	PSMB8	BUB3	2	PSMA1	LONP1	2	PSMD8	RBBP4	2	PSMC1	WDR18	3	PSMD1	HNRNPK	3	PSMD5	YRDC	4	PSMA6	SMU1
1	PSME3	NCAPD3	2	PSMC1	BUB3	2	PSMA4	LONP1	2	PSMD8	RFC2	2	PSMC3	WDR18	3	PSMA4	HSPA5	3	PSMF1	YRDC	4	PSMC1	SMU1
1	PSME3	NCAPG2	2	PSMC2	BUB3	2	PSMA5	LONP1	2	PSMA2	RPL12	2	PSMC4	WDR18	3	PSMD8	HSPA5	3	PSMB10	ZEB2	4	PSMD1	SMU1
1	PSMB10	NOL7	2	PSMC3	BUB3	2	PSMB11	LONP1	2	PSMD13	RPL12	2	PSMC5	WDR18	3	PSME3	INTS6	3	PSMB8	ZNF217	4	PSMD8	SMU1
1	PSMB8	PDCD2	2	PSMC4	BUB3	2	PSMB2	LONP1	2	PSMD8	RPL12	2	PSMD1	WDR18	3	PSMA4	ISCU				4	PSME1	SMU1
1	PSME3	PDCD2	2	PSMC5	BUB3	2	PSMC1	LONP1	2	PSME3	RPL12	2	PSMD11	WDR18	3	PSMB7	ITGAV				4	PSMD8	SNRPA1
1	PSME2	PHF12	2	PSMC6	BUB3	2	PSMC2	LONP1	2	PSMA1	RPP21	2	PSMD13	WDR18	3	PSMF1	ITGAV				4	PSMC1	SNRPF
1	PSME3	POLE3	2	PSMD1	BUB3	2	PSMC3	LONP1	2	PSMA2	RPP21	2	PSMD2	WDR18	3	PSMB10	KEAP1				4	PSMD8	SNRPF
1	PSMB10	POLR1F	2	PSMD11	BUB3	2	PSMC4	LONP1	2	PSMA4	RPP21	2	PSMD8	WDR18	3	PSMB9	KEAP1				4	PSMD8	SPC25
1	PSMB8	POU2AF1	2	PSMD12	BUB3	2	PSMD1	LONP1	2	PSMA5	RPP21	2	PSMC1	WDR77	3	PSMB7	KIF18A				4	PSMD8	SRSF3
1	PSMB10	PPARGC1B	2	PSMD13	BUB3	2	PSMD11	LONP1	2	PSMA6	RPP21	2	PSMC4	WDR77	3	PSMB8	KIF18A				4	PSMB11	TSG101
1	PSMB8	PPCDC	2	PSMD2	BUB3	2	PSMD13	LONP1	2	PSMB11	RPP21	2	PSMC5	WDR77	3	PSMD5	KIF18A				4	PSMD8	TSG101
1	PSME1	PPM1D	2	PSMD8	BUB3	2	PSMD2	LONP1	2	PSMC1	RPP21	2	PSMD8	WDR77	3	PSMC3	KRAS				4	PSMD8	TUT1
1	PSME3	PPM1D	2	PSMG3	BUB3	2	PSMD8	LONP1	2	PSMC2	RPP21	2	PSMB8	WDR82	3	PSMD2	KRAS				4	PSMD8	UPF1
1	PSME3	PPM1G	2	PSMB8	CCDC115	2	PSMD8	LYRM4	2	PSMC3	RPP21	2	PSMD8	WDR82	3	PSMB10	LSM6				4	PSMD8	USPL1
1	PSME3	PPP2R2A	2	PSMD13	CCDC115	2	PSMA4	MARS2	2	PSMC4	RPP21	2	PSMD8	XRN2	3	PSMB8	MAGOH				4	PSMC1	XAB2
1	PSMB8	PRRC1	2	PSMD8	CCDC115	2	PSMA5	MARS2	2	PSMC5	RPP21	2	PSMA5	YARS2	3	PSMB10	MAT2A				4	PSMC4	XAB2
1	PSME2	RAD51D	2	PSMB7	CDAN1	2	PSMB11	MARS2	2	PSMD1	RPP21	2	PSMB7	YARS2	3	PSMB8	MED30				4	PSMD1	XAB2
1	PSMB8	RPL11	2	PSMD8	CDAN1	2	PSMB7	MARS2	2	PSMD11	RPP21	2	PSMB8	YARS2	3	PSMB9	MMS22L				4	PSMD11	XAB2
1	PSMB10	RPL19	2	PSMC1	CDIPT	2	PSMB8	MARS2	2	PSMD13	RPP21	2	PSMD8	YARS2	3	PSMB10	MRPL33				4	PSMD2	XAB2
1	PSME1	RPL36AL	2	PSMC4	CDIPT	2	PSMC3	MARS2	2	PSMD2	RPP21	2	PSMD8	ZBTB8OS	3	PSMB8	MRPL33				4	PSMD8	XAB2
1	PSME3	RPL4	2	PSMD8	CDIPT	2	PSMC5	MARS2	2	PSMD3	RPP21				3	PSMB11	MRPL41				4	PSMD8	YPEL5
1	PSMB10	RPS12	2	PSMD8	CENPA	2	PSMD8	MARS2	2	PSMD8	RPP21				3	PSMB11	MRPL43				4	PSMD8	ZNF207
1	PSMB8	SH3GL1	2	PSMD8	CIAO1	2	PSMD8	METTL1	2	PSMG4	RPP21				3	PSMB8	MSTO1				4	PSMC1	ZNF830
1	PSME1	SOX10	2	PSMC1	CINP	2	PSMD8	METTL16	2	PSMC1	RTCB				3	PSME2	NFS1				4	PSMD8	ZNF830
1	PSME3	SOX10	2	PSMC4	CINP	2	PSMD8	MRE11	2	PSMD8	RTCB				3	PSME1	NHLRC2				NA	NA	NA
1	PSMB8	SUPT7L	2	PSMD1	CINP	2	PSMB7	MRPL21	2	PSMB7	SAE1				3	PSME2	NHLRC2				NA	NA	NA
1	PSMB10	TEDC1	2	PSMD13	CINP	2	PSMD8	MRPL21	2	PSMD8	SAE1				3	PSMB9	NOP58				NA	NA	NA
1	PSMB10	UBE2D3	2	PSMD8	CINP	2	PSMD9	MRPL21	2	PSMB7	SDHB				3	PSMD5	NSF				NA	NA	NA
1	PSME1	UBE2D3	2	PSMC1	CPSF4	2	PSMD8	MRPS23	2	PSMB7	SDHC				3	PSMA4	NUF2				NA	NA	NA
1	PSME2	UBE2D3	2	PSMD8	CPSF4	2	PSMD8	MRPS6	2	PSME2	SDHC				3	PSMB10	NUF2				NA	NA	NA
1	PSME3	UBE2D3	2	PSMD8	DARS2	2	PSMA4	MYC	2	PSMD8	SLC25A26				3	PSMB8	NUF2				NA	NA	NA
1	PSME1	USP7	2	PSMC1	DBR1	2	PSMB8	MYC	2	PSMB9	SOD1				3	PSMC5	NUF2				NA	NA	NA
1	PSME3	USP7	2	PSMC4	DBR1	2	PSMD8	MYC	2	PSMC1	SOD1				3	PSMB8	NUP54				NA	NA	NA
1	PSMB10	VDAC1	2	PSMD8	DBR1	2	PSMD9	MYC	2	PSMC4	SOD1				3	PSMB9	NXF1				NA	NA	NA
1	PSMB8	VPS52	2	PSMG3	DBR1	2	PSMD8	N6AMT1	2	PSMD2	SOD1				3	PSMB9	PABPN1				NA	NA	NA
1	PSMB10	XRCC3	2	PSMD8	DDX49	2	PSMB7	NAMPT	2	PSMD8	SOD1				3	PSMB8	PET117				NA	NA	NA
1	PSMB10	ZNF674	2	PSMD8	DHPS	2	PSMD8	NELFB	2	PSME1	SOD1				3	PSME1	PHF5A				NA	NA	NA
1	PSME1	ZNF674	2	PSMA4	DHX33	2	PSMC1	NIP7	2	PSMG4	SOD1				3	PSMB8	POLR3B				NA	NA	NA
NA	NA	NA	2	PSMC3	DHX33	2	PSMC4	NIP7	2	PSMA4	SOD2				3	PSME2	POLR3F				NA	NA	NA

Supplementary Table S14. List of siRNA Sequence in this study

	Sequence
**siPSMB5-1**	GAG AAG UAU AGU GGC UCU A=tt (AS)<br>UAG AGC CAC UAU ACU UCU C=tt (AA)
**siPSMB5-2**	GAG UCA UAG UUG CAG CUG A=tt (AS)<br>UCA GCU GCA ACU AUG ACU C=tt (AA)
**siPSMB5-3**	CAG UCA ACC UCU ACC ACG U=tt (AS)<br>ACG UGG UAG AGG UUG ACU G=tt (AA)
**siPSMB6-1**	CGA GUG ACU GAC AAG CUG A=tt (AS)<br>UCA GCU UGU CAG UCA CUC G=tt (AA)
**siPSMB6-2**	ACA ACC ACU GGG UCC UAC A=tt (AS)<br>UGU AGG ACC CAG UGG UUG U=tt (AA)
**siPSMB6-3**	CGA UAC CGG GAA GAC CUG A=tt (AS)<br>UCA GGU CUU CCC GGU AUC G=tt (AA)

Supplementary Table S15. List of quantitative PCR primers in this study

Target name	Forward (5'→3')	Reverse (5'→3')
**GAPDH**	GAGTCCACTGGCTCTTCAC	ATGACGAACATGGGGGCATC
**PSMB5**	GTGTCCCAGAAGAGCCAGGAA	TCTTCACCGTCTGGGAGGCAA
**PSMB6**	CGTTCACTCCAGACTGGGAAAG	CGGTCGTGAATAGGTGTCAGCT
